# Bidirectional skeletal remodelling of SF_5_-nitrobenzenes into azepine, bicyclic, and benzimidazole frameworks†

**DOI:** 10.1039/d6sc01441k

**Published:** 2026-03-20

**Authors:** Muhamad Zulfaqar Bacho, Shiwei Wu, Takuya Muramatsu, Chavakula Nagababu, Daiki Harano, Seishu Ochiai, Norio Shibata

**Affiliations:** a Department of Nanopharmaceutical Sciences, Nagoya Institute of Technology Gokiso, Showa-ku Nagoya 466-8555 Japan nozshiba@nitech.ac.jp; b Department of Engineering, Nagoya Institute of Technology Gokiso, Showa-ku Nagoya 466-8555 Japan

## Abstract

The development of PFAS-free fluorinated scaffolds that preserve the desirable physicochemical attributes of perfluoroalkyl groups remains a central challenge in contemporary fluorine chemistry. Herein, we report a rapid and bidirectional skeletal-remodelling platform that enables controlled interconversion between aromatic, medium-sized, and bicyclic SF_5_-containing heterocycles from readily accessible SF_5_-nitrobenzenes. Phosphorus-catalyzed iterative deoxygenation of SF_5_-nitrobenzenes generates arylnitrene intermediates that undergo remarkably accelerated dearomative ring expansion, furnishing seven-membered SF_5_-azepines within dramatically shortened reaction times compared to non-SF_5_ analogues. These azepines function as versatile skeletal nodes, enabling divergent downstream transformations: photoinduced 4π-electrocyclization provides access to previously unexplored SF_5_-azabicyclo[3.2.0]hepta-2,6-diene frameworks, while selective fluoroacylative activation promotes reverse skeletal reconstruction to restore aromaticity and deliver SF_5_-substituted benzimidazoles. Collectively, this work demonstrates that strategic incorporation of the SF_5_ group not only expands accessible heterocyclic architectures but also fundamentally alters skeletal rearrangement kinetics, enabling rapid and controllable skeletal editing from a common, practical precursor. Given the OECD classification of SF_5_-containing molecules as non-PFAS, this unified skeletal-remodelling approach substantially broadens the design space of fluorinated scaffolds for applications in pharmaceuticals, agrochemicals, and functional materials, advancing the principles of sustainable fluorine chemistry.

## Abstract 2

The development of PFAS-free fluorinated scaffolds that preserve the desirable physicochemical attributes of perfluoroalkyl groups remains a central challenge in contemporary fluorine chemistry.

## Graphical abstract

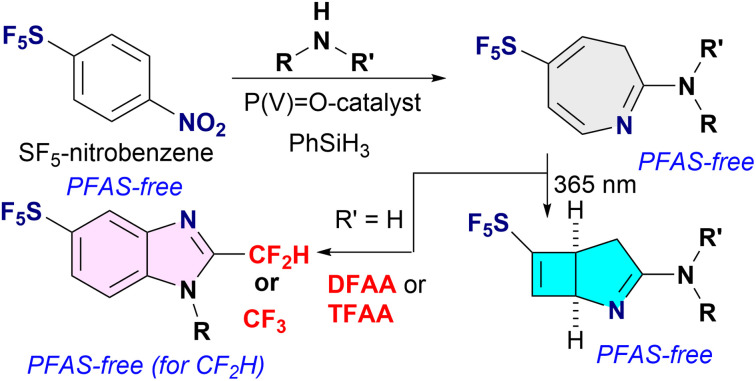

## Introduction

Fluorinated heterocycles are indispensable motifs in agrochemicals, pharmaceuticals, and functional materials, as fluorine substitution profoundly enhances lipophilicity, metabolic stability, and target engagement.^1^ In agrochemical design, CF_3_-substituted heterocycles are particularly dominant, accounting for more than 40% of all fluorinated agrochemicals.^1*b*^ However, CF_3_ groups are classified as per- and polyfluoroalkyl substances (PFAS) under the OECD definition, and their extreme environmental persistence has become a serious global concern.^2^ The exceptional stability of C–F bonds leads to long-term accumulation and the formation of highly mobile degradation products such as trifluoroacetic acid (TFA, CF_3_CO_2_H).^3^ While CF_3_ groups remain manageable in pharmaceuticals,^1*a*^ their intentional environmental release *via* agrochemicals^1*b*,3^ is increasingly unsustainable. Consequently, the development of PFAS-free fluorinated alternatives that retain the functional advantages of CF_3_ substituents has emerged as a critical challenge.

**Fig. 1:**
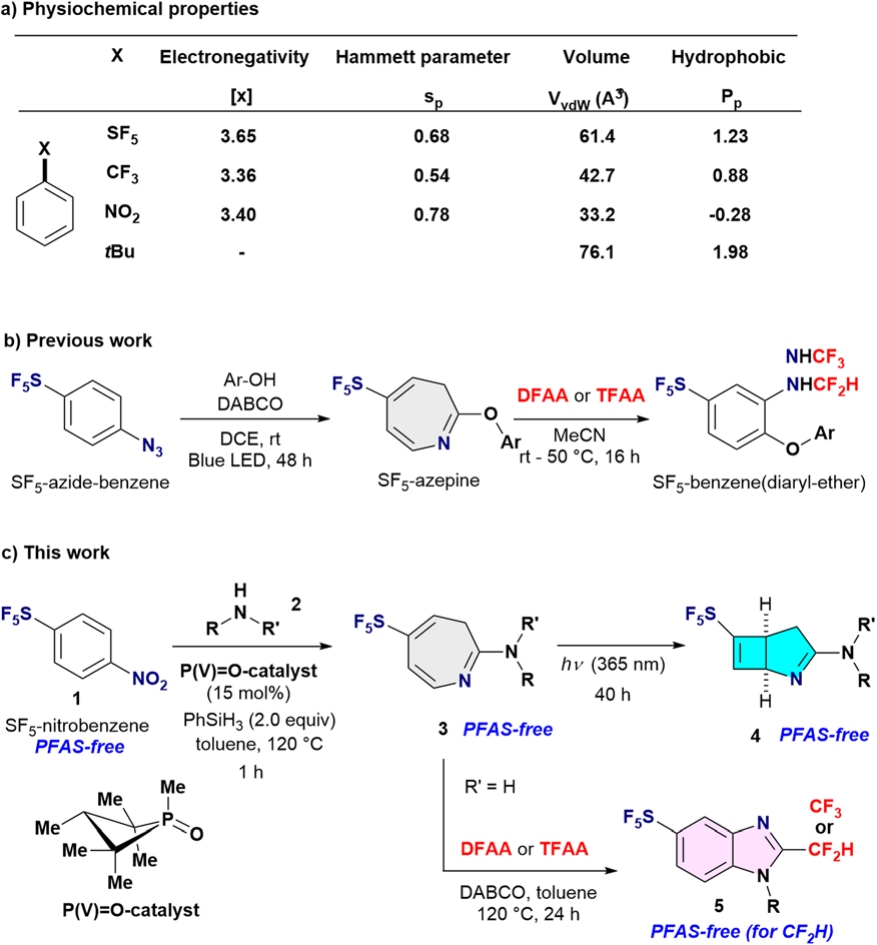
Background of this work. (a) Properties of SF_5_*vs.* CF_3_, NO_2_ and *t*Bu-benzenes. (b) Previous work (c) This work.

The pentafluorosulfanyl (SF_5_) group represents a compelling solution.^4,5^ As a hypervalent sulfur–fluorine functionality, SF_5_ combines strong electron-withdrawing character, high lipophilicity, and substantial steric bulk, and is therefore widely regarded as a “super-CF_3_” substituent. Importantly, unlike CF_3_, SF_5_ does not rely on persistent C–F bonds and exhibits the potential for environmentally benign mineralization, positioning SF_5_-containing molecules as classified as PFAS-free.^3*d*^ In molecular design, SF_5_ functions as a bioisostere for CF_3_, *tert*-butyl, and nitro groups, with comparable hydrophobicity and intrinsic volume (Fig. 1a). Despite these advantages, the structural diversity of SF_5_-containing molecules—particularly SF_5_-heterocycles—remains severely limited.^5*c*^

This limitation arises from a fundamental synthetic bottleneck. Traditional SF_5_ installation typically relies on radical reactions with alkenes or alkynes using SF_5_Cl or related reagents, which significantly limits substrate scope.^5*h*^ In contrast, oxidative chloro-fluorination of aryl sulfides and related protocols^4*b*^ require harsh reaction conditions, specialized equipment, and stringent moisture control, rendering their application to the synthesis of SF_5_-heterocycles highly limited.^6^ As a result, SF_5_ chemistry has remained largely limited to simple alkyl, alkenyl, and alkynyl motifs. Only recently have new classes of SF_5_ reagents or new protocols been developed,^7^ expanding the diversity of accessible substrates and reactivity profiles. Nevertheless, structurally complex heterocycles—particularly those incorporating SF_5_ or SF_4_ (ref. 8)—remain largely unexplored and synthetically inaccessible.

Concurrently, skeletal rearrangements have emerged as a powerful strategy for heterocycle synthesis, enabling rapid access to strained and unconventional architectures that are difficult to construct using classical cyclization methods.^9^ We recognized that this framework-reorganization approach is uniquely suited to SF_5_ chemistry, where conventional ring-construction strategies are intrinsically constrained. Consistent with this concept, we recently reported the photo-induced synthesis of SF_5_-azepine aryl ethers from SF_5_-azido benzene with phenols *via* skeletal rearrangement, thereby establishing this approach as a viable entry to medium-sized SF_5_-heterocycles (Fig. 1b).^10^ Notably, the strong electron-withdrawing nature of the SF_5_ substituent was found to facilitate the key ring-expansion step from benzene to azepine frameworks. Encouraged by these findings, we herein extend this concept to sequential skeletal remodelling, enabling access to a range of previously inaccessible SF_5_-containing heterocycles, including SF_5_-azepin-2-amines 3, SF_5_-azabicyclo[3.2.0]hepta-2,6-dienes 4, and SF_5_-benzimidazoles 5. In particular, the SF_5_-azabicyclo[3.2.0]hepta-2,6-diene derivatives represent the first examples of this rigid bicyclic scaffold bearing an SF_5_ substituent (Fig. 1c). Importantly, the synthesis originates from readily available SF_5_-nitrobenzenes 1, rather than inherently energetic SF_5_-azido benzenes, and proceeds through consecutive skeletal rearrangements. These transformations establish a unified skeletal-editing manifold that interconverts aromatic, medium-sized, and bicyclic SF_5_-containing architectures.

## Results and discussion

### Development of a rapid skeletal rearrangement to amino-SF_5_-azepines

**Fig. 2:**
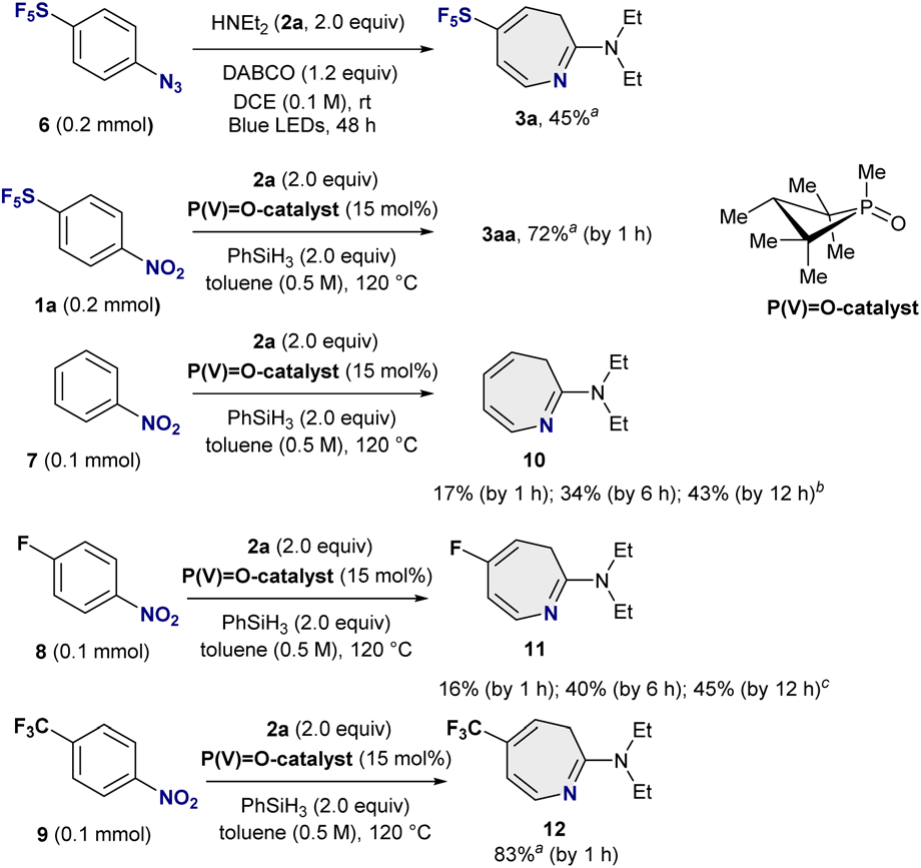
Skeletal rearrangement to amino-SF_5_-azepines. ^*a*^Yield was determined by ^19^F-NMR spectroscopy (fluorobenzene as standard). ^*b*^Yields were determined by ^1^H-NMR spectroscopy (1,3,5-trimethylbenzene as standard). ^*c*^Yields were determined by ^19^F-NMR spectroscopy (benzotrifluoride as standard).

Seven-membered SF_5_-heterocycles are essentially unexplored, despite the prevalence of azepine frameworks in biologically active molecules and marketed drugs.^11^ Given our interest in 2-amino-SF_5_-azepines, we first examined the skeletal remodelling of SF_5_-azido benzene 6 using diethylamine 2a as the nucleophile in the presence of DABCO, following our reported LED conditions (Fig. 2).^10^ Under these conditions, the desired 2-diethylamino-SF_5_-azepine 3a was obtained in 45% yield after 48 h at room temperature, confirming the feasibility of azepine formation from SF_5_-azido benzene, albeit with limited efficiency and prolonged reaction times. We next turned to an alternative nitrene-generation strategy based on phosphorus-catalyzed deoxygenation of SF_5_-nitrobenzene, as developed by Radosevich and co-workers.^12^ Under these conditions, phenylnitrene are generated thermally *via*P(iii)/P(v)=O redox cycling from P(V)=O-catalyst with phenylsilane. Pleasingly, application of this protocol to SF_5_-nitrobenzene 1a proved markedly more effective, delivering the corresponding SF_5_-azepine 3a in 72% yield within only 1 h at 120 °C. Notably, in non-SF_5_ systems, analogous nitrobenzene-to-azepine conversions under Radosevich conditions typically require approximately 12 h to reach completion.^12^ To further probe the origin of this pronounced rate enhancement, we evaluated nitrobenzene 7 and fluoro-substituted nitrobenzene 8 under identical conditions. In both cases, the corresponding azepin-2-amines (10 and 11) were obtained in only 16–17% yield after 1 h, with yields increasing gradually to 43–45% after 12 h. In sharp contrast, the SF_5_-substituted nitrobenzene underwent rapid and efficient skeletal rearrangement, suggesting that the strongly electron-withdrawing SF_5_ substituent significantly accelerates the ring-expansion process, likely by facilitating arylnitrene insertion and subsequent electrocyclic rearrangement.^10^ Given this substantial rate enhancement and the improved safety and practicality of using SF_5_-nitrobenzenes instead of SF_5_-azido benzenes, we undertook a systematic investigation of the phosphorus-catalyzed conversion of SF_5_-nitrobenzenes into SF_5_-azepines. A similar acceleration in the ring-expansion was observed for trifluoromethyl-substituted nitrobenzene 9 under standard conditions, affording azepine-2-amine 12 in 83% yield within 1 h, compared to 12 h in the reported method.^12^

### Substrate scope for skeletal rearrangement to amino-SF_5_-azepines

**Scheme 1:**
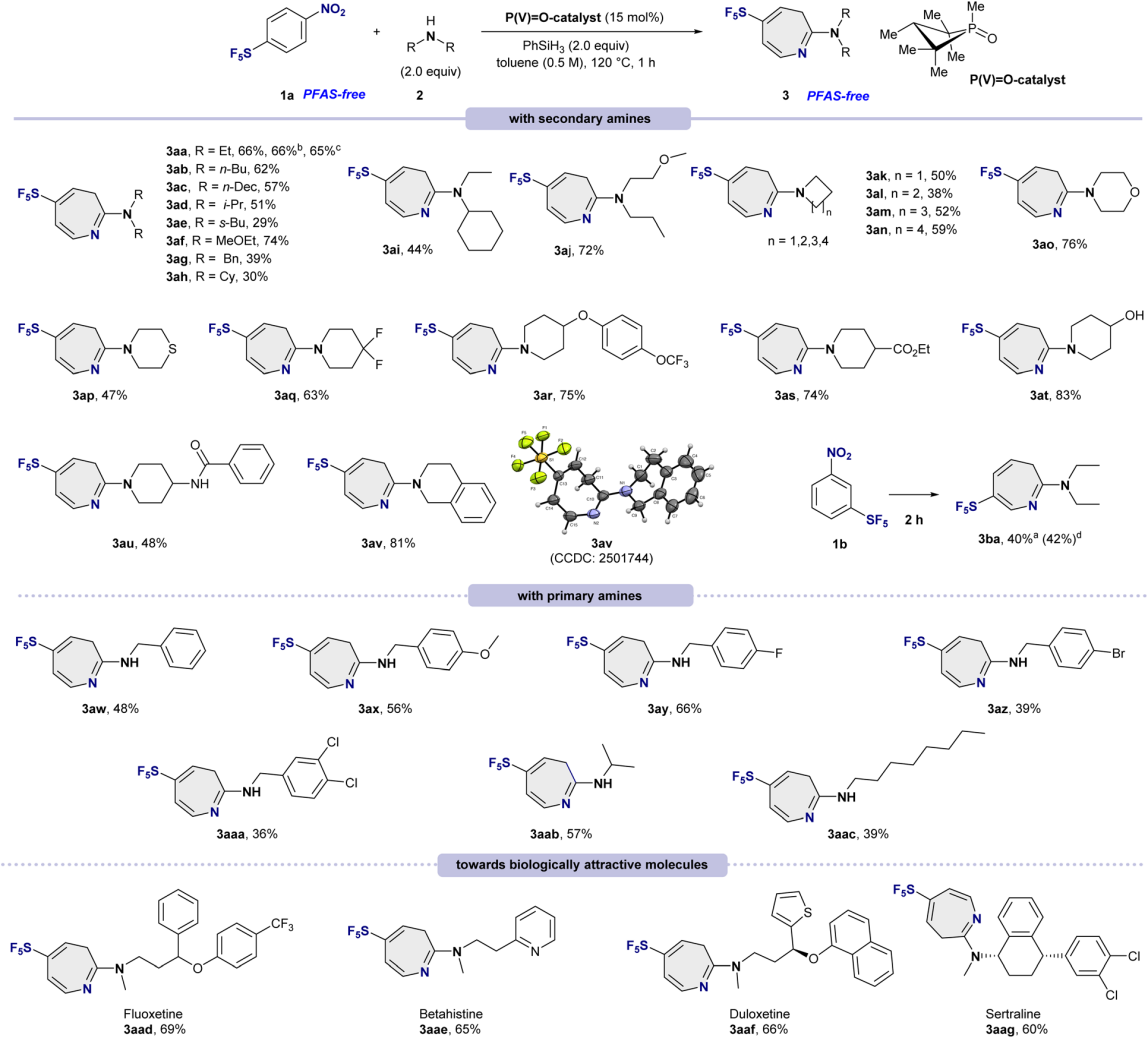
Substrate scope for skeletal rearrangement to amino-SF_5_-azepines. ^*a*^Unless otherwise noted, the reactions were carried out with 1a (0.2 mmol, 1.0 equiv.), 2 (0.4 mmol, 2.0 equiv.), P(V)=O-catalyst (15 mol%), PhSiH_3_ (0.4 mmol, 2.0 equiv.), in toluene (0.4 mL, 0.5 M) stirred at 120 °C for 1 hour. Isolated yields are given. ^*b*^A mixture of 1a (0.1 mmol, 1.0 equiv.), 2a (0.2 mmol, 2.0 equiv.), P(V)=O-catalyst (15 mol%), PhSiH_3_ (0.2 mmol, 2.0 equiv.), in toluene (0.2 mL, 0.5 M) stirred at 120 °C for 1 hour. Isolated yields are given. ^*c*^A mixture of 1a (4.0 mmol, 1.0 equiv., 1.0 g), 2a (8.0 mmol, 2.0 equiv.), P(V)=O-catalyst (15 mol%), PhSiH_3_ (8.0 mmol, 2.0 equiv.), in toluene (8.0 mL, 0.5 M) stirred at 120 °C for 1 hour. Isolated yields are given. ^*d*^Yield was determined by ^19^F-NMR spectroscopy (fluorobenzene as standard).

The generality of the phosphorus-catalyzed ring-expansion reaction was first evaluated with a broad range of acyclic secondary amines (Scheme 1, top). Straight-chain dialkylamines such as diethylamine (2a), dibutylamine (2b), and didecylamine (2c) were smoothly converted into the corresponding azepines 3aa–3ac in 57–66% yields. Branched secondary amines were also compatible, although increased steric demand led to somewhat diminished efficiencies, affording products 3ad and 3ae in 29–51% yields. An electron-rich ether-substituted amine (2f) was well tolerated, delivering 3af in 74% yield. Aromatic and alicyclic secondary amines, including dibenzylamine (2g), dicyclohexylamine (2h), and unsymmetrical amines (2i–2j), furnished azepines 3ag–3aj in 30–72% yields. The influence of cyclic amine ring size was next evaluated. Four-, five-, six-, and seven-membered cyclic amines (2k–2n) participated successfully, affording azepines 3ak–3an in moderate yields (38–59%). Notably, heteroatom-containing cyclic amines also proved to be competent nucleophiles: morpholine (2o), thiomorpholine (2p), and difluorinated piperidine (2q) delivered products 3ao–3aq in good to excellent yields (47–76%). In addition, a complex aryloxy-substituted piperidine (2r) underwent smooth coupling to afford 3ar in 75% yield, highlighting the functional-group tolerance of the process.

To further assess the generality of the ketenimine intermediate derived from 1a, we investigated nucleophiles bearing diverse functional groups. Esters (2s), alcohols (2t), amides (2u), and bicyclic amines (2v) were all successfully incorporated, furnishing products 3as–3av in 48–83% yields. The structure of 3av was unambiguously confirmed by single-crystal X-ray diffraction (CCDC 2501744). Notably, this transformation is applicable not only to *p*-substituted SF_5_-nitrobenzene 1a but also to *m*-substituted analogue 1b, which afforded the regioselective product 3ba in 40% isolated yield.

Encouraged by the breadth of secondary amine compatibility, we extended the methodology to primary amines (Scheme 1, middle). Benzylamines bearing either electron-donating or electron-withdrawing substituents at the *para* position (2w–2aa) underwent efficient ring expansion to give products 3aw–3aaa in 36–66% yields. Primary aliphatic amines (2ab and 2ac) were likewise competent substrates, affording azepines 3aab and 3aac in 39–57% yields.

Finally, the synthetic utility of this transformation was demonstrated through late-stage functionalization of biologically relevant amines (Scheme 1, bottom). Complex drug-derived amines, including fluoxetine (2ad), betahistine (2ae), duloxetine (2af), and sertraline (2ag), were successfully converted into the corresponding SF_5_-azepine derivatives 3aad–3aag in good yields (60–69%). These results underscore the robustness of the method and its potential for late-stage diversification in medicinal and agrochemical discovery.

Anilines were not suitable as amine nucleophiles; instead of azepines 3, N–N bond coupling products were formed, consistent with previous reports.^13^

### Second skeletal rearrangement to bicyclic SF_5_- azabicyclo[3.2.0]hepta-2,6-dienes

**Scheme 2:**
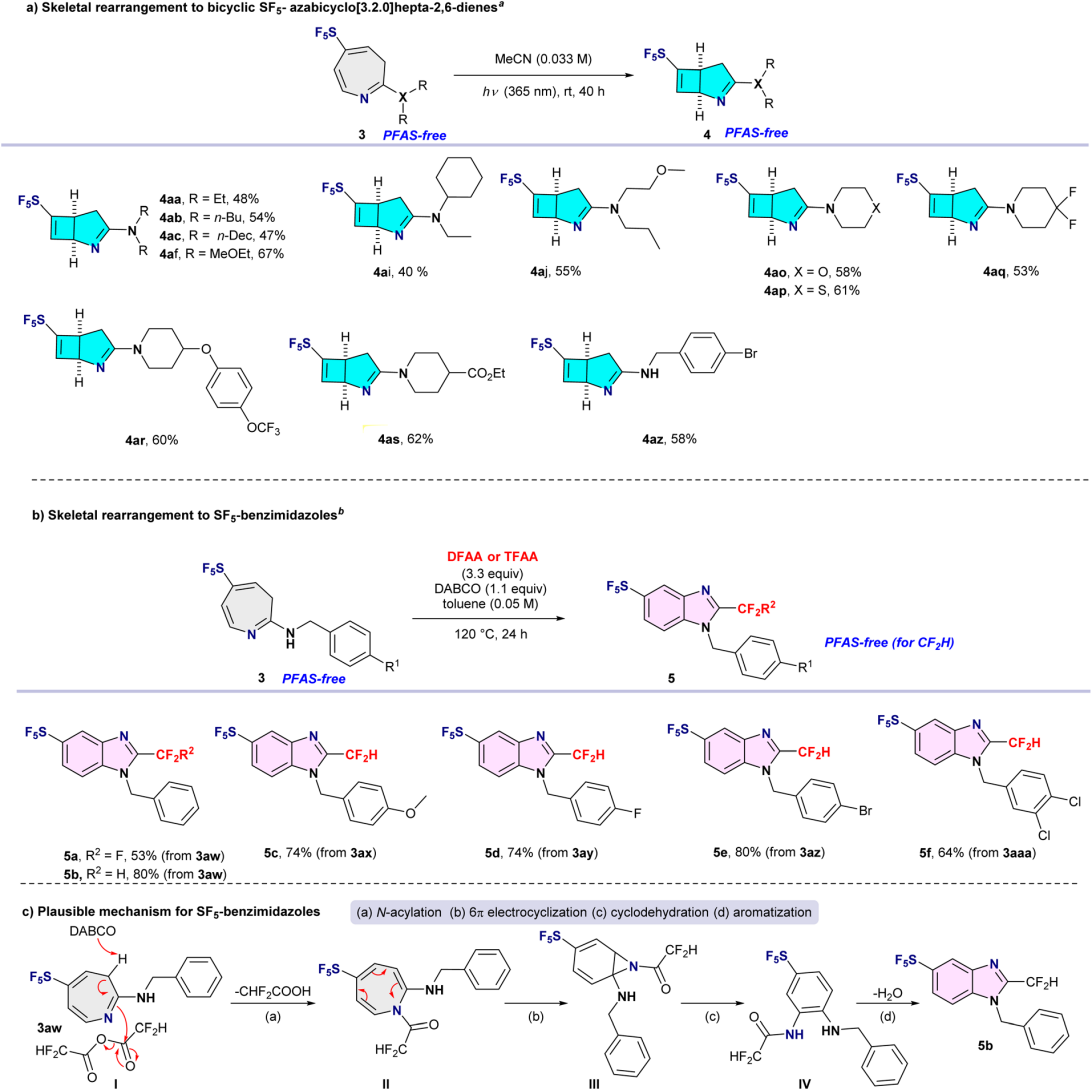
Skeletal editing of azepines: transformations to bicyclic SF_5_-azabicyclo[3.2.0]hepta-2,6-dienes and SF_5_-benzimidazoles ^*a*^Reaction conditions: 3 (0.1 mmol, 1.0 equiv.) in MeCN (3.0 mL, 0.033M) irradiated under (365 nm) at rt for 40 hours. Isolated yields are given. ^*b*^Reaction conditions: 3 (0.1 mmol, 1.0 equiv.), DABCO (0.11 mmol, 1.1 equiv.), TFAA or DFAA (0.33 mmol, 3.3 equiv.) in toluene (2.0 mL, 0.05 M) at 120 °C for 24 hours. Isolated yields are given.

With gram-scale quantities of azepine 3aa available, we next investigated the photochemical 4π-electrocyclization developed by Leonori^14^ for the synthesis of bicyclic SF_5_-attached azabicyclo[3.2.0]hepta-2,6-dienes (Scheme 2a, top). Under simple irradiation of azepines 3 at 365 nm in acetonitrile at room temperature cleanly delivered the corresponding bicyclic products, SF_5_-attached azabicyclo[3.2.0]hepta-2,6-dienes 4 in moderate to good yields. SF_5_-azepines 3 bearing electron-donating alkyl substituents, including ethyl (3aa), *n*-butyl (3ab), *n*-decyl (3ac), and 2-methoxyethyl (3af) groups, were well tolerated, affording bicyclic derivatives 4aa, 4ab, 4ac, and 4af in yields of up to 67%. Azepines containing unsymmetrical secondary amines (3ai and 3aj) also underwent smooth skeletal rearrangement to furnish bicyclic products 4ai and 4aj in 40–55% yields. Notably, this photochemical transformation proved compatible with cyclic amine-substituted azepines: substrates 3ao–3as were efficiently converted into the corresponding bicyclic compounds 4ao–4as in consistently good yields (53–62%). Furthermore, SF_5_-azepines bearing primary amine substituents, exemplified by 4-bromobenzylamine-derived azepine 3az, participated smoothly to afford bicyclic product 4az in 58% yield. The relative stereochemistry of the bicyclic products 4 was established by detailed 2D NMR analysis of compound 4az (see SI) and was found to be fully consistent with previously reported azabicyclo[3.2.0] frameworks.^14^

### Third skeletal rearrangement to SF_5_-benzimidazoles

To further probe the structural plasticity of the SF_5_-azepine framework and to demonstrate bidirectional skeletal editing from a common intermediate, we investigated the re-aromatization of SF_5_-azepines to SF_5_-benzimidazoles. Following a protocol related to that reported by Radosevich and co-workers,^12^ a primary amine-substituted azepine (3aw, derived from benzylamine) was treated with trifluoroacetic anhydride (TFAA) in the presence of DABCO in toluene at 120 °C for 24 h. Under these conditions, re-aromatization of the azepine core was accompanied by intramolecular cyclodehydration, affording the corresponding SF_5_-benzimidazole 5a in 53% yield (Scheme 2b, middle). This cyclodehydration is plausibly driven by electrophilic activation of the azepine nitrogen by the acyl anhydride, followed by intramolecular nucleophilic attack of the pendant amine and subsequent loss of water to restore aromaticity (Scheme 2c, bottom)

Because the CF_3_ group is classified as a PFAS motif, we next examined the transformation using difluoroacetic anhydride (DFAA) under otherwise identical conditions. As anticipated, the reaction proceeded efficiently to deliver the CF_2_H-substituted SF_5_-benzimidazole 5b in 80% yield. Notably, this skeletal reconstruction pathway proved tolerant of diverse substituents on the aromatic ring, including OMe, F, Br, and Cl, furnishing a series of CF_2_H-substituted SF_5_-benzimidazoles (5c–5f) in good to excellent yields.

## Conclusions

This study demonstrates that skeletal rearrangement of SF_5_-nitrobenzenes provides a general and efficient strategy for constructing diverse SF_5_-heterocycles, including SF_5_-azepines, SF_5_-azabicyclo[3.2.0] frameworks, and SF_5_-benzimidazoles. Importantly, these motifs represent PFAS-free alternatives to CF_3_-based scaffolds, offering structurally rich and functionally attractive building blocks for pharmaceuticals, agrochemicals, and materials science. In light of the OECD classification of SF_5_-containing molecules as non-PFAS, this work contributes to the advancement of sustainable fluorine chemistry by demonstrating that strategic skeletal remodelling can unlock both reactivity and chemical space unique to SF_5_ substitution.

## Author contributions

MZB optimized the reaction conditions, surveyed the substrate scope, analyzed the data and discussed the results with NS. SW, TM, and CN prepared starting materials and attempted reactions. DH helped to prepare the starting materials. SO took the X-ray crystallography of 3av. MZB wrote the initial draft and NS wrote the manuscript. NS supervised the study. All authors contributed to the manuscript and approved the final version of the manuscript.

## Conflicts of interest

There are no conflicts to declare.

## Supplementary Material

SC-017-D6SC01441K-s001

SC-017-D6SC01441K-s002

## Data Availability

CCDC 2501744 contains the supplementary crystallographic data for this paper.^15^ The data that support the findings of this study are available within the article and the supplementary information (SI). Supplementary information: materials and methods, experimental procedures, characterization data, and NMR spectra. See DOI: https://doi.org/10.1039/d6sc01441k.
