# Supplementary material for: Bidirectional skeletal remodelling of SF5-nitrobenzenes into azepine, bicyclic, and benzimidazole frameworks
Source: Chem Sci. 2026 Mar 20;17(21):10434–40. doi: 10.1039/d6sc01441k (PMC13085320; doi:10.1039/d6sc01441k)
Supplement: SC-017-D6SC01441K-s001 [file SC-017-D6SC01441K-s001.pdf]

Supplementary Information (SI) for Chemical Science.
This journal is © The Royal Society of Chemistry 2026

Supporting Information

Bidirectional skeletal remodelling of SF_5-nitrobenzenes into azepine, bicyclic, and benzimidazole frameworks

Muhamad Zulfaqar Bacho,[a] Shiwei Wu,[b] Takuya Muramatsu,[b] Chavakula Nagababu,[b] Daiki Harano,[b] Seishu Ochiai[b] and Norio Shibata*[a,b,]

[a]Department of Nanopharmaceutical Sciences, Nagoya Institute of Technology, Gokiso, Showa-Ku, Nagoya 466-8555, Japan
[b]Department of Engineering, Nagoya Institute of Technology Gokiso, Showa-ku, Nagoya 466-8555, Japan

*E-mail: nozshiba@nitech.ac.jp.

Table of Contents

1. Materials and General Information S2
2. Optimization of Reaction Conditions S3
   2.1. Optimization of reaction conditions for pentafluoro(4-nitrophenyl)-λ^6-sulfane (**1a**) and amine (**2a**) S3
3. Experimental Section S8
   3.1. General Procedures S8
   3.2. Characterization S12
4. 2D NMR of Compound (**4az**) S29
5. References S30
6. X-ray crystallography data (**3av**) S31
7. NMR Spectral Data S33

1. Materials and General Information

Toluene and THF were purchased from commercial sources and dried by passage through an activated alumina column under N_2 and degassed prior use. Anhydrous solvents (ethyl acetate, 1,4-dioxane, DMF, DMSO, 1,2-DCE and MeCN) were purchased from commercial sources and degassed prior use. *t*-BuOAc, *m*-xylene, CPME, *i*-PrOH were purchased from commercial sources and degassed prior use. All liquid amines were degassed using Freeze-Pump-Thaw method before use. All liquid phosphorus catalysts were degassed using Freeze-Pump-Thaw method before use. Chemicals were purchased from commercial sources and used without further purification.

All reactions were performed using oven-dried glassware and were performed under positive pressure of nitrogen unless otherwise mentioned. All the reactions were monitored by thin-layer chromatography (TLC) carried out on 0.25 mm Merck silica gel (60-F_{254}) or measurement of ^{19}F-NMR. The TLC plates were visualized with UV light. Products were purified by column chromatography carried out on columns packed with silica gel (60N spherical neutral size 63–210 μm). The ^{19}F-NMR (282 MHz, 376 MHz, 658 MHz, 659 MHz) spectra were recorded on a Varian Mercury 300, BRUKER AVANCE-400 and a JEOL RESONANCE ECZ700R. ^{1}H-NMR (500 MHz, 700 MHz) and ^{13}C-NMR (126 MHz, 176 MHz) spectra were recorded on BRUKER 500 Ultra Shield TR and a JEOL RESONANCE ECZ700R. Chemical shifts (δ) are expressed in ppm downfield from TMS (δ = 0.00 ppm) and $CDCl_3$ (δ = 7.26 ppm) for ^{1}H-NMR, and $CDCl_3$ (δ = 77.16 ppm) for ^{13}C-NMR or internal standard, C_6F_6 (δ = −162.2 ppm) and C_6H_5F (δ = −113.57 ppm) for ^{19}F-NMR. The High-resolution mass spectrometry (HRMS) was carried out on an electrospray ionization mass spectrometer (ESI) with a micro-TOF analyzer and recorded on a Waters (Model number: SELECT SERIES Cyclic IMS ACQUITY UPLC H-Class PLUS ACQUITY UPLC M-Class). Infrared spectra were recorded on a JASCO FT/IR-4100 spectrometer. Melting points were recorded on a BUCHI M-565. The EvoluChem PhotoRedOx Box™ was used as the photoreactor, and equipped with EvoluChem HCK1012-01-006, LED lighting (30 W, 365 nm) was used as the light source for irradiation and an operating fan (temperature ~34 °C).

2. Optimization of Reaction Conditions

2.1. Optimization of reaction conditions for pentafluoro(4-nitrophenyl)-λ^6-sulfane (1a) and amine (2a)

Table S1. Optimization of Reaction Time[a]

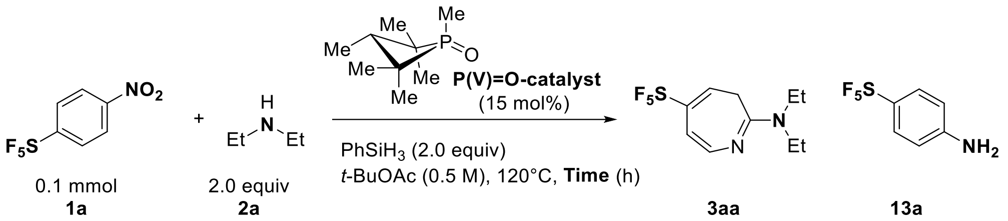

Entry	Time	Yield of 1a (%)[b]	Yield of 3aa (%)[b]	Yield of 13a (%)[b]
1	1 h	8	62	13
2	2 h	0	62	15
3	3 h	0	62	15
4	4 h	0	65	17
5	6 h	0	64	17
6	12 h	0	65	17
**7**	**16 h**	**0**	**70**	**19**
8	24 h	0	70	19

[a]**Reaction condition**: **1a** (0.1 mmol, 1.0 equiv), **2a** (0.2 mmol, 2.0 equiv), **P(V)=O-catalyst** (15 mol%), $PhSiH_3$ (0.2 mmol, 2.0 equiv), in *t*-BuOAc (0.2 mL, 0.5 M) stirred at 120 °C for **specified time**. [b]Determined by ^{19}F-NMR spectroscopy using fluorobenzene as internal standard.

Table S2. Optimization of Phosphorus Catalyst[a]

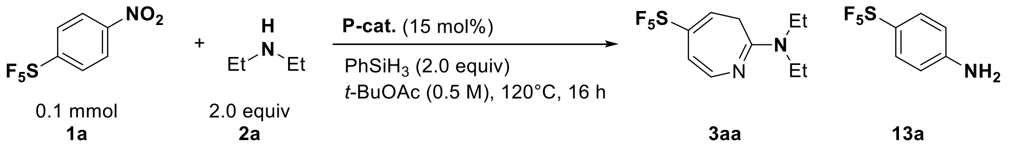

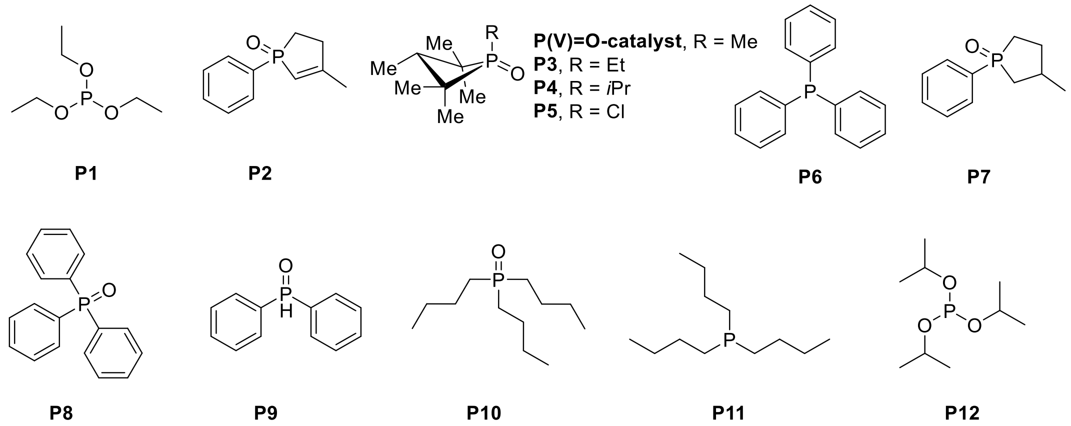

Entry	P-catalyst	Yield of 1a (%)[b]	Yield of 3aa (%)[b]	Yield of 13a (%)[b]
1	P1	90	4	3
2	P2	0	29	58
**3**	P(V)=O-catalyst	**0**	**70**	**19**
4	P3	0	64	11
5	P4	69	20	11
6	P5	5	50	45
7	P6	0	13	58
8	P7	0	40	50
9	P8	0	8	42
10	P9	81	11	6
11	P10	34	25	10
12	P11	34	26	27
13	P12	91	5	0

[a]**Reaction condition**: **1a** (0.1 mmol, 1.0 equiv), **2a** (0.2 mmol, 2.0 equiv), **P-cat.** (15 mol%), $PhSiH_3$ (0.2 mmol, 2.0 equiv), in *t*-BuOAc (0.2 mL, 0.5 M) stirred at 120 °C for 16 hours. [b]Determined by ^{19}F-NMR spectroscopy using fluorobenzene as internal standard

Table S3. Optimization of Silane[a]

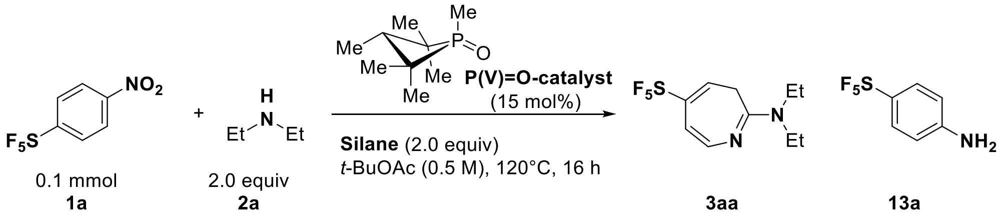

Entry	Phosphorus-catalyst	Yield of 1a (%)[b]	Yield of 3aa (%)[b]	Yield of 13a (%)[b]
1	PMHS (6 equiv)	0	60	15
2	Et_3SiH (6 equiv)	100	0	0
3	$Ph(CH_2)_2SiH$ (6 equiv)	97	traces	traces
4	Ph_3SiH (6 equiv)	9	19	68
5	Ph_2SiH_2 (3 equiv)	0	60	21
**6**	$PhSiH_3$ (2 equiv)	**0**	**70**	**19**

[a]**Reaction condition**: **1a** (0.1 mmol, 1.0 equiv), **2a** (0.2 mmol, 2.0 equiv), **P(V)=O-catalyst** (15 mol%), **silane** (0.2 mmol, 2.0 equiv), in *t*-BuOAc (0.2 mL, 0.5 M) stirred at 120 °C for 16 hours. [b]Determined by ^{19}F-NMR spectroscopy using fluorobenzene as internal standard.

Table S4. Optimization of Temperature[a]

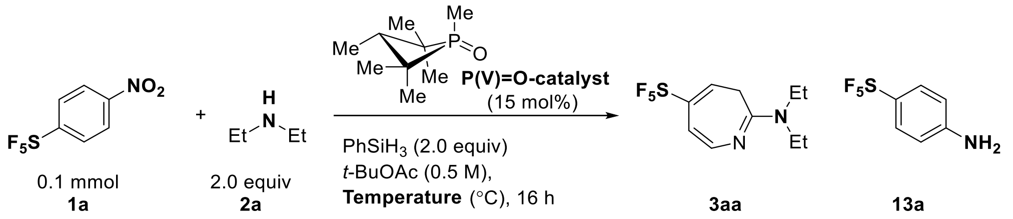

Entry	Temperature	Yield of 1a (%)[b]	Yield of 3aa (%)[b]	Yield of 13a (%)[b]
1	rt	40	18	38
2	50 °C	0	60	19
**3**	**120 °C**	**0**	**70**	**19**
4	135 °C	0	52	22
5	150 °C	0	18	25

[a]**Reaction condition**: **1a** (0.1 mmol, 1.0 equiv), **2a** (0.2 mmol, 2.0 equiv), **P(V)=O-catalyst** (15 mol%), $PhSiH_3$ (0.2 mmol, 2.0 equiv), in *t*-BuOAc (0.2 mL, 0.5 M) stirred at **specified temperature** for 16 hours. [b]Determined by ^{19}F-NMR spectroscopy using fluorobenzene as internal standard.

Table S5. Optimization of Amine Equivalent[a]

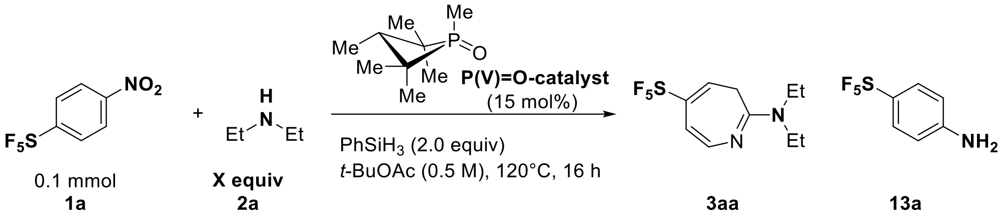

Entry	X (equiv)	Yield of 1a (%)[b]	Yield of 3aa (%)[b]	Yield of 13a (%)[b]
1	1.0	0	54	23
2	1.2	0	59	21
3	1.5	0	61	20
**4**	**2.0**	**0**	**70**	**19**
5	3.0	0	70	15
6	as solvent	0	41	18

[a]**Reaction condition**: **1a** (0.1 mmol, 1.0 equiv), **2a** (0.2 mmol, **X equiv**), **P(V)=O-catalyst** (15 mol%), $PhSiH_3$ (0.2 mmol, 2.0 equiv), in *t*-BuOAc (0.2 mL, 0.5 M) stirred at 120 °C for 16 hours. [b]Determined by ^{19}F-NMR spectroscopy using fluorobenzene as internal standard.

Table S6. Optimization of Solvent[a]

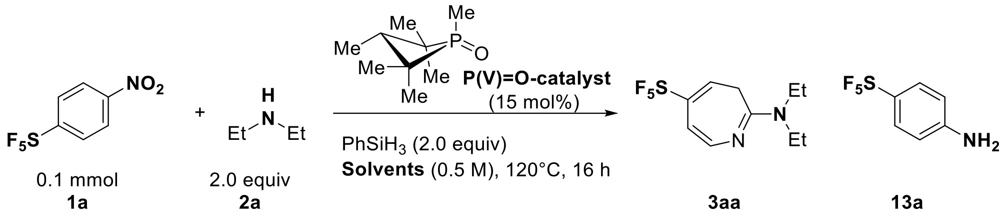

Entry	Solvents	Yield of 1a (%)[b]	Yield of 3aa (%)[b]	Yield of 13a (%)[b]
1	*t*-BuOAc	0	70	19
2	Dioxane	0	60	17
**3**	**Toluene**	**0**	**71**	**19**
4	DMF	0	0	0
5	DMSO	0	0	0
6	THF	0	37	34
7	EA	0	0	0
8	MeCN	0	0	0
9	CPME	0	63	19
10	*m*-xylene	0	65	18
11	*i*-PrOH	100	0	0

[a]**Reaction condition**: **1a** (0.1 mmol, 1.0 equiv), **2a** (0.2 mmol, 2.0 equiv), **P(V)=O-catalyst** (15 mol%), $PhSiH_3$ (0.2 mmol, 2.0 equiv), in **specified solvent** (0.2 mL, 0.5 M) stirred at 120 °C for 16 hours. [b]Determined by ^{19}F-NMR spectroscopy using fluorobenzene as internal standard.

Table S6. Optimization of Time[a]

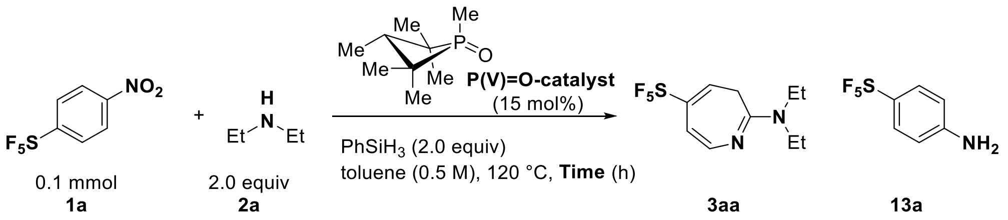

Entry	Time	Yield of 1a (%)[b]	Yield of 3aa (%)[b]	Yield of 13a (%)[b]
1	16	0	71	19
2	3	0	70	13
**3**	**1**	**0**	**71**	**13**
4	30	0	67	10

[a]**Reaction condition**: **1a** (0.1 mmol, 1.0 equiv), **2a** (0.2 mmol, 2.0 equiv), **P(V)=O-catalyst** (15 mol%), $PhSiH_3$ (0.2 mmol, 2.0 equiv), in toluene (0.2 mL, 0.5 M) stirred at 120 °C for **specified time**.
[b]Determined by ^{19}F-NMR spectroscopy using fluorobenzene as internal standard.

3. Experimental Section

3.1. General Procedures

General Procedure A: Preparation of pentafluoro(nitrophenyl)-λ^6-sulfane (1), Amine (2) and Phosphorus Catalyst P-cat.

a) pentafluoro(4-nitrophenyl)-λ^6-sulfane **1** were prepared according to the reported methods.[1,2]

1) pentafluoro(4-nitrophenyl)-λ^6-sulfane **1a** were prepared according to the reported methods.[1]

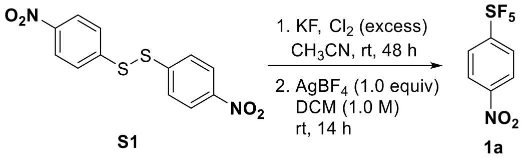

S1 1a

An oven-dried 250 mL PFA bottle with a magnetic stirring bar was charged with disulfide (**S1**, 6.17 g, 20 mmol), spray-dried KF (18.59 g, 320 mmol), and anhydrous acetonitrile (120 mL) inside the glove box. The bottle was then capped and sealed with parafilm and taken out from the glovebox. The bottle was cooled in an ice/water bath while chlorine gas was bubbled through the stirred reaction mixture for approximately 8 minutes. The bottle was sealed, and the reaction mixture was stirred at 0 °C for 3 hours. Stirring then continued at room temperature for 48 hours. After the reaction was completed, the solution was filtered under a nitrogen atmosphere to another 250 ml PFA bottle using a PP/ETFE filter. The residue was washed with MeCN (2.5 ml × 2). MeCN was evaporated in vacuo to give crude arylsulfur chlorotetrafluoride (9.17 g) 86% yield. The crude was directly used for the next step without further purification.

Crude arylsulfur chlorotetrafluoride (9.17 g, 34.5 mmol, 1.0 equiv) and $AgBF_4$ (6.72 g, 34.5 mmol, 1.0 equiv) were weighed into a PFA tube containing a magnetic stirrer bar in a glove box. Anhydrous dichloromethane (34.5 mL, 1.0 M) was added in the glovebox. The mixture was stirred at room temperature for 14 h outside the glovebox. After complete conversion, the reaction mixture was filtered through a pad of silica and washed with dichloromethane. The solvent was removed in vacuo to give the crude product, which was purified by chromatography on silica gel (eluted with pentane/DCM: 3/1) to afford the corresponding product **1a** as a white or pale-yellow solid (7.36 g, 86% yield).

2) pentafluoro(3-nitrophenyl)-λ^6-sulfane**1b** were prepared according to the reported methods.[1]

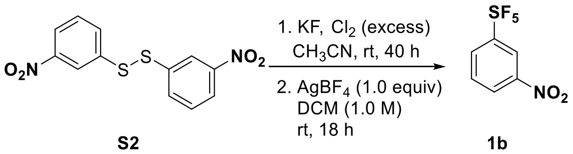

S2 1b

An oven-dried 250 mL PFA bottle with a magnetic stirring bar was charged with disulfide (**S2**, 4.41 g, 14.3 mmol), spray-dried KF (13.29 g, 228.8 mmol), and anhydrous acetonitrile (79 mL) inside the glove box. The bottle was then capped and sealed with parafilm and taken out from the glovebox. The bottle was cooled in an ice/water bath while chlorine gas was bubbled through the stirred reaction mixture for approximately 8 minutes. The bottle was sealed, and the reaction mixture was stirred at 0 °C for 3 hours. Stirring then continued at room temperature for 40 hours. After the reaction was completed, the solution was filtered under a nitrogen atmosphere to another 250 ml PFA bottle using a PP/ETFE filter. The residue was washed with MeCN (2.5 ml × 2). MeCN was evaporated in vacuo to give crude arylsulfur chlorotetrafluoride (5.98 g) 79% yield. The crude was directly used for the next step without further purification.

Crude arylsulfur chlorotetrafluoride (5.98 g, 22.5 mmol, 1.0 equiv) and $AgBF_4$ (4.38 g, 22.5 mmol, 1.0 equiv) were weighed into a PFA tube containing a magnetic stirrer bar in a glove box. Anhydrous dichloromethane (22.5 mL, 1.0 M) was added in the glovebox. The mixture was stirred at room

temperature for 18 h outside the glovebox. After complete conversion, the reaction mixture was filtered through a pad of silica and washed with dichloromethane. The solvent was removed in vacuo to give the crude product, which was purified by chromatography on silica gel (eluted with pentane/DCM: 3/1) to afford the corresponding product **1b** as a pale-yellow oil (4.52 g, 81% yield).

b) Amine **2a-2ag** were purchased and used as received.

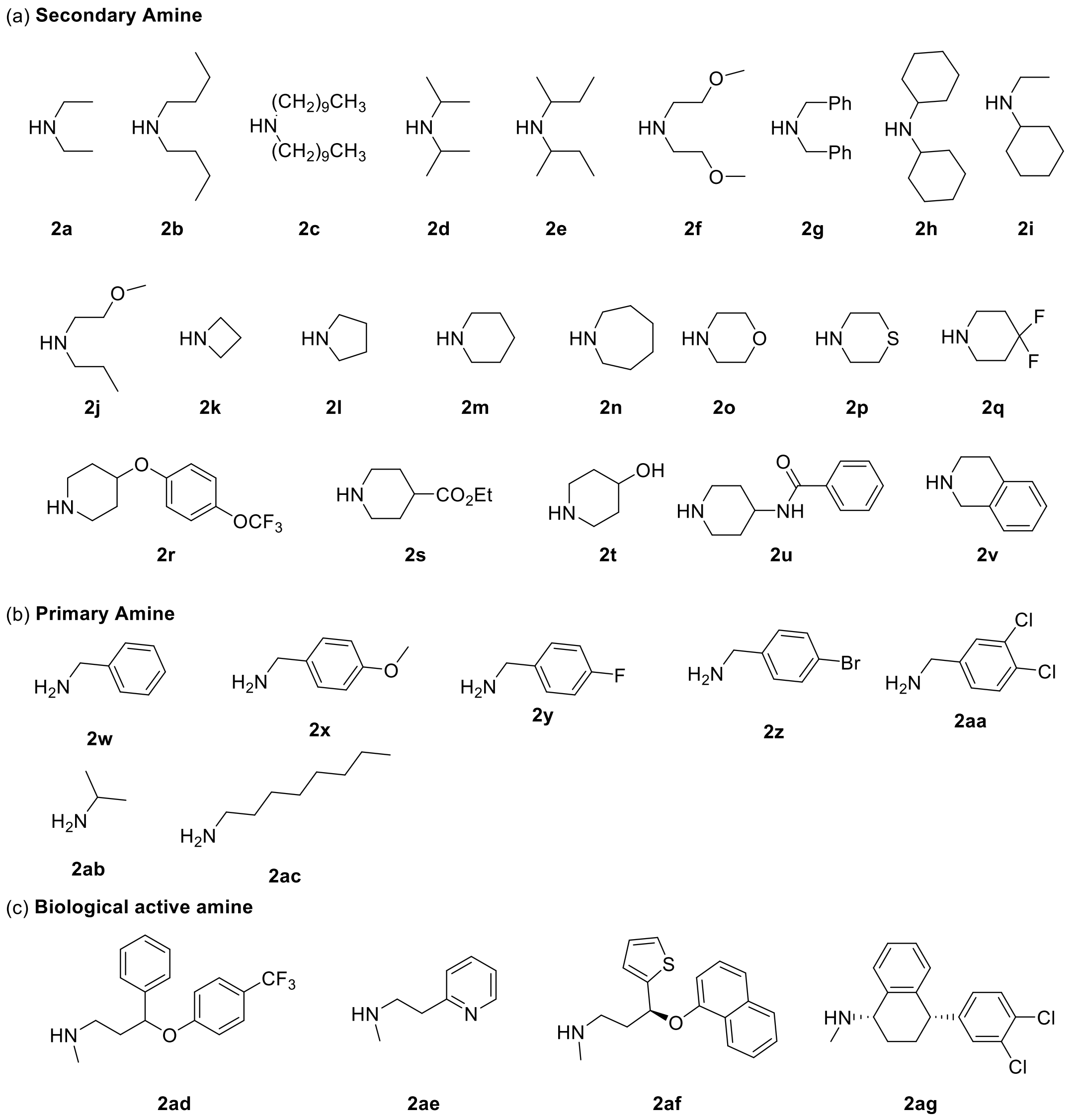

Figure S1. Structures of substrate amine **2**

c) Phosphorus catalysts (**P1**, **P2**, **P(V)=O-catalyst**, **P6**, **P7**, **P8**, **P19**, **P10**, **P11**, **P12**) were purchased and used as received. Phosphorus catalysts (**P3**,[2] **P4**,[2] **P5**[3] **and P7**[3]) are known and were prepared according to the reported methods.[2,3]

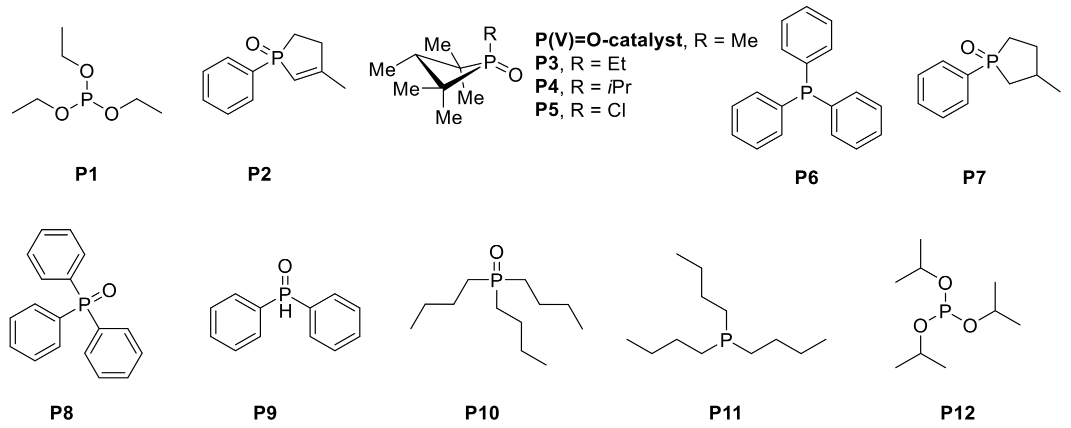

Figure S2. Structures of substrate Phosphorus catalysts **P-cat.**

General Procedure B: The Synthesis of Substituted 3*H*-azepin-2-amine (3)

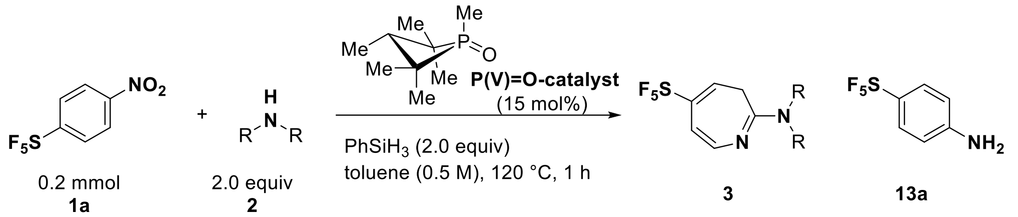

To an oven-dried screw-cap tube equipped with a small stir bar was added the nitroarene SF_5 **1a** (0.20 mmol, 1.0 equiv), phosphetane oxide precatalyst **P(V)=O-catalyst** (5.2 mg, 0.03 mmol, 15 mol%), anhydrous degassed toluene (0.4 mL, 0.5 M), degassed amine **2** (0.4 mmol, 2.0 equiv), and phenylsilane (49.2 µL, 0.4 mmol, 2.0 equiv) were then added sequentially inside the glovebox. The tube threads were wrapped with parafilm, and a phenolic screw-thread open-top cap fitted with a PTFE-lined silicone septum was secured. The reaction mixture was stirred at 120 °C for 1 hour. Upon completion, the crude residue was concentrated by rotary evaporation and purified directly by column chromatography. Columns were slurry-packed with hexanes/ethyl acetate, hexanes/DCM, hexanes/acetone, or a ternary mixture (hexanes/acetone/DCM), with mobile-phase polarity gradually increased as indicated.

General Procedure C: The Synthesis of Substituted 3*H*-azepin-2-amine (3)

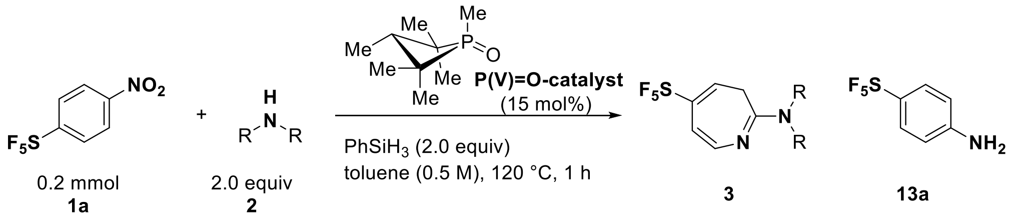

To a solution of amine hydrochloride **2** (1.5 mmol) dissolved in 25 mL of DCM was added 25 mL of a saturated solution of sodium hydrogen carbonate in DI water. After vigorous mixing, the layers were separated, and the aqueous layer was extracted with 25 mL of DCM. The combined organic layers were dried with sodium sulphate, filtered, and concentrated under reduced pressure to obtain the free-base of amine as a slightly yellow oil.[4] The reaction was performed by following **General Procedure B** on a reduced scale. To an oven-dried screw-cap tube equipped with a small stir bar was added the nitroarene SF_5 **1a** (0.20 mmol, 1.0 equiv), phosphetane oxide precatalyst **P(V)=O-catalyst** (5.2 mg, 0.03 mmol, 15 mol%), anhydrous degassed toluene (0.4 mL, 0.5 M), degassed amine **2** (0.4 mmol, 2.0 equiv), and phenylsilane (49.2 µL, 0.4 mmol, 2.0 equiv) were then added sequentially inside the glovebox. The tube threads were wrapped with parafilm, and a phenolic screw-thread open-top cap fitted with a PTFE-lined

silicone septum was secured. The reaction mixture was stirred at 120 °C for 1 hour. Upon completion, the crude residue was concentrated by rotary evaporation and purified directly by column chromatography. Columns were slurry-packed with hexanes/ethyl acetate, hexanes/DCM, hexanes/acetone, or a ternary mixture (*n*-hexanes/acetone/DCM), with mobile-phase polarity gradually increased as indicated.

General Procedure D: The synthesis of substituted bicyclic pyrroline (4)

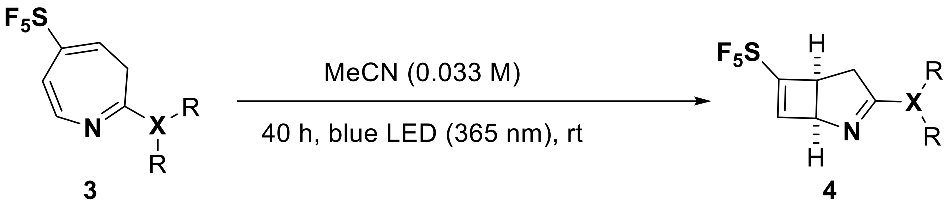

To an oven-dried screw-cap tube equipped with a small stir bar was charged with the 3*H*-azepin-2-amine **3** (0.1 or 0.15 mmol, 1.0 equiv.). The tube was capped with rubber septum, evacuated and refilled with argon (x 3). Freshly degassed MeCN (0.033 M) was added. The septum was then immediately replaced with a phenolic screw-thread open-top cap fitted with a PTFE-lined silicone septum was secured and the EvoluChem PhotoRedOx Box™ was used as the photoreactor (365 nm) and the fan was switched on and the reaction mixture was stirred under irradiation for 40 hours. The mixture evaporated, and the residue was purified via column chromatography (*n*-hexanes/acetone/ethy acetate) with triethyl amine to give the desired product **4**.

General Procedure E: The synthesis of substituted bicyclic pyrroline (5)

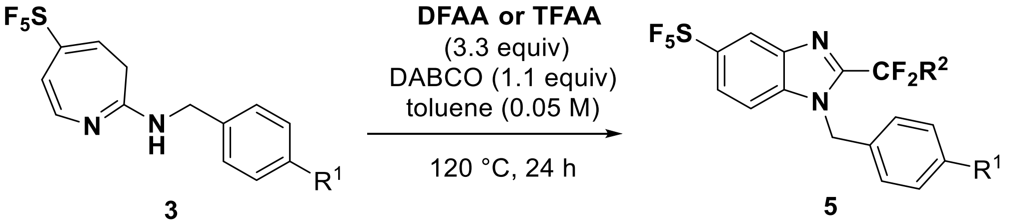

To an oven-dried screw-cap tube equipped with a small stir bar was added 3*H*-azepin-2-amine **3** (0.1 mmol, 1.0 equiv) and DABCO (12.3 mg, 0.11 mmol, 1.1 equiv). The vial was capped with a septum, evacuated and refilled with N_2 (x 3), the mixture was dissolved in toluene (2.0 mL, 0.05 M). Lastly, the trifluoroacetic anhydride or difluoroacetic anhydride (0.22 mmol, 2.2 equiv) was added to the reaction mixture and the reaction tube was stirred at 120 °C. After 6 hours, another 1.1 equiv of the trifluoroacetic anhydride or difluoroacetic anhydride was added to the reaction reaction vial for another 24 hours. (a little bit more equivalency of the corresponding trifluoroacetic anhydride or difluoroacetic anhydride need to be added if the quality is not great). Upon completion, 5 mL of ethyl acetate and 5 mL NaOH solution (1 M) were added followed by extraction with 3x5 mL ethyl acetate. After mixing and separating the organic layer, the combined organic layers were dried over anhydrous sodium sulfate, filtered and concentrated with the aid of a rotary evaporator. The crude residues were purified via column chromatography using *n*-Hexane/EtOAc (9/1) or *n*-Hexane/DCM (50/50) to obtained pure product **5**.

General Procedure F: Structural assignments
Structural assignments of **4k** were made with additional information from ^{1}H-^{1}H COSY, and NOESY experiments.

3.2. Characterization

***N,N*-diethyl-5-(pentafluoro-λ^6-sulfaneyl)-3*H*-azepin-2-amine (3aa)**

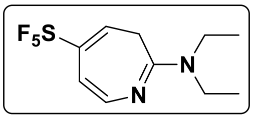

According to **General Procedure B**, pentafluoro(4-nitrophenyl)-λ^6-sulfane **1a** (49.8 mg, 0.2 mmol, 1.0 equiv) and phosphetane oxide **P(V)=O-catalyst** (5.2 mg, 0.03 mmol, 15 mol%) were dissolved in toluene. Then, amine **2a** (41.7 µL, 0.4 mmol, 2.0 equiv) and phenylsilane (49.2 µL, 0.4 mmol, 2.0 equiv) were added sequentially. The reaction mixture was stirred at 120 °C for 1 hour. **3aa** was obtained as a yellow oil (38.2 mg, 66%) using *n*-Hexane/EtOAc (80/20). **^{1}H-NMR** (500 MHz, $CDCl_3$) δ 7.23 (d, *J* = 8.3 Hz, 1H), 6.02 (dd, *J* = 8.4, 1.6 Hz, 1H), 5.65 (td, *J* = 8.0, 1.6 Hz, 1H), 3.39 (s, 4H), 1.16 (s, 6H). **^{13}C-NMR** (126 MHz, $CDCl_3$) δ 156.1 (quint, *J* = 14.0 Hz), 145.8, 143.1, 112.1 (p, *J* = 4.6 Hz), 103.9 (p, *J* = 3.8 Hz), 43.6, 30.0, 13.6 (d, *J* = 211.1 Hz). **^{19}F-NMR** (282 MHz, $CDCl_3$) δ 86.63 – 83.42 (m, 1F), 61.50 (d, *J* = 148.2 Hz, 4F). **HRMS** (ESI) calculated for $C_{10}H_{16}F_5N_2S$ $[M+H]^+$: 291.0954, found 291.0960. **IR (KBr)**: v = 2978, 2936, 1600, 1566, 1509, 1266, 1122, 1012, 836, cm^{-1}. The CH_2 in the azepine core was not observed in the ^{1}H NMR spectrum but was confirmed by ^{13}C NMR and HRMS analysis.

***N,N*-dibutyl-5-(pentafluoro-λ^6-sulfaneyl)-3*H*-azepin-2-amine (3ab)**

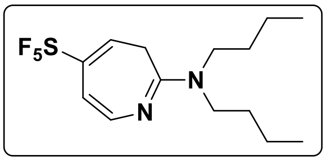

According to **General Procedure B**, pentafluoro(4-nitrophenyl)-λ^6-sulfane **1a** (49.8 mg, 0.2 mmol, 1.0 equiv) and phosphetane oxide **P(V)=O-catalyst** (5.2 mg, 0.03 mmol, 15 mol%) were dissolved in toluene. Then, amine **2b** (67.0 µL, 0.4 mmol, 2.0 equiv) and phenylsilane (49.2 µL, 0.4 mmol, 2.0 equiv) were added sequentially. The reaction mixture was stirred at 120 °C for 1 hour. **3ab** was obtained as a yellow oil (42.75 mg, 62%) using DCM/*n*-hexane (50/50 to 60/40). **^{1}H-NMR** (500 MHz, $CDCl_3$) δ 7.22 (d, *J* = 8.3 Hz, 1H), 6.02 (dd, *J* = 8.3, 1.5 Hz, 1H), 5.63 (td, *J* = 8.0, 1.6 Hz, 1H), 3.24 (s, 4H), 1.69 – 1.11 (m, 8H), 1.05 – 0.77 (m, 6H). **^{13}C-NMR** (126 MHz, $CDCl_3$) δ 156.1 (quint, *J* = 14.0 Hz), 146.1, 142.7, 111.8 (t, *J* = 4.8 Hz), 103.6 (t, *J* = 3.9 Hz), 49.2, 31.7, 30.3, 29.5, 20.2, 13.9. **^{19}F-NMR** (376 MHz, $CDCl_3$) δ 85.21 (quint, *J* = 148.4 Hz, 1F), 61.57 (d, *J* = 148.1 Hz, 4F). **HRMS** (ESI) calculated for $C_{14}H_{24}F_5N_2S$ $[M+H]^+$: 347.1580, found 347.1590. **IR (KBr)**: v = 2962, 2934, 2874, 1600, 1565, 1507, 1372, 1228, 1149, 1010, 924, 837 cm^{-1}. The CH_2 in the azepine core was not observed in the ^{1}H NMR spectrum but was confirmed by ^{13}C NMR and HRMS analysis.

***N,N*-didecyl-5-(pentafluoro-λ^6-sulfaneyl)-3*H*-azepin-2-amine (3ac)**

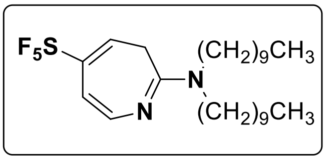

According to **General Procedure B**, pentafluoro(4-nitrophenyl)-λ^6-sulfane **1a** (49.8 mg, 0.2 mmol, 1.0 equiv) and phosphetane oxide **P(V)=O-catalyst** (5.2 mg, 0.03 mmol, 15 mol%) were dissolved in toluene. Then, amine **2c** (119.0 mg, 0.4 mmol, 2.0 equiv) and phenylsilane (49.2 µL, 0.4 mmol, 2.0 equiv) were added sequentially. The reaction mixture was stirred at 120 °C for 1 hour. **3ac** was obtained as a brown oil (58.4 mg, 57%) using *n*-Hexane/EtOAc (30/1) and *n*-Hexane/DCM (5/1). **^{1}H-NMR** ^{1}H NMR (500 MHz, $CDCl_3$) δ 7.22 (d, *J* = 8.3 Hz, 1H), 6.01 (dd, *J* = 8.3, 1.5 Hz, 1H), 5.62 (td, *J* = 8.0, 1.6 Hz, 1H), 3.22 (s, 4H), 1.26 (s, 32H), 0.88 (s, 6H). **^{13}C-NMR** (126 MHz, $CDCl_3$) δ 156.1 (quint, *J* = 13.8 Hz), 146.1, 142.8, 111.9 – 111.5 (m), 103.5, 49.4, 32.0, 30.3, 29.7, 29.5, 29.4, 27.4, 27.0, 22.8, 14.3. **^{19}F-NMR** (282 MHz, $CDCl_3$) δ 85.23 (quint, *J* = 148.1 Hz, 1F), 61.55 (d, *J* = 148.4 Hz, 4F). **HRMS** (ESI) calculated for $C_{26}H_{48}F_5N_2S$ $[M+H]^+$: 515.3458, found 515.3463. **IR (KBr)**: v = 2926, 2855, 1599, 1506, 1464, 1429, 1373, 1323, 1278, 1172, 1110, 840 cm^{-1}. The CH_2 in the azepine core was not observed in the ^{1}H NMR spectrum but was confirmed by ^{13}C NMR and HRMS analysis.

***N,N*-diisopropyl-5-(pentafluoro-λ^6-sulfaneyl)-3*H*-azepin-2-amine (3ad)**

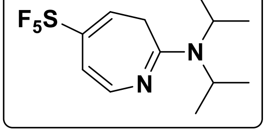

According to **General Procedure B**, pentafluoro(4-nitrophenyl)-λ^6-sulfane **1a** (49.8 mg, 0.2 mmol, 1.0 equiv) and phosphetane oxide **P(V)=O-catalyst** (5.2 mg, 0.03 mmol, 15 mol%) were dissolved in toluene. Then, amine **2d** (56.2 µL, 0.4 mmol, 2.0 equiv) and phenylsilane (49.2 µL, 0.4 mmol, 2.0 equiv) were added sequentially. The reaction mixture was stirred at 120 °C for 1 hour. **3ad** was obtained as a pale yellow oil (32.47 mg, 51%) using *n*-Hexane/DCM (50/50). **^{1}H-NMR** (500 MHz, $CDCl_3$) δ 7.21 (d, *J* = 8.2 Hz, 1H), 6.02 (dd, *J* = 8.2, 1.6 Hz, 1H), 5.49 (td, *J* = 8.1, 1.6 Hz, 1H), 4.04 (dd, *J* = 14.3, 7.4 Hz, 2H), 1.26 (s, 12H). **^{13}C-NMR** ^{13}C NMR (126 MHz, $CDCl_3$) δ 156.0 (quint,

J = 13.7 Hz), 143.6, 141.9, 112.05 (quint J = 4.6 Hz), 103.0 (quint, J = 3.7 Hz), 47.9, 32.8, 21.2. **^{19}F-NMR** ^{19}F NMR (282 MHz, cdcl$_3$) δ 85.40 (quint, 1F), 61.79 (d, J = 148.3 Hz, 4F). **HRMS** (ESI) calculated for $C_{12}H_{20}F_5N_2S$ [M+H]$^+$: 319.1267, found 319.1275. **IR (KBr)**: v = 2972, 2934, 1889, 1598, 1556, 1453, 1373, 1156, 1112, 1012, 982, 836 cm^{-1}. The CH_2 in the azepine core was not observed in the ^{1}H NMR spectrum but was confirmed by ^{13}C NMR and HRMS analysis.

***N,N*-di-*sec*-butyl-5-(pentafluoro-λ^6-sulfaneyl)-3*H*-azepin-2-amine (3ae)**

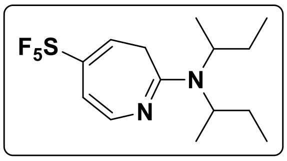

According to **General Procedure B**, pentafluoro(4-nitrophenyl)-λ^6-sulfane **1a** (49.8 mg, 0.2 mmol, 1.0 equiv) and phosphetane oxide **P(V)=O-catalyst** (5.2 mg, 0.03 mmol, 15 mol%) were dissolved in toluene. Then, amine **2e** (68.9 µL, 0.4 mmol, 2.0 equiv) and phenylsilane (49.2 µL, 0.4 mmol, 2.0 equiv) were added sequentially. The reaction mixture was stirred at 120 °C for 1 hour. **3ae** was obtained as a yellow oil (19.75 mg, 29%) using *n*-Hexane/acetone (90/10) and *n*-Hexane/DCM (80/20). **^{1}H-NMR** (500 MHz, $CDCl_3$) δ 7.19 (d, J = 8.2 Hz, 1H), 6.01 (dd, J = 8.2, 1.6 Hz, 1H), 5.50 (tdd, J = 8.2, 3.7, 1.5 Hz, 1H), 3.61 (d, 2H), 1.75 – 1.50 (m, 4H), 1.45 – 1.11 (m, 7H), 1.05 – 0.50 (m, 5H). **^{13}C-NMR** (126 MHz, $CDCl_3$) δ 155.9 (quint, J = 13.6 Hz), 143.7, 141.8, 112.3, 102.9, 55.1, 32.8, 28.8, 19.2, 12.0. **^{19}F-NMR** (376 MHz, $CDCl_3$) δ 85.41 (quint, J = 148.4 Hz, 1F), 61.73 (d, J = 148.4 Hz, 4F). **HRMS** (ESI) calculated for $C_{14}H_{24}F_5N_2S$ [M+H]$^+$: 347.1580, found 347.1587. **IR (KBr)**: v = 2970, 2877, 1597, 1556, 1460, 1375, 1196, 1110, 1071, 1010, 992, 881 cm^{-1}. The CH_2 in the azepine core was not observed in the ^{1}H NMR spectrum but was confirmed by ^{13}C NMR and HRMS analysis.

***N,N*-bis(2-methoxyethyl)-5-(pentafluoro-λ^6-sulfaneyl)-3*H*-azepin-2-amine (3af)**

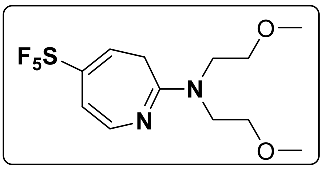

According to **General Procedure B**, pentafluoro(4-nitrophenyl)-λ^6-sulfane **1a** (24.9 mg, 0.1 mmol, 1.0 equiv) and phosphetane oxide **P(V)=O-catalyst** (2.61 mg, 0.015 mmol, 15 mol%) were dissolved in toluene. Then, amine **2f** (29.3 µL, 0.2 mmol, 2.0 equiv) and phenylsilane (24.6 µL, 0.2 mmol, 2.0 equiv) were added sequentially. The reaction mixture was stirred at 120 °C for 1 hour. **3af** was obtained as a yellow oil (26 mg, 74%) using *n*-Hexane/DCM (3/2) and *n*-Hexane/acetone (7/1). **^{1}H-NMR** (500 MHz, $CDCl_3$) δ 7.19 (d, J = 8.3 Hz, 1H), 6.03 (dd, J = 8.4, 1.6 Hz, 1H), 5.66 (td, J = 7.9, 1.6 Hz, 1H), 3.60 (s, 4H), 3.51 (d, J = 24.7 Hz, 4H), 3.38 – 3.20 (m, 6H). **^{13}C-NMR** (126 MHz, $CDCl_3$) δ 155.3 (quint J = 14.0 Hz), 146.8, 142.4, 114.2, 104.1, 70.8 (d, J = 38.3 Hz), 60.4 – 57.7 (m), 50.2, 30.2. **^{19}F-NMR** (282 MHz, $CDCl_3$) δ 85.29 (quint, 1F), 61.48 (d, J = 148.2 Hz, 4F). **HRMS** (ESI) calculated for $C_{12}H_{20}F_5N_2O_2S$ [M+H]$^+$: 351,1166, found 351.1167. **IR (KBr)**: v = 2987, 2894, 1600, 1564, 1510, 1459, 1362, 1271, 1187, 1066, 883, 813 cm^{-1}. The CH_2 in the azepine core was not observed in the ^{1}H NMR spectrum but was confirmed by ^{13}C NMR and HRMS analysis.

***N,N*-dibenzyl-5-(pentafluoro-λ^6-sulfaneyl)-3*H*-azepin-2-amine (3ag)**

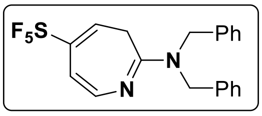

According to **General Procedure B**, pentafluoro(4-nitrophenyl)-λ^6-sulfane **1a** (49.8 mg, 0.2 mmol, 1.0 equiv) and phosphetane oxide **P(V)=O-catalyst** (5.2 mg, 0.03 mmol, 15 mol%) were dissolved in toluene. Then, amine **2g** (76.6 µL, 0.4 mmol, 2.0 equiv) and phenylsilane (49.2 µL, 0.4 mmol, 2.0 equiv) were added sequentially. The reaction mixture was stirred at 120 °C for 1 hour. **3ag** was obtained as a brown solid (32.6 mg, 39%) using *n*-Hexane/EtOAc (95/5). **m.p.**: 58.0 – 59.5 °C. **^{1}H-NMR** (500 MHz, $CDCl_3$) δ 7.38 – 7.31 (m, 3H), 7.29 (d, J = 8.1 Hz, 4H), 7.14 (d, J = 6.7 Hz, 4H), 6.13 (dd, J = 8.4, 1.5 Hz, 1H), 5.43 (td, J = 7.9, 1.5 Hz, 1H), 4.61 (s, 4H). **^{13}C-NMR** (126 MHz, $CDCl_3$, rotameric mixtures) δ 155.6 (quint, J = 14.1 Hz), 146.7, 142.7, 136.9, 129.1, 127.8 (major rotamers), 126.6 (minor rotamers), 113.1 (quint, J = 4.8 Hz), 104.9 (quint, J = 3.7 Hz), 51.9 (d, J = 79.3 Hz), 30.3. **^{19}F-NMR** (376 MHz, $CDCl_3$) δ 84.91 (quint, 1F), 61.61 (d, J = 148.5 Hz, 4F). **HRMS** (ESI) calculated for $C_{20}H_{20}F_5N_2S$ [M+H]$^+$: 415.1267, found 415.1272. **IR (KBr)**: v = 3085, 3027, 2955, 1945, 1602, 1507, 1352, 1191, 1154, 1082, 975, 902 cm^{-1}. The CH_2 in the azepine core was not observed in the ^{1}H NMR spectrum but was confirmed by ^{13}C NMR and HRMS analysis.

***N,N*-dicyclohexyl-5-(pentafluoro-λ^6-sulfaneyl)-3*H*-azepin-2-amine (3ah)**

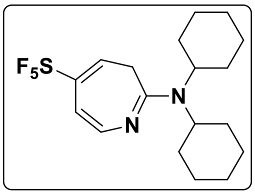

According to **General Procedure B**, pentafluoro(4-nitrophenyl)-λ^6-sulfane **1a** (49.8 mg, 0.2 mmol, 1.0 equiv) and phosphetane oxide **P(V)=O-catalyst** (5.2 mg, 0.03 mmol, 15 mol%) were dissolved in toluene. Then, amine **2h** (79.7.7 µL, 0.4 mmol, 2.0 equiv) and phenylsilane (49.2 µL, 0.4 mmol, 2.0 equiv) were added sequentially. The reaction mixture was stirred at 120 °C for 1 hour. **3ah** was obtained as a brown solid (23.75 mg, 30%) using *n*-Hexane/DCM (70/30). **m.p.**: 125.5 – 126.4 °C. **^{1}H-NMR** (500 MHz, $CDCl_3$, rotameric mixture) δ 7.19 (d, *J* = 8.2 Hz, 1H), 6.01 (dd, *J* = 8.2, 1.6 Hz, 1H), 5.45 (td, *J* = 8.1, 1.6 Hz, 1H), 3.90 (d, *J* = 360.0 Hz, 2H, rotamers), 1.74 (dd, *J* = 77.0, 13.7 Hz, 13H), 1.32 (d, *J* = 13.6 Hz, 5H), 1.14 (ddt, *J* = 16.8, 13.3, 6.7 Hz, 2H). **^{13}C-NMR** (126 MHz, $CDCl_3$) δ 155.9 (quint, *J* = 13.6 Hz), 143.7, 141.8, 112.4 (quint), 102.9 (quint, *J* = 3.6 Hz), 57.8, 33.1, 31.6, 26.4, 25.6. **^{19}F-NMR** (376 MHz, $CDCl_3$) δ 85.54 (quint, 1F), 61.89 (d, *J* = 148.6 Hz, 4F). **HRMS** (ESI) calculated for $C_{18}H_{28}F_5N_2S$ $[M+H]^+$:399.1893, found 399.1898. **IR (KBr)**: *v* = 3033, 2932, 2857, 1600, 1551, 1448, 1383, 1242, 1184, 1131, 1039, 952 cm^{-1}. The CH_2 in the azepine core was not observed in the ^{1}H NMR spectrum but was confirmed by ^{13}C NMR and HRMS analysis.

***N*-cyclohexyl-*N*-ethyl-5-(pentafluoro-λ^6-sulfaneyl)-3*H*-azepin-2-amine (3ai)**

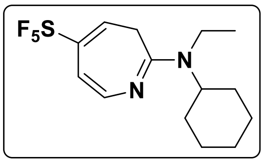

According to **General Procedure B**, pentafluoro(4-nitrophenyl)-λ^6-sulfane **1a** (49.8 mg, 0.2 mmol, 1.0 equiv) and phosphetane oxide **P(V)=O-catalyst** (5.2 mg, 0.03 mmol, 15 mol%) were dissolved in toluene. Then, amine **2i** (59.9 µL, 0.4 mmol, 2.0 equiv) and phenylsilane (49.2 µL, 0.4 mmol, 2.0 equiv) were added sequentially. The reaction mixture was stirred at 120 °C for 1 hour. **3ai** was obtained as a yellow oil (30.5 mg, 44%) using *n*-Hexane/DCM (3/1 to 1/1) and *n*-Hexane EtOAc (20/1). **^{1}H-NMR** (500 MHz, $CDCl_3$) δ 7.24 (d, *J* = 8.2 Hz, 1H), 6.02 (dd, *J* = 8.2, 1.6 Hz, 1H), 5.63 (s, 1H), 4.37 (s, 1H), 3.26 (s, 2H), 1.86 – 1.56 (m, 5H), 1.42 – 1.02 (m, 8H). **^{13}C-NMR** (126 MHz, $CDCl_3$, rotameric mixture) δ 156.1 (p, *J* = 13.7 Hz), 146.1, 142.8, 111.4, 103.6, 58.1 (minor rotamers), 56.6 (major rotamers), 38.3, 30.7, 30.5, 25.9, 25.7, 16.9 (major rotamers), 14.6 (minor rotamers). **^{19}F-NMR** ^{19} (659 MHz, CDCl3) δ 85.32 (quint, *J* = 148.2 Hz, 1F), 61.67 (d, *J* = 148.6 Hz, 4F). **HRMS** (ESI) calculated for $C_{14}H_{22}F_5N_2S$ $[M+H]^+$: 345.1424, found 345.1432. **IR (KBr)**: *v* = 2931, 2857, 1643, 1584, 1433, 1375, 1213, 1148, 1098, 1011, 964, 850 cm^{-1}. The CH_2 in the azepine core was not observed in the ^{1}H NMR spectrum but was confirmed by ^{13}C NMR and HRMS analysis.

***N*-(2-methoxyethyl)-5-(pentafluoro-λ^6-sulfaneyl)-*N*-propyl-3*H*-azepin-2-amine (3aj)**

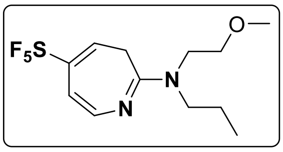

According to **General Procedure B**, pentafluoro(4-nitrophenyl)-λ^6-sulfane **1a** (49.8 mg, 0.2 mmol, 1.0 equiv) and phosphetane oxide **P(V)=O-catalyst** (5.2 mg, 0.03 mmol, 15 mol%) were dissolved in toluene. Then, amine **2j** (56.5 µL, 0.4 mmol, 2.0 equiv) and phenylsilane (49.2 µL, 0.4 mmol, 2.0 equiv) were added sequentially. The reaction mixture was stirred at 120 °C for 1 hour. **3aj** was obtained as a yellow oil (48.25 mg, 72%) using *n*-Hexane/DCM/EtOAc (60/38/2 to 60/36/4). **^{1}H-NMR** (500 MHz, $CDCl_3$, rotameric mixture) δ 7.21 (d, *J* = 8.3 Hz, 1H), 6.03 (dd, *J* = 8.4, 1.6 Hz, 1H), 5.65 (t, *J* = 8.0 Hz, 1H), 3.63 – 3.19 (m, 9H), 1.58 (d, *J* = 82.6 Hz, 2H, rotamers), 1.04 – 0.71 (m, 3H, rotamers). **^{13}C-NMR** (126 MHz, $CDCl_3$, rotameric mixture) δ 155.8, 146.6 (minor rotamers), 146.2 (major rotamers), 113.5 (minor rotamers), 112.4 (major rotamers), 104.0 – 103.9 (m), 70.60, 59.1, 52.3 (major rotamers), 51.3 (minor rotamers), 49.9 (major rotamers), 48.9 (minor rotamers), 30.2, 22.6 (major rotamers), 20.4 (minor rotamers, 11.3). **^{19}F-NMR** (376 MHz, $CDCl_3$) δ 85.14 (quint, *J* = 149.4 Hz, 1F), 61.56 (d, *J* = 148.4 Hz, 4F). **HRMS** (ESI) calculated for $C_{12}H_{20}F_5N_2OS$ $[M+H]^+$: 335.1217, found 335.1225. **IR (KBr)**: *v* = 2968, 2879, 1600, 1565, 1429, 1367, 1242, 1187, 1082, 1007, 882, 813 cm^{-1}. The CH_2 in the azepine core was not observed in the ^{1}H NMR spectrum but was confirmed by ^{13}C NMR and HRMS analysis.

2-(azetidin-1-yl)-5-(pentafluoro-λ^6-sulfaneyl)-3*H*-azepine (3ak)

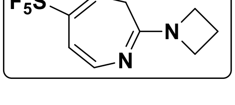

According to **General Procedure B**, pentafluoro(4-nitrophenyl)-λ^6-sulfane **1a** (99.67 mg, 0.4 mmol, 1.0 equiv) and phosphetane oxide **P(V)=O-catalyst** (10.5 mg, 0.06 mmol, 15 mol%) were dissolved in toluene. Then, amine **2k** (53.9 µL, 0.8 mmol, 2.0 equiv) and phenylsilane (98.4 µL, 0.8 mmol, 2.0 equiv) were added sequentially. The reaction mixture was stirred at 120 °C for 1 hour. **3ak** was obtained as a brown solid

(54.85 mg, 50%) using *n*-Hexane/DCM/EtOAc (60/38/2). **m.p.**: 90.9 – 91.9 °C. **^{1}H-NMR** (500 MHz, $CDCl_3$) δ 7.22 (d, *J* = 8.5 Hz, 1H), 6.05 (dd, *J* = 8.5, 1.6 Hz, 1H), 5.74 (td, *J* = 7.7, 1.6 Hz, 1H), 4.09 (s, 4H), 2.48 (s, 2H), 2.32 (tt, *J* = 8.2, 7.2 Hz, 2H). **^{13}C-NMR** (126 MHz, $CDCl_3$) δ 155.8 (quint, *J* = 14.1 Hz), 147.9, 143.6, 112.5 (quint, *J* = 4.6 Hz), 105.2 (quint, *J* = 3.9 Hz), 50.2 (d, *J* = 12.8 Hz), 28.0, 15.5. **^{19}F-NMR** (282 MHz, $CDCl_3$) δ 85.06 (quint, 1F), 61.42 (d, *J* = 148.3 Hz, 4F). **HRMS** (ESI) calculated for $C_9H_{12}F_5N_2S$ $[M+H]^+$: 275.0641, found 275.0651. **IR (KBr)**: *v* = 3011, 2958, 2880, 1912, 1581, 1470, 1442, 1240, 1203, 1170, 1119, 1074, 970 cm^{-1}.

5-(pentafluoro-λ^6-sulfaneyl)-2-(pyrrolidin-1-yl)-3*H*-azepine (3al)

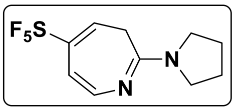

According to **General Procedure B**, pentafluoro(4-nitrophenyl)-λ^6-sulfane **1a** (49.8 mg, 0.2 mmol, 1.0 equiv) and phosphetane oxide **P(V)=O-catalyst** (5.2 mg, 0.03 mmol, 15 mol%) were dissolved in toluene. Then, amine **2l** (33.08 μL, 0.4 mmol, 2.0 equiv) and phenylsilane (49.2 μL, 0.4 mmol, 2.0 equiv) were added sequentially. The reaction mixture was stirred at 120 °C for 1 hour. **3al** was obtained as a brown solid (21.95 mg, 38%) using *n*-Hexane/EtOAc (80/20) and *n*-Hexane/acetone (80/20). **m.p.**: 84.0 – 85.0 °C. **^{1}H-NMR** (500 MHz, $CDCl_3$) δ 7.26 (d, *J* = 8.5 Hz, 1H), 6.04 (dd, *J* = 8.4, 1.6 Hz, 1H), 5.75 (td, *J* = 7.8, 1.6 Hz, 1H), 3.49 (q, *J* = 6.8 Hz, 4H), 2.66 (s, 2H), 1.99 (quint, *J* = 6.9 Hz, 2H), 1.89 (quint, *J* = 6.8 Hz, 2H). **^{13}C-NMR** (126 MHz, $CDCl_3$) δ 156.0 (quint, *J* = 13.9 Hz), 145.9, 143.5, 111.1 (quint, *J* = 4.9 Hz), 104.3 (quint *J* = 3.7 Hz), 48.2, 47.2, 31.4, 25.6, 24.7. **^{19}F-NMR** (376 MHz, $CDCl_3$) δ 85.25 (quint, 1F), 61.54 (d, *J* = 148.5 Hz, 4F). **HRMS** (ESI) calculated for $C_{10}H_{14}F_5N_2S$ $[M+H]^+$: 289.0798, found 289.0803. **IR (KBr)**: *v* = 3059, 2954, 2875, 1910, 1607, 1569, 1457, 1427, 1207, 1172, 1074, 955 cm^{-1}.

5-(pentafluoro-λ^6-sulfaneyl)-2-(piperidin-1-yl)-3*H*-azepine (3am)

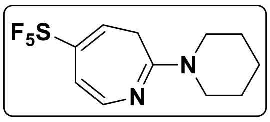

According to **General Procedure B**, pentafluoro(4-nitrophenyl)-λ^6-sulfane **1a** (49.8 mg, 0.2 mmol, 1.0 equiv) and phosphetane oxide **P(V)=O-catalyst** (5.2 mg, 0.03 mmol, 15 mol%) were dissolved in toluene. Then, amine **2m** (39.6 μL, 0.4 mmol, 2.0 equiv) and phenylsilane (49.2 μL, 0.4 mmol, 2.0 equiv) were added sequentially. The reaction mixture was stirred at 120 °C for 1 hour. **3am** was obtained as a yellow oil (31.2 mg, 52%) using *n*-Hexane/DCM (50/50 to 30/80) and *n*-Hexane/acetone (95/5 to 90/10). **^{1}H-NMR** (500 MHz, $CDCl_3$) δ 7.24 (d, *J* = 8.3 Hz, 1H), 6.05 (dd, *J* = 8.4, 1.6 Hz, 1H), 5.65 (td, *J* = 8.1, 1.6 Hz, 1H), 3.54 (t, *J* = 5.5 Hz, 4H), 1.67 – 1.61 (m, 2H), 1.58 (q, *J* = 5.8 Hz, 4H). **^{13}C-NMR** (^{13}C NMR (126 MHz, $CDCl_3$) δ 156.0 (quint, *J* = 14.1 Hz), 146.0, 143.0, 112.9 (dt, *J* = 8.7, 3.8 Hz), 104.3 (dt, *J* = 6.4, 2.4 Hz), 47.3, 29.5, 25.5, 24.6. **^{19}F-NMR** (282 MHz, $CDCl_3$) δ 85.06 (quint, 1F), 61.58 (d, *J* = 148.4 Hz, 4F). **HRMS** (ESI) calculated for $C_{11}H_{16}F_5N_2S$ $[M+H]^+$: 303.0954, found 303.0948. **IR (KBr)**: *v* = 2946, 2860, 1636, 1563, 1452, 1428, 1356, 1230, 1182, 1082, 1006, 949 cm^{-1}. The CH_2 in the azepine core was not observed in the ^{1}H NMR spectrum but was confirmed by ^{13}C NMR and HRMS analysis.

2-(azepan-1-yl)-5-(pentafluoro-λ^6-sulfaneyl)-3*H*-azepine (3an)

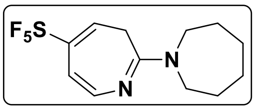

According to **General Procedure B**, pentafluoro(4-nitrophenyl)-λ^6-sulfane **1a** (49.8 mg, 0.2 mmol, 1.0 equiv) and phosphetane oxide **P(V)=O-catalyst** (5.2 mg, 0.03 mmol, 15 mol%) were dissolved in toluene. Then, amine **2n** (39.7 mg, 45.1 μL 0.4 mmol, 2.0 equiv) and phenylsilane (49.2 μL, 0.4 mmol, 2.0 equiv) were added sequentially. The reaction mixture was stirred at 120 °C for 1 hour. **3an** was obtained as a yellow oil (37.6 mg, 59%) using *n*-Hexane/EtOAc (7/1) and *n*-Hexane/DCM (3/1 to 1/1). **^{1}H-NMR** (500 MHz, $CDCl_3$) δ 7.24 (d, *J* = 8.3 Hz, 1H), 6.02 (dd, *J* = 8.3, 1.6 Hz, 1H), 5.66 (td, *J* = 8.0, 1.5 Hz, 1H), 3.55 (dt, *J* = 40.8, 6.0 Hz, 4H), 1.82 – 1.63 (m, 4H), 1.60 – 1.48 (m, 4H). **^{13}C-NMR** (126 MHz, $CDCl_3$) δ 156.0 (quint, *J* = 13.8 Hz), 146.6, 143.2, 112.1 (quint, *J* = 4.8 Hz), 103.8 (quint, *J* = 3.8 Hz), 49.6, 49.0, 30.0, 28.5, 27.3, 27.0, 26.4. **^{19}F-NMR** ^{19}F NMR (659 MHz, $CDCl_3$) δ 85.24 (quint, *J* = 148.2 Hz, 1F), 61.58 (d, *J* = 149.6 Hz, 4F). **HRMS** (ESI) calculated for $C_{12}H_{18}F_5N_2S$ $[M+H]^+$: 317.1111, found 317.1120. **IR (KBr)**: *v* = 2933, 2856, 1599, 1564, 1463, 1372, 1245, 1190, 1120, 969, 903, 881 cm^{-1}. The CH_2 in the azepine core was not observed in the ^{1}H NMR spectrum but was confirmed by ^{13}C NMR and HRMS analysis.

4-(5-(pentafluoro-λ^6-sulfaneyl)-3*H*-azepin-2-yl)morpholine (3ao)

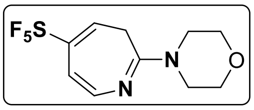

According to **General Procedure B**, pentafluoro(4-nitrophenyl)-λ⁶-sulfane **1a** (49.8 mg, 0.2 mmol, 1.0 equiv) and phosphetane oxide **P(V)=O-catalyst** (5.2 mg, 0.03 mmol, 15 mol%) were dissolved in toluene. Then, amine **2o** (34.6 µL, 0.4 mmol, 2.0 equiv) and phenylsilane (49.2 µL, 0.4 mmol, 2.0 equiv) were added sequentially. The reaction mixture was stirred at 120 °C for 1 hour. **3ao** was obtained as a yellow oil (46.3 mg, 76%) using *n*-Hexane/EtOAc (80/20). **^{1}H-NMR** (500 MHz, $CDCl_3$) δ 7.24 (d, J = 8.4 Hz, 1H), 6.12 (dd, J = 8.4, 1.5 Hz, 1H), 5.68 (td, J = 8.0, 1.5 Hz, 1H), 3.72 – 3.68 (m, 4H), 3.54 (t, J = 4.8 Hz, 4H). **^{13}C-NMR** (126 MHz, $CDCl_3$) δ 155.8 (quint, *J* = 14.1 Hz), 146.4, 142.8, 113.3, 105.6, 66.4, 46.3, 29.0. **^{19}F-NMR** (376 MHz, $CDCl_3$) δ 84.78 (quint, 1F), 61.73 (d, *J* = 148.4 Hz, 4F). **HRMS** (ESI) calculated for $C_{10}H_{14}F_5N_2OS$ $[M+H]^+$: 305.0747, found 305.0744. **IR (KBr)**: ν = 3075, 2983, 2902, 1963, 1603, 1570, 1366, 1294, 1211, 1183, 1030, 980, 884 cm^{-1}. The CH_2 in the azepine core was not observed in the ^{1}H NMR spectrum but was confirmed by ^{13}C NMR and HRMS analysis.

4-(5-(pentafluoro-λ⁶-sulfaneyl)-3*H*-azepin-2-yl)thiomorpholine (3ap)

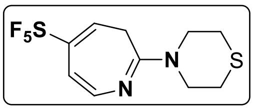

According to **General Procedure B**, pentafluoro(4-nitrophenyl)-λ⁶-sulfane **1a** (49.8 mg, 0.2 mmol, 1.0 equiv) and phosphetane oxide **P(V)=O-catalyst** (5.2 mg, 0.03 mmol, 15 mol%) were dissolved in toluene. Then, amine **2p** (37.9 µL, 0.4 mmol, 2.0 equiv) and phenylsilane (49.2 µL, 0.4 mmol, 2.0 equiv) were added sequentially. The reaction mixture was stirred at 120 °C for 1 hour. **3ap** was obtained as a white solid (30.25 mg, 47%) using *n*-Hexane/acetone (80/20) and *n*-Pentane wash. **m.p.**: 84.6 – 85.7 °C. **^{1}H-NMR** (500 MHz, $CDCl_3$) δ 7.23 (d, *J* = 8.4 Hz, 1H), 6.10 (dd, *J* = 8.4, 1.6 Hz, 1H), 5.66 (td, *J* = 8.0, 1.5 Hz, 1H), 3.89 (dd, *J* = 6.6, 3.8 Hz, 4H), 2.65 (dd, *J* = 6.4, 3.9 Hz, 4H). **^{13}C-NMR** (126 MHz, $CDCl_3$) δ 155.9 (quint, *J* = 14.2 Hz), 145.3, 142.7, 113.5 (quint, *J* = 5.0 Hz), 105.2 (quint, *J* = 3.7 Hz), 49.0, 29.3, 26.7. **^{19}F-NMR** (376 MHz, $CDCl_3$) δ 84.76 (quint), 61.70 (d, *J* = 148.3 Hz). **HRMS** (ESI) calculated for $C_{10}H_{14}F_5N_2S_2$ $[M+H]^+$: 321.0519, found 321.0522. **IR (KBr)**: ν = 2914, 1600, 1564, 1424, 1364, 1249, 1184, 1114, 1009, 955, 903, 834 cm^{-1}. The CH_2 in the azepine core was not observed in the ^{1}H NMR spectrum but was confirmed by ^{13}C NMR and HRMS analysis.

2-(4,4-difluoropiperidin-1-yl)-5-(pentafluoro-λ⁶-sulfaneyl)-3*H*-azepine (3aq)

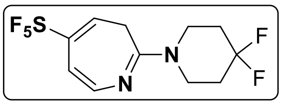

According to **General Procedure B**, pentafluoro(4-nitrophenyl)-λ⁶-sulfane **1a** (49.8 mg, 0.2 mmol, 1.0 equiv) and phosphetane oxide **P(V)=O-catalyst** (5.2 mg, 0.03 mmol, 15 mol%) were dissolved in toluene. Then, amine **2q** (42.0 µL, 0.4 mmol, 2.0 equiv) and phenylsilane (49.2 µL, 0.4 mmol, 2.0 equiv) were added sequentially. The reaction mixture was stirred at 120 °C for 1 hour. **3aq** was obtained as a white solid (42.4 mg, 63%) using *n*-Hexane/DCM/acetone (80/10/10). **m.p.**: 55.1 – 56.1 °C. **^{1}H-NMR** (700 MHz, $CDCl_3$) δ 7.24 (d, *J* = 8.4 Hz, 1H), 6.15 (dd, *J* = 8.4, 1.5 Hz, 1H), 5.70 (t, *J* = 8.0 Hz, 1H), 3.68 (t, *J* = 5.9 Hz, 4H), 2.00 (tt, *J* = 13.3, 5.9 Hz, 4H). **^{13}C-NMR** (176 MHz, $CDCl_3$) δ 155.9 (quint, *J* = 13.9 Hz), 145.3, 142.8, 121.5 (t, *J* = 241.0 Hz), 113.6, 105.8, 43.0, 33.7 (t, *J* = 23.0 Hz), 29.3. **^{19}F-NMR** (376 MHz, $CDCl_3$) δ 84.53 (quint, 1F), 61.68 (d, *J* = 148.6 Hz, 4F), -98.04 (d, 2F). **HRMS** (ESI) calculated for $C_{11}H_{14}F_7N_2S$ $[M+H]^+$: 339.0766, found 339.0772. **IR (KBr)**: ν = 3015, 2980, 2946, 1675, 1653, 1567, 1462, 1429, 1356, 1231, 1168, 1063, 940 cm^{-1}. The CH_2 in the azepine core was not observed in the ^{1}H NMR spectrum but was confirmed by ^{13}C NMR and HRMS analysis.

5-(pentafluoro-λ⁶-sulfaneyl)-2-(4-(4-(trifluoromethoxy)phenoxy)piperidin-1-yl)-3*H*-azepine (3ar)

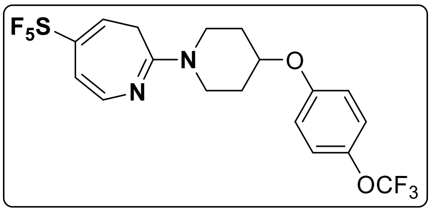

According to **General Procedure B**, pentafluoro(4-nitrophenyl)-λ⁶-sulfane **1a** (49.8 mg, 0.2 mmol, 1.0 equiv) and phosphetane oxide **P(V)=O-catalyst** (5.2 mg, 0.03 mmol, 15 mol%) were dissolved in toluene. Then, amine **2r** (104.5 mg, 0.4 mmol, 2.0 equiv) and phenylsilane (49.2 µL, 0.4 mmol, 2.0 equiv) were added sequentially. The reaction mixture was stirred at 120 °C for 1 hour. **3ar** was obtained as a yellow oil (72.05 mg, 75%) using *n*-Hexane/acetone (90/10). **^{1}H-NMR** (500 MHz, $CDCl_3$) δ 7.26 (s, 1H), 7.15 (dd, *J* = 9.1, 1.0 Hz, 2H), 6.92 – 6.87 (m, 2H), 6.10 (dd, *J* = 8.3, 1.6 Hz, 1H), 5.69 (td, *J* = 8.0, 1.6 Hz, 1H), 4.52 (tt, *J* = 6.5, 3.5 Hz, 1H), 3.76 (ddd, *J* = 12.7, 8.6, 3.6 Hz, 2H), 3.61 (dt, *J* = 8.5, 4.7 Hz, 2H), 1.95 (ddt, *J* = 12.6, 8.0, 3.7 Hz, 2H), 1.84 (dtd, *J* = 13.4, 6.5, 3.5 Hz, 2H). **^{13}C-NMR** (126 MHz, $CDCl_3$)

δ 155.9 (quint, J = 14.1 Hz), 155.6, 122.8, 120.7 (q, J = 256.3 Hz), 117.0, 113.1 (q, J = 4.8 Hz), 105.0 (q, J = 3.7 Hz), 72.1, 42.67, 30.0, 29.4. **^{19}F-NMR** (376 MHz, $CDCl_3$) δ 86.26 – 83.03 (m, 1F), 61.67 (d, J = 148.5 Hz, 4F), -58.86 (s, 3F). **HRMS** (ESI) calculated for $C_{18}H_{19}F_8N_2O_2S$ $[M+H]^+$: 479.1039, found 479.1046. **IR (KBr)**: v = 2956, 2866, 1600, 1567, 1464, 1432, 1368, 1324, 1241, 1197, 1117, 1027, 977, 918, 883 cm^{-1}. The CH_2 in the azepine core was not observed in the ^{1}H NMR spectrum but was confirmed by ^{13}C NMR and HRMS analysis.

ethyl 1-(5-(pentafluoro-λ^6-sulfaneyl)-3*H*-azepin-2-yl)piperidine-4-carboxylate (3as)

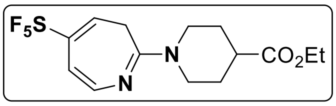

According to **General Procedure B**, pentafluoro(4-nitrophenyl)-λ^6-sulfane **1a** (49.8 mg, 0.2 mmol, 1.0 equiv) and phosphetane oxide **P(V)=O-catalyst** (5.2 mg, 0.03 mmol, 15 mol%) were dissolved in toluene. Then, amine **2s** (61.6 µL, 0.4 mmol, 2.0 equiv) and phenylsilane (49.2 µL, 0.4 mmol, 2.0 equiv) were added sequentially. The reaction mixture was stirred at 120 °C for 1 hour. **3as** was obtained as a yellow oil (55.65 mg, 74%) using DCM (100%) to DCM (100/6 drops EtOAc). **^{1}H-NMR** (500 MHz, $CDCl_3$) δ 7.24 (d, J = 8.4 Hz, 1H), 6.09 (dd, J = 8.4, 1.5 Hz, 1H), 5.67 (td, J = 8.0, 1.6 Hz, 1H), 4.15 (q, J = 7.1 Hz, 4H), 3.08 – 2.99 (m, 2H), 2.53 (tt, J = 10.6, 4.1 Hz, 1H), 1.99 – 1.90 (m, 2H), 1.72 (dtd, J = 14.3, 10.8, 3.9 Hz, 2H), 1.26 (t, J = 7.1 Hz, 3H). **^{13}C-NMR** (126 MHz, $CDCl_3$) δ 174.2, 155.9 (quint, J = 14.3 Hz), 145.9, 142.9, 113.2 (quint, J = 4.2 Hz), 104.9 (quint, J = 3.6 Hz), 60.8, 45.5, 41.0, 29.4, 27.6, 14.3. **^{19}F-NMR** (376 MHz, $CDCl_3$) δ 84.87 (quint, J = 148.5 Hz, 1F), 61.67 (d, J = 148.0 Hz, 4F). **HRMS** (ESI) calculated for $C_{14}H_{20}F_5N_2O_2S$ $[M+H]^+$: 375.1166, found 375.1178. **IR (KBr)**: v = 2982, 2860, 1728, 1600, 1564, 1509, 1465, 1372, 1321, 1294, 1118, 1010, 974, 883 cm^{-1}. The CH_2 in the azepine core was not observed in the ^{1}H NMR spectrum but was confirmed by ^{13}C NMR and HRMS analysis.

1-(5-(pentafluoro-λ^6-sulfaneyl)-3*H*-azepin-2-yl)piperidin-4-ol (3at)

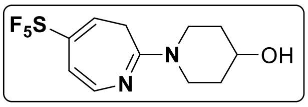

According to **General Procedure B**, pentafluoro(4-nitrophenyl)-λ^6-sulfane **1a** (99.7 mg, 0.4 mmol, 1.0 equiv) and phosphetane oxide **P(V)=O-catalyst** (10.5 mg, 0.06 mmol, 15 mol%) were dissolved in toluene. Then, amine **2t** (80.9 mg, 0.8 mmol, 2.0 equiv) and phenylsilane (98.4 µL, 0.8 mmol, 2.0 equiv) were added sequentially. The reaction mixture was stirred at 120 °C for 1 hour. **3at** was obtained as a pale yellow solid (105.4 mg, 83%) using DCM/MeOH (90/10). And *n*-Hexane/EtOAc (50/50). **m.p.**: 99.7 – 100.6 °C. **^{1}H-NMR** (700 MHz, $CDCl_3$) δ 7.23 (d, J = 8.4 Hz, 1H), 6.08 (dd, J = 8.3, 1.6 Hz, 1H), 5.68 (td, J = 8.0, 1.5 Hz, 1H), 3.98 – 3.86 (m, 3H), 3.26 (ddd, J = 13.2, 9.2, 3.4 Hz, 2H), 2.31 (s, 1H), 1.93 – 1.84 (m, 2H), 1.53 (dtd, J = 12.8, 8.6, 3.7 Hz, 2H). **^{13}C-NMR** (176 MHz, $CDCl_3$) δ 155.8 (quint, J = 13.4 Hz), 146.0, 142.85, 113.2 (t, J = 4.5 Hz), 104.8 (t, J = 4.2 Hz), 67.0, 43.4, 33.6, 29.4. **^{19}F-NMR** (658 MHz, $CDCl_3$) δ 84.93 (quint, J = 148.6 Hz, 1F), 61.67 (d, J = 148.5 Hz, 4F). **HRMS** (ESI) calculated for $C_{11}H_{16}F_5N_2OS$ $[M+H]^+$: 319.0904, found 319.0909. **IR (KBr)**: v = 3286, 2949, 2531, 1604, 1563, 1513, 1428, 1370, 1332, 1245, 1147, 1009, 980 cm^{-1}. The CH_2 in the azepine core was not observed in the ^{1}H NMR spectrum but was confirmed by ^{13}C NMR and HRMS analysis.

***N*-(1-(5-(pentafluoro-λ^6-sulfaneyl)-3*H*-azepin-2-yl)piperidin-4-yl)benzamide (3au)**

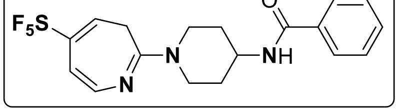

According to **General Procedure B**, pentafluoro(4-nitrophenyl)-λ^6-sulfane **1a** (74.8 mg, 0.3 mmol, 1.0 equiv) and phosphetane oxide **P(V)=O-catalyst** (7.8 mg, 0.045 mmol, 15 mol%) were dissolved in toluene. Then, amine **2u** (122.6 mg, 0.6 mmol, 2.0 equiv) and phenylsilane (73.8 µL, 0.6 mmol, 2.0 equiv) were added sequentially. The reaction mixture was stirred at 120 °C for 1 hour. **3au** was obtained as a pale yellow solid (60.68 mg, 48%) using *n*-Hexane/DCM/EtOAc (60/38/2). **m.p.**: 178.6 – 179.4 °C. **^{1}H-NMR** (700 MHz, $CDCl_3$) δ 7.75 – 7.72 (m, 2H), 7.52 – 7.49 (m, 1H), 7.45 – 7.41 (m, 2H), 7.24 (d, J = 8.4 Hz, 1H), 6.11 (dd, J = 8.4, 1.6 Hz, 1H), 6.04 (d, J = 7.8 Hz, 1H), 5.69 (td, J = 8.0, 1.6 Hz, 1H), 4.27 (s, 2H), 4.24 – 4.15 (m, 1H), 3.05 (s, 2H), 2.10 (d, J = 12.7 Hz, 2H), 1.46 (d, J = 12.7 Hz, 2H). **^{13}C-NMR** (176 MHz, $CDCl_3$) δ 167.1, 155.9 (quint, J = 13.6 Hz), 145.7, 142.9, 134.5, 131.8, 128.8, 126.0, 113.3, 105.1, 47.3, 45.1, 31.7, 29.4. **^{19}F-NMR** (658 MHz, $CDCl_3$) δ 84.85 (quint, J = 148.8 Hz, 1F), 61.66 (d, J = 148.6 Hz, 4F). **HRMS** (ESI) calculated for $C_{18}H_{21}F_5N_3OS$ $[M+H]^+$: 422.1325, found 422.1340. **IR (KBr)**: v

= 3071, 3031, 2953, 2861, 1967, 1703, 1628, 1465, 1369, 1237, 1017, 941 cm^{-1}. The CH_2 in the azepine core was not observed in the ^{1}H NMR spectrum but was confirmed by ^{13}C NMR and HRMS analysis.

2-(5-(pentafluoro-λ^6-sulfaneyl)-3*H*-azepin-2-yl)-1,2,3,4-tetrahydroisoquinoline (3av)

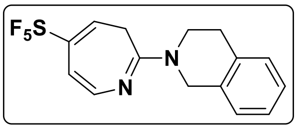

According to **General Procedure B**, pentafluoro(4-nitrophenyl)-λ^6-sulfane **1a** (49.8 mg, 0.2 mmol, 1.0 equiv) and phosphetane oxide **P(V)=O-catalyst** (5.2 mg, 0.03 mmol, 15 mol%) were dissolved in toluene. Then, amine **2v** (50.7 μL, 0.4 mmol, 2.0 equiv) and phenylsilane (49.2 μL, 0.4 mmol, 2.0 equiv) were added sequentially. The reaction mixture was stirred at 120 °C for 1 hour. **3av** was obtained as a white solid (57.1 mg, 81%) using *n*-Hexane/DCM (2/1 to 1/1). **m.p.**: 76.7 – 77.5 °C. **^{1}H-NMR** (500 MHz, $CDCl_3$) δ 7.29 (d, *J* = 8.4 Hz, 1H), 7.24 – 7.18 (m, 2H), 7.17 – 7.13 (m, 2H), 6.11 (dd, *J* = 8.4, 1.6 Hz, 1H), 5.71 (td, *J* = 8.0, 1.6 Hz, 1H), 4.72 (s, 2H), 3.80 (t, *J* = 5.8 Hz, 2H), 2.92 (t, *J* = 5.8 Hz, 2H). **^{13}C-NMR** (126 MHz, $CDCl_3$) δ 156.0 (quint, *J* = 14.3 Hz), 146.4, 143.0, 134.6, 133.1, 128.5, 127.0, 126.8, 126.6, 112.4, 105.0 (quint, *J* = 3.8 Hz), 48.0, 43.9, 29.8, 28.9. **^{19}F-NMR** (282 MHz, $CDCl_3$) δ 84.85 (quint, 1F), 61.62 (d, *J* = 148.5 Hz, 4F). **HRMS** (ESI) calculated for $C_{15}H_{16}F_5N_2S$ $[M+H]^+$: 351.0954, found 351.0960. **IR (KBr)**: *ν* = 3044, 2842, 1601, 1429, 1366, 1246, 1183, 1117, 1006, 930, 894 cm^{-1}. The CH_2 in the azepine core was not observed in the ^{1}H NMR spectrum but was confirmed by ^{13}C NMR and HRMS analysis.

***N*-benzyl-5-(pentafluoro-λ^6-sulfaneyl)-3*H*-azepin-2-amine (3aw)**

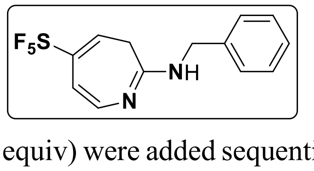

According to **General Procedure B**, pentafluoro(4-nitrophenyl)-λ^6-sulfane **1a** (49.8 mg, 0.2 mmol, 1.0 equiv) and phosphetane oxide **P(V)=O-catalyst** (5.2 mg, 0.03 mmol, 15 mol%) were dissolved in toluene. Then, amine **2w** (43.7 μL, 0.4 mmol, 2.0 equiv) and phenylsilane (49.2 μL, 0.4 mmol, 2.0 equiv) were added sequentially. The reaction mixture was stirred at 120 °C for 1 hour. **3aw** was obtained as a brown solid (31.1 mg, 48%) using *n*-Hexane/DCM/EtOAc (60/38/2). **m.p.**: 105.0 – 106.1 °C. **^{1}H-NMR** (500 MHz, $CDCl_3$) δ 7.36 – 7.28 (m, 3H), 7.27 – 7.21 (m, 3H), 6.10 (dd, *J* = 8.6, 1.6 Hz, 1H), 5.79 (t, *J* = 7.6 Hz, 1H), 4.74 (s, 1H), 4.49 (s, 2H), 2.67 (s, 2H). **^{13}C-NMR** (126 MHz, $CDCl_3$) δ 155.3 (quint, *J* = 14.3 Hz), 146.6, 143.5, 137.8, 128.9, 128.0, 127.9, 116.2, 106.1, 46.6, 34.3. **^{19}F-NMR** (282 MHz, $CDCl_3$) δ 84.66 (quint, *J* = 148.5 Hz, 1F), 61.27 (d, *J* = 148.5 Hz, 4F). **HRMS** (ESI) calculated for $C_{13}H_{14}F_5N_2S$ $[M+H]^+$: 325.0798, found 325.0793. **IR (KBr)**: *ν* = 3082, 2917, 1946, 1584, 1557, 1452, 1353, 1247, 1204, 1026, 1009, 979, 954 cm^{-1}.

***N*-(4-methoxybenzyl)-5-(pentafluoro-λ^6-sulfaneyl)-3*H*-azepin-2-amine (3ax)**

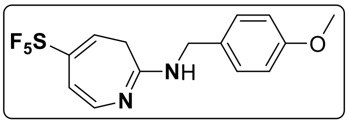

According to **General Procedure B**, pentafluoro(4-nitrophenyl)-λ^6-sulfane **1a** (49.8 mg, 0.2 mmol, 1.0 equiv) and phosphetane oxide **P(V)=O-catalyst** (5.2 mg, 0.03 mmol, 15 mol%) were dissolved in toluene. Then, amine **2x** (51.8 μL, 0.4 mmol, 2.0 equiv) and phenylsilane (49.2 μL, 0.4 mmol, 2.0 equiv) were added sequentially. The reaction mixture was stirred at 120 °C for 1 hour. **3ax** was obtained as a brown solid (39.7 mg, 56%) using *n*-Hexane/DCM/EtOAc (60/38/2). **m.p.**: 101.1 – 102.9 °C. **^{1}H-NMR** (500 MHz, $CDCl_3$) δ 7.24 (d, *J* = 8.6 Hz, 1H), 7.21 – 7.17 (m, 2H), 6.88 – 6.84 (m, 2H), 6.09 (dd, *J* = 8.6, 1.5 Hz, 1H), 5.82 – 5.74 (m, 1H), 4.65 (s, 1H), 4.43 (d, *J* = 4.4 Hz, 2H), 3.80 (s, 3H), 2.66 (s, 2H). **^{13}C-NMR** (126 MHz, $CDCl_3$) δ 159.3, 155.3 (quint, *J* = 14.3 Hz), 146.6, 143.6, 129.8, 129.5, 116.2, 114.3, 106.4 – 105.8 (m), 55.5 (q, *J* = 2.9 Hz), 46.2, 34.3. **^{19}F-NMR** (282 MHz, $CDCl_3$) δ 84.74 (quint, 1F), 61.28 (d, *J* = 148.1 Hz, 4F). **HRMS** (ESI) calculated for $C_{14}H_{16}F_5N_2OS$ $[M+H]^+$: 355.0904, found 355.0908. **IR (KBr)**: *ν* = 3073, 2993, 2960, 2941, 1611, 1582, 1447, 1422, 1247, 1194, 1009, 968, 885 cm^{-1}.

***N*-(4-fluorobenzyl)-5-(pentafluoro-λ^6-sulfaneyl)-3*H*-azepin-2-amine (3ay)**

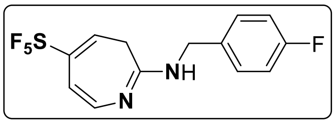

According to **General Procedure B**, pentafluoro(4-nitrophenyl)-λ^6-sulfane **1a** (49.8 mg, 0.2 mmol, 1.0 equiv) and phosphetane oxide **P(V)=O-catalyst** (5.2 mg, 0.03 mmol, 15 mol%) were dissolved in toluene. Then, amine **2y** (45.5 μL, 0.4 mmol, 2.0 equiv) and phenylsilane (49.2 μL, 0.4 mmol, 2.0 equiv) were added sequentially. The reaction mixture was stirred at 120 °C for 1 hour. **3ay** was obtained as a brown solid (45.2 mg, 66%) using *n*-Hexane/DCM/EtOAc

(60/38/2). **m.p.**: 107.0 – 108.5 °C. **^{1}H-NMR** (500 MHz, $CDCl_3$) δ 7.26 – 7.19 (m, 3H), 7.04 – 6.98 (m, 2H), 6.11 (dd, *J* = 8.6, 1.6 Hz, 1H), 5.83 – 5.75 (m, 1H), 4.69 (s, 1H), 4.47 (t, 2H), 2.68 (s, 2H). **^{13}C-NMR** (126 MHz, $CDCl_3$) δ 162.4 (d, *J* = 246.1 Hz), 155.3 (quint, *J* = 14.2 Hz), 146.3, 143.4, 133.6, 129.7 (d, *J* = 8.1 Hz), 116.2, 115.9, 115.7, 106.2, 45.9, 34.3. **^{19}F-NMR** (376 MHz, $CDCl_3$) δ 84.60 (quint, *J* = 148.4 Hz, 1F), 61.28 (d, *J* = 148.5 Hz, 4F), -115.04 (s, 1F). **HRMS** (ESI) calculated for $C_{13}H_{13}F_6N_2S$ $[M+H]^+$: 343.0704, found 343.0706. **IR (KBr)**: *ν* = 3085, 2930, 1884, 1608, 1583, 1427, 1356, 1247, 1084, 1010, 956, 890 cm^{-1}.

N-(4-bromobenzyl)-5-(pentafluoro-λ^6-sulfaneyl)-3*H*-azepin-2-amine (3az)

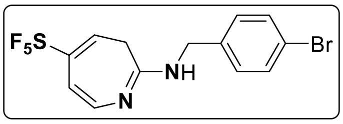

According to **General Procedure B**, pentafluoro(4-nitrophenyl)-λ^6-sulfane **1a** (49.8 mg, 0.2 mmol, 1.0 equiv) and phosphetane oxide **P(V)=O-catalyst** (5.2 mg, 0.03 mmol, 15 mol%) were dissolved in toluene. Then, amine **2z** (74.4 mg, 0.4 mmol, 2.0 equiv) and phenylsilane (49.2 µL, 0.4 mmol, 2.0 equiv) were added sequentially. The reaction mixture was stirred at 120 °C for 1 hour. **3az** was obtained as a white solid (31.7 mg, 39%) using *n*-Hexane/acetone (7/1) and *n*-Hexane/DCM (1/2). **m.p.**: 95.9 – 97.7 °C. **^{1}H-NMR** (500 MHz, $CDCl_3$) δ 7.47 – 7.43 (m, 2H), 7.22 (d, *J* = 8.5 Hz, 1H), 7.15 – 7.09 (m, 2H), 6.10 (dd, *J* = 8.6, 1.5 Hz, 1H), 5.79 (t, *J* = 7.6 Hz, 1H), 4.73 (s, 1H), 4.49 – 4.41 (m, 2H), 2.68 (s, 1H). **^{13}C-NMR** (126 MHz, $CDCl_3$) δ 155.3 (quint, *J* = 14.2 Hz), 146.3, 143.4, 137.0, 132.0, 129.6, 121.7, 116.2, 106.3 (t), 45.8, 34.3. **^{19}F-NMR** (659 MHz, $CDCl_3$) δ 84.59 (quint, *J* = 150.3 Hz, 1F), 61.31 (d, *J* = 149.2 Hz, 4F). **HRMS** (ESI) calculated for $C_{13}H_{13}BrF_5N_2S$ $[M+H]^+$: 402.9903, found 402.9912. **IR (KBr)**: *ν* = 3037, 2869, 1578, 1429, 1250, 1203, 1189, 1133, 1070, 1010, 909, 890 cm^{-1}.

N-(3,4-dichlorobenzyl)-5-(pentafluoro-λ^6-sulfaneyl)-3*H*-azepin-2-amine (3aaa)

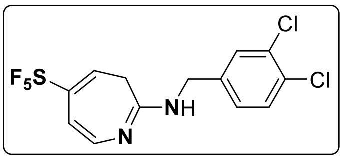

According to **General Procedure B**, pentafluoro(4-nitrophenyl)-λ^6-sulfane **1a** (49.8 mg, 0.2 mmol, 1.0 equiv) and phosphetane oxide **P(V)=O-catalyst** (5.2 mg, 0.03 mmol, 15 mol%) were dissolved in toluene. Then, amine **2aa** (52.9 µL, 0.4 mmol, 2.0 equiv) and phenylsilane (49.2 µL, 0.4 mmol, 2.0 equiv) were added sequentially. The reaction mixture was stirred at 120 °C for 1 hour. **3aaa** was obtained as a white solid (28.2 mg, 36%) using *n*-Hexane/DCM/EtOAc (80/10/10).). **m.p.**: 96.6 – 98.1 °C. **^{1}H-NMR** (500 MHz, $CDCl_3$) δ 7.38 (d, *J* = 8.2 Hz, 1H), 7.34 (d, *J* = 2.1 Hz, 1H), 7.20 (d, *J* = 8.5 Hz, 1H), 7.07 (dd, *J* = 8.2, 2.1 Hz, 1H), 6.11 (dd, *J* = 8.6, 1.6 Hz, 1H), 5.81 (t, *J* = 7.6 Hz, 1H), 4.81 (s, 1H), 4.46 (s, 2H), 2.69 (s, 2H). **^{13}C-NMR** (126 MHz, $CDCl_3$) δ 155.3 (quint, *J* = 14.5 Hz), 146.2, 143.3, 138.3, 132.9, 131.7, 130.8, 129.7, 127.1, 116.3, 106.9 – 106.1 (m), 45.2, 34.2. **^{19}F-NMR** (659 MHz, $CDCl_3$) δ 84.50 (quint, *J* = 147.9 Hz, 1F), 61.36 (d, *J* = 148.9 Hz, 4F). **HRMS** (ESI) calculated for $C_{13}H_{12}Cl_2F_5N_2S$ $[M+H]^+$: 393.0018, found 393.0030. **IR (KBr)**: *ν* = 3030, 2999, 2923, 1586, 1462, 1321, 1193, 1136, 1007, 888 cm^{-1}.

N-isopropyl-5-(pentafluoro-λ^6-sulfaneyl)-3*H*-azepin-2-amine (3aab)

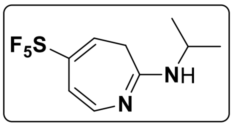

According to **General Procedure B**, pentafluoro(4-nitrophenyl)-λ^6-sulfane **1a** (49.8 mg, 0.2 mmol, 1.0 equiv) and phosphetane oxide **P(V)=O-catalyst** (5.2 mg, 0.03 mmol, 15 mol%) were dissolved in toluene. Then, amine **2ab** (34.3 µL, 0.4 mmol, 2.0 equiv) and phenylsilane (49.2 µL, 0.4 mmol, 2.0 equiv) were added sequentially. The reaction mixture was stirred at 120 °C for 1 hour. **3aab** was obtained as a brown solid (31.35 mg, 57%) using *n*-Hexane (100%) to *n*-Hexane/DCM/acetone (80/10/10). **m.p.**: 117.5 – 118.0 °C. **^{1}H-NMR** (700 MHz, $CDCl_3$) δ 7.20 (d, *J* = 8.5 Hz, 1H), 6.03 (dd, *J* = 8.5, 1.6 Hz, 1H), 5.74 (td, *J* = 7.7, 1.5 Hz, 1H), 4.28 (s, 1H), 4.06 (q, *J* = 6.9 Hz, 1H), 2.61 (s, 2H), 1.15 (d, *J* = 6.5 Hz, 6H). **^{13}C-NMR** (176 MHz, $CDCl_3$) δ 155.4 (quint, *J* = 12.8 Hz), 145.8, 143.7, 115.8, 105.4, 44.0, 34.8, 22.5. **^{19}F-NMR** (376 MHz, $CDCl_3$) δ 84.88 (quint, *J* = 148.1 Hz, 1F), 61.24 (d, *J* = 148.5 Hz, 4F). **HRMS** (ESI) calculated for $C_9H_{14}F_5N_2S$ $[M+H]^+$: 277.0798, found 277.0806. **IR (KBr)**: *ν* = 3073, 2978, 2875, 1728, 1469, 1428, 1385, 1256, 1165, 1017, 958 cm^{-1}.

N-octyl-5-(pentafluoro-λ⁶-sulfaneyl)-3H-azepin-2-amine (3aac)

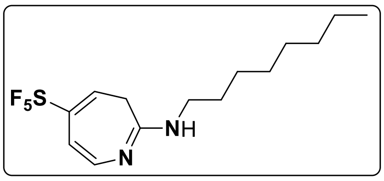

According to **General Procedure B**, pentafluoro(4-nitrophenyl)-λ⁶-sulfane **1a** (49.8 mg, 0.2 mmol, 1.0 equiv) and phosphetane oxide **P(V)=O-catalyst** (5.2 mg, 0.03 mmol, 15 mol%) were dissolved in toluene. Then, amine **2ac** (66.2 μL, 0.4 mmol, 2.0 equiv) and phenylsilane (49.2 μL, 0.4 mmol, 2.0 equiv) were added sequentially. The reaction mixture was stirred at 120 °C for 1 hour. **3aac** was obtained as a yellow solid (27.1 mg, 39%) *n*-Hexane (100%) to *n*-Hexane/DCM/acetone (60/38/2). **m.p.**: 40.0 – 41.6 °C. **^{1}H-NMR** (700 MHz, $CDCl_3$) δ 7.21 (d, *J* = 8.6 Hz, 1H), 6.04 (d, *J* = 8.5 Hz, 1H), 5.76 (td, *J* = 7.6, 1.5 Hz, 1H), 4.46 (s, 1H), 3.31 (q, *J* = 5.5 Hz, 2H), 2.64 (s, 2H), 1.51 (q, *J* = 7.2 Hz, 2H), 1.37 – 1.21 (m, 10H), 0.88 (t, *J* = 7.1 Hz, 3H). **^{13}C-NMR** (176 MHz, $CDCl_3$) δ 155.4 (quint, *J* = 12.2 Hz), 147.0, 143.7, 115.9, 105.5, 42.6, 34.5, 31.9, 29.4, 29.3, 29.2, 27.0, 22.8, 14.2. **^{19}F-NMR** (376 MHz, $CDCl_3$) δ 84.85 (quint, *J* = 148.6 Hz, 1F), 61.25 (d, *J* = 148.5 Hz, 4F). **HRMS** (ESI) calculated for $C_{14}H_{24}F_5N_2S$ $[M+H]^+$: 347.1580, found 347.1590. **IR (KBr)**: *ν* = 3078, 2956, 2926, 2854, 1471, 1430, 1379, 1257, 1199, 1142, 1010, 889 cm^{-1}.

N-methyl-5-(pentafluoro-λ⁶-sulfaneyl)-N-(3-phenyl-3-(4-(trifluoromethyl)phenoxy)propyl)-3H-azepin-2-amine (3aad)

According to **General Procedure C**, pentafluoro(4-nitrophenyl)-λ⁶-sulfane **1a** (49.8 mg, 0.2 mmol, 1.0 equiv) and phosphetane oxide **P(V)=O-catalyst** (5.2 mg, 0.03 mmol, 15 mol%) were dissolved in toluene. Then, amine **2ad** (123.7 mg, 106.7 μL, 0.4 mmol, 2.0 equiv) and phenylsilane (49.2 μL, 0.4 mmol, 2.0 equiv) were added sequentially. The reaction mixture was stirred at 120 °C for 1 hour. **3aad** was obtained as a yellow oil (72.8 mg, 69%) using *n*-Hexane/acetone (90/10). **^{1}H-NMR** (500 MHz, $CDCl_3$, rotameric mixture) δ 7.43 (d, *J* = 8.5 Hz, 2H), 7.37 – 7.20 (m, 6H), 7.17 (s, 1H), 6.87 (d, *J* = 8.3 Hz, 2H), 6.05 (dd, *J* = 8.3, 1.6 Hz, 1H), 5.34 (d, *J* = 316.5 Hz, 1H, rotamers), 3.62 (d, *J* = 54.2 Hz, 2H, rotamers), 3.00 (s, 3H), 2.16 (s, 2H). **^{13}C-NMR** (126 MHz, $CDCl_3$, rotameric mixtures) δ 160.2, 156.1, 146.7 (d, *J* = 96.2 Hz, rotamers), 143.0, 140.4, 129.1, 128.2, 127.0, 125.7, 122.3 (t, *J* = 270.9 Hz), 115.7, 112.9, 110.7, 104.5, 47.8, 37.4, 36.4, 29.8. **^{19}F-NMR** (658 MHz, $CDCl_3$) δ 85.24 (quint, *J* = 130.9 Hz, 1F), 61.82 (d, *J* = 139.9 Hz, 4F). **HRMS** (ESI) calculated for $C_{23}H_{23}F_8N_2OS$ $[M+H]^+$: 527.1403, found 527.1402. **IR (KBr)**: *ν* = 3064, 3031, 2929, 2554, 2220, 1953, 1731, 1433, 1203, 1067, 1008, 984, 882 cm^{-1}. The CH_2 in the azepine core was not observed in the ^{1}H NMR spectrum but was confirmed by ^{13}C NMR and HRMS analysis.

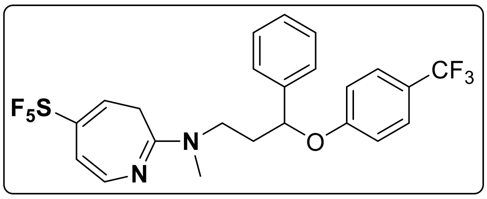

N-methyl-5-(pentafluoro-λ⁶-sulfaneyl)-N-(2-(pyridin-2-yl)ethyl)-3H-azepin-2-amine (3aae)

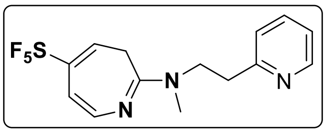

According to **General Procedure B**, pentafluoro(4-nitrophenyl)-λ⁶-sulfane **1a** (49.8 mg, 0.2 mmol, 1.0 equiv) and phosphetane oxide **P(V)=O-catalyst** (5.2 mg, 0.03 mmol, 15 mol%) were dissolved in toluene. Then, amine **2ae** (55.4 μL, 0.4 mmol, 2.0 equiv) and phenylsilane (49.2 μL, 0.4 mmol, 2.0 equiv) were added sequentially. The reaction mixture was stirred at 120 °C for 1 hour. **3aae** was obtained as a yellow oil (45.9 mg, 65%) using DCM (100%) to DCM/Et_3N (100/1) and EtOAc. **^{1}H-NMR** (500 MHz, $CDCl_3$, rotameric mixtures) δ 8.57 (s, 1H), 7.58 (s, 1H), 7.26 – 6.87 (m, 3H, rotamers), 6.03 (s, 1H), 5.45 (d, *J* = 165.0 Hz, 1H, rotamers), 3.83 (s, 2H), 3.17 – 2.73 (m, 5H, rotamers). **^{13}C-NMR** (126 MHz, $CDCl_3$, rotameric mixtures) δ 159.1 (minor rotamers, 158.2 (major rotamers), 155.8, 149.8, 147.6 (major rotamers), 146.0 (minor rotamers), 142.9, 136.8, 123.9, 122.0, 113.4 (major rotamers), 110.6 (minor rotamers), 104.5 (major rotamers), 104.0 (minor rotamers), 51.3 (minor rotamers), 50.8 (major rotamers), 37.3 (major rotamers), 36.9 (minor rotamers), 35.5, 29.8 (minor rotamerd), 29.4 (major rotamers). **^{19}F-NMR** (658 MHz, $CDCl_3$) δ 85.13 (quint, *J* = 149.8 Hz, 1F), 61.59 (d, *J* = 148.4 Hz, 4F). **HRMS** (ESI) calculated for $C_{14}H_{17}F_5N_3S$ $[M+H]^+$: 354.1063, found 354.1075. **IR (KBr)**: *ν* = 3011, 2932, 1951, 1571, 1436, 1360, 1176, 1115, 1052, 944 cm^{-1}. The CH_2 in the azepine core was not observed in the ^{1}H NMR spectrum but was confirmed by ^{13}C NMR and HRMS analysis.

(*S*)-*N*-methyl-*N*-(3-(naphthalen-1-yloxy)-3-(thiophen-2-yl)propyl)-5-(pentafluoro-λ⁶-sulfaneyl)-3*H*-azepin-2-amine (3aaf)

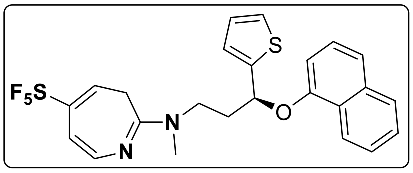

According to **General Procedure C**, pentafluoro(4-nitrophenyl)-λ^6-sulfane **1a** (49.8 mg, 0.2 mmol, 1.0 equiv) and phosphetane oxide **P(V)=O-catalyst** (5.2 mg, 0.03 mmol, 15 mol%) were dissolved in toluene. Then, amine **2af** (119.0 mg, 0.4 mmol, 2.0 equiv) and phenylsilane (49.2 μL, 0.4 mmol, 2.0 equiv) were added sequentially. The reaction mixture was stirred at 120 °C for 1 hour. **3aaf** was obtained as a yellow oil (67.9 mg, 66%) using DCM/*n*-Hexane (60/40). **^{1}H-NMR** (500 MHz, $CDCl_3$, rotameric mixtures) δ 8.36 – 8.31 (m, 1H), 7.80 – 7.74 (m, 1H), 7.53 – 7.44 (m, 2H), 7.39 (d, *J* = 8.3 Hz, 1H, rotamers), 7.24 (t, *J* = 8.0 Hz, 1H), 7.18 (s, 2H), 7.02 (s, 1H), 6.91 (t, *J* = 4.3 Hz, 1H), 6.78 (d, *J* = 7.5 Hz, 1H), 6.09 – 5.99 (m, 1H), 5.63 (s, 2H), 3.65 (s, 2H), 2.94 (s, 3H), 2.38 (d, *J* = 45.5 Hz, 2H). **^{13}C-NMR** (126 MHz, $CDCl_3$, rotameric mixtures) δ 155.9, 152.9, 146.8 (d, *J* = 108.5 Hz), 144.6, 143.0, 134.7, 127.8, 126.8, 126.6, 126.0, 125.8, 125.6, 125.1, 125.0, 121.9 (major rotamers), 121.1 (minor rotamers), 113.0, 110.7, 107.0, 104.5, 73.7, 47.7 (d, *J* = 66.5 Hz), 37.3, 36.5, 29.7 (d, *J* = 24.0 Hz). **^{19}F-NMR** (376 MHz, $CDCl_3$) δ 85.22 (quint, *J* = 148.7 Hz, 1F), 61.78 (d, *J* = 148.5 Hz, 4F). **HRMS** (ESI) calculated for $C_{24}H_{24}F_5N_2OS_2$ $[M+H]^+$: 515.1250, found 515.1249. **IR (KBr)**: *v* = 3054, 3011, 2931, 1918, 1804, 1727, 1628, 1461, 1434, 1366, 1117, 1039, 950 cm^{-1}. The CH_2 in the azepine core was not observed in the ^{1}H NMR spectrum but was confirmed by ^{13}C NMR and HRMS analysis.

***N*-((1*R*,4*S*)-4-(3,4-dichlorophenyl)-1,2,3,4-tetrahydronaphthalen-1-yl)-*N*-methyl-5-(pentafluoro-λ⁶-sulfaneyl)-3*H*-azepin-2-amine (3aag)**

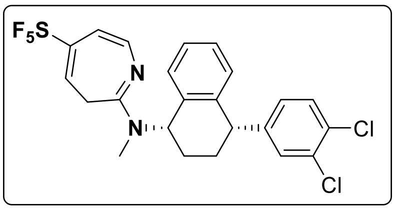

According to **General Procedure C**, pentafluoro(4-nitrophenyl)-λ^6-sulfane **1a** (49.8 mg, 0.2 mmol, 1.0 equiv) and phosphetane oxide **P(V)=O-catalyst** (5.2 mg, 0.03 mmol, 15 mol%) were dissolved in toluene. Then, amine **2ag** (122.5 mg, 0.4 mmol, 2.0 equiv) and phenylsilane (49.2 μL, 0.4 mmol, 2.0 equiv) were added sequentially. The reaction mixture was stirred at 120 °C for 1 hour. **3aag** was obtained as a yellow oil (62.8 mg, 60%) using DCM/*n*-Hexane (60/40). **^{1}H-NMR** (500 MHz, $CDCl_3$, rotameric mixtures) δ 7.34 (d, *J* = 8.3 Hz, 1H), 7.28 (d, *J* = 8.3 Hz, 1H), 7.23 – 7.14 (m, 2H), 7.09 (d, *J* = 19.3 Hz, 1H), 7.03 – 6.73 (m, 3H), 6.16 – 6.09 (m, 1H), 6.01 – 5.18 (m, 2H, rotamers), 4.29 – 4.12 (m, 1H, rotamers, 2.79 (s, 3H), 2.43 – 1.93 (m, 2H, rotamers), 1.93 – 1.52 (m, 2H). **^{13}C-NMR** (126 MHz, $CDCl_3$, rotameric mixtures) δ 156.2 (quint, *J* = 12.8 Hz), 147.8 – 146.3 (m), 143.0 (minor rotamer), 142.6 (major rotamer), 138.0, 136.3, 135.8, 132.6 (minor rotamer), 132.4 (major rotamer), 131.3 (minor rotamer), 131.0 (major rotamer), 130.7, 130.2, 128.1, 127.6, 127.0, 113.4, 110.6, 104.9 (minor rotamer), 104.4 (major rotamer), 57.8 (minor rotamer), 56.2 (major rotamer), 43.0, 32.9 (mnor rotamer), 32.0 (major rotamer), 30.6 (minor rotamer), 30.2 (major rotamer), 29.8, 22.8 (minor rotamer), 20.8 (major rotamer). **^{19}F-NMR** (282 MHz, $CDCl_3$) δ 84.95 (quint, *J* = 148.7 Hz, 1F), 61.78 (d, *J* = 148.5 Hz, 4F). **HRMS** (ESI) calculated for $C_{23}H_{22}Cl_2F_5N_2S$ $[M+H]^+$: 523.0801, found 523.0808. **IR (KBr)**: *v* = 3019, 2938, 2867, 2344, 1900, 1435, 1327, 1215, 1131, 1060, 1008, 966, 883 cm^{-1}. The CH_2 in the azepine core was not observed in the ^{1}H NMR spectrum but was confirmed by ^{13}C NMR and HRMS analysis.

***N*,*N*-diethyl-6-(pentafluoro-λ⁶-sulfaneyl)-3*H*-azepin-2-amine (3ba)**

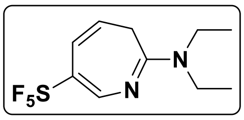

According to **General Procedure B**, pentafluoro(3-nitrophenyl)-λ^6-sulfane **1b** (124.6 mg, 0.5 mmol, 1.0 equiv) and phosphetane oxide **P(V)=O-catalyst** (13.1 mg, 0.075 mmol, 15 mol%) were dissolved in toluene. Then, amine **2a** (104.2 μL, 1.0 mmol, 2.0 equiv) and phenylsilane (123.0 μL, 1.0 mmol, 2.0 equiv) were added sequentially. The reaction mixture was stirred at 120 °C for 2 hours. **3ba** was obtained as a yellow oily (58.0 mg, 40%) using *n*-Hexane/DCM (5/1 to 1/1) then *n*-Hexane/EtOAc (7/1). **^{1}H-NMR** (500 MHz, $CDCl_3$) δ 7.84 (s, 1H), 6.73 (dd, *J* = 9.3, 1.7 Hz, 1H), 5.16 (q, *J* = 7.9 Hz, 1H), 3.39 (dt, *J* = 30.4, 7.5 Hz, 4H), 2.64 (s, 2H), 1.24 (t, *J* = 7.3 Hz, 3H), 1.08 (t, *J* = 7.0 Hz, 3H). **^{13}C-NMR** (126 MHz, $CDCl_3$) δ 146.2, 142.3, 140.3 (quint, *J* = 11.5 Hz), 125.9, 112.2, 44.2, 43.5, 32.0, 14.2, 12.5. **^{19}F-NMR** (658 MHz, $CDCl_3$) δ 90.52 (quint, *J* = 148.8 Hz, 1F), 66.28 (d,

J = 149.0 Hz, 4F). **HRMS** (ESI) calculated for calculated for $C_{10}H_{16}F_5N_2S$ $[M+H]^+$: 291.0954, found 291.0951. **IR (KBr)**: v = 2980, 2937, 1565, 1516, 1318, 1258, 1032, 894, 827, cm^{-1}

***N,N*-diethyl-6-(pentafluoro-λ⁶-sulfaneyl)-2-azabicyclo[3.2.0]hepta-2,6-dien-3-amine (4aa)**

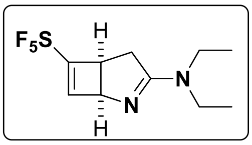

According to **General Procedure D**, 3*H*-azepin-2-amine **3aa** (43.54 mg, 0.15 mmol, 1.0 equiv) was dissolved in freshly dry degassed MeCN (4.5 mL, 0.033 M). The reaction mixture was stirred under irradiation (365 nm) for 40 hours. **4aa** was obtained as a yellow oil (21.1 mg, 48%) using *n*-Hexane/DCM/Et$_3$N (80/20/3%). **^{1}H-NMR** (500 MHz, CDCl$_3$) δ 6.57 (t, J = 1.8 Hz, 1H), 4.85 – 4.73 (m, 1H), 3.81 (d, J = 10.0 Hz, 1H), 3.28 (s, 4H), 2.74 (ddq, J = 16.8, 3.0, 1.4 Hz, 1H), 2.63 (ddd, J = 16.9, 10.1, 1.0 Hz, 1H), 1.14 (t, J = 7.1 Hz, 6H). **^{13}C-NMR** (126 MHz, CDCl$_3$) δ 167.5, 153.9 (quint), 143.5 (quint, J = 6.4 Hz), 69.5, 48.8, 42.9, 32.7, 13.67. **^{19}F-NMR** (376 MHz, CDCl$_3$) δ 79.78 (quint, 1F), 57.07 (d, J = 149.8 Hz, 4F). **HRMS** (ESI) calculated for $C_{10}H_{16}F_5N_2S$ $[M+H]^+$: 291.0954, found 291.0963. **R (KBr)**: v = 2971, 2933, 2877, 1596, 1451, 1378, 1219, 1146, 1099, 1004, 964, 850 cm^{-1}.

***N,N*-dibutyl-6-(pentafluoro-λ⁶-sulfaneyl)-2-azabicyclo[3.2.0]hepta-2,6-dien-3-amine (4ab)**

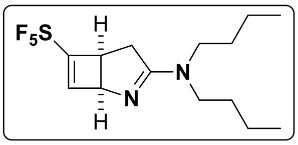

According to **General Procedure D**, 3*H*-azepin-2-amine **3ab** (27.1 mg, 0.0782 mmol, 1.0 equiv) was dissolved in freshly dry degassed MeCN (2.4 mL, 0.033 M). The reaction mixture was stirred under irradiation (365 nm) for 40 hours. **4ab** was obtained as a yellow oil (14.7 mg, 54%) using *n*-Hexane/EtOAc/Et$_3$N (90/10/2%). **^{1}H-NMR** (500 MHz, CDCl$_3$) δ 6.57 (t, J = 1.7 Hz, 1H), 4.80 (tt, J = 4.4, 2.5 Hz, 1H), 3.79 (dt, J = 10.0, 2.9 Hz, 1H), 3.20 (s, 4H), 2.74 (ddd, J = 16.8, 3.1, 1.6 Hz, 1H), 2.61 (ddd, J = 16.9, 10.0, 1.0 Hz, 1H), 1.52 (quint, J = 7.8 Hz, 4H), 1.36 – 1.20 (m, 4H), 0.93 (t, J = 7.4 Hz, 6H). **^{13}C-NMR** (126 MHz, CDCl$_3$) δ 167.9, 153.9 (quint), 143.58 (quint, J = 6.2 Hz), 69.5, 48.7, 32.9, 29.9, 20.3, 14.1. **^{19}F-NMR** (376 MHz, CDCl$_3$) δ 79.81 (quint, J = 149.4 Hz, 1F), 57.07 (d, J = 150.2 Hz, 4F). **HRMS** (ESI) calculated for $C_{14}H_{24}F_5N_2S$ $[M+H]^+$: 347.1580, found 347.1590. **IR (KBr)**: v = 2960, 2931, 2872, 1593, 1459, 1377, 1268, 1146, 1021, 928, 838 cm^{-1}.

***N,N*-didecyl-6-(pentafluoro-λ⁶-sulfaneyl)-2-azabicyclo[3.2.0]hepta-2,6-dien-3-amine (4ac)**

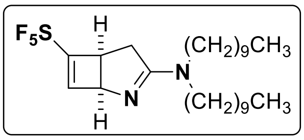

According to **General Procedure D**, 3*H*-azepin-2-amine **3ac** (51.5 mg, 0.1 mmol, 1.0 equiv) was dissolved in freshly dry degassed MeCN (3.0 mL, 0.033 M). The reaction mixture was stirred under irradiation (365 nm) for 40 hours. **4ac** was obtained as a yellow oil (24.4 mg, 47%) using *n*-Hexane/EtOAc/Et$_3$N (90/10/2%) **^{1}H-NMR** (500 MHz, CDCl$_3$) δ 6.56 (t, J = 1.7 Hz, 1H), 4.84 – 4.73 (m, 1H), 3.78 (dt, J = 10.2, 3.1 Hz, 1H), 3.18 (s, 4H), 2.74 (ddt, J = 18.3, 3.2, 1.5 Hz, 1H), 2.60 (ddd, J = 16.8, 10.1, 1.0 Hz, 1H), 1.52 (quint, J = 7.2 Hz, 4H), 1.27 (d, J = 7.7 Hz, 28zH), 0.88 (t, J = 6.9 Hz, 6H). **^{13}C-NMR** (126 MHz, CDCl$_3$) δ 167.9, 153.9 (quint, J = 15.6 Hz), 143.6 (quint, J = 6.5 Hz), 69.6, 48.8, 33.0, 32.0, 29.7, 29.7, 29.6, 29.5, 27.1, 22.8, 14.3. **^{19}F-NMR** (376 MHz, CDCl$_3$) δ 79.82 (quint, J = 149.9 Hz, 1F), 57.09 (d, J = 150.1 Hz, 4F). **HRMS** (ESI) calculated for $C_{26}H_{48}F_5N_2S$ $[M+H]^+$: 515.3458, found 515.3463. **IR (KBr)**: v = 2926, 2855, 1593, 1462, 1306, 1220, 1185, 1116, 1097, 965, 841 cm^{-1}.

***N,N*-bis(2-methoxyethyl)-6-(pentafluoro-λ⁶-sulfaneyl)-2-azabicyclo[3.2.0]hepta-2,6-dien-3-amine (4af)**

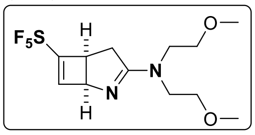

According to **General Procedure D**, 3*H*-azepin-2-amine **3af** (35.0 mg, 0.1 mmol, 1.0 equiv) was dissolved in freshly dry degassed MeCN (3.0 mL, 0.033 M). The reaction mixture was stirred under irradiation (365 nm) for 40 hours. **4af** was obtained as a brown solid (23.5 mg, 67%) using *n*-Hexane/EtOAc/Et$_3$N (80/20/3% to 80/20/5%). **m.p.**: 62.3 – 64.2 °C. **^{1}H-NMR** (500 MHz, CDCl$_3$) δ 6.55 (t, J = 1.8 Hz, 1H), 4.83 – 4.71 (m, 1H), 3.84 – 3.75 (m, 1H), 3.49 (s, 8H), 3.33 (s, 6H), 2.91 (ddq, J = 17.3, 3.6, 1.5 Hz, 1H), 2.70 (ddd, J = 17.0, 10.1, 1.1 Hz, 1H). **^{13}C-NMR** (126 MHz, CDCl$_3$) δ 168.7, 154.0 (quint, J = 15.9 Hz), 143.1 (quint, J = 6.4 Hz), 70.9, 69.5, 59.1, 48.9, 33.0. **^{19}F-NMR** (376 MHz, CDCl$_3$) δ 79.68 (quint, J = 150.2 Hz, 1F), 57.06 (d, J = 150.3 Hz, 4F). **HRMS** (ESI) calculated for $C_{12}H_{20}F_5N_2O_2S$ $[M+H]^+$: 351.1166, found 351.1178. **IR (KBr)**: v = 3041, 2995, 2931, 2898, 2850, 1588, 1467, 1416, 1228, 1072, 1007, 962, 864 cm^{-1}.

N-cyclohexyl-N-ethyl-6-(pentafluoro-λ^6-sulfaneyl)-2-azabicyclo[3.2.0]hepta-2,6-dien-3-amine (4ai)

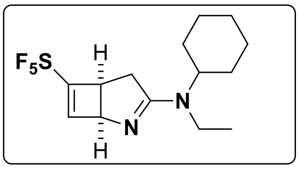

According to **General Procedure D**, 3*H*-azepin-2-amine **3ai** (73 mg, 0.211 mmol, 1.0 equiv) was dissolved in freshly dry degassed MeCN (4.5 mL, 0.047 M). The reaction mixture was stirred under irradiation (365 nm) for 40 hours. **4ai** was obtained as a yellow oil (29.1 mg, 40%) using *n*-Hexane/EtOAc/Et_3N (80/20/3%). **^{1}H-NMR** (500 MHz, $CDCl_3$) δ 6.58 (t, *J* = 1.8 Hz, 1H), 4.85 – 4.73 (m, 1H), 3.85 – 3.69 (m, 1H), 3.26 (s, 2H), 2.78 – 2.71 (m, 1H), 2.64 (ddd, *J* = 16.8, 10.0, 1.0 Hz, 1H), 1.87 – 1.61 (m, 5H), 1.51 – 1.25 (m, 5H), 1.15 (t, *J* = 7.0 Hz, 3H), 1.08 (tt, *J* = 12.9, 3.7 Hz, 1H). **^{13}C-NMR** (126 MHz, $CDCl_3$) δ 167.6, 153.9 (quint, *J* = 15.7 Hz), 144.0 – 143.1 (m), 69.3, 58.2, 48.4, 38.7, 33.0, 31.7 (d), 26.0 (d, *J* = 8.7 Hz), 25.6. **^{19}F-NMR** (658 MHz, $CDCl_3$) δ 79.87 (quint, *J* = 150.2 Hz, 1F), 57.08 (d, *J* = 149.9 Hz, 4F). **HRMS** (ESI) calculated for $C_{14}H_{22}F_5N_2S$ $[M+H]^+$: 345.1424, found 345.1432. **IR (KBr)**: ν = 2931, 2857, 1643, 1584, 1433, 1375, 1213, 1148, 1098, 1054, 964, 893 cm^{-1}.

N-(2-methoxyethyl)-6-(pentafluoro-λ^6-sulfaneyl)-N-propyl-2-azabicyclo[3.2.0]hepta-2,6-dien-3-amine (4aj)

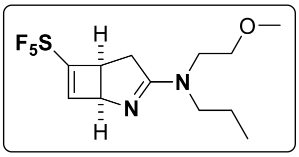

According to **General Procedure D**, 3*H*-azepin-2-amine **3aj** (33.1 mg, 0.1 mmol, 1.0 equiv) was dissolved in freshly dry degassed MeCN (3.0 mL, 0.033 M). The reaction mixture was stirred under irradiation (365 nm) for 40 hours. **4aj** was obtained as a pale yellow solid (18.1 mg, 55%) using *n*-n-Hexane/EtOAc/Et_3N (90/10/5%). **m.p.**: 58.2 – 59.9 °C. **^{1}H-NMR** (500 MHz, $CDCl_3$) δ 6.56 (t, *J* = 1.7 Hz, 1H), 4.86 – 4.76 (m, 1H), 3.79 (dt, *J* = 10.2, 3.2 Hz, 1H), 3.65 – 3.35 (m, 4H), 3.33 (s, 3H), 3.22 (dt, *J* = 15.7, 8.9 Hz, 2H), 2.81 (d, *J* = 17.0 Hz, 1H), 2.66 (ddd, *J* = 16.9, 10.2, 1.1 Hz, 1H), 1.57 (h, *J* = 7.3 Hz, 2H), 0.89 (t, *J* = 7.4 Hz, 3H). **^{13}C-NMR** (126 MHz, $CDCl_3$) δ 168.2, 153.9 (quint, *J* = 15.7 Hz), 143.9 – 142.9 (m), 70.7, 69.5, 59.1, 51.9, 48.8, 48.5, 32.9, 21.5, 11.3. **^{19}F-NMR** (658 MHz, $CDCl_3$) δ 79.74 (quint, *J* = 150.1 Hz, 1F), 57.07 (d, *J* = 150.1 Hz, 4F). **HRMS** (ESI) calculated for $C_{12}H_{20}F_5N_2OS$ $[M+H]^+$: 335.1217, found 335.1225. **IR (KBr)**: ν = 3046, 2965, 2932, 1591, 1456, 1416, 1376, 1186, 1149, 1112, 1023, 990, 862 cm^{-1}.

4-(6-(pentafluoro-λ^6-sulfaneyl)-2-azabicyclo[3.2.0]hepta-2,6-dien-3-yl)morpholine (4ao)

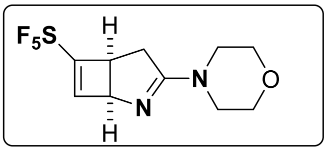

According to **General Procedure D**, 3*H*-azepin-2-amine **3ao** (45.6 mg, 0.15 mmol, 1.0 equiv) was dissolved in freshly dry degassed MeCN (4.5 mL, 0.033 M). The reaction mixture was stirred under irradiation (365 nm) for 66 hours. **4ao** was obtained as a pale yellow solid (26.43 mg, 58%) using *n*-Hexane/DCM/Et_3N (50/50/3%). **m.p.**: 76.3 – 77.9 °C. **^{1}H-NMR** (500 MHz, $CDCl_3$) δ 6.58 (t, *J* = 1.7 Hz, 1H), 4.82 (p, *J* = 3.0 Hz, 1H), 3.83 (dt, *J* = 10.2, 3.3 Hz, 1H), 3.71 (t, *J* = 4.9 Hz, 4H), 3.37 (td, *J* = 11.1, 5.8 Hz, 4H), 2.77 – 2.67 (m, 1H), 2.61 (ddd, *J* = 16.9, 10.0, 1.1 Hz, 1H). **^{13}C-NMR** (126 MHz, $CDCl_3$) δ 168.7, 154.0 (quint, *J* = 16.8 Hz), 143.1 (t), 69.4, 66.5, 48.6, 46.2, 32.6. **^{19}F-NMR** (658 MHz, $CDCl_3$) δ 79.38 (quint, *J* = 150.3 Hz, 1F), 57.11 (d, *J* = 150.2 Hz, 4F). **HRMS** (ESI) calculated for $C_{10}H_{14}F_5N_2OS$ $[M+H]^+$: 305.0747, found 305.0753. **IR (KBr)**: ν = 2962, 2926, 1596, 1440, 1309, 1149, 917, cm^{-1}.

4-(6-(pentafluoro-λ^6-sulfaneyl)-2-azabicyclo[3.2.0]hepta-2,6-dien-3-yl)thiomorpholine (4ap)

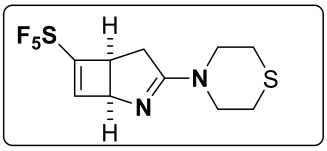

According to **General Procedure D**, 3*H*-azepin-2-amine **3ao** (39.0 mg, 0.122 mmol, 1.0 equiv) was dissolved in freshly dry degassed MeCN (3.7 mL, 0.033 M). The reaction mixture was stirred under irradiation (365 nm) for 40 hours. **4ap** was obtained as a yellow solid (23.7 mg, 61%) using *n*-Hexane/EtOAc/Et_3N (80/20/5%). **m.p.**: 69.5 – 70.9 °C. **^{1}H-NMR** (500 MHz, $CDCl_3$) δ 6.57 (t, *J* = 1.9 Hz, 1H), 4.89 – 4.73 (m, 1H), 3.83 (dd, *J* = 10.1, 3.3 Hz, 1H), 3.79 – 3.58 (m, 4H), 2.71 (dtd, *J* = 16.8, 3.1, 1.4 Hz, 1H), 2.66 – 2.55 (m, 5H). **^{13}C-NMR** (126 MHz, $CDCl_3$) δ 167.7, 154.0 (quint, *J* = 16.3 Hz), 143.5 – 142.8 (m), 69.3, 48.6, 32.9, 26.9. **^{19}F-NMR** (658 MHz, $CDCl_3$) δ 79.45 (quint, *J* = 150.2 Hz, 1F), 57.15 (d, *J* = 150.3 Hz, 4F). **HRMS** (ESI) calculated for

$C_{10}H_{14}F_5N_2S_2$ [M+H]$^+$: 321.0519, found 321.0525. **IR (KBr)**: *v* = 2923, 2854, 1592, 1434, 1377, 1226, 1179, 1102, 967, 864 cm^{-1}.

3-(4,4-difluoropiperidin-1-yl)-6-(pentafluoro-λ^6-sulfaneyl)-2-azabicyclo[3.2.0]hepta-2,6-diene (4aq)

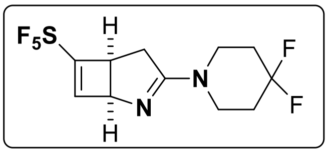

According to **General Procedure D**, 3*H*-azepin-2-amine **3aq** (50.7 mg, 0.15 mmol, 1.0 equiv) was dissolved in freshly dry degassed MeCN (4.5 mL, 0.033 M). The reaction mixture was stirred under irradiation (365 nm) for 40 hours. **4aq** was obtained as a yellow solid (27.0 mg, 53%) using *n*-Hexane/EtOAc/Et$_3$N (70/30/1%). **m.p.**: 49.5 – 50.1 °C. **^{1}H-NMR** (500 MHz, $CDCl_3$) δ 6.59 (s, 1H), 4.84 (dddq, *J* = 4.3, 3.3, 2.4, 1.1 Hz, 1H), 3.91 – 3.81 (m, 1H), 3.54 (s, 4H), 2.76 (dddd, *J* = 16.9, 4.3, 2.4, 1.2 Hz, 1H), 2.65 (ddd, *J* = 17.0, 10.0, 1.2 Hz, 1H), 2.09 – 1.87 (m, 4H). **^{13}C-NMR** (126 MHz, $CDCl_3$) δ 167.7, 154.1 (td, *J* = 16.4, 2.6 Hz), 143.1 (quint, *J* = 6.5 Hz), 121.7 (t, *J* = 242.1 Hz), 69.5, 49.0 (t, *J* = 1.8 Hz), 43.0, 33.8 (t, *J* = 23.1 Hz), 32.8, 29.4. **^{19}F-NMR** (376 MHz, $CDCl_3$) δ 79.26 (quint, *J* = 150.6 Hz, 1F), 57.14 (d, *J* = 150.1 Hz, 4F), -98.18 (t, *J* = 14.5 Hz, 2F). **HRMS** (ESI) calculated for $C_{11}H_{14}F_7N_2S$ [M+H]$^+$: 339.0766, found 339.0773. **IR (KBr)**: *v* = 2961, 2933, 1922, 1600, 1443, 1358, 1227, 1189, 1019, 979, 872 cm^{-1}.

6-(pentafluoro-λ^6-sulfaneyl)-3-(4-(4-(trifluoromethoxy)phenoxy)piperidin-1-yl)-2-azabicyclo[3.2.0]hepta-2,6-diene (4ar)

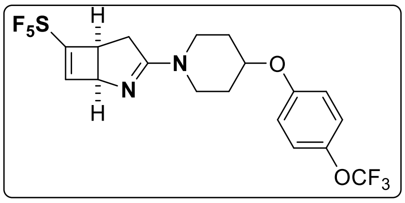

According to **General Procedure D**, 3*H*-azepin-2-amine **3ar** (71.8 mg, 0.15 mmol, 1.0 equiv) was dissolved in freshly dry degassed MeCN (4.5 mL, 0.033 M). The reaction mixture was stirred under irradiation (365 nm) for 40 hours. **4ar** was obtained as an orange solid (43.2 mg, 60%) using *n*-Hexane/acetone/Et$_3$N (90/10/10%). **m.p.**: 105.8 – 106.4 °C. **^{1}H-NMR** (500 MHz, $CDCl_3$) δ 7.14 (dq, *J* = 8.1, 1.1 Hz, 2H), 6.93 – 6.86 (m, 2H), 6.58 (s, 1H), 4.82 (q, *J* = 3.0 Hz, 1H), 4.48 (tt, *J* = 7.1, 3.4 Hz, 1H), 3.83 (dt, *J* = 10.0, 3.2 Hz, 1H), 3.66 (t, *J* = 10.9 Hz, 2H), 3.36 (dd, *J* = 15.3, 7.7 Hz, 2H), 2.79 – 2.70 (m, 1H), 2.63 (dd, *J* = 16.9, 10.1 Hz, 1H), 2.01 – 1.90 (m, 2H), 1.86 – 1.71 (m, 2H). **^{13}C-NMR** (126 MHz, $CDCl_3$) δ 168.3, 155.7, 154.1 (quint, *J* = 16.6 Hz), 143.1 (d, *J* = 18.5 Hz), 122.7, 120.7 (q, *J* = 256.3 Hz), 116.9, 72.4, 69.4, 48.8, 42.8, 32.8, 30.2. **^{19}F-NMR** (658 MHz, $CDCl_3$) δ 79.48 (quint, *J* = 150.2 Hz, 1F), 57.14 (d, *J* = 150.3 Hz, 4F), -58.85 (s, 3F). **HRMS** (ESI) calculated for $C_{18}H_{19}F_8N_2O_2S$ [M+H]$^+$: 479.1039, found 479.1046. **IR (KBr)**: *v* = 2937, 2868, 1668, 1594, 1436, 1244, 1225, 1162, 1040, 961, 864 cm^{-1}.

ethyl 1-(6-(pentafluoro-λ^6-sulfaneyl)-2-azabicyclo[3.2.0]hepta-2,6-dien-3-yl)piperidine-4-carboxylate (4as)

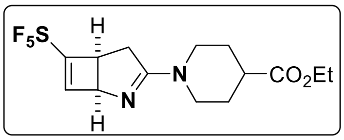

According to **General Procedure D**, 3*H*-azepin-2-amine **3as** (41.0 mg, 0.11 mmol, 1.0 equiv) was dissolved in freshly dry degassed MeCN (3.3 mL, 0.033 M). The reaction mixture was stirred under irradiation (365 nm) for 40 hours. **4as** was obtained as a colorless oil (25.5 mg, 62%) using *n*-Hexane/EtOAc/Et$_3$N (50/50/1%). **^{1}H-NMR** (500 MHz, $CDCl_3$) δ 6.57 (t, *J* = 1.7 Hz, 1H), 4.85 – 4.68 (m, 1H), 4.15 (q, *J* = 7.1 Hz, 2H), 3.89 – 3.70 (m, 3H), 3.04 – 2.92 (m, 2H), 2.72 (ddq, *J* = 17.0, 3.2, 1.5 Hz, 1H), 2.61 (ddd, *J* = 16.9, 10.1, 1.1 Hz, 1H), 2.49 (tt, *J* = 11.0, 3.9 Hz, 1H), 1.97 – 1.87 (m, 2H), 1.69 (dqd, *J* = 13.3, 11.2, 4.2 Hz, 2H), 1.26 (t, *J* = 7.2 Hz, 3H). **^{13}C-NMR** (126 MHz, $CDCl_3$) δ 174.4, 168.3, 154.0 (quint, *J* = 15.4 Hz), 143.5 – 142.8 (m), 69.4, 60.7, 48.8, 45.4, 41.1, 32.8, 27.8 (d, *J* = 18.7 Hz), 14.3. **^{19}F-NMR** (658 MHz, $CDCl_3$) δ 79.54 (quint, *J* = 150.3 Hz, 1F), 57.14 (d, *J* = 150.1 Hz, 4F). **HRMS** (ESI) calculated for $C_{14}H_{20}F_5N_2O_2S$ [M+H]$^+$: 375.1166, found 375.1172. **IR (KBr)**: *v* = 2932, 2857, 1595, 1450, 1376, 1223, 1099, 1042, 965, 938 cm^{-1}.

N-(4-bromobenzyl)-6-(pentafluoro-λ⁶-sulfaneyl)-2-azabicyclo[3.2.0]hepta-2,6-dien-3-amine (4az)

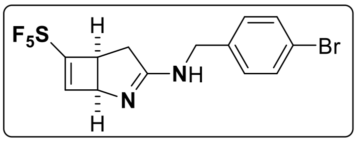

According to **General Procedure D**, 3*H*-azepin-2-amine **3az** (80.0 mg, 0.2 mmol, 1.0 equiv) was dissolved in freshly dry degassed MeCN (6.0 mL, 0.033 M). The reaction mixture was stirred under irradiation (365 nm) for 40 hours. **4az** was obtained as a yellow solid (46.0 mg, 58%) using *n*-Hexane/EtOAc/Et_3N (80/20/5%). **m.p.**: 119.1 – 120.9 °C. **^{1}H-NMR** (500 MHz, $CDCl_3$) δ 7.48 – 7.44 (m, 2H), 7.20 – 7.16 (m, 2H), 6.59 (d, *J* = 1.7 Hz, 1H), 4.88 – 4.80 (m, 1H), 4.42 (q, 2H), 3.85 – 3.80 (m, 1H), 2.69 (ddd, *J* = 16.8, 3.1, 1.6 Hz, 1H), 2.60 (ddd, *J* = 17.0, 9.9, 1.2 Hz, 1H). **^{13}C-NMR** (126 MHz, $CDCl_3$) δ 166.8, 154.2 (quint), 143.0 (t), 137.5, 131.9, 129.4, 121.5, 70.0, 48.7, 46.3, 33.6. **^{19}F-NMR** (376 MHz, $CDCl_3$) δ 79.33 (quint, *J* = 150.3 Hz, 1F), 57.08 (d, *J* = 150.4 Hz, 4F). **HRMS** (ESI) calculated for $C_{13}H_{13}BrF_5N_2S$ $[M+H]^+$: 402.9903, found 404.9891. **IR (KBr)**: *ν* = 3060, 2923, 2853, 1895, 1614, 1564, 1428, 1404, 1224, 1156, 1013, 986, 857 cm^{-1}.

1-benzyl-5-(pentafluoro-λ⁶-sulfaneyl)-2-(trifluoromethyl)-1*H*-benzo[*d*]imidazole (5a)

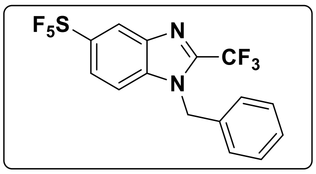

According to **General Procedure E**, **3aw** (32.4 mg, 0.1 mmol, 1.0 equiv) and DABCO (12.3 mg, 0.11 mmol, 1.1 equiv) were dissolved in toluene (2.0 mL, 0.05 M). Trifluoroacetic anhydride (0.22 mmol, 2.2 equiv) was added to the reaction mixture and the reaction tube was stirred at 120 °C. After 6 hours, another 1.1 equiv of the trifluoroacetic anhydride was added to the reaction vial for another 24 hours. The crude residues were purified via column chromatography using *n*-Hexane/DCM (70/30). Compound **5a** was obtained as a white solid (21.2 mg, 53%). **m.p.**: 98.2 – 98.8 °C. **^{1}H-NMR** (500 MHz, $CDCl_3$) δ 8.35 (d, *J* = 2.1 Hz, 1H), 7.75 (dd, *J* = 9.1, 2.1 Hz, 1H), 7.37 – 7.33 (m, 3H), 7.32 (d, *J* = 9.0 Hz, 1H), 7.11 (dd, *J* = 7.4, 2.1 Hz, 2H), 5.57 (s, 2H). **^{13}C-NMR** (126 MHz, $CDCl_3$) δ 150.1 (quint, *J* = 17.3 Hz), 143.6 (q, *J* = 38.9 Hz), 140.2, 136.9, 134.1, 129.4, 128.9, 126.5, 123.3, 120.9, 118.8 (q, *J* = 272.6 Hz), 111.3, 49.1. **^{19}F-NMR** (658 MHz, $CDCl_3$) δ 84.24 (quint, *J* = 150.5 Hz, 1F), 64.45 (d, *J* = 150.1 Hz, 4F), -62.44 (s, 3F). **HRMS** (ESI) calculated for $C_{15}H_{11}F_8N_2S$ $[M+H]^+$: 403.0515, found 403.0516. **IR (KBr)**: *ν* = 3080, 1616, 1529, 1099, 930, 804, 728 cm^{-1}.

1-benzyl-2-(difluoromethyl)-5-(pentafluoro-λ⁶-sulfaneyl)-1*H*-benzo[*d*]imidazole (5b)

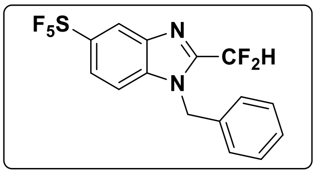

According to **General Procedure E**, **3aw** (32.4 mg, 0.1 mmol, 1.0 equiv) and DABCO (12.3 mg, 0.11 mmol, 1.1 equiv) were dissolved in toluene (2.0 mL, 0.05 M). Difluoroacetic anhydride (0.22 mmol, 2.2 equiv) was added to the reaction mixture and the reaction tube was stirred at 120 °C. After 6 hours, another 1.1 equiv of the difluoroacetic anhydride was added to the reaction vial for another 24 hours. The crude residues were purified via column chromatography using *n*-Hexane/EtOAc (90/10). Compound **5b** was obtained as a white solid (30.75 mg, 80%). **m.p.**: 97.5 – 98.4 °C. **^{1}H-NMR** (500 MHz, $CDCl_3$) δ 8.28 (d, *J* = 2.1 Hz, 1H), 7.70 (dd, *J* = 9.0, 2.1 Hz, 1H), 7.38 – 7.26 (m, 4H), 7.17 – 7.13 (m, 2H), 6.99 (t, *J* = 52.4 Hz, 1H), 5.61 (s, 2H). **^{13}C-NMR** (126 MHz, $CDCl_3$) δ 149.7 (quint, *J* = 17.2 Hz), 147.6 (t, *J* = 27.2 Hz), 140.9, 137.1, 134.6, 129.3, 128.7, 126.8, 122.6, 120.3, 111.1, 110.9 (t, *J* = 238.8 Hz), 48.8. **^{19}F-NMR** (376 MHz, $CDCl_3$) δ 84.76 (quint, 1F), 64.64 (d, *J* = 150.1 Hz, 4F), -113.99 (d, *J* = 53.0 Hz, 2F). **HRMS** (ESI) calculated for $C_{15}H_{12}F_7N_2S$ $[M+H]^+$: 385.0609, found 385.0627. **IR (KBr)**: *ν* = 3081, 1530, 1345, 1240, 1038, 832, 726 cm^{-1}.

2-(difluoromethyl)-1-(4-methoxybenzyl)-5-(pentafluoro-λ⁶-sulfaneyl)-1*H*-benzo[*d*]imidazole (5c)

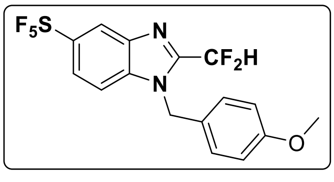

According to **General Procedure E**, **3ax** (35.4 mg, 0.1 mmol, 1.0 equiv) and DABCO (12.3 mg, 0.11 mmol, 1.1 equiv) were dissolved in toluene (2.0 mL, 0.05 M). Difluoroacetic anhydride (0.22 mmol, 2.2 equiv) was added to the reaction mixture and the reaction tube was stirred at 120 °C. After 6 hours, another 1.1 equiv of the difluoroacetic anhydride was added to the reaction vial for another 24 hours. The crude residues were purified via column chromatography using *n*-Hexane/EtOAc (90/10). Compound **5c** was obtained as a white solid (30.85 mg, 74%). **m.p.**: 117.5 – 118.8 °C. **^{1}H-NMR** (500 MHz, $CDCl_3$) δ 8.27 (d, *J* = 2.0

Hz, 1H), 7.70 (dd, J = 9.1, 2.1 Hz, 1H), 7.30 (d, J = 9.0 Hz, 1H), 7.12 (d, J = 8.7 Hz, 2H), 6.99 (t, J = 52.4 Hz, 1H), 6.86 (d, J = 8.7 Hz, 2H), 5.54 (s, 2H), 3.78 (s, 3H). **^{13}C-NMR** (126 MHz, $CDCl_3$) δ 159.8, 149.6 (quint, J = 17.1 Hz), 147.5 (t, J = 27.1 Hz), 141.0, 137.0, 128.3, 126.6, 122.5, 120.2, 114.6, 111.2, 110.9 (t, J = 238.8 Hz), 55.4, 48.5. **^{19}F-NMR** (376 MHz, $CDCl_3$) δ 84.83 (quint, 1F), 64.66 (d, J = 150.1 Hz, 4F), -113.89 (d, J = 52.1 Hz, 2F). **HRMS** (ESI) calculated for $C_{16}H_{14}F_7N_2OS$ $[M+H]^+$: 415.0715, found 415.0727. **IR (KBr)**: ν = 3077, 3008, 2845, 1614, 1515, 1254, 1031, 833, 751 cm^{-1}.

2-(difluoromethyl)-1-(4-fluorobenzyl)-5-(pentafluoro-λ^6-sulfaneyl)-1*H*-benzo[*d*]imidazole (5d)

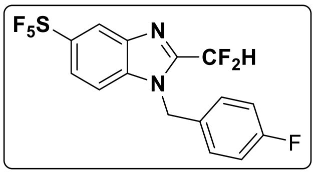

According to **General Procedure E**, **3ay** (34.2 mg, 0.1 mmol, 1.0 equiv) and DABCO (12.3 mg, 0.11 mmol, 1.1 equiv) were dissolved in toluene (2.0 mL, 0.05 M). Difluoroacetic anhydride (0.22 mmol, 2.2 equiv) was added to the reaction mixture and the reaction tube was stirred at 120 °C. After 6 hours, another 1.1 equiv of the difluoroacetic anhydride was added to the reaction vial for another 24 hours. The crude residues were purified via column chromatography using *n*-Hexane/DCM (50/50). Compound **5d** was obtained as a white solid (29.8 mg, 74%). **m.p.**: 102.7 – 103.8 °C. **^{1}H-NMR** (500 MHz, $CDCl_3$) δ 8.29 (d, J = 2.1 Hz, 1H), 7.72 (dd, J = 9.0, 2.1 Hz, 1H), 7.28 (d, J = 9.1 Hz, 1H), 7.17 – 7.13 (m, 2H), 7.04 (t, J = 8.6 Hz, 2H), 6.99 (t, J = 52.4 Hz, 1H), 5.58 (s, 2H). **^{13}C-NMR** (126 MHz, $CDCl_3$) δ 162.7 (d, J = 247.9 Hz), 149.8 (quint, J = 17.9 Hz), 147.5 (t, J = 27.2 Hz), 141.0, 136.9, 130.4, 128.6 (d, J = 8.4 Hz), 122.8, 120.4, 116.3 (d, J = 21.9 Hz), 110.9 (t, J = 240.7 Hz), 48.2. **^{19}F-NMR** (376 MHz, $CDCl_3$) δ 84.67 (quint, 1F), 64.64 (d, J = 150.2 Hz, 4F), -113.43 (tt, J = 8.9, 5.1 Hz, 1F), -113.81 (d, J = 52.3 Hz, 2F). **HRMS** (ESI) calculated for $C_{15}H_{11}F_8N_2S$ $[M+H]^+$: 403.0515, found 403.0533. **IR (KBr)**: ν = 3086, 1607, 1511, 1343, 1224, 1038, 831, 751, cm^{-1}.

1-(4-bromobenzyl)-2-(difluoromethyl)-5-(pentafluoro-λ^6-sulfaneyl)-1*H*-benzo[*d*]imidazole (5e)

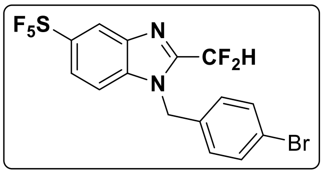

According to **General Procedure E**, **3az** (40.3 mg, 0.1 mmol, 1.0 equiv) and DABCO (12.3 mg, 0.11 mmol, 1.1 equiv) were dissolved in toluene (2.0 mL, 0.05 M). Difluoroacetic anhydride (0.22 mmol, 2.2 equiv) was added to the reaction mixture and the reaction tube was stirred at 120 °C. After 6 hours, another 1.1 equiv of the difluoroacetic anhydride was added to the reaction vial for another 24 hours. The crude residues were purified via column chromatography using *n*-Hexane/EtOAc (90/10). Compound **5e** was obtained as a white solid (37.4 mg, 80%). **m.p.**: 116.5 – 117.5 °C. **^{1}H-NMR** (500 MHz, $CDCl_3$) δ 8.29 (d, J = 2.1 Hz, 1H), 7.73 (dd, J = 9.0, 2.1 Hz, 1H), 7.47 (d, J = 8.5 Hz, 2H), 7.26 (t, J = 4.5 Hz, 1H), 7.02 (d, J = 8.6 Hz, 2H), 6.99 (t, J = 52.3 Hz, 1H), 5.56 (s, 2H). **^{13}C-NMR** (126 MHz, $CDCl_3$) δ 149.8 (quint, J = 17.3 Hz), 147.5 (t, J = 27.2 Hz), 140.9, 136.8, 133.7, 132.5, 128.4, 122.8, 122.7, 120.4, 110.9 (t, J = 241.3 Hz), 110.8, 48.2. **^{19}F-NMR** (376 MHz, $CDCl_3$) δ 84.56 (quint, 1F), 64.60 (d, J = 150.3 Hz, 4F), -113.82 (d, J = 53.2 Hz, 2F). **HRMS** (ESI) calculated for $C_{15}H_{11}BrF_7N_2S$ $[M+H]^+$: 462.9715, found 462.9721. **IR (KBr)**: ν = 3082, 1617, 1405, 1343, 1241, 1037, 926, 635 cm^{-1}.

1-(3,4-dichlorobenzyl)-2-(difluoromethyl)-5-(pentafluoro-λ^6-sulfaneyl)-1*H*-benzo[*d*]imidazole (5f)

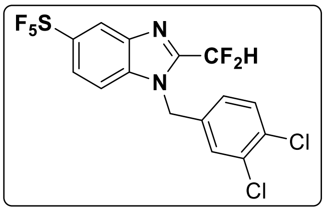

According to **General Procedure E**, **3aaa** (39.3 mg, 0.1 mmol, 1.0 equiv) and DABCO (12.3 mg, 0.11 mmol, 1.1 equiv) were dissolved in toluene (2.0 mL, 0.05 M). Difluoroacetic anhydride (0.22 mmol, 2.2 equiv) was added to the reaction mixture and the reaction tube was stirred at 120 °C. After 6 hours, another 1.1 equiv of the difluoroacetic anhydride was added to the reaction vial for another 72 hours. The crude residues were purified via column chromatography using *n*-Hexane/EtOAc (10/1) and *n*-Hexane/EtOAc (90/10). Compound **5e** was obtained as a yellow solid (28.95 mg, 64%). **m.p.**: 77.2 – 78.8 °C. **^{1}H-NMR** (500 MHz, $CDCl_3$) δ 8.30 (d, J = 2.1 Hz, 1H), 7.76 (dd, J = 9.0, 2.1 Hz, 1H), 7.41 (d, J = 8.3 Hz, 1H), 7.31 – 7.23 (m, 2H), 7.00 (t, J = 52.3 Hz, 1H), 6.94 (dd, J = 8.3, 2.2 Hz, 1H), 5.56 (s, 2H). **^{13}C-NMR** (126 MHz, $CDCl_3$) δ 150.0 (quint, J = 17.9 Hz), 147.4 (t, J = 27.3 Hz), 141.0, 136.7, 134.8, 133.6, 133.0, 131.3, 128.7, 125.9, 123.0, 120.6, 110.9 (t, J = 238.6 Hz), 110.6, 47.6. **^{19}F-NMR** (376 MHz, $CDCl_3$) δ 84.45 (quint, 1F), 64.60 (d, J = 149.9 Hz, 4F), -113.66

(d, J = 52.3 Hz, 2F). **HRMS** (ESI) calculated for $C_{15}H_{10}Cl_2F_7N_2S$ $[M+H]^+$: 452.9830, found 452.9837. **IR (KBr)**: ν = 3118, 2963, 1475, 1340, 1239, 1036, 930, 672 cm^{-1}.

4-(pentafluoro-λ^6-sulfaneyl)aniline (13a)

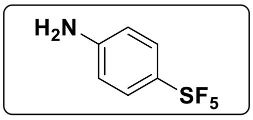

The data of compound **14a** was in accordance with previous literature.[5] **^{1}H-NMR** (700 MHz, $CDCl_3$) δ 7.52 (d, J = 9.0 Hz, 2H), 6.61 (d, J = 8.3 Hz, 2H), 3.99 (s, 2H). **^{13}C-NMR** (176 MHz, $CDCl_3$) δ 149.1, 144.6 (quint), 127.6 (quint, J = 4.8 Hz), 113.5. **^{19}F-NMR** (658 MHz, $CDCl_3$) δ 87.05 (quint, 1F), 64.00 (d, J = 150.1 Hz, 4F).

4. 2D NMR of Compound (4az)

(a) ^1H-^1H COSY of **4az** (700 MHz, $CDCl_3$)

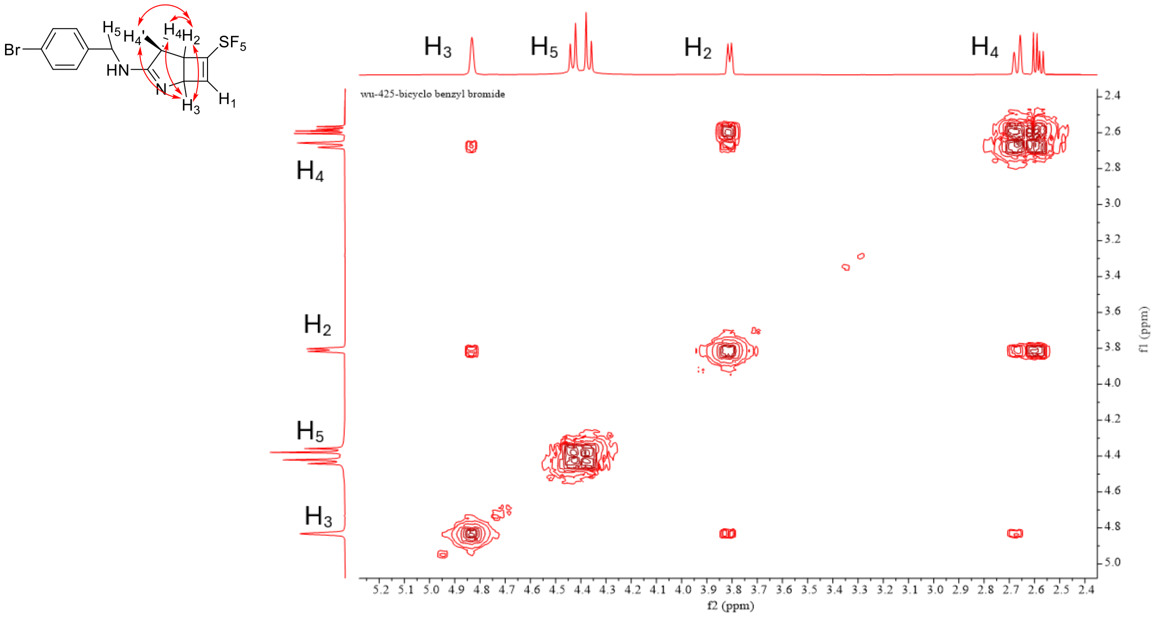

(b) NOESY spectra of **4az** (700 MHz, $CDCl_3$)

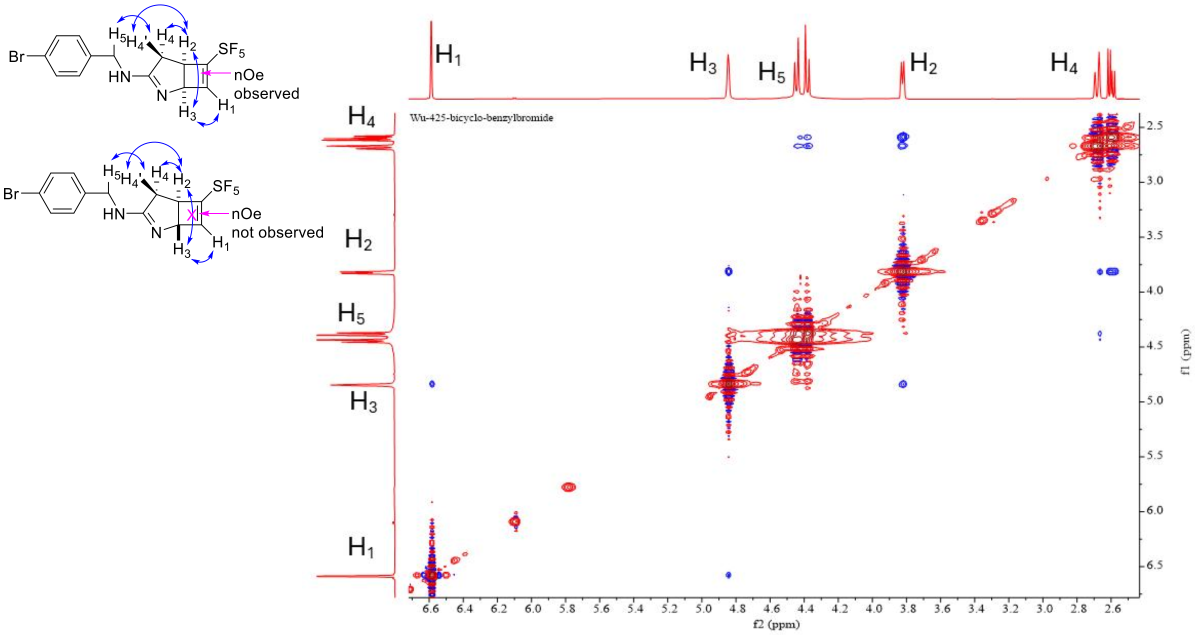

Figure S3. COSY and NOESY spectra of **4az**

.

5. References

1. K. Tanagawa, Z. Zhao, N. Saito, N. Shibata, *Bull. Chem. Soc. Jpn*. 2021, **94**, 1682–1684.
2. S. Hu, A. T. Radosevich, *Angew. Chem. Int. Ed.* 2024, **63**, e202409854.
3. T. V. Nykaza, T. S. Harrison, A. Ghosh, R. A. Putnik, A. T. Radosevich, *J. Am. Chem. Soc.* 2017, **139**, 6839−6842.
4. M. S. Faculak, A. M. Veatch, E. J. Alexanian, *Science*, 2024, **383**, 77–81.
5. J. T. Welch, D. S. Lim, *Bioorg. Med. Chem.* 2007, **15**, 6659–6666.

6. X-ray crystallography data (3av)

Data Collection

A colourless chip crystal of **3av** (**CCDC 2501744**) was prepared in *n*-hexane/dichloromethane (4/1) at room temperature, having approximate dimensions of 0.200 x 0.200 x 0.100 mm was mounted on a glass fiber. All measurements were made on a Rigaku R-AXIS RAPID diffractometer using graphite monochromated Mo-Ka radiation. The crystal-to-detector distance was 127.40 mm. Cell constants and an orientation matrix for data collection corresponded to a primitive orthorhombic cell with dimensions:

a = 28.613(3) Å
b = 8.978(1) Å
c = 11.687(2) Å
V = 3002.4(6) $Å^3$

For Z = 8 and F.W. = 350.35, the calculated density is 1.550 g/cm^3. The reflection conditions of:

0kl: k = 2n
h0l: l = 2n
hk0: h+k = 2n

uniquely determine the space group to be:

Pbcn (#60)

The data were collected at a temperature of -100 $\pm$ 1°C to a maximum 2θ value of 54.9°. A total of 44 oscillation images were collected. A sweep of data was done using ω scans from 130.0 to 190.0° in 5.0° step, at χ=45.0° and ϕ = 0.0° The exposure rate was 4.0 [sec./°]. A second sweep was performed using ω scans from 0.0 to 160.0° in 5.0° step, at χ =45.0° and ϕ = 180.0°. The exposure rate was 4.0 [sec./°]. The crystal-to-detector distance was 127.40 mm. Readout was performed in the 0.100 mm pixel mode.

X-Ray crystallographic structure of Product 3av

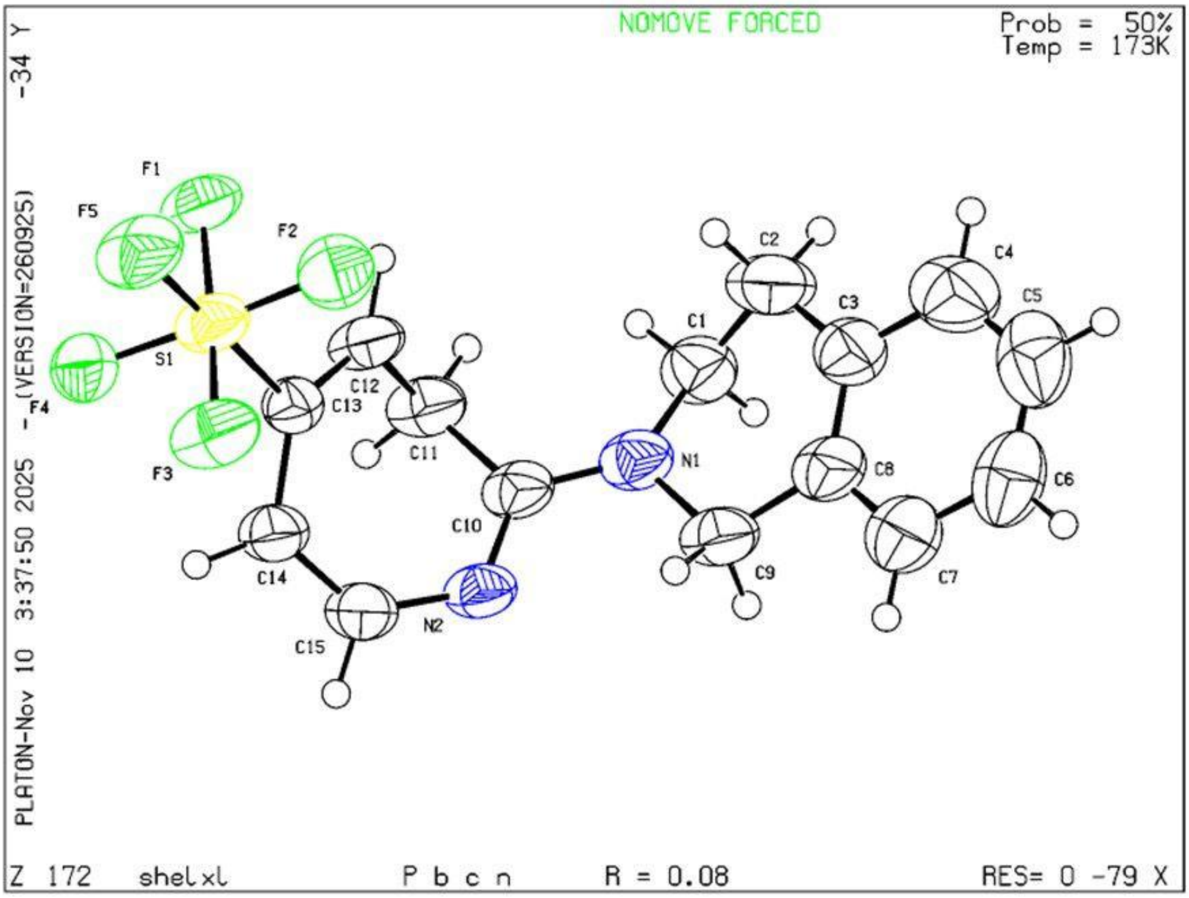

Figure S4: Ortep diagram of **3av**

7. NMR Spectral Data

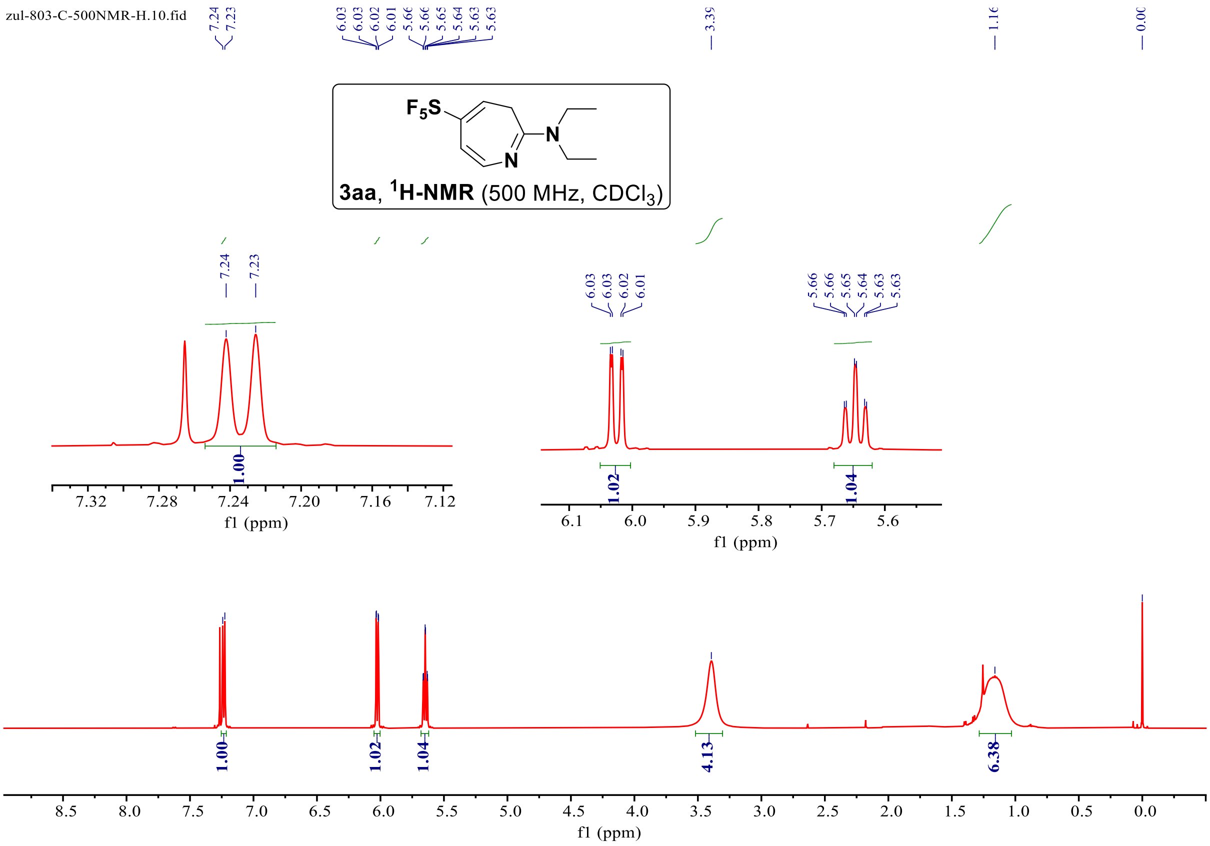

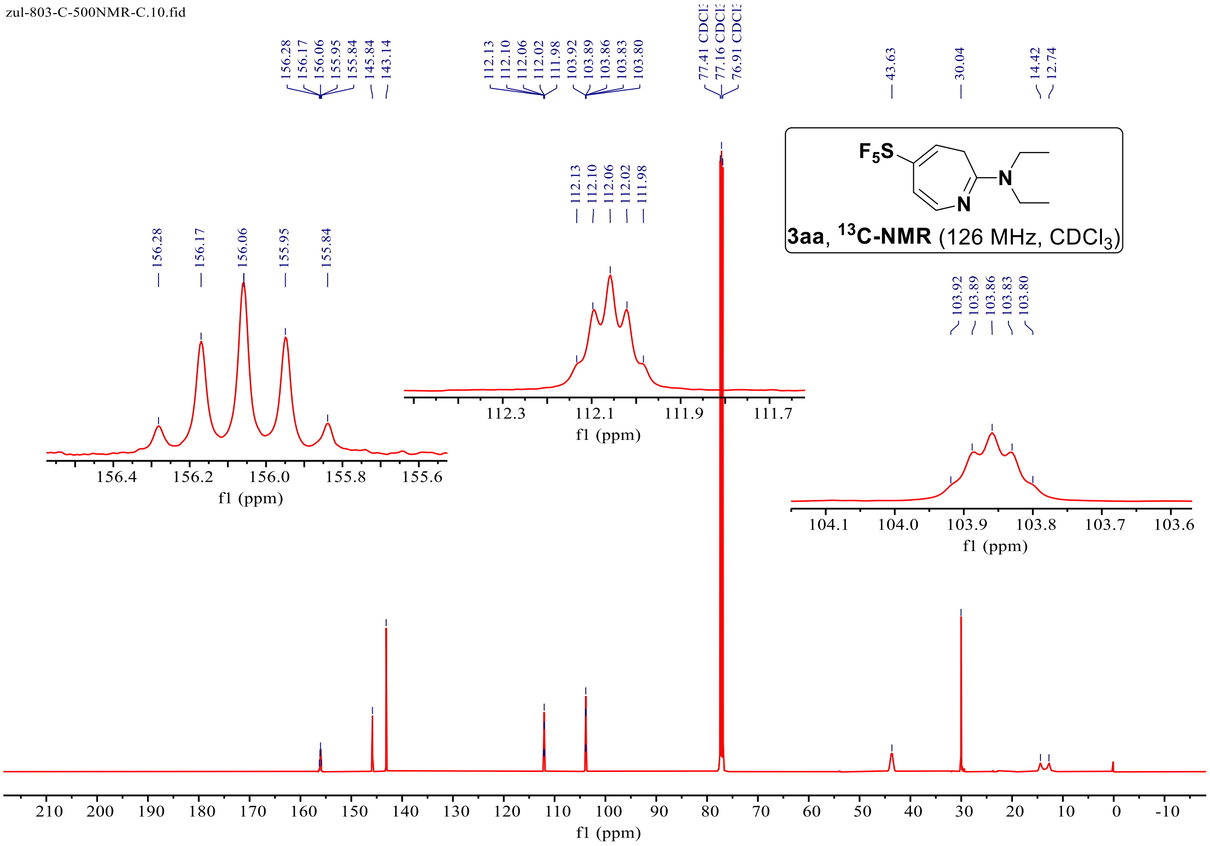

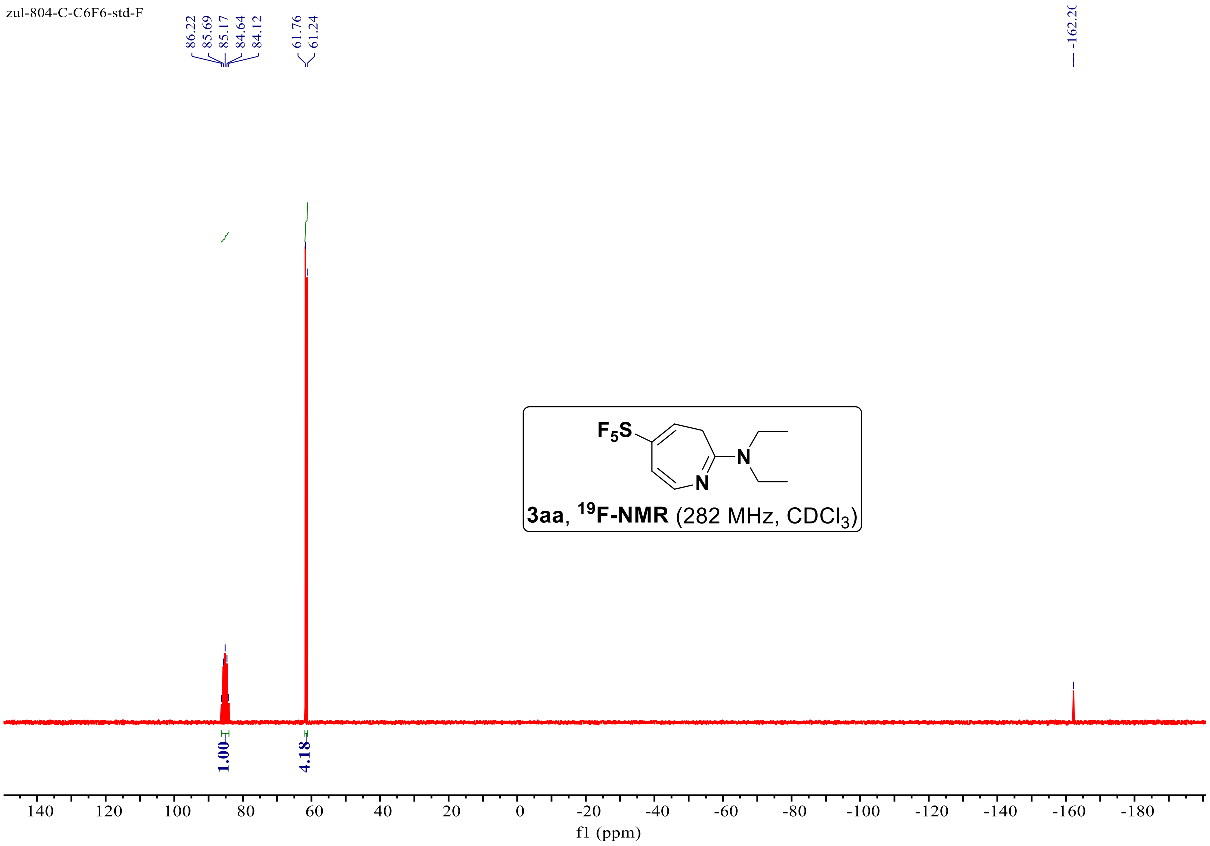

zul-804-C-C6F6-std-F
86.22
85.69
85.17
84.64
84.12
61.76
61.24
-162.20
F5S
N
N
3aa, 19F-NMR (282 MHz, CDCl3)
1.00
4.18
140 120 100 80 60 40 20 0 -20 -40 -60 -80 -100 -120 -140 -160 -180
f1 (ppm)

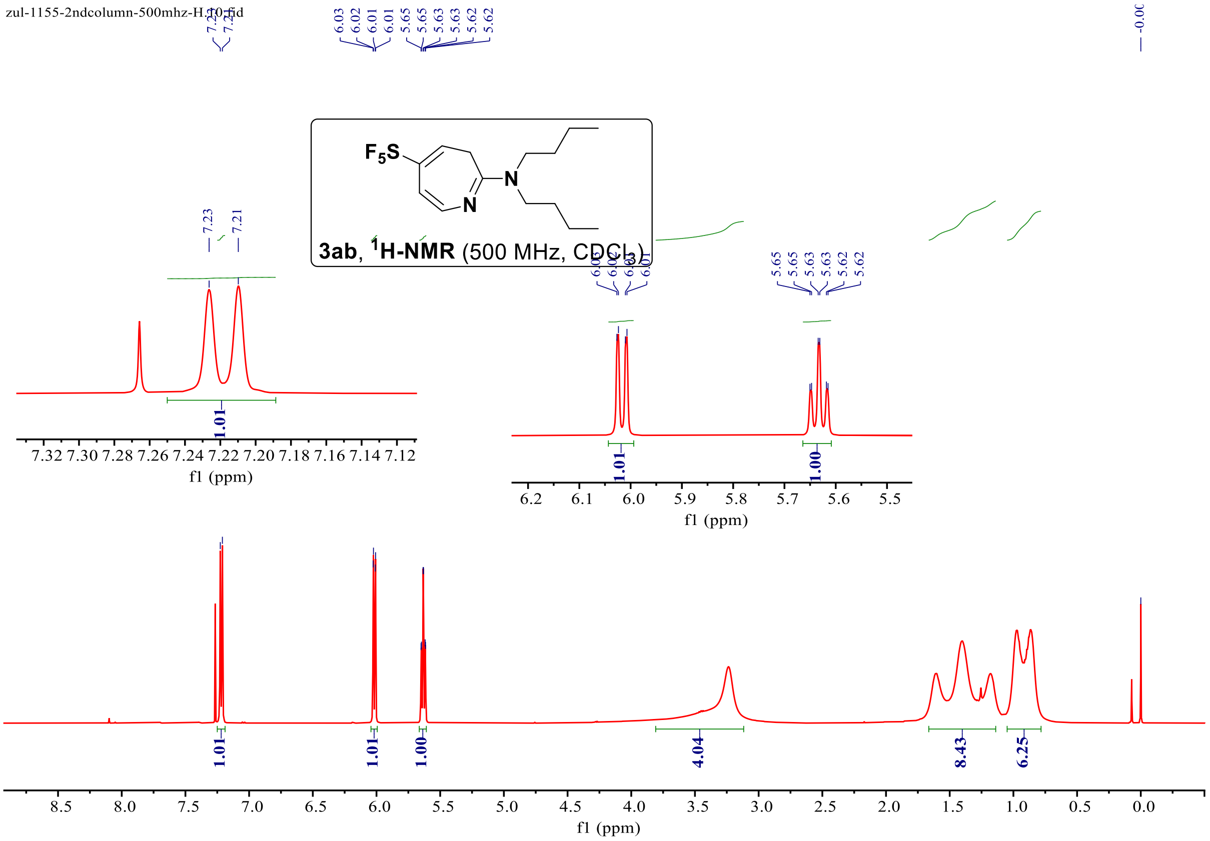

zul-1155-2ndcolumn-500mhz-H.10.fid
6.03
6.02
6.01
6.01
5.65
5.65
5.63
5.63
5.62
5.62
-0.00
F5S
N
N
3ab, 1H-NMR (500 MHz, CDCl3)
7.23
7.21
1.01
7.32 7.30 7.28 7.26 7.24 7.22 7.20 7.18 7.16 7.14 7.12
f1 (ppm)
5.65
5.65
5.63
5.63
5.62
5.62
1.01
1.00
6.2 6.1 6.0 5.9 5.8 5.7 5.6 5.5
f1 (ppm)
1.01
1.01
1.00
4.04
8.43
6.25
8.5 8.0 7.5 7.0 6.5 6.0 5.5 5.0 4.5 4.0 3.5 3.0 2.5 2.0 1.5 1.0 0.5 0.0
f1 (ppm)

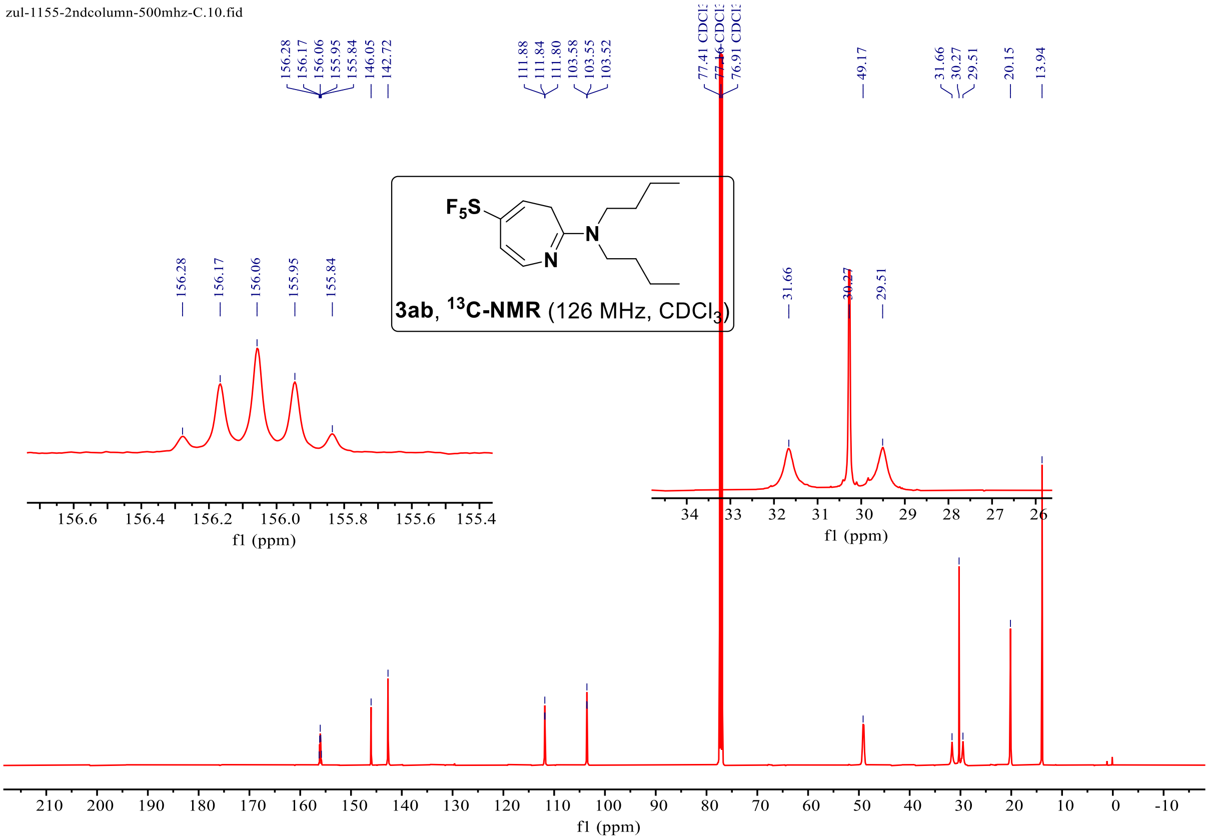
zul-1155-2ndcolumn-500mhz-C.10.fid
156.28
156.17
156.06
155.95
155.84
146.05
142.72
111.88
111.84
111.80
103.58
103.55
103.52
77.41 CDCl3
77.16 CDCl3
76.91 CDCl3
49.17
31.66
30.27
29.51
20.15
13.94
F5S
N
N
3ab, 13C-NMR (126 MHz, CDCl3)
f1 (ppm)

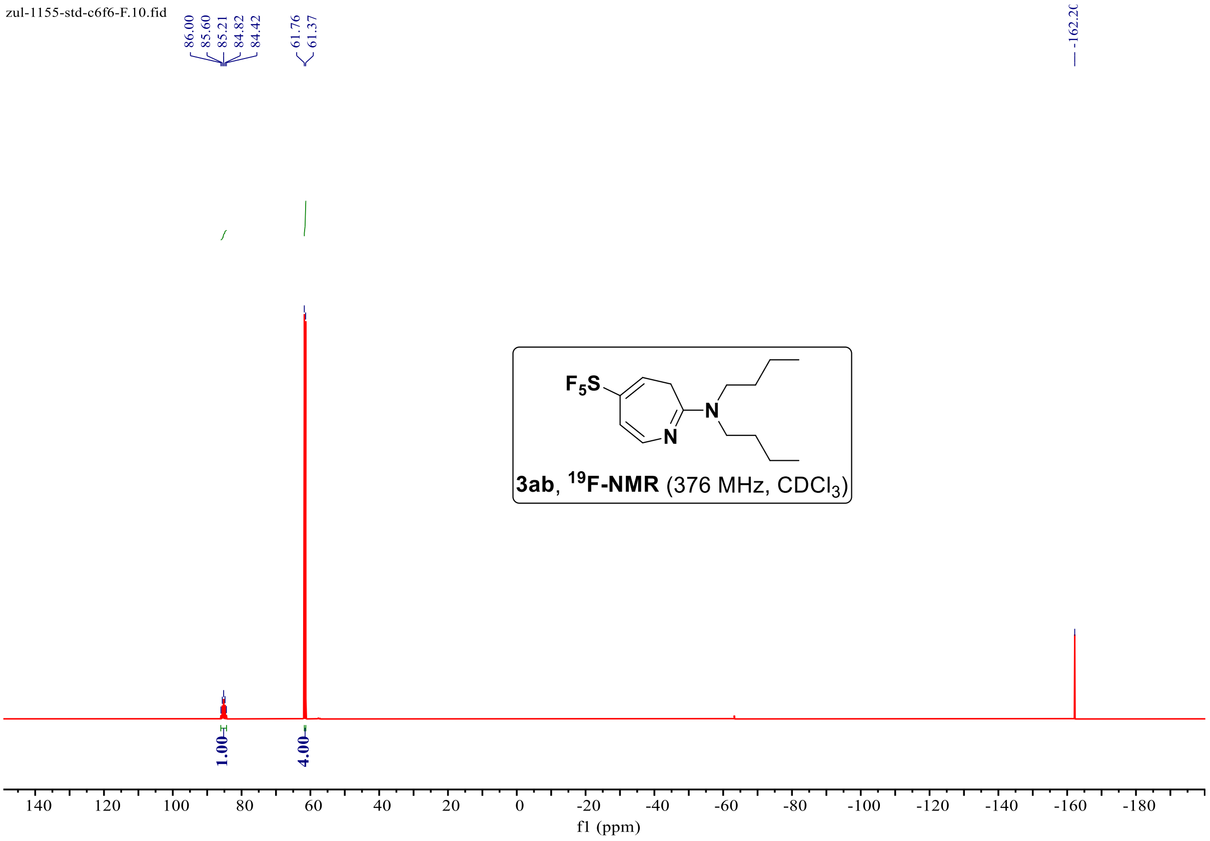
zul-1155-std-c6f6-F.10.fid
86.00
85.60
85.21
84.82
84.42
61.76
61.37
-162.20
F5S
N
N
3ab, 19F-NMR (376 MHz, CDCl3)
1.00
4.00
f1 (ppm)

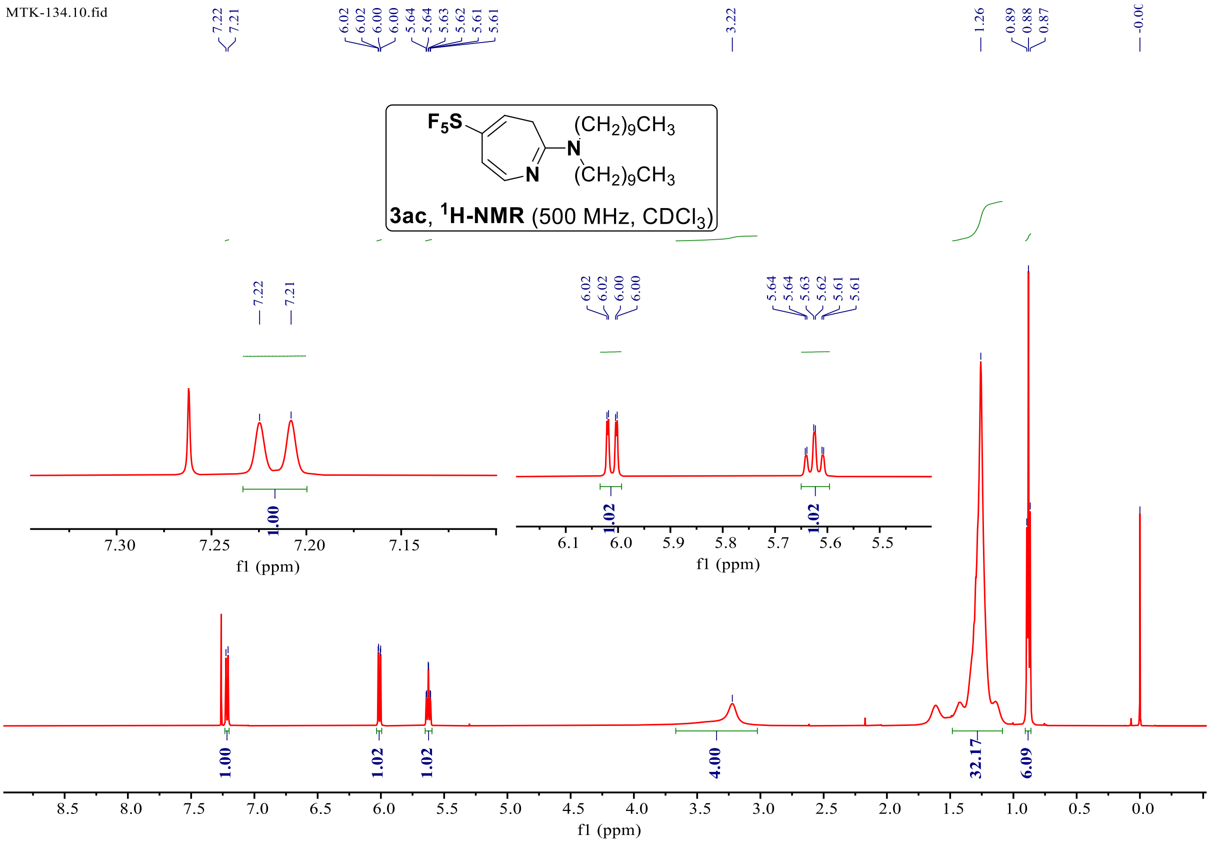
MTK-134.10.fid
3ac, 1H-NMR (500 MHz, CDCl3)
F5S
(CH2)9CH3
N
(CH2)9CH3
f1 (ppm)

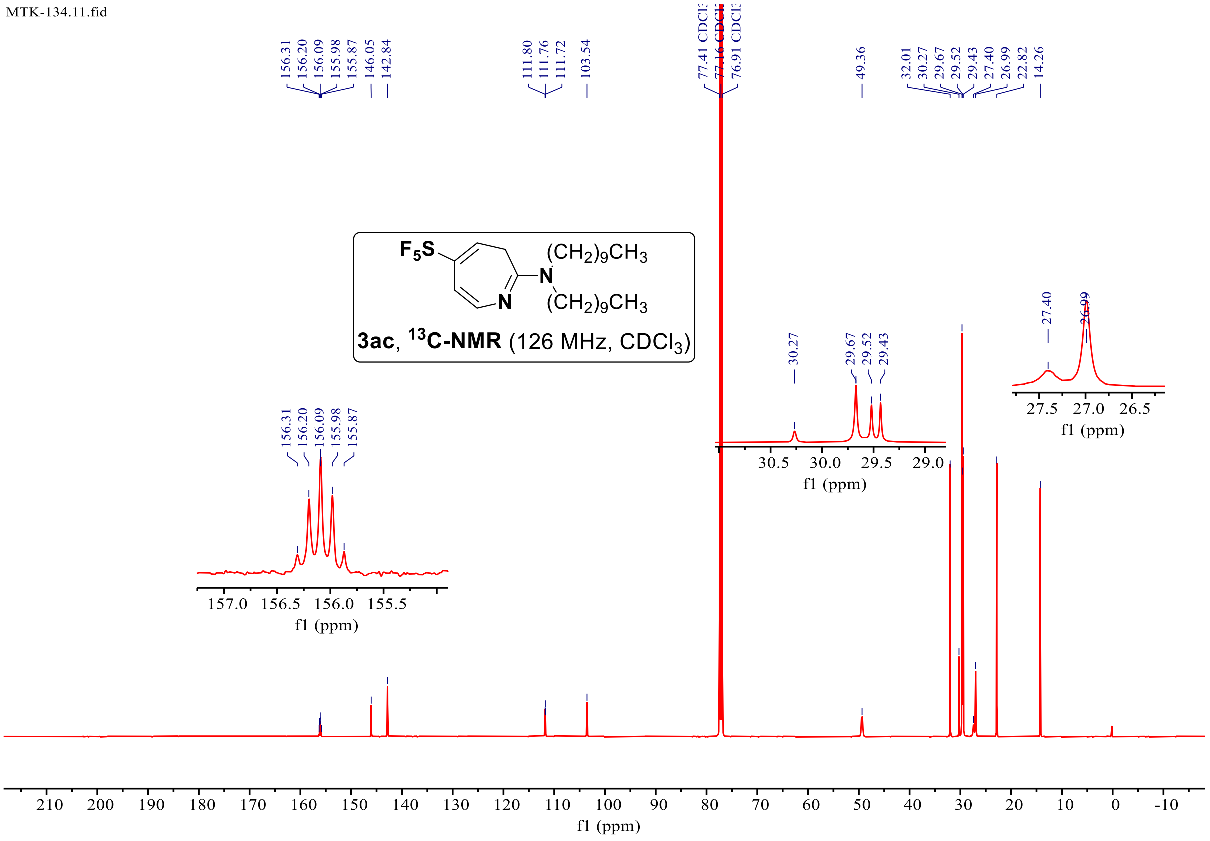
MTK-134.11.fid
3ac, 13C-NMR (126 MHz, CDCl3)
F5S
(CH2)9CH3
N
(CH2)9CH3
f1 (ppm)

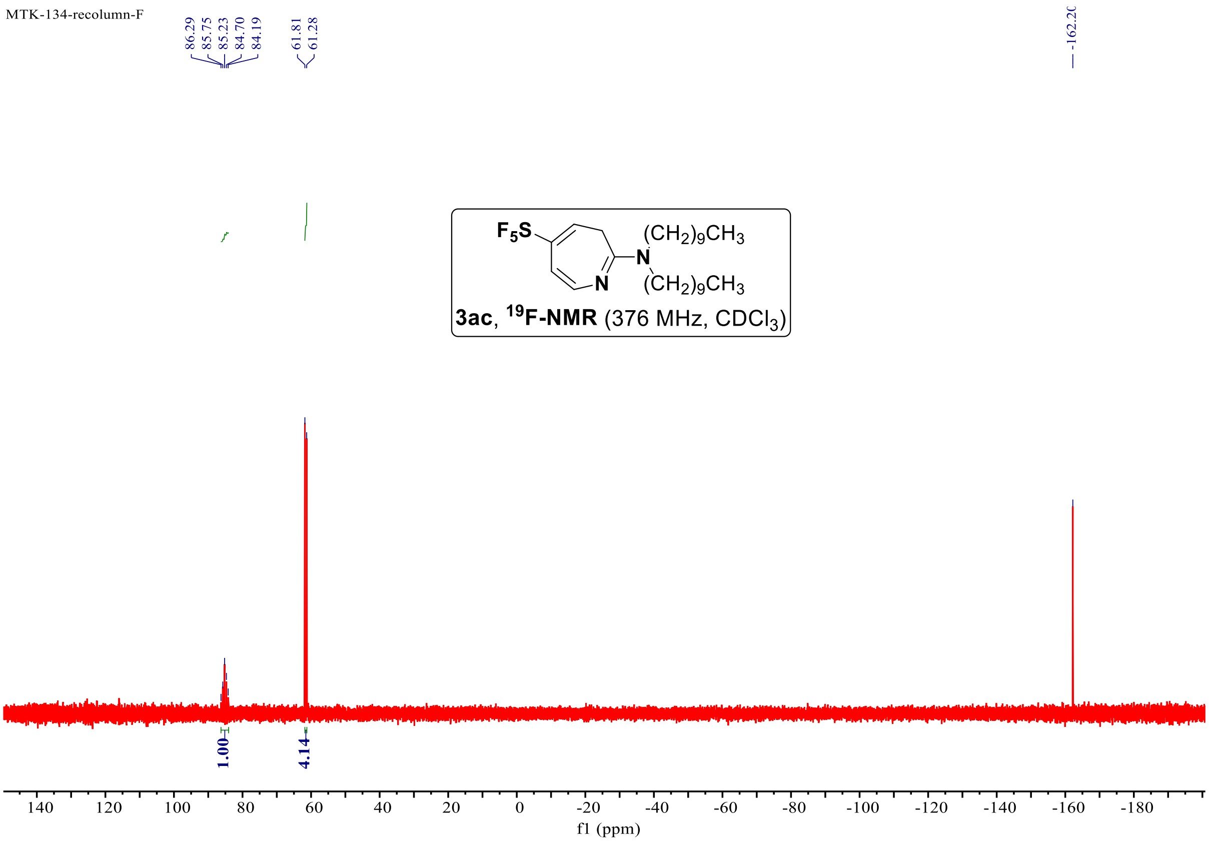
MTK-134-recolumn-F
86.29
85.75
85.23
84.70
84.19
61.81
61.28
-162.20
F5S
(CH2)9CH3
N
N
(CH2)9CH3
3ac, 19F-NMR (376 MHz, CDCl3)
1.00
4.14
140 120 100 80 60 40 20 0 -20 -40 -60 -80 -100 -120 -140 -160 -180
f1 (ppm)

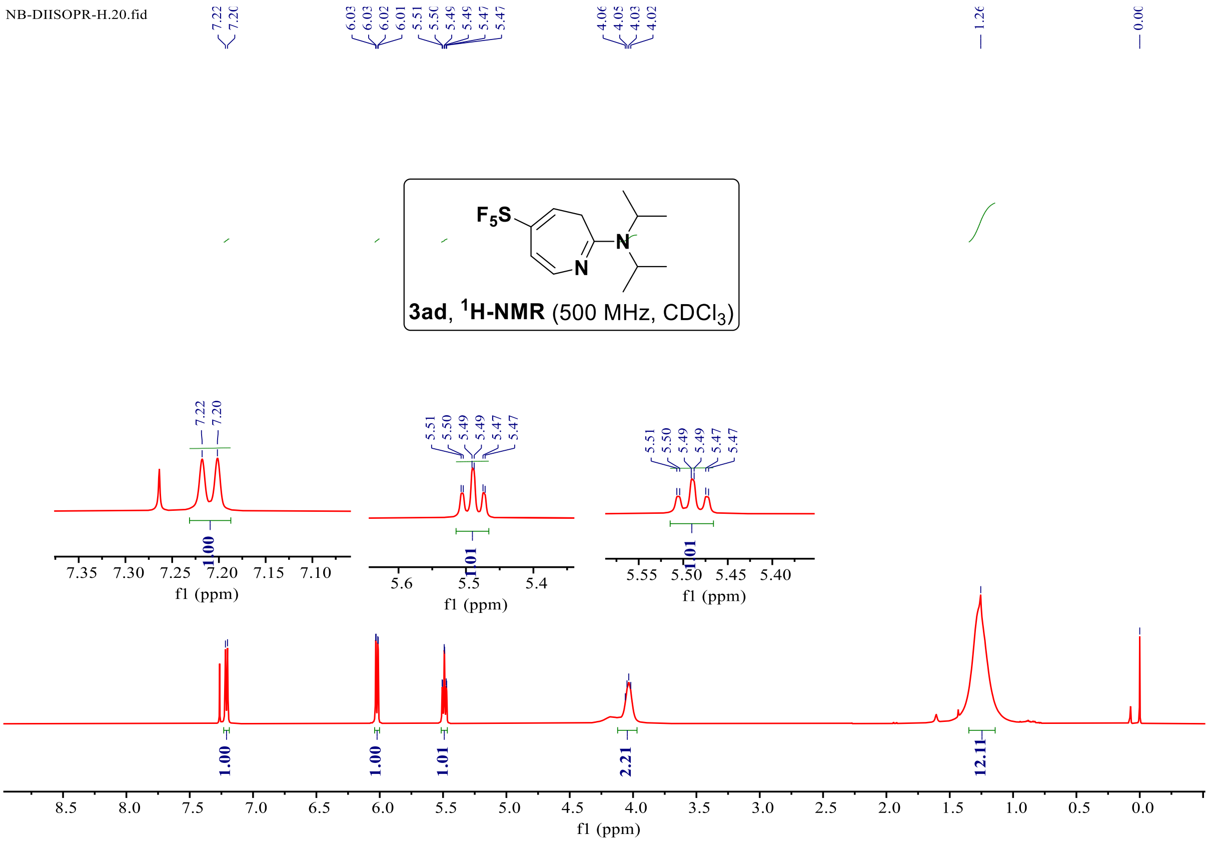
NB-DIISOPR-H.20.fid
7.22
7.20
6.03
6.03
6.02
6.01
5.51
5.50
5.49
5.49
5.47
5.47
4.06
4.05
4.03
4.02
1.26
0.00
F5S
N
N
3ad, 1H-NMR (500 MHz, CDCl3)
7.35 7.30 7.25 7.20 7.15 7.10
f1 (ppm)
5.6 5.5 5.4
f1 (ppm)
5.55 5.50 5.45 5.40
f1 (ppm)
1.00
1.00
1.01
2.21
12.11
8.5 8.0 7.5 7.0 6.5 6.0 5.5 5.0 4.5 4.0 3.5 3.0 2.5 2.0 1.5 1.0 0.5 0.0
f1 (ppm)

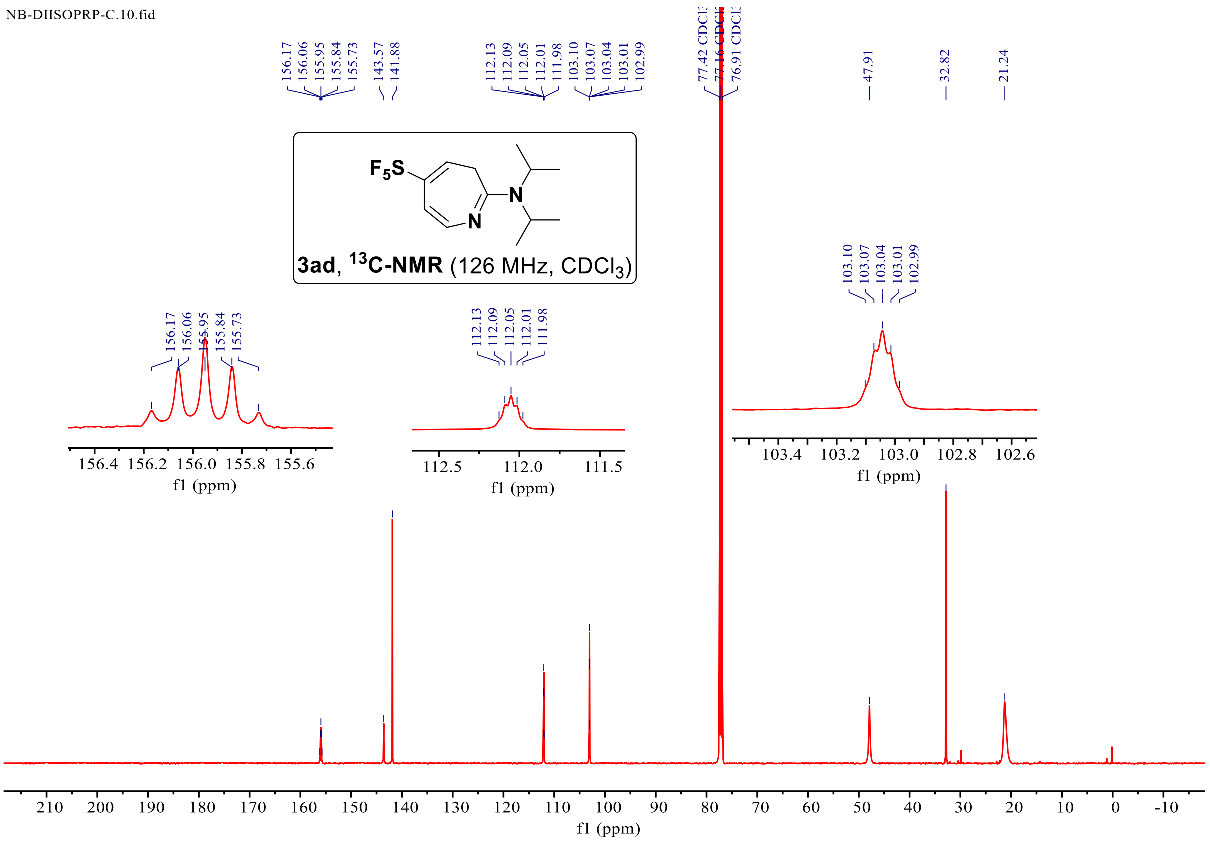
NB-DIISOPRP-C.10.fid
156.17 156.06 155.95 155.84 155.73 143.57 141.88
112.13 112.09 112.05 112.01 111.98 103.10 103.07 103.04 103.01 102.99
77.42 CDCl3 77.16 CDCl3 76.91 CDCl3
47.91 32.82 21.24
F5S
N
3ad, 13C-NMR (126 MHz, CDCl3)
f1 (ppm)

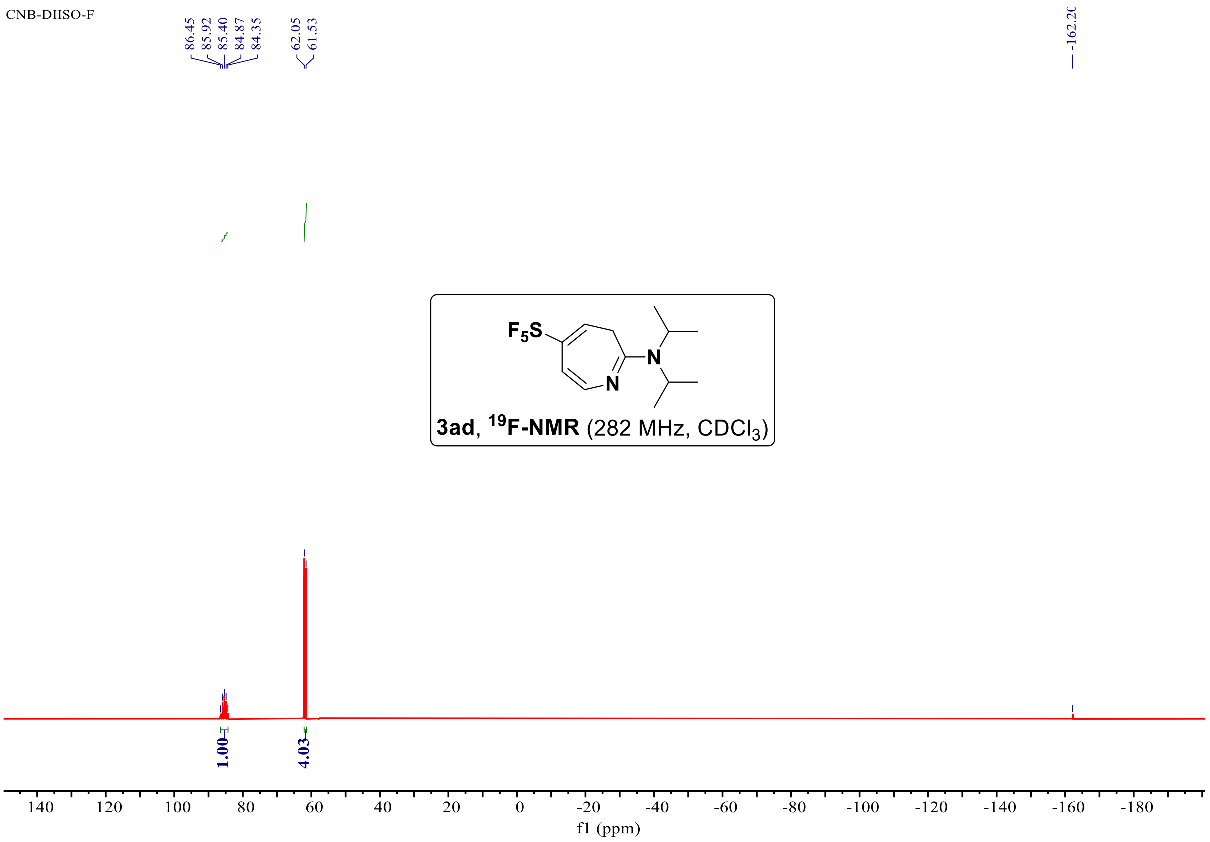
CNB-DIISO-F
86.45 85.92 85.40 84.87 84.35 62.05 61.53
-162.20
F5S
N
3ad, 19F-NMR (282 MHz, CDCl3)
1.00
4.03
f1 (ppm)

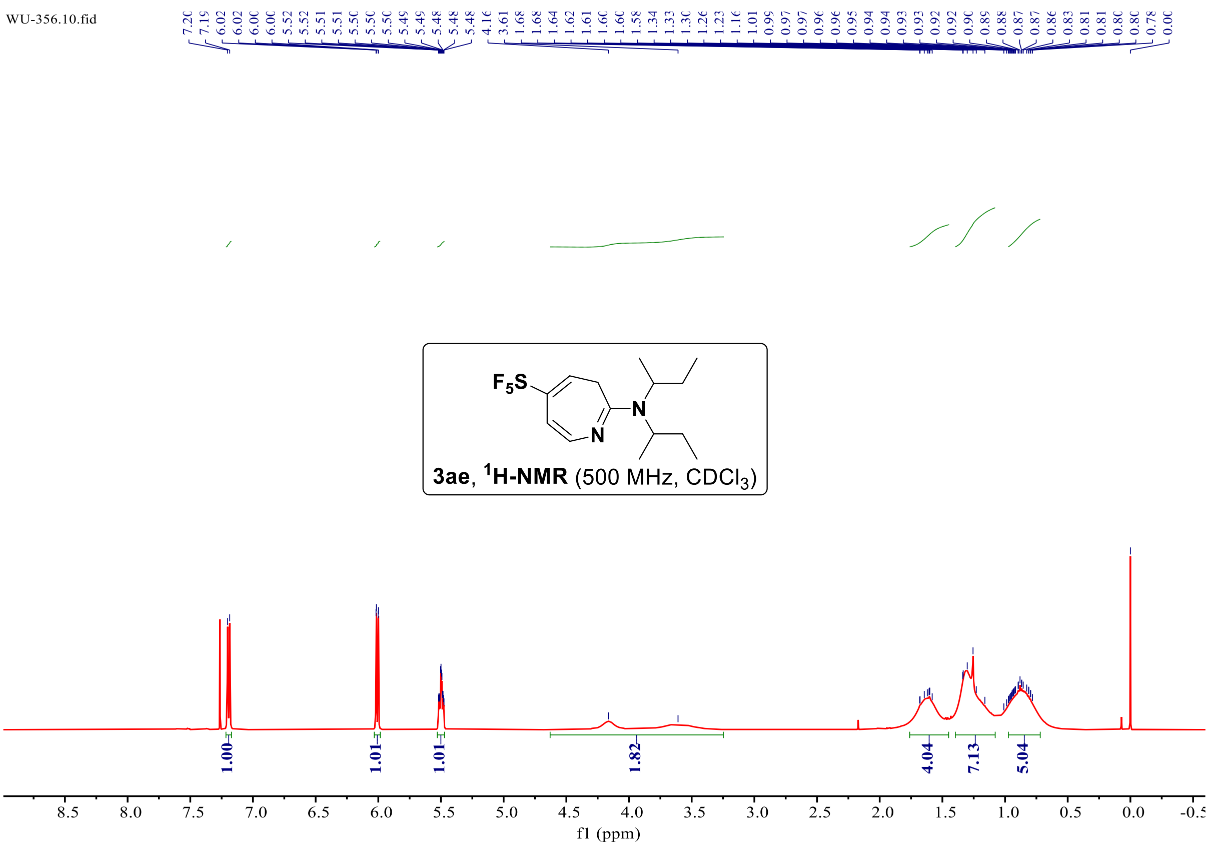
WU-356.10.fid
7.20 7.19 6.02 6.02 6.00 6.00 5.52 5.52 5.51 5.51 5.50 5.50 5.49 5.49 5.48 5.48 5.48 4.16 3.61 1.68 1.68 1.64 1.62 1.61 1.60 1.60 1.58 1.34 1.33 1.30 1.26 1.23 1.16 1.01 0.99 0.97 0.97 0.97 0.96 0.96 0.95 0.94 0.94 0.93 0.93 0.92 0.92 0.90 0.89 0.88 0.87 0.87 0.86 0.83 0.81 0.81 0.80 0.80 0.78 0.00
F_5S
N
N
3ae, ^{1}H-NMR (500 MHz, $CDCl_3$)
1.00
1.01
1.01
1.82
4.04
7.13
5.04
8.5 8.0 7.5 7.0 6.5 6.0 5.5 5.0 4.5 4.0 3.5 3.0 2.5 2.0 1.5 1.0 0.5 0.0 -0.5
f1 (ppm)

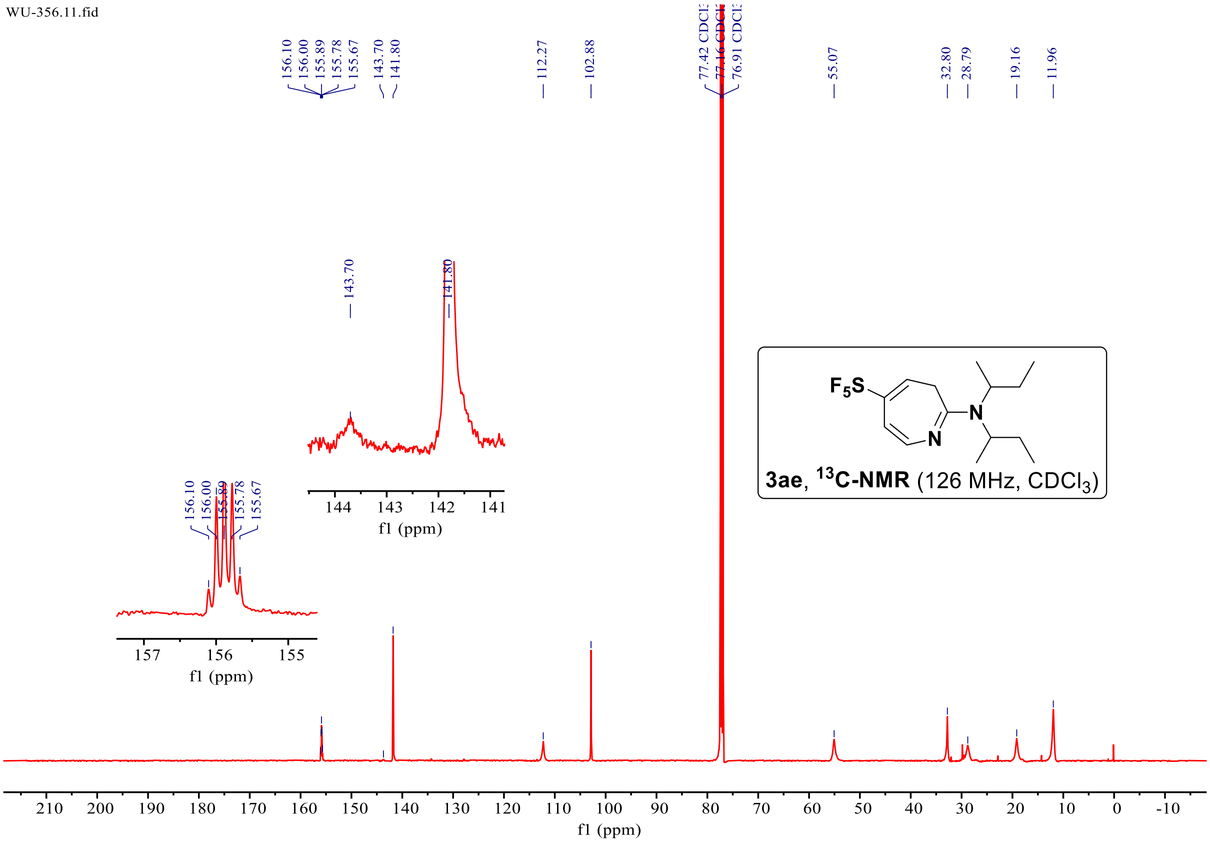
WU-356.11.fid
156.10 156.00 155.89 155.78 155.67 143.70 141.80
112.27
102.88
77.42 CDCl3 77.16 CDCl3 76.91 CDCl3
55.07
32.80 28.79
19.16
11.96
143.70
141.80
144 143 142 141
f1 (ppm)
156.10 156.00 155.89 155.78 155.67
157 156 155
f1 (ppm)
F_5S
N
N
3ae, ^{13}C-NMR (126 MHz, $CDCl_3$)
210 200 190 180 170 160 150 140 130 120 110 100 90 80 70 60 50 40 30 20 10 0 -10
f1 (ppm)

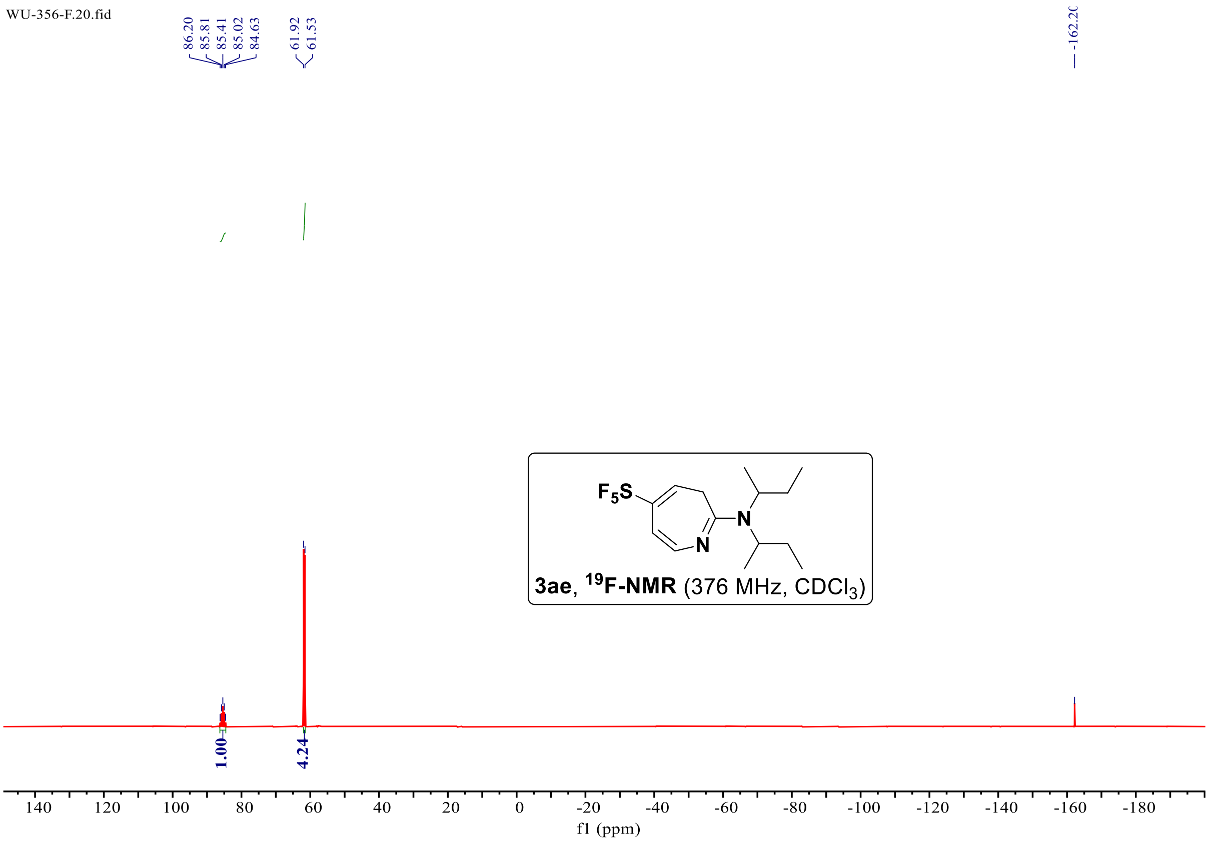
WU-356-F.20.fid
86.20
85.81
85.41
85.02
84.63
61.92
61.53
-162.20
3ae, ^{19}F-NMR (376 MHz, CDCl$_3$)
1.00
4.24
f1 (ppm)

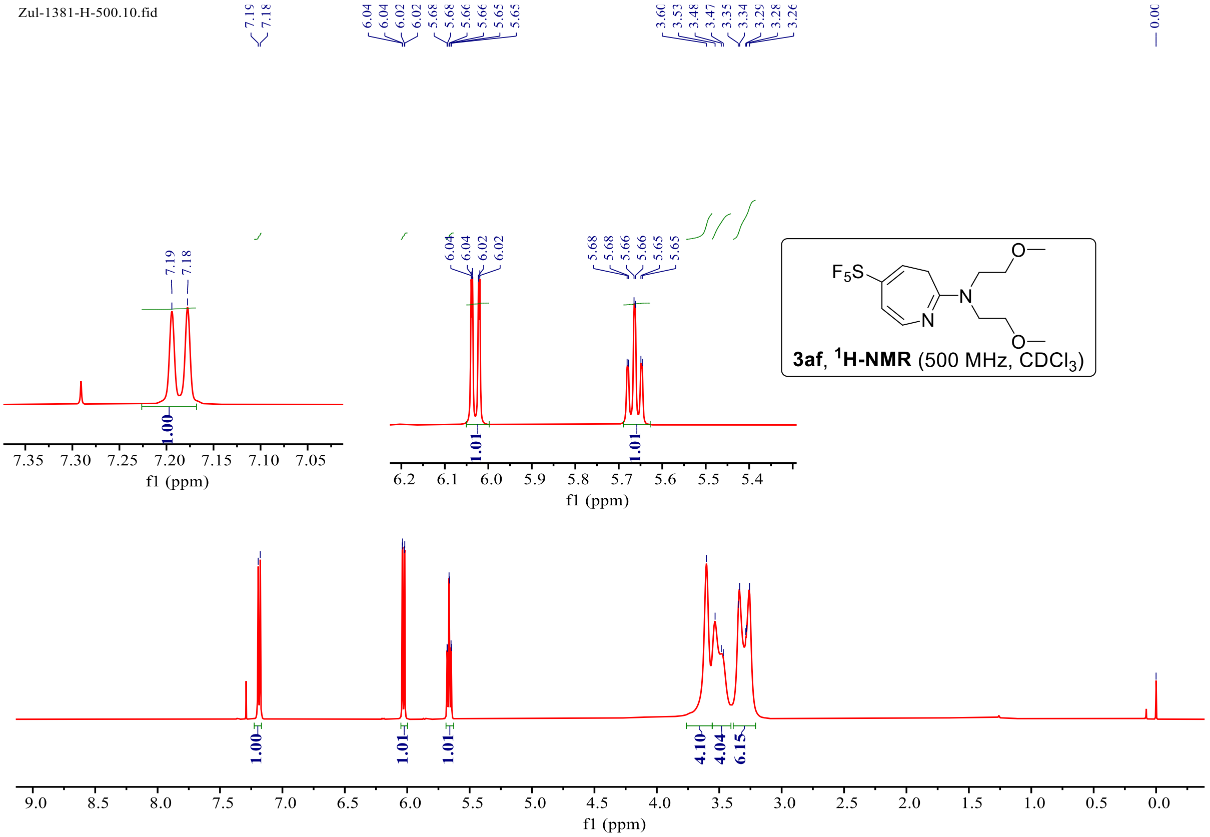
Zul-1381-H-500.10.fid
7.19
7.18
6.04
6.04
6.02
6.02
5.68
5.68
5.66
5.66
5.65
5.65
3.60
3.53
3.48
3.47
3.35
3.34
3.29
3.28
3.26
0.00
3af, ^{1}H-NMR (500 MHz, CDCl$_3$)
1.00
1.01
1.01
4.10
4.04
6.15
f1 (ppm)

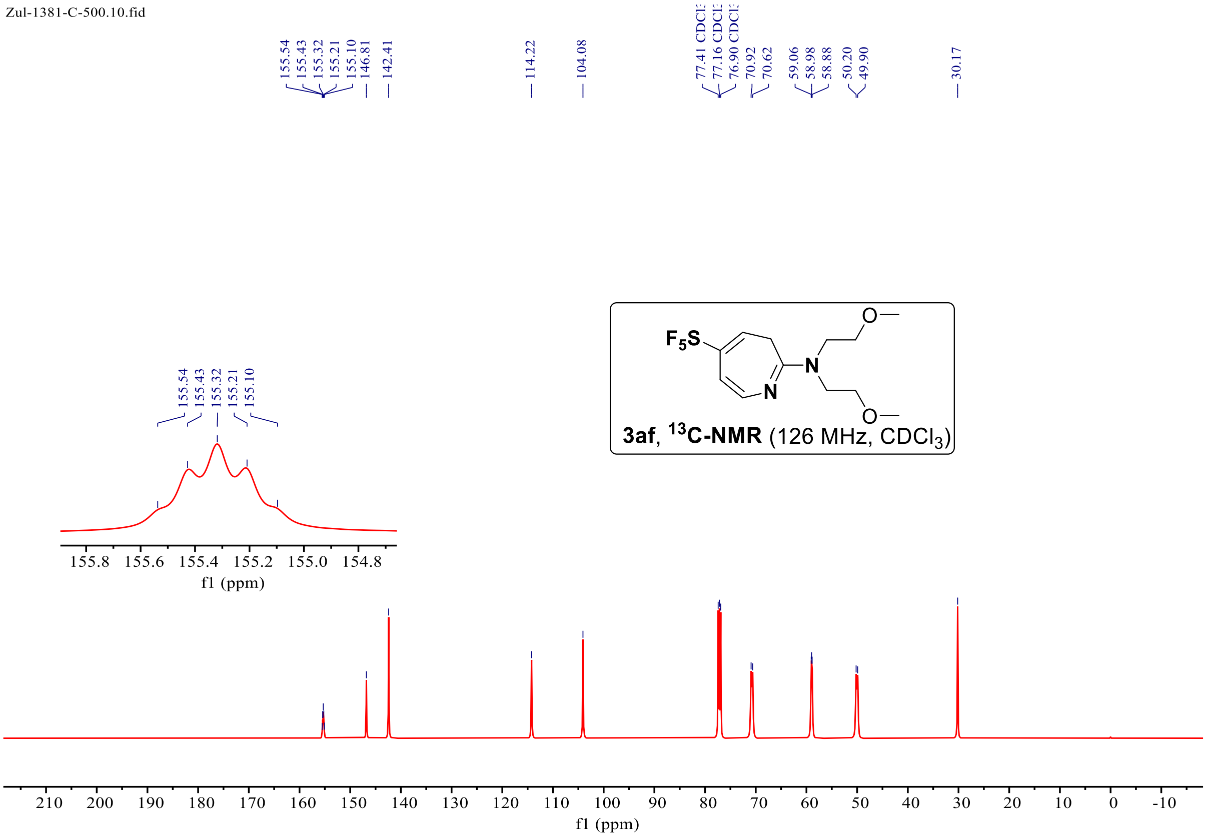
Zul-1381-C-500.10.fid
155.54
155.43
155.32
155.21
155.10
146.81
142.41
114.22
104.08
77.41 CDCl
77.16 CDCl
76.90 CDCl
70.92
70.62
59.06
58.98
58.88
50.20
49.90
30.17
F5S
N
N
O
O
3af, 13C-NMR (126 MHz, CDCl3)
f1 (ppm)

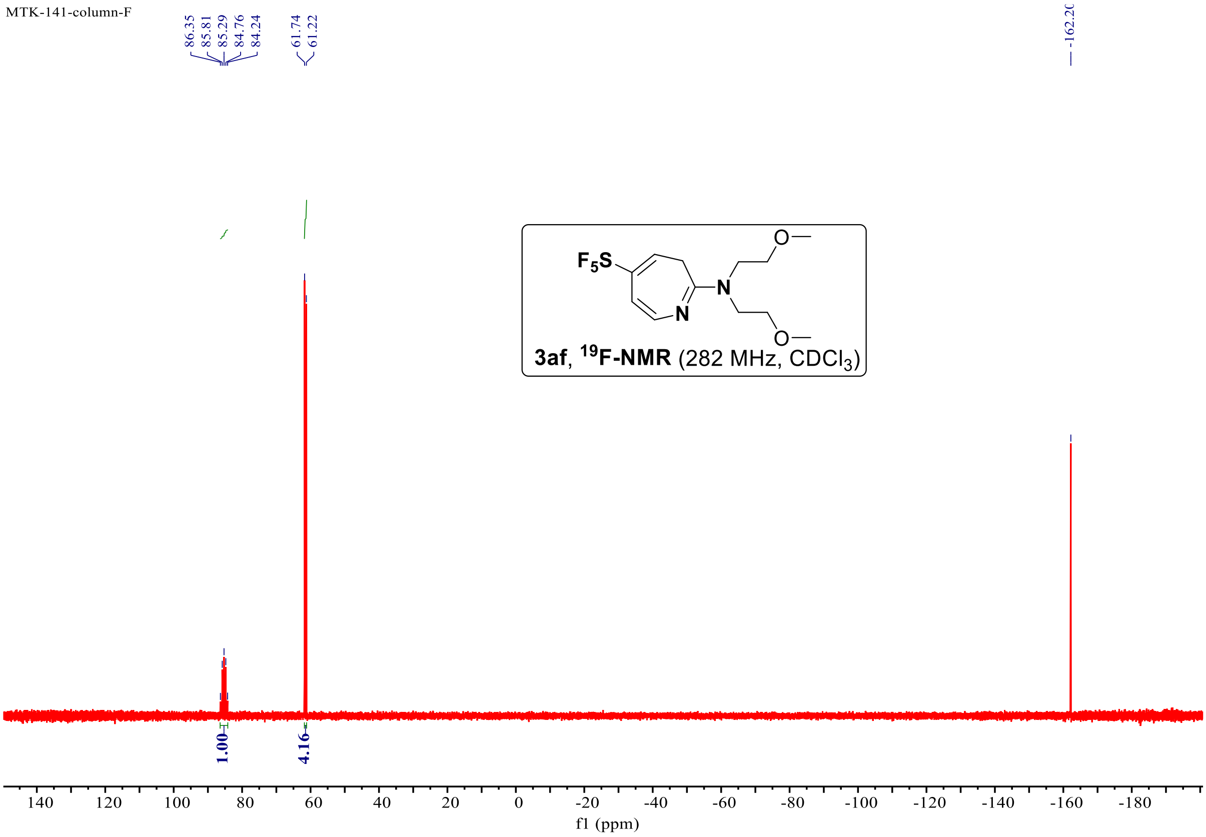
MTK-141-column-F
86.35
85.81
85.29
84.76
84.24
61.74
61.22
-162.2
F5S
N
N
O
O
3af, 19F-NMR (282 MHz, CDCl3)
1.00
4.16
f1 (ppm)

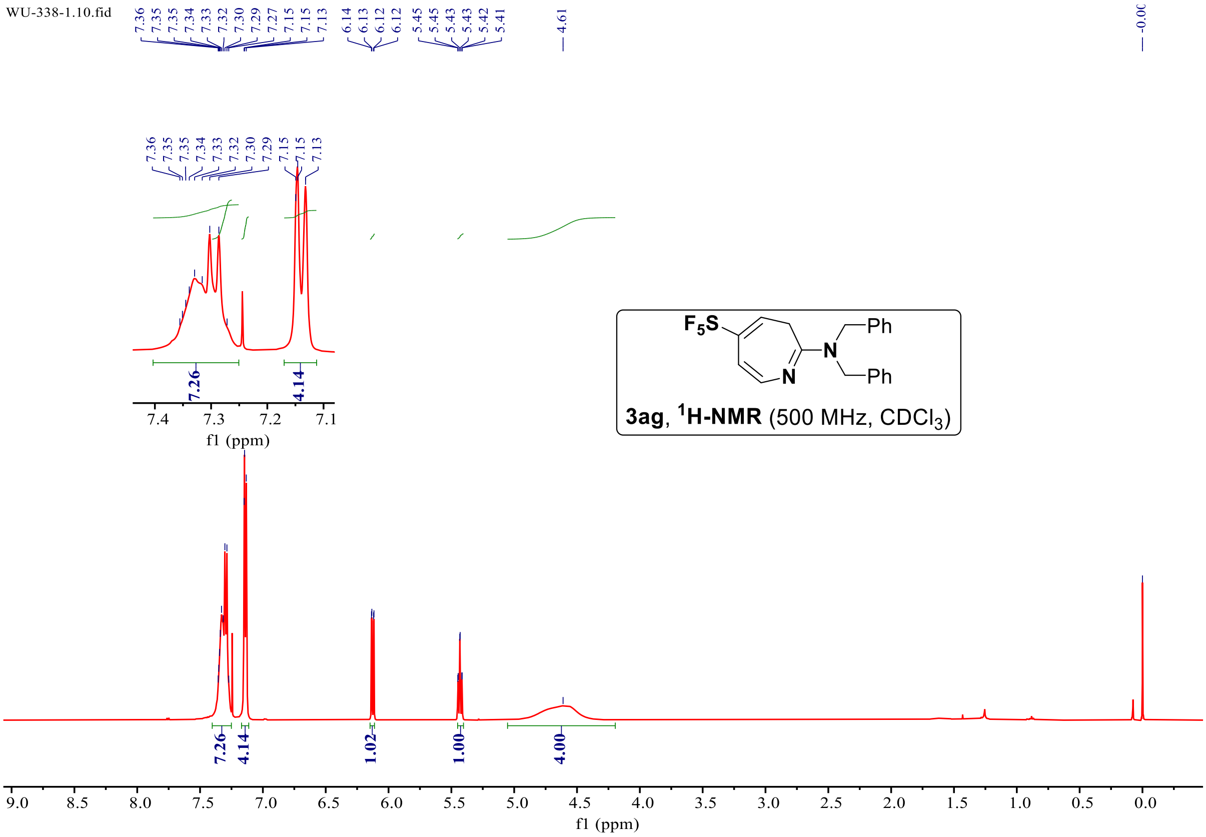
WU-338-1.10.fid
7.36 7.35 7.35 7.34 7.33 7.32 7.30 7.29 7.27 7.15 7.15 7.13 6.14 6.13 6.12 6.12 5.45 5.45 5.43 5.43 5.42 5.41 4.61 -0.00
7.26 4.14 1.02 1.00 4.00
F5S N Ph Ph
3ag, 1H-NMR (500 MHz, CDCl3)
f1 (ppm)

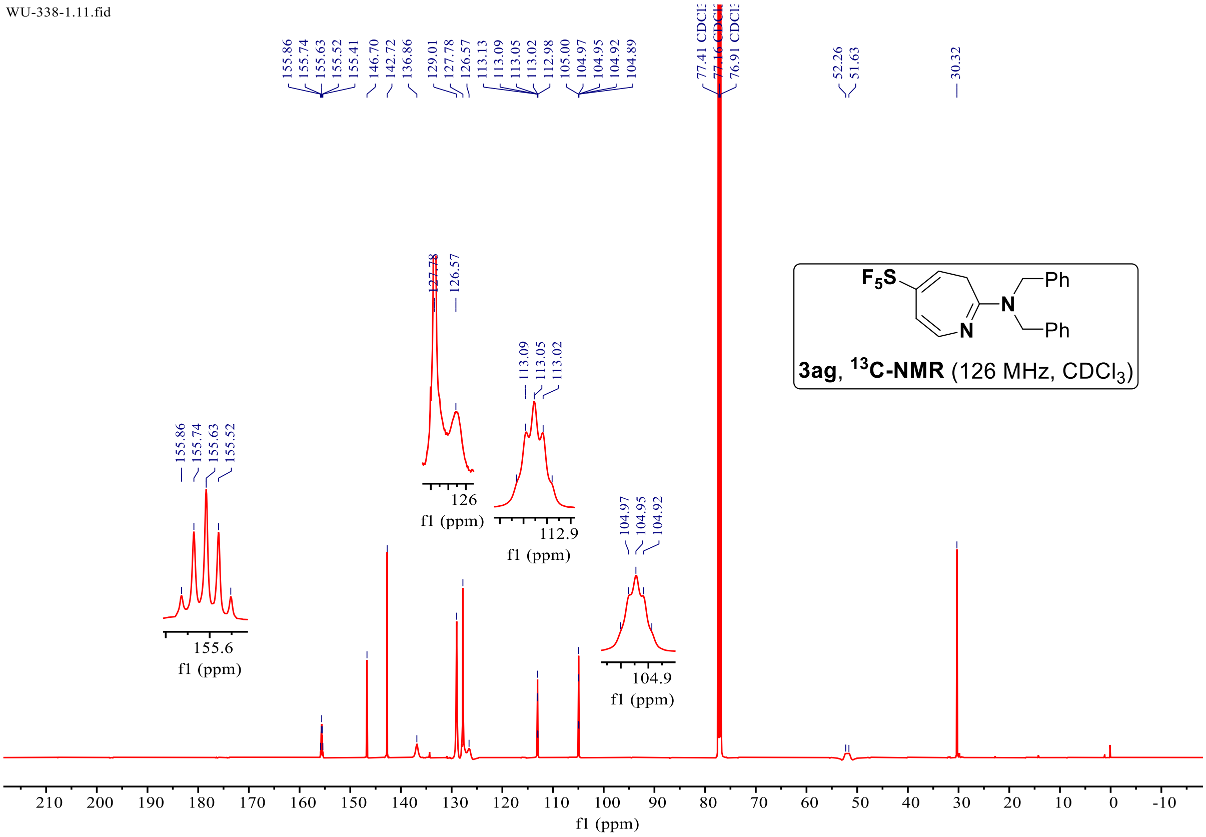
WU-338-1.11.fid
155.86 155.74 155.63 155.52 155.41 146.70 142.72 136.86 129.01 127.78 126.57 113.13 113.09 113.05 113.02 112.98 105.00 104.97 104.95 104.92 104.89 77.41 CDCl3 77.16 CDCl3 76.91 CDCl3 52.26 51.63 30.32
F5S N Ph Ph
3ag, 13C-NMR (126 MHz, CDCl3)
f1 (ppm)

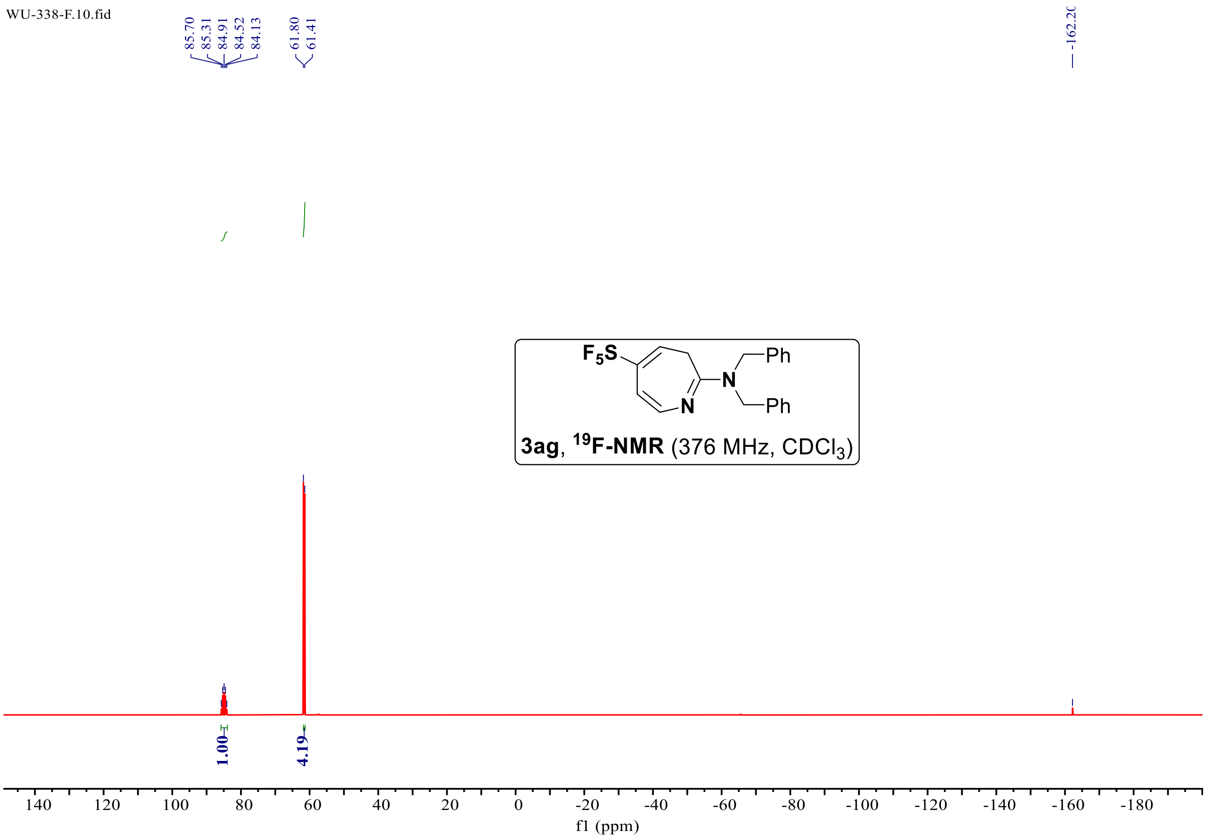

WU-338-F.10.fid
85.70
85.31
84.91
84.52
84.13
61.80
61.41
-162.20
F5S
N
N
Ph
Ph
3ag, 19F-NMR (376 MHz, CDCl3)
1.00
4.19
f1 (ppm)

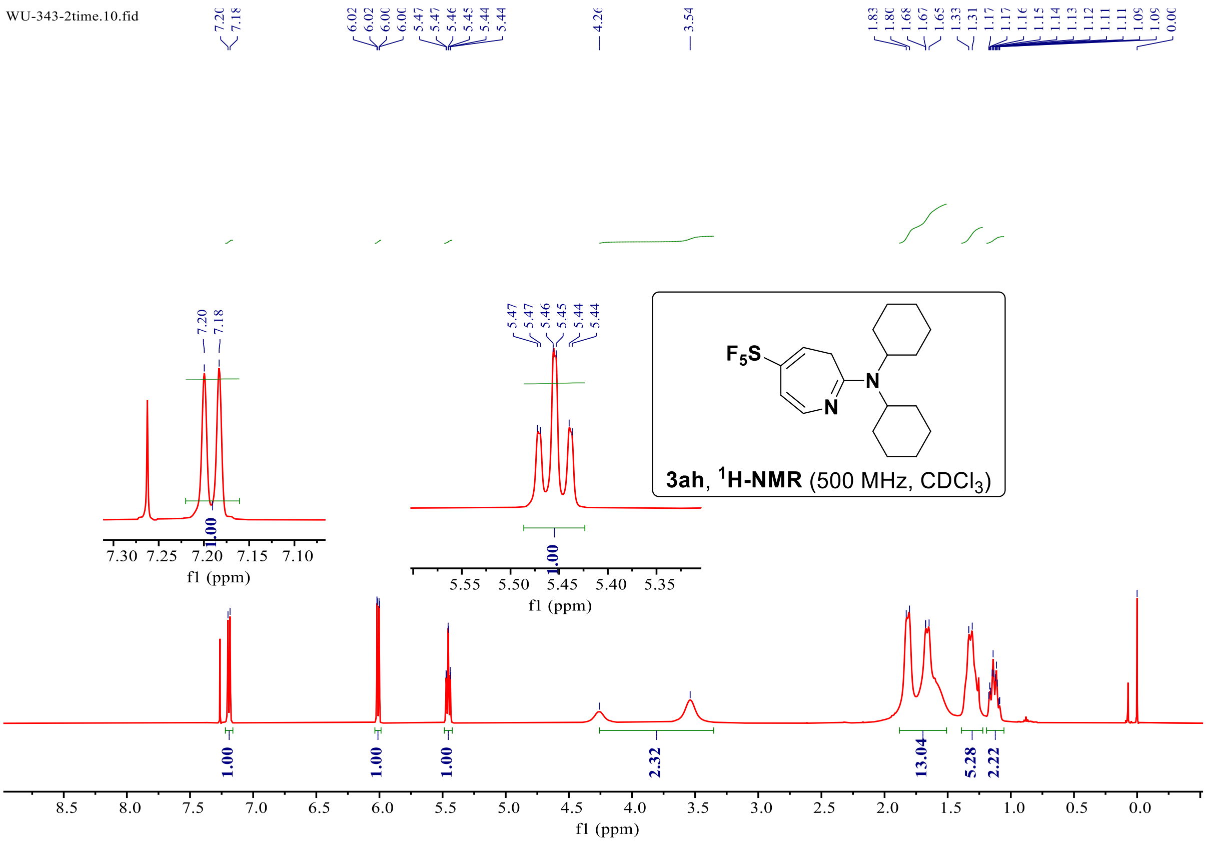

WU-343-2time.10.fid
F5S
N
N
3ah, 1H-NMR (500 MHz, CDCl3)
1.00
2.32
13.04
5.28
2.22
f1 (ppm)

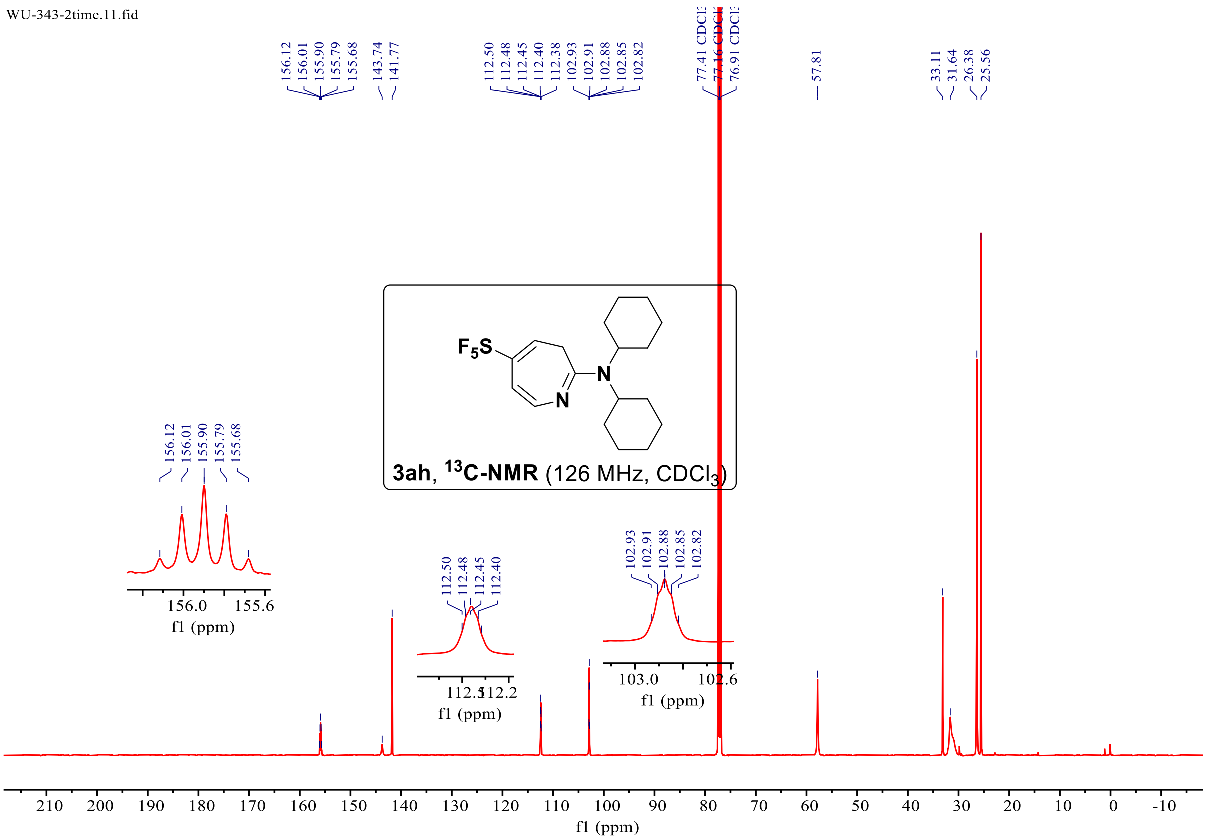

WU-343-2time.11.fid
156.12
156.01
155.90
155.79
155.68
143.74
141.77
112.50
112.48
112.45
112.40
112.38
102.93
102.91
102.88
102.85
102.82
57.81
33.11
31.64
26.38
25.56
F5S
N
N
3ah, 13C-NMR (126 MHz, CDCl3)
f1 (ppm)

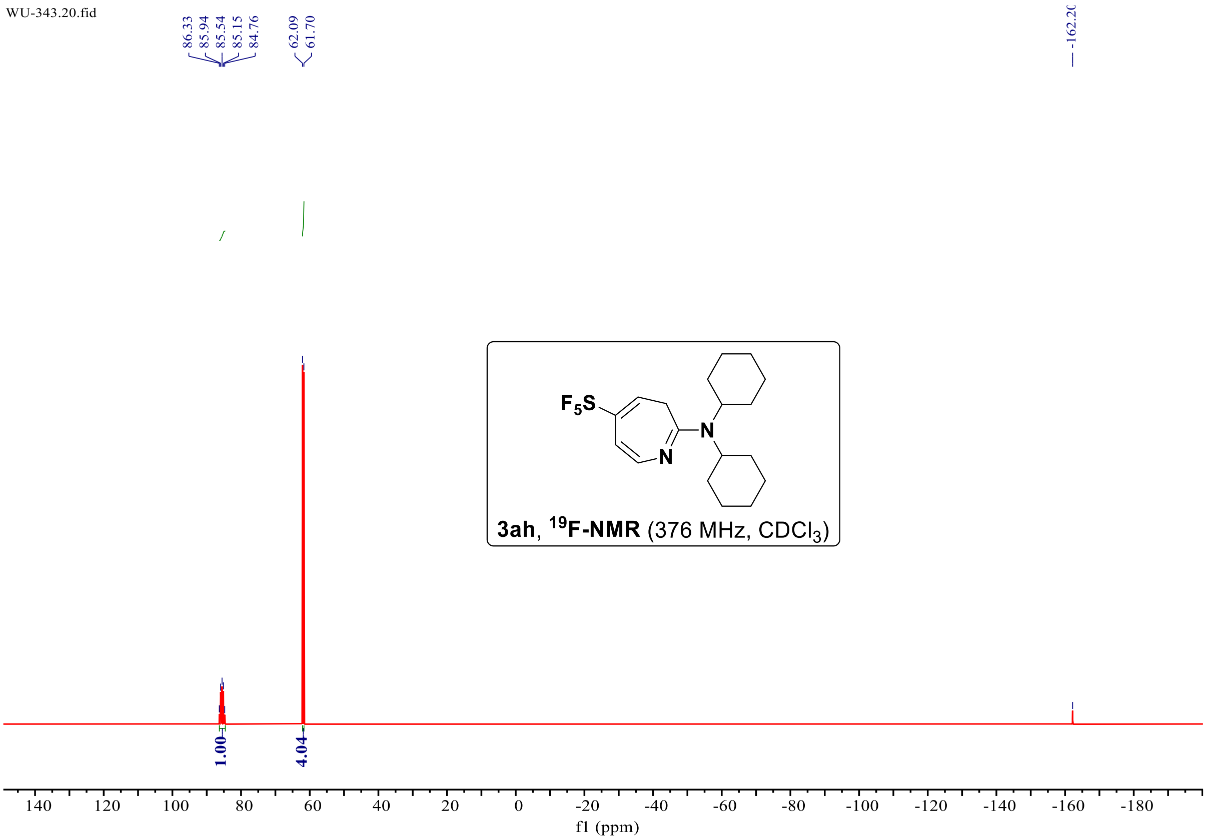

WU-343.20.fid
86.33
85.94
85.54
85.15
84.76
62.09
61.70
F5S
N
N
3ah, 19F-NMR (376 MHz, CDCl3)
1.00
4.04
f1 (ppm)

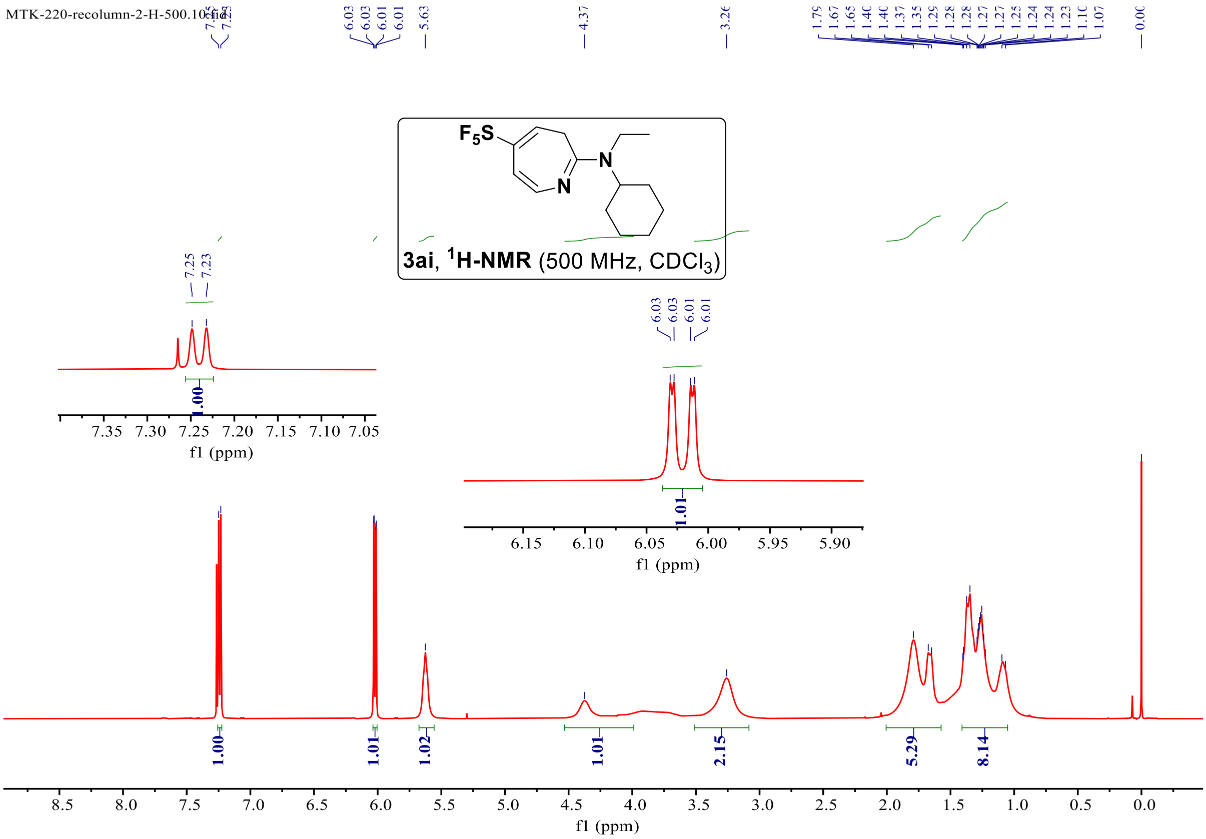
MTK-220-recolumn-2-H-500.10.fid
3ai, 1H-NMR (500 MHz, CDCl3)
F5S
N
N
7.25
7.23
6.03
6.03
6.01
6.01
1.00
1.01
1.02
1.01
2.15
5.29
8.14
f1 (ppm)

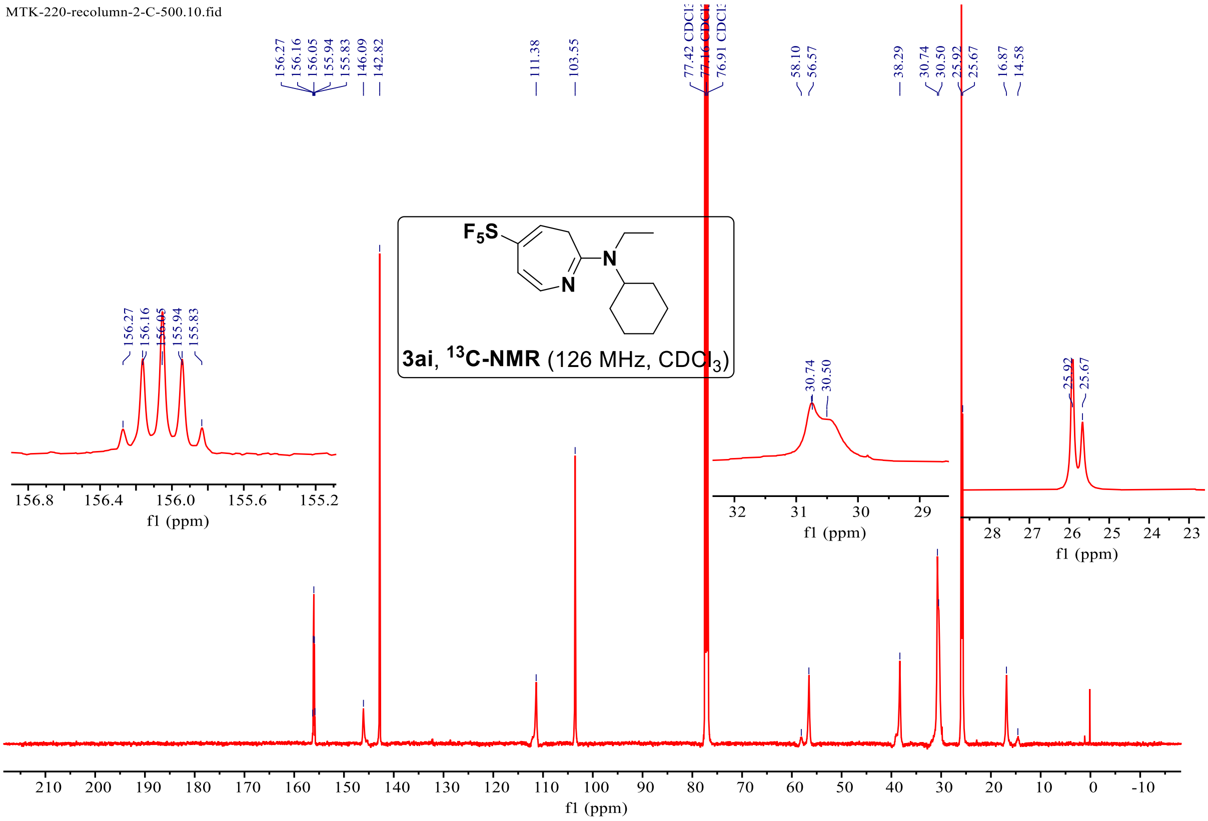
MTK-220-recolumn-2-C-500.10.fid
156.27
156.16
156.05
155.94
155.83
146.09
142.82
111.38
103.55
58.10
56.57
38.29
30.74
30.50
25.92
25.67
16.87
14.58
3ai, 13C-NMR (126 MHz, CDCl3)
F5S
N
N
f1 (ppm)

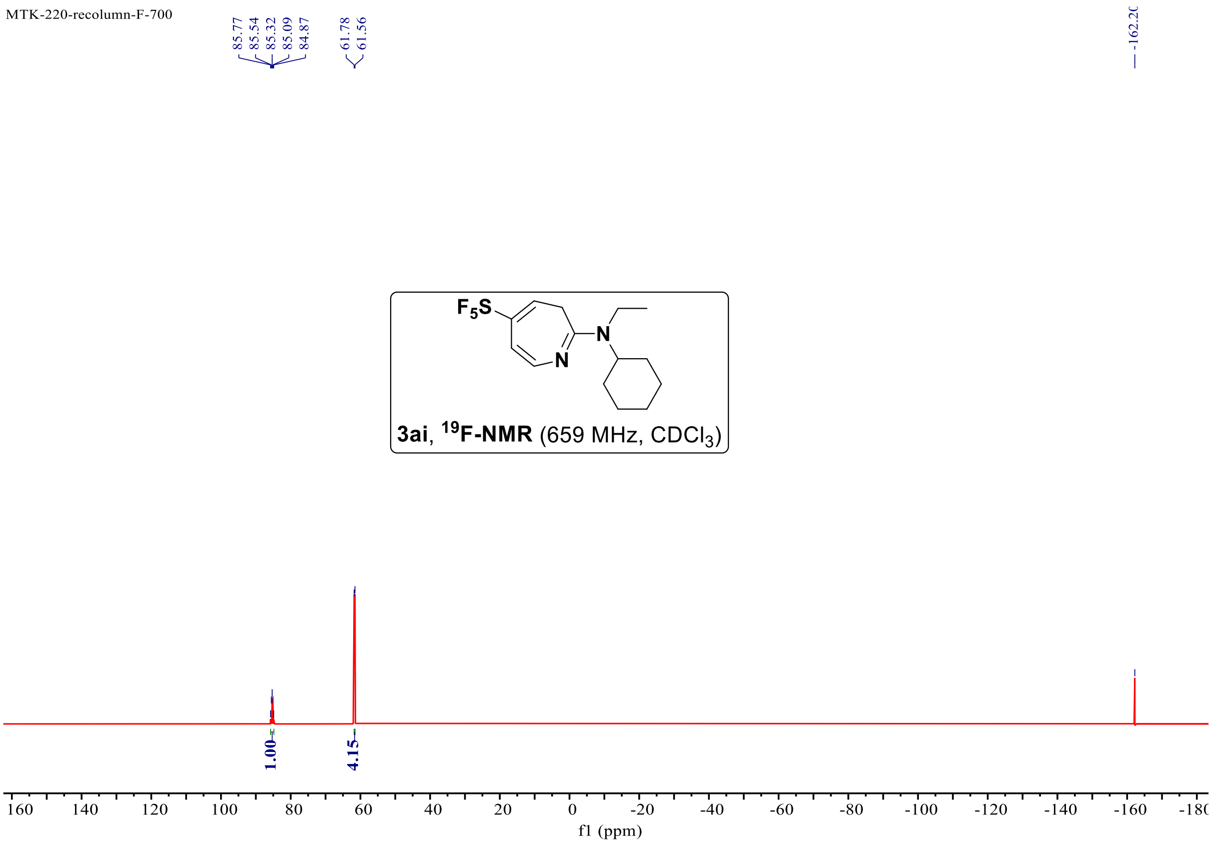
MTK-220-recolumn-F-700
85.77
85.54
85.32
85.09
84.87
61.78
61.56
-162.20
F5S
N
N
3ai, 19F-NMR (659 MHz, CDCl3)
1.00
4.15
160 140 120 100 80 60 40 20 0 -20 -40 -60 -80 -100 -120 -140 -160 -180
f1 (ppm)

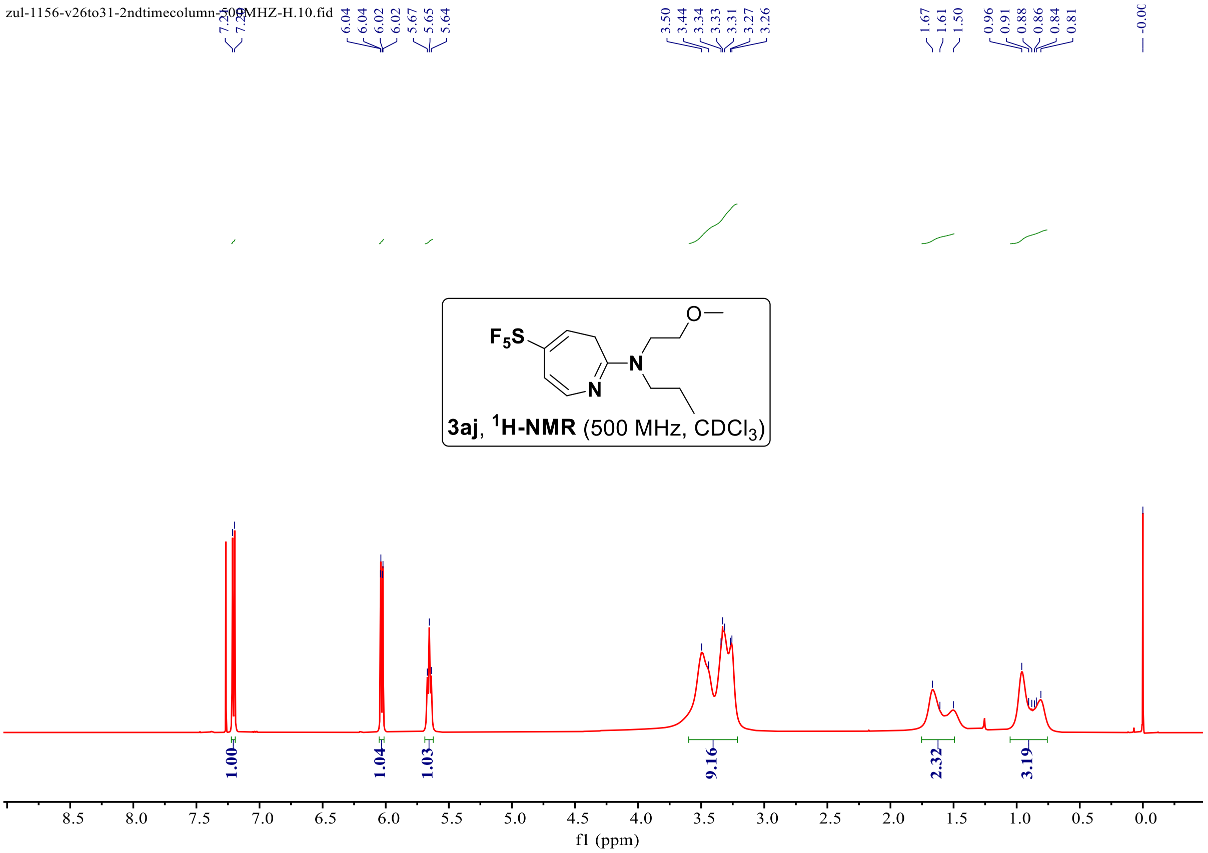
zul-1156-v26to31-2ndtimecolumn-500MHZ-H.10.fid
6.04
6.04
6.02
6.02
5.67
5.65
5.64
3.50
3.44
3.34
3.33
3.31
3.27
3.26
1.67
1.61
1.50
0.96
0.91
0.88
0.86
0.84
0.81
-0.00
F5S
O
N
N
3aj, 1H-NMR (500 MHz, CDCl3)
1.00
1.04
1.03
9.16
2.32
3.19
8.5 8.0 7.5 7.0 6.5 6.0 5.5 5.0 4.5 4.0 3.5 3.0 2.5 2.0 1.5 1.0 0.5 0.0
f1 (ppm)

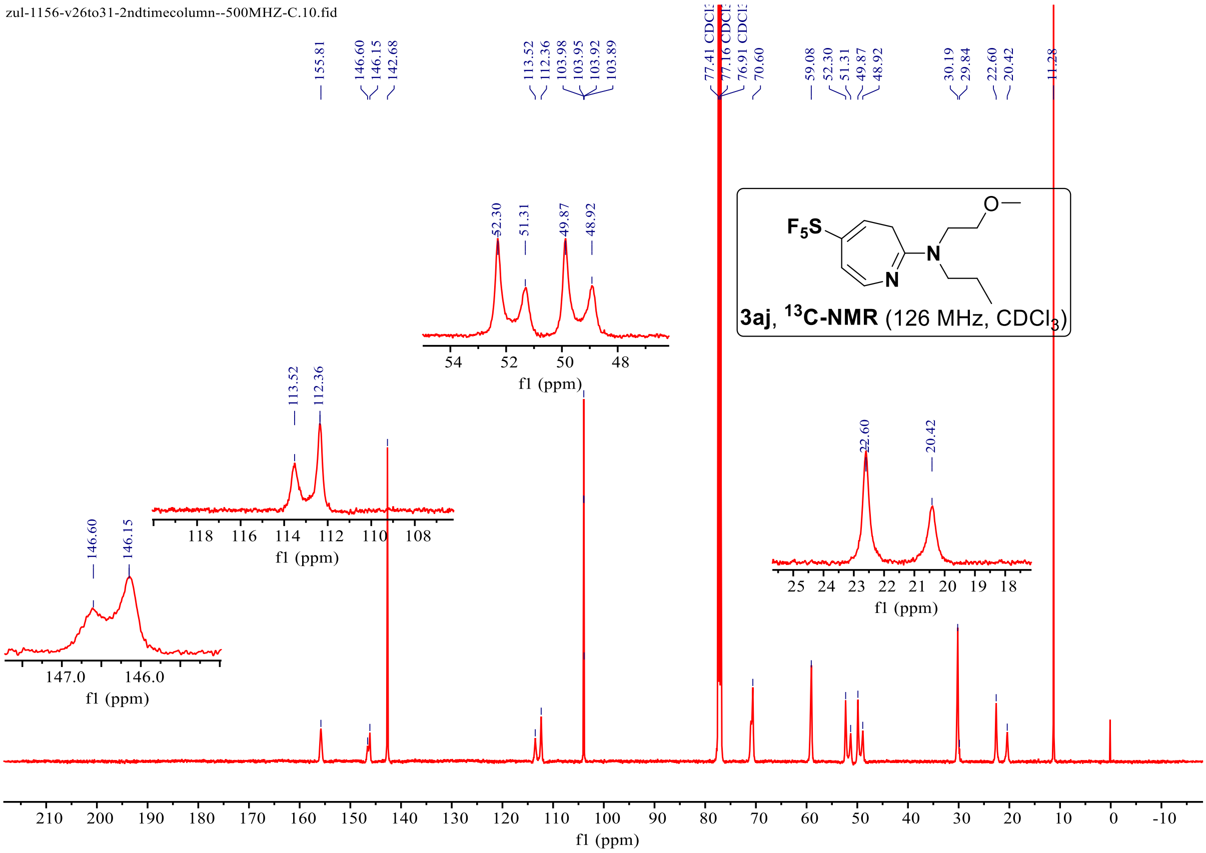
zul-1156-v26to31-2ndtimecolumn--500MHZ-C.10.fid
3aj, ¹³C-NMR (126 MHz, CDCl3)
F5S
N
O
f1 (ppm)

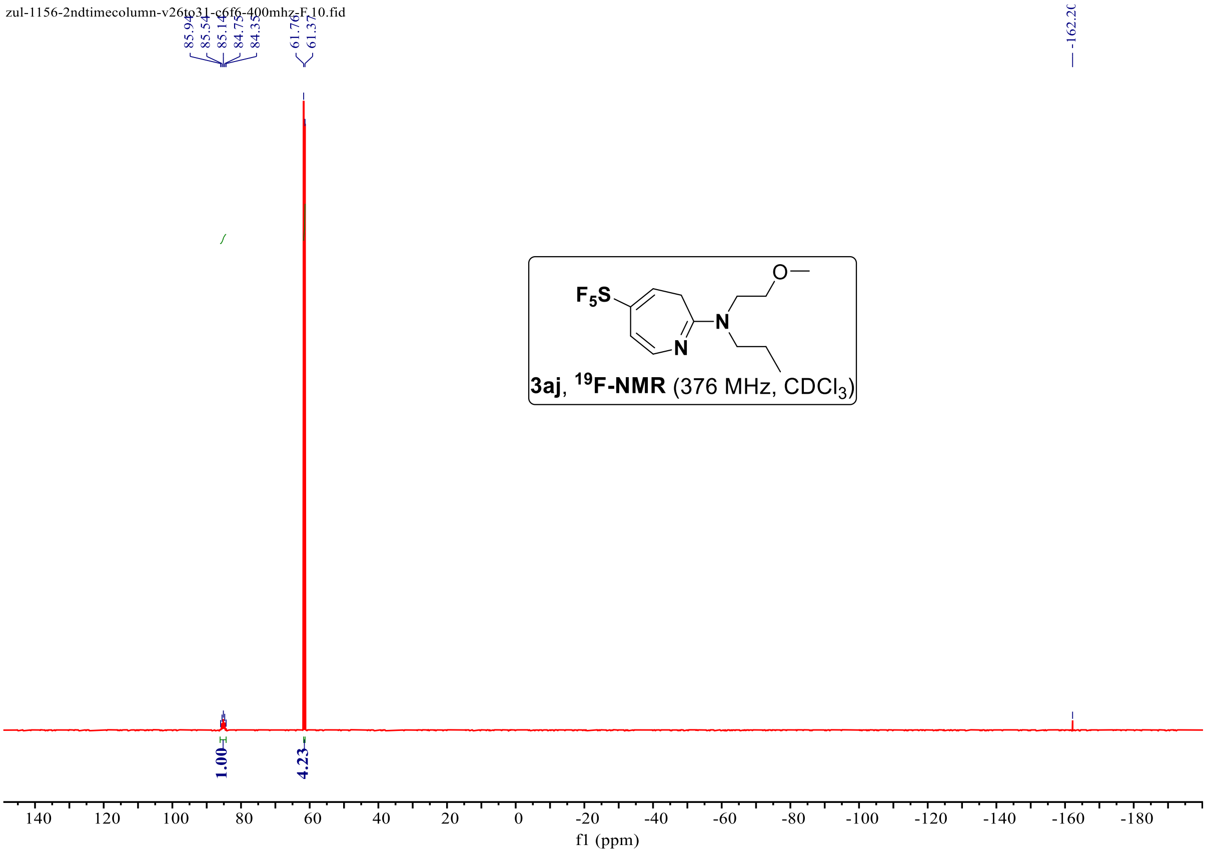
zul-1156-2ndtimecolumn-v26to31-c6f6-400mhz-F.10.fid
3aj, ¹⁹F-NMR (376 MHz, CDCl3)
F5S
N
O
1.00
4.23
f1 (ppm)

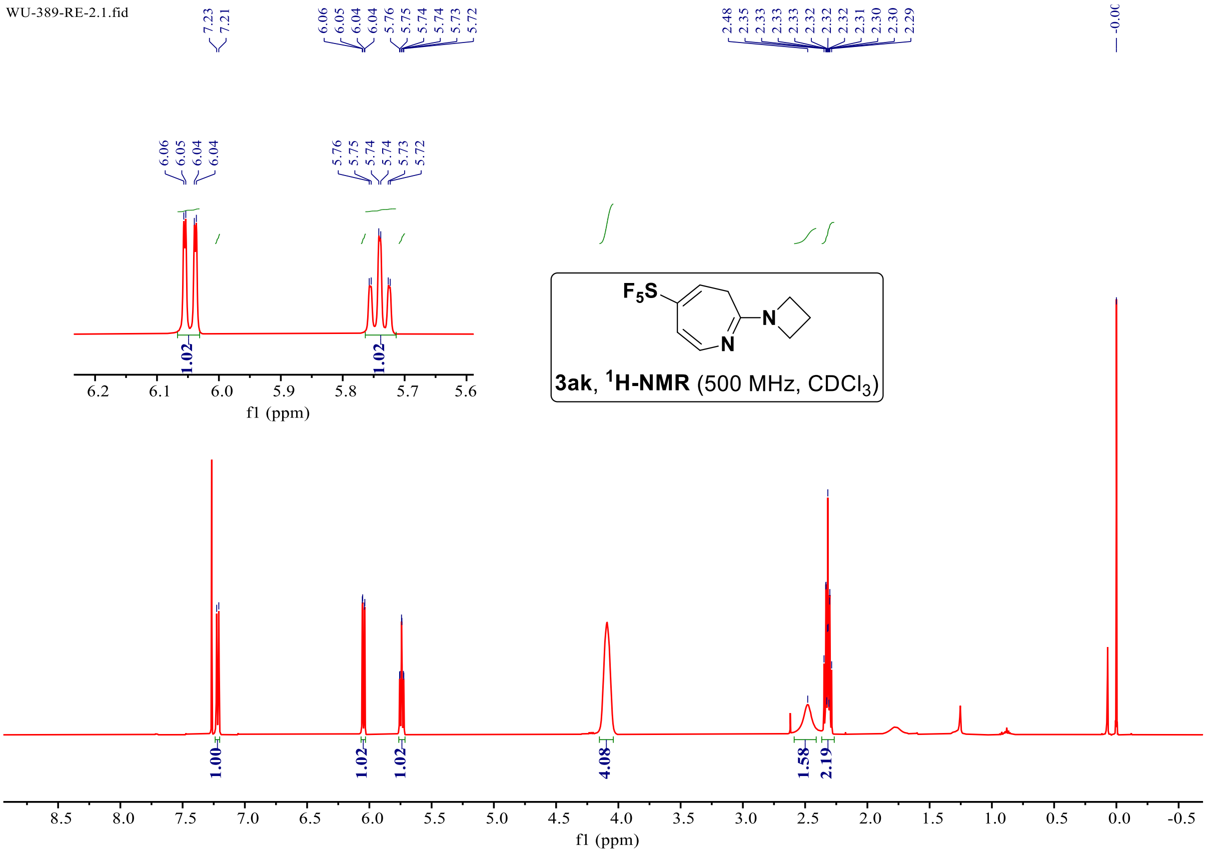
WU-389-RE-2.1.fid
7.23
7.21
6.06
6.05
6.04
6.04
5.76
5.75
5.74
5.74
5.73
5.72
2.48
2.35
2.33
2.33
2.33
2.32
2.32
2.32
2.31
2.30
2.30
2.29
-0.00
1.02
1.02
f1 (ppm)
F5S
N
N
3ak, 1H-NMR (500 MHz, CDCl3)
1.00
1.02
1.02
4.08
1.58
2.19
f1 (ppm)

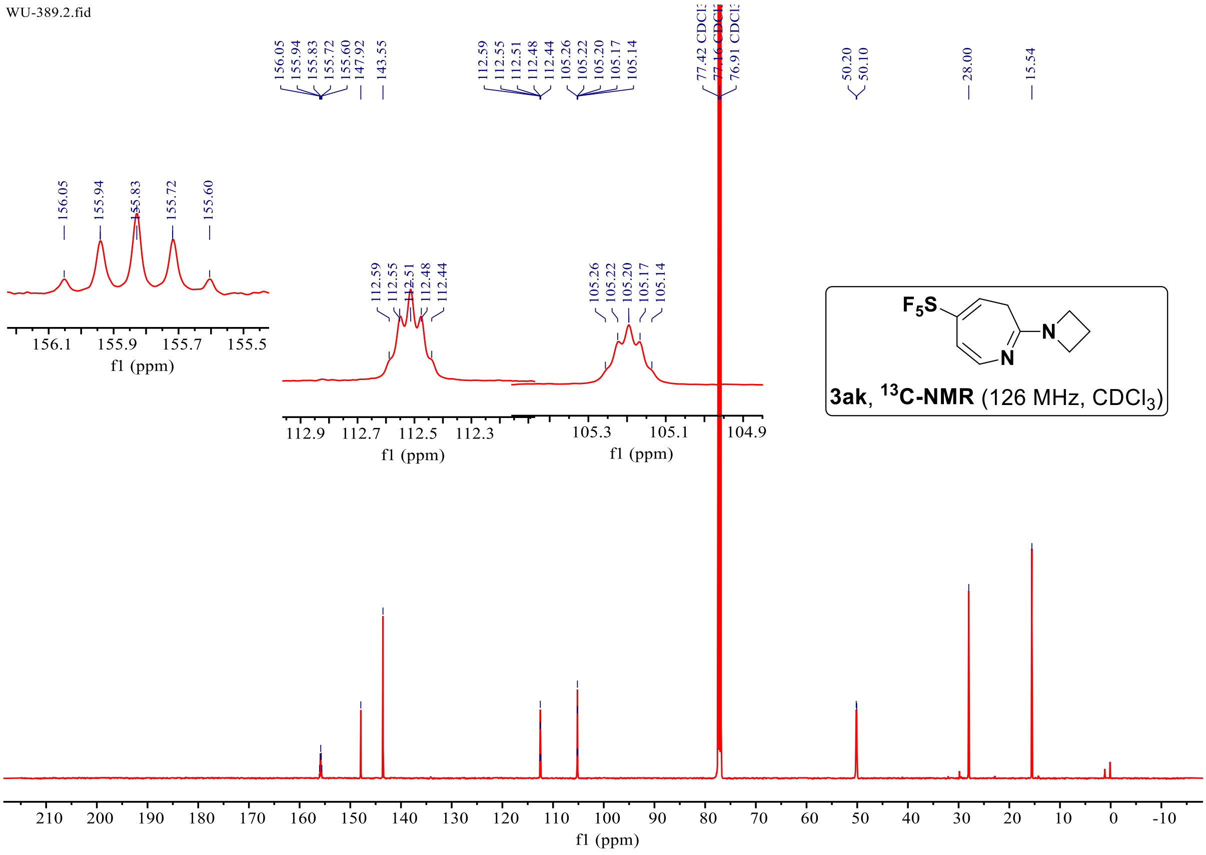
WU-389.2.fid
156.05
155.94
155.83
155.72
155.60
147.92
143.55
112.59
112.55
112.51
112.48
112.44
105.26
105.22
105.20
105.17
105.14
77.42 CDCl3
77.16 CDCl3
76.91 CDCl3
50.20
50.10
28.00
15.54
f1 (ppm)
f1 (ppm)
f1 (ppm)
F5S
N
N
3ak, 13C-NMR (126 MHz, CDCl3)
f1 (ppm)

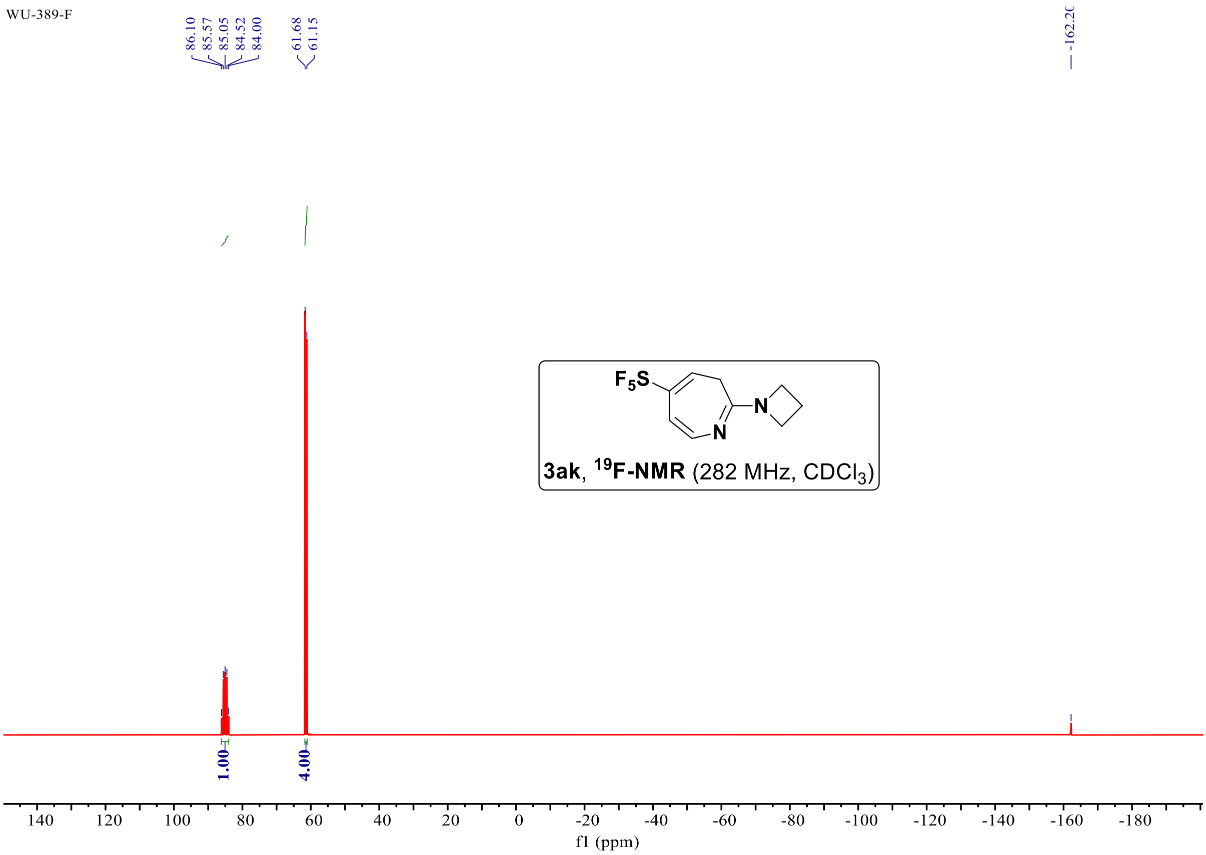
WU-389-F
86.10
85.57
85.05
84.52
84.00
61.68
61.15
-162.20
F5S
N
N
3ak, 19F-NMR (282 MHz, CDCl3)
1.00
4.00
f1 (ppm)

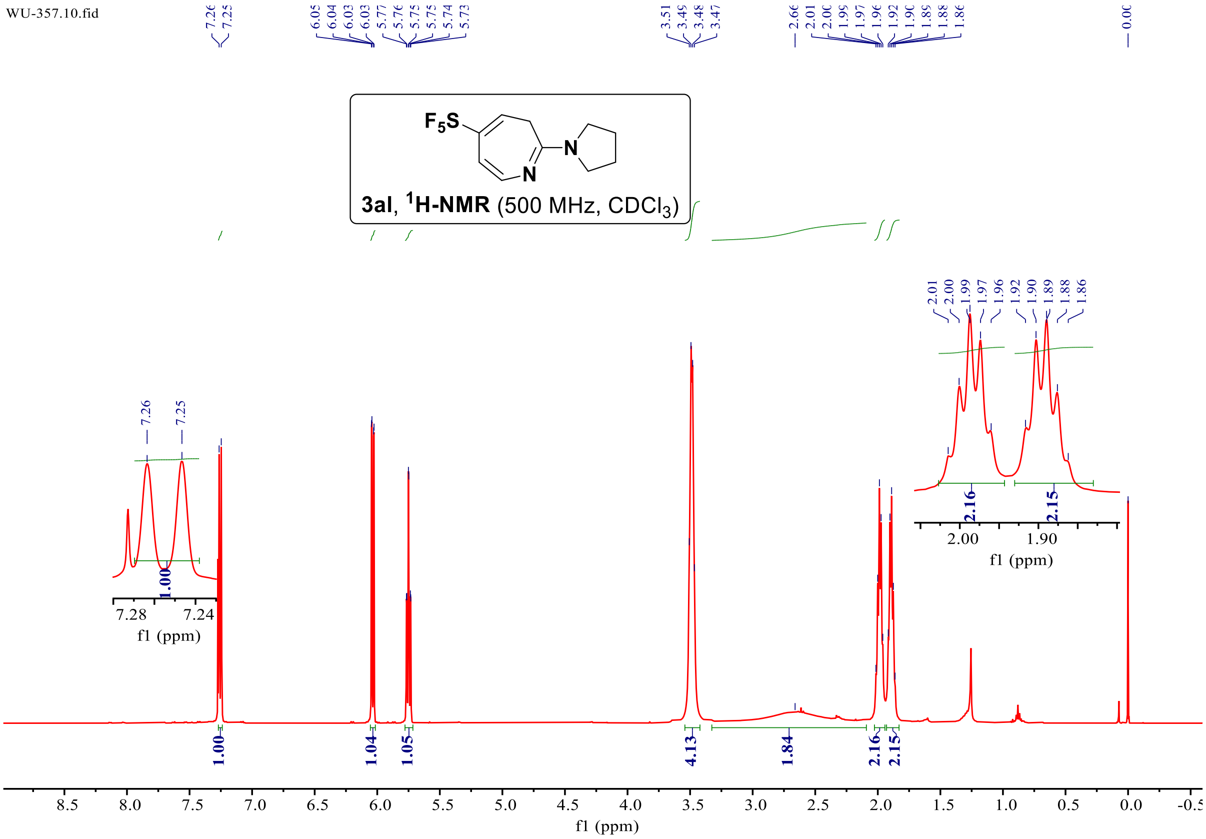
WU-357.10.fid
F5S
N
N
3al, 1H-NMR (500 MHz, CDCl3)
7.26
7.25
1.00
1.04
1.05
4.13
1.84
2.16
2.15
f1 (ppm)

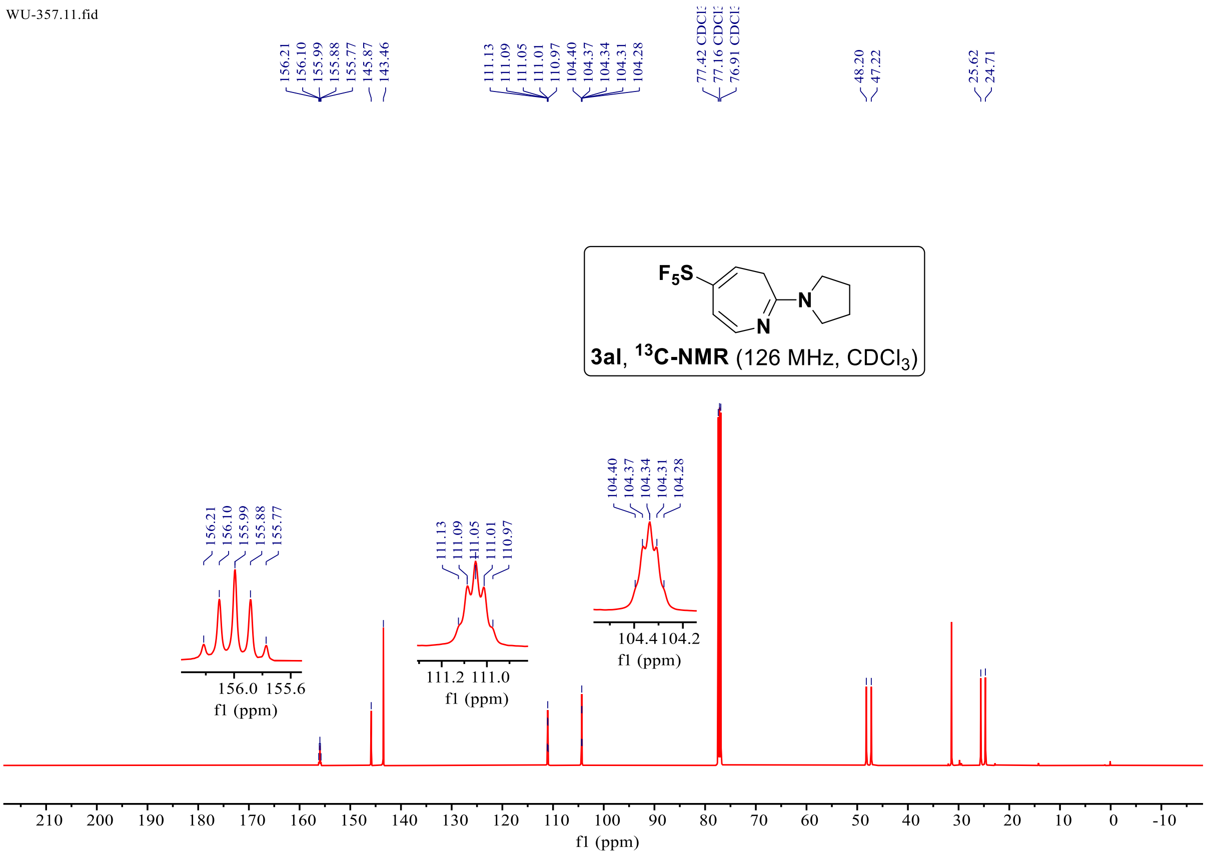
WU-357.11.fid
156.21
156.10
155.99
155.88
155.77
145.87
143.46
111.13
111.09
111.05
111.01
110.97
104.40
104.37
104.34
104.31
104.28
77.42 CDCl3
77.16 CDCl3
76.91 CDCl3
48.20
47.22
25.62
24.71
F5S
N
N
3al, 13C-NMR (126 MHz, CDCl3)
f1 (ppm)

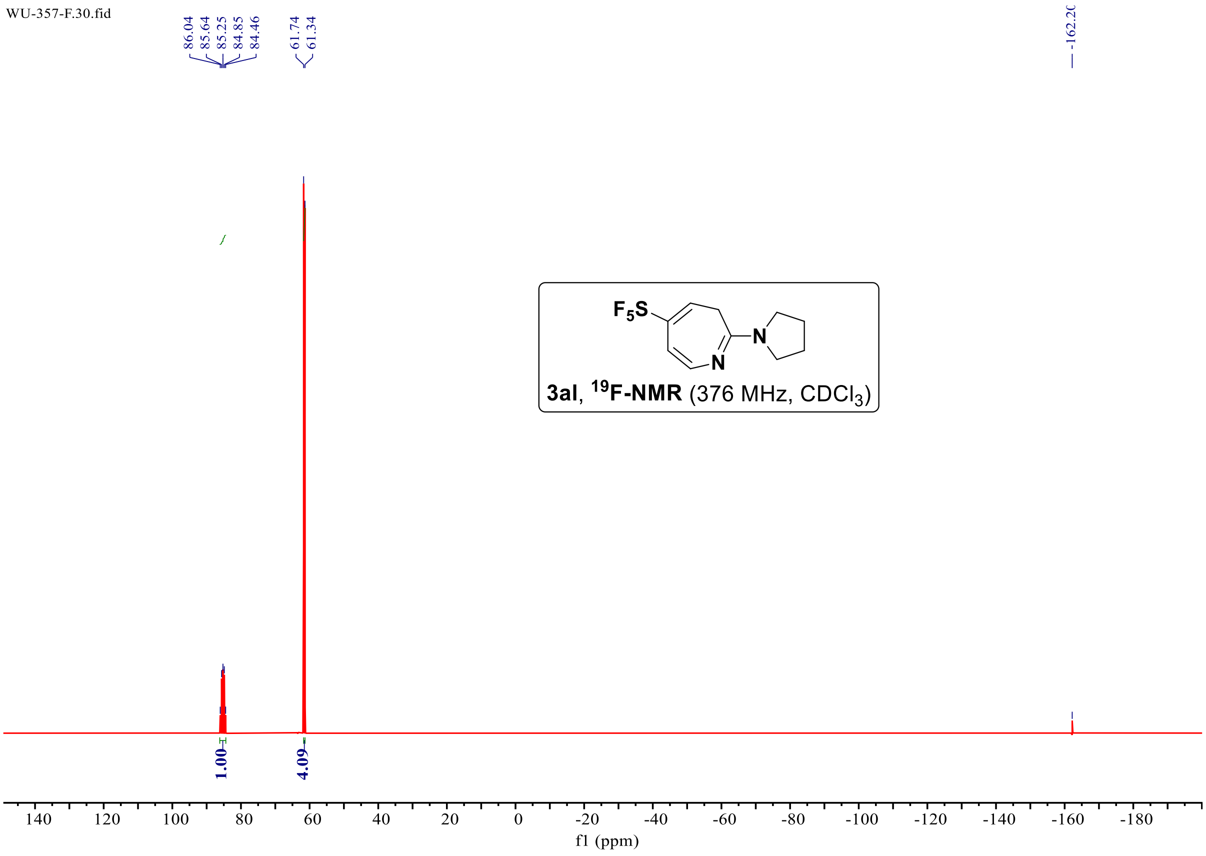
WU-357-F.30.fid
86.04
85.64
85.25
84.85
84.46
61.74
61.34
-162.20
F5S
N
N
3al, 19F-NMR (376 MHz, CDCl3)
1.00
4.09
f1 (ppm)

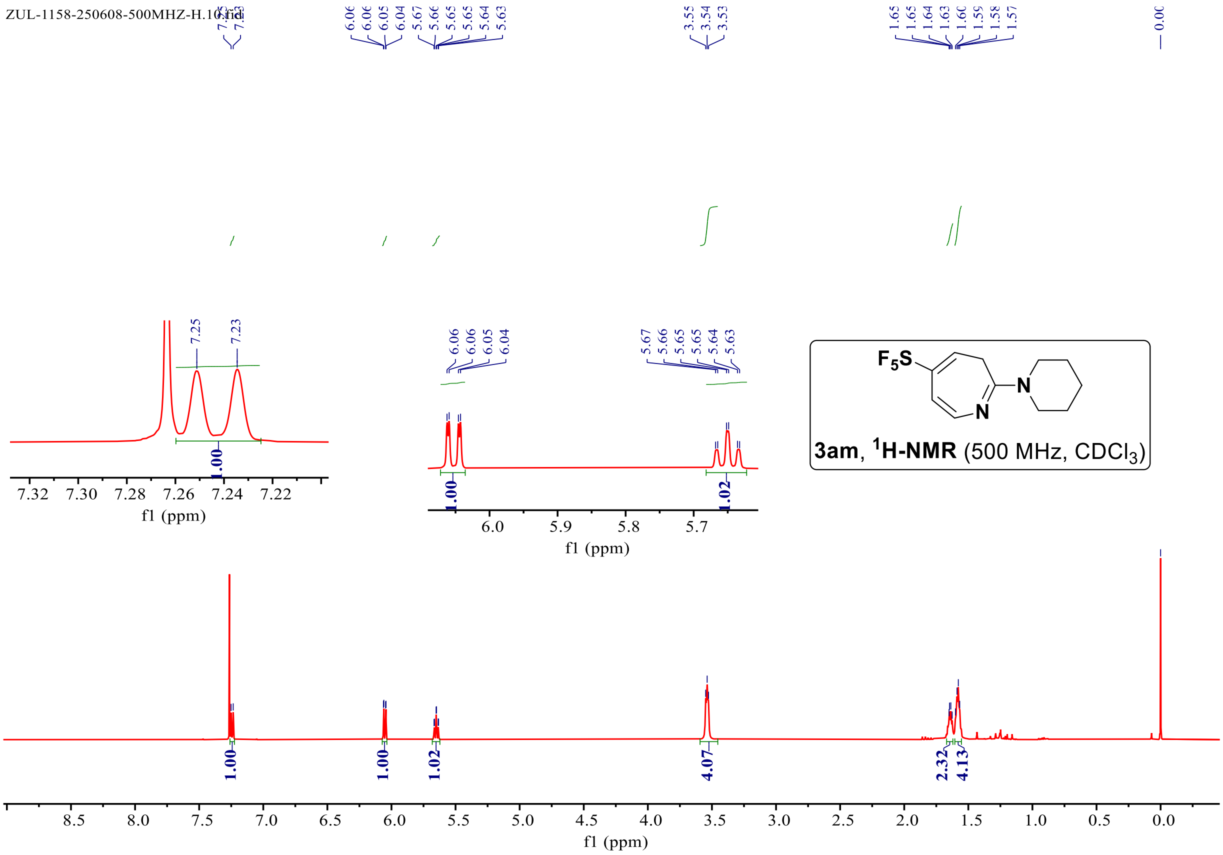
ZUL-1158-250608-500MHZ-H.10.fid
3am, ¹H-NMR (500 MHz, CDCl3)
F5S
N
1.00
1.00
1.02
4.07
2.32
4.13
f1 (ppm)

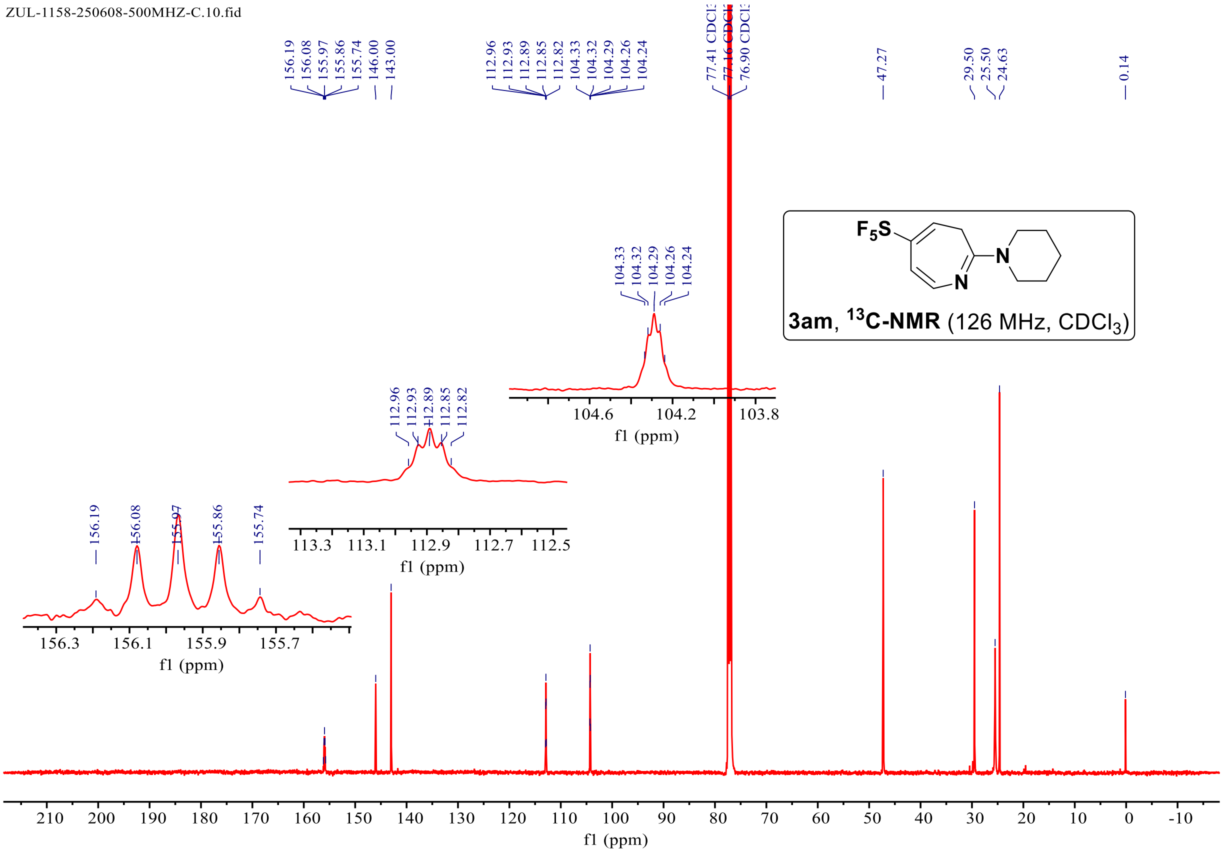
ZUL-1158-250608-500MHZ-C.10.fid
156.19
156.08
155.97
155.86
155.74
146.00
143.00
112.96
112.93
112.89
112.85
112.82
104.33
104.32
104.29
104.26
104.24
47.27
29.50
25.50
24.63
0.14
3am, ¹³C-NMR (126 MHz, CDCl3)
F5S
N
f1 (ppm)

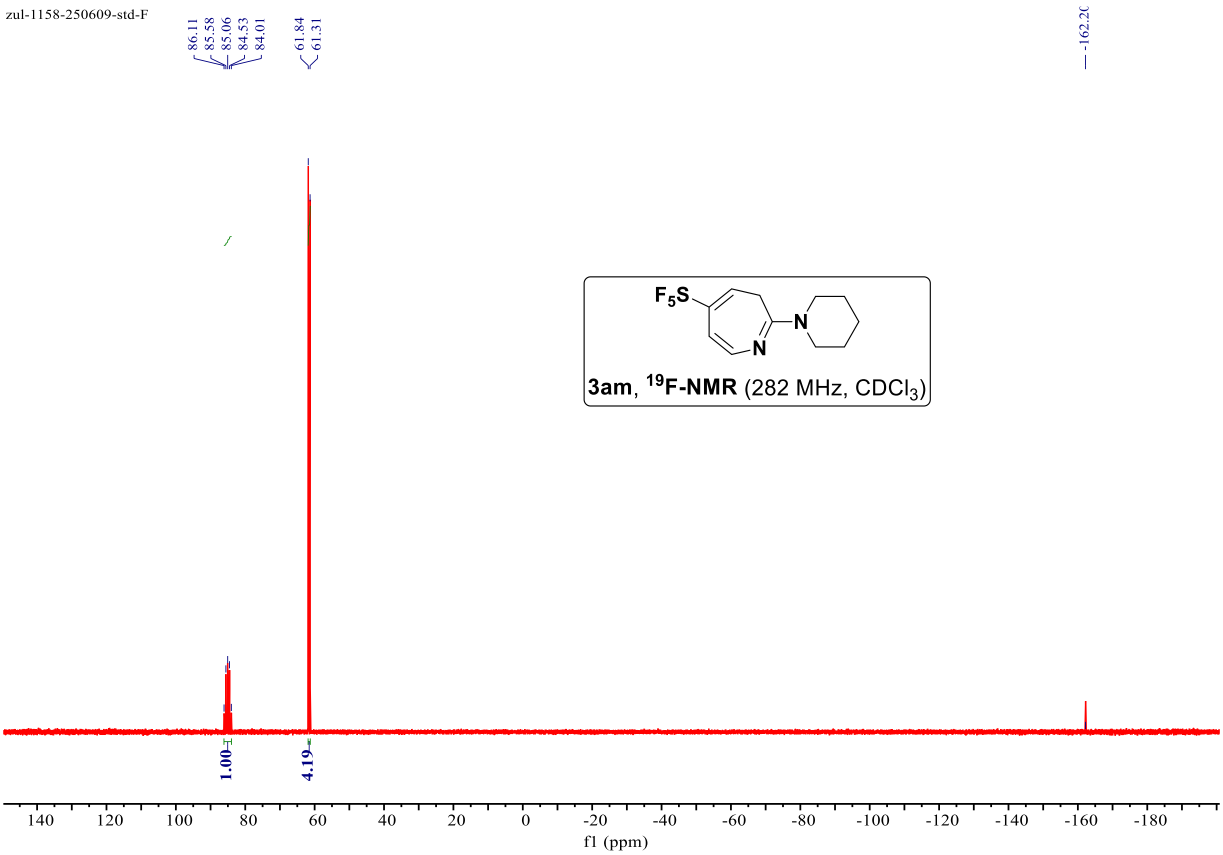
zul-1158-250609-std-F
3am, ^{19}F-NMR (282 MHz, CDCl$_3$)
f1 (ppm)

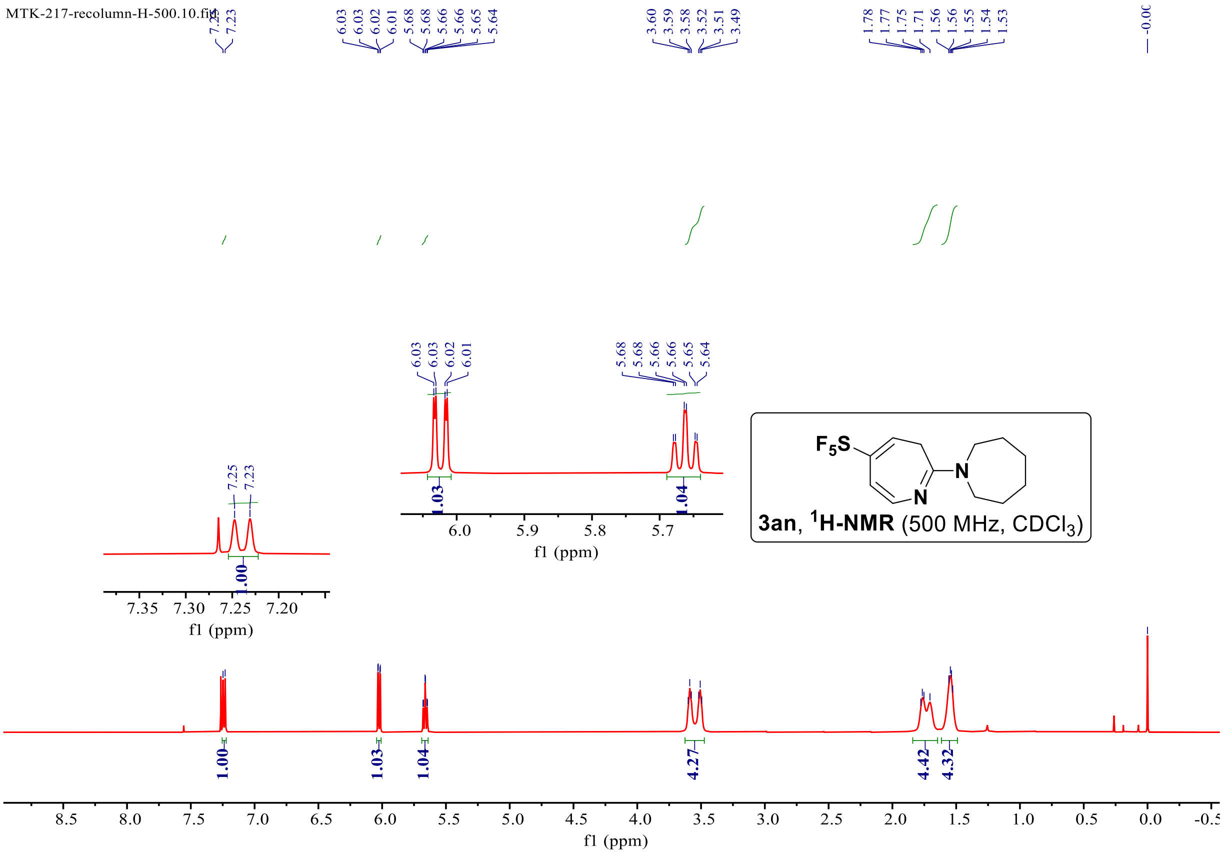
MTK-217-recolumn-H-500.10.fid
3an, ^{1}H-NMR (500 MHz, CDCl$_3$)
f1 (ppm)

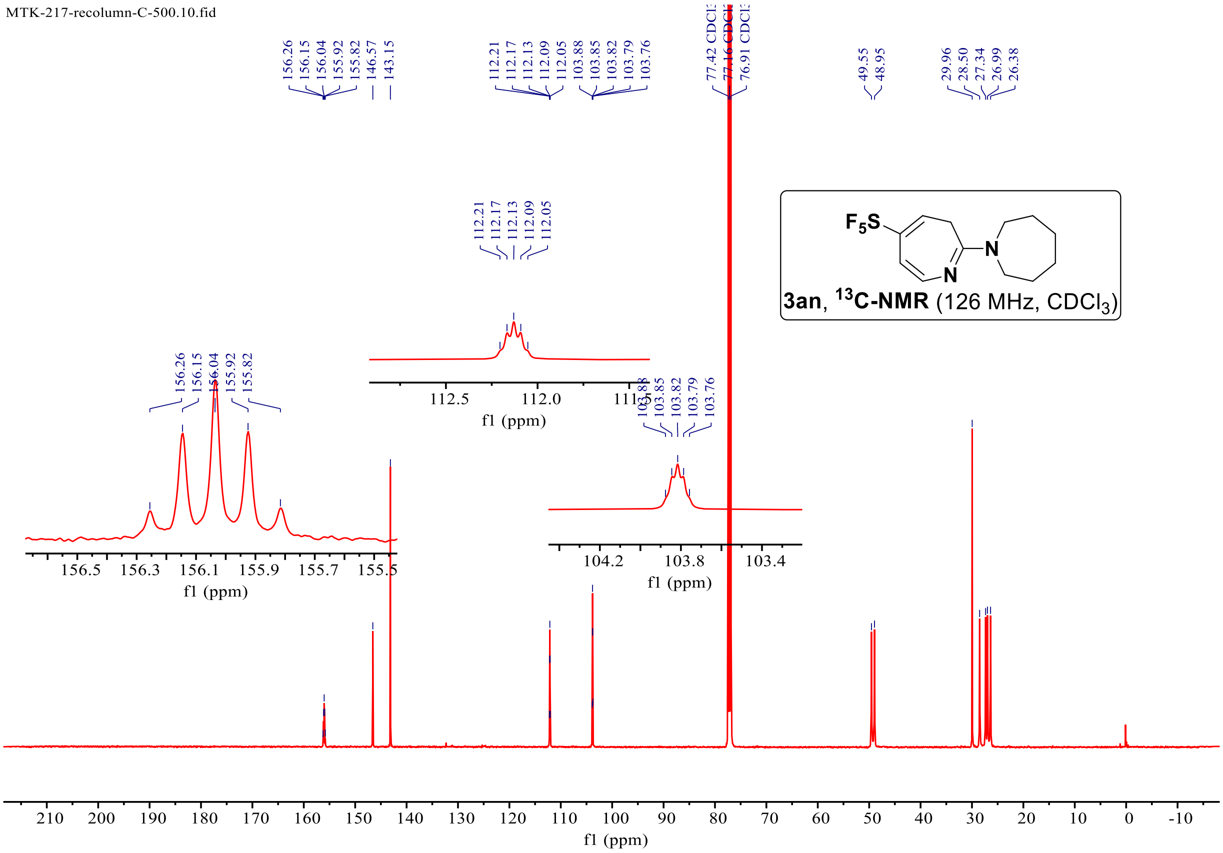
MTK-217-recolumn-C-500.10.fid
156.26
156.15
156.04
155.92
155.82
146.57
143.15
112.21
112.17
112.13
112.09
112.05
103.88
103.85
103.82
103.79
103.76
77.42 CDCl3
76.91 CDCl3
49.55
48.95
29.96
28.50
27.34
26.99
26.38
F5S
N
3an, 13C-NMR (126 MHz, CDCl3)
f1 (ppm)

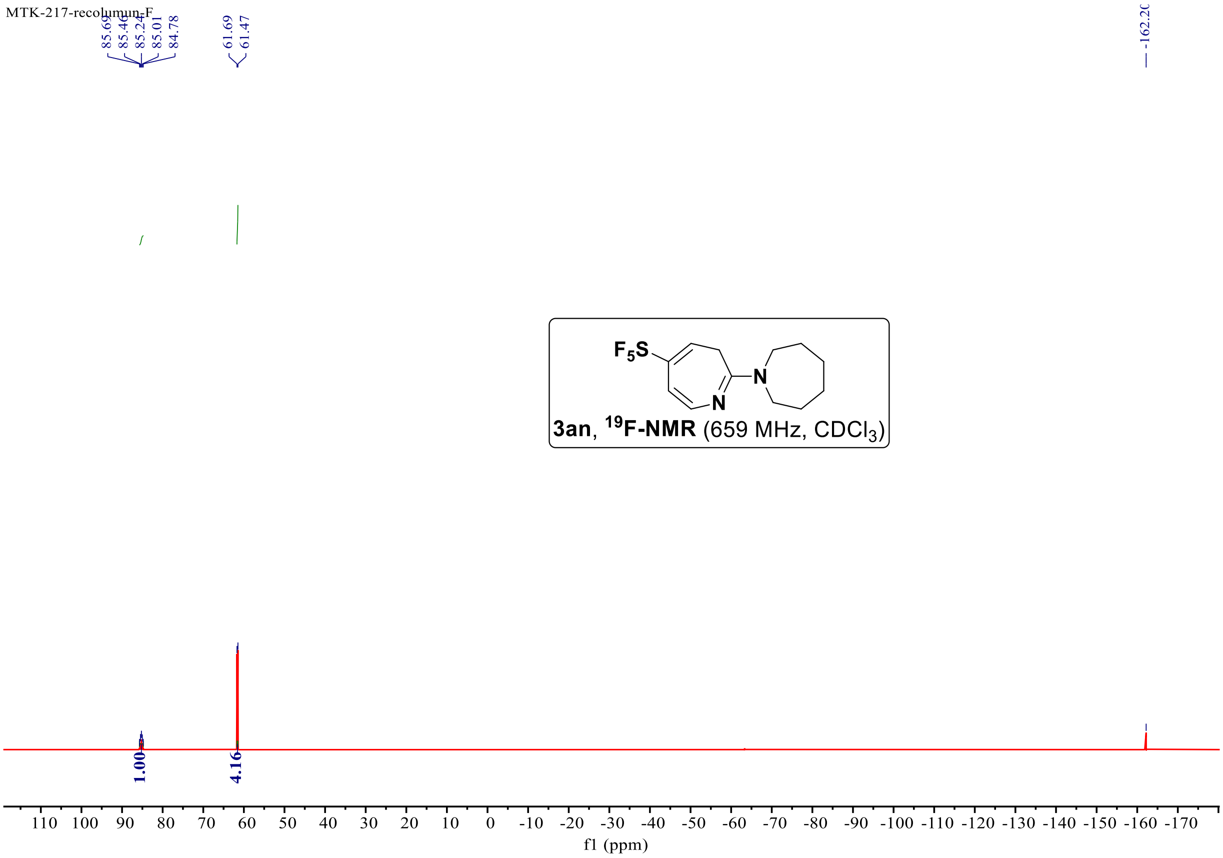
85.01
84.78
61.69
61.47
F5S
N
3an, 19F-NMR (659 MHz, CDCl3)
1.00
4.16
f1 (ppm)

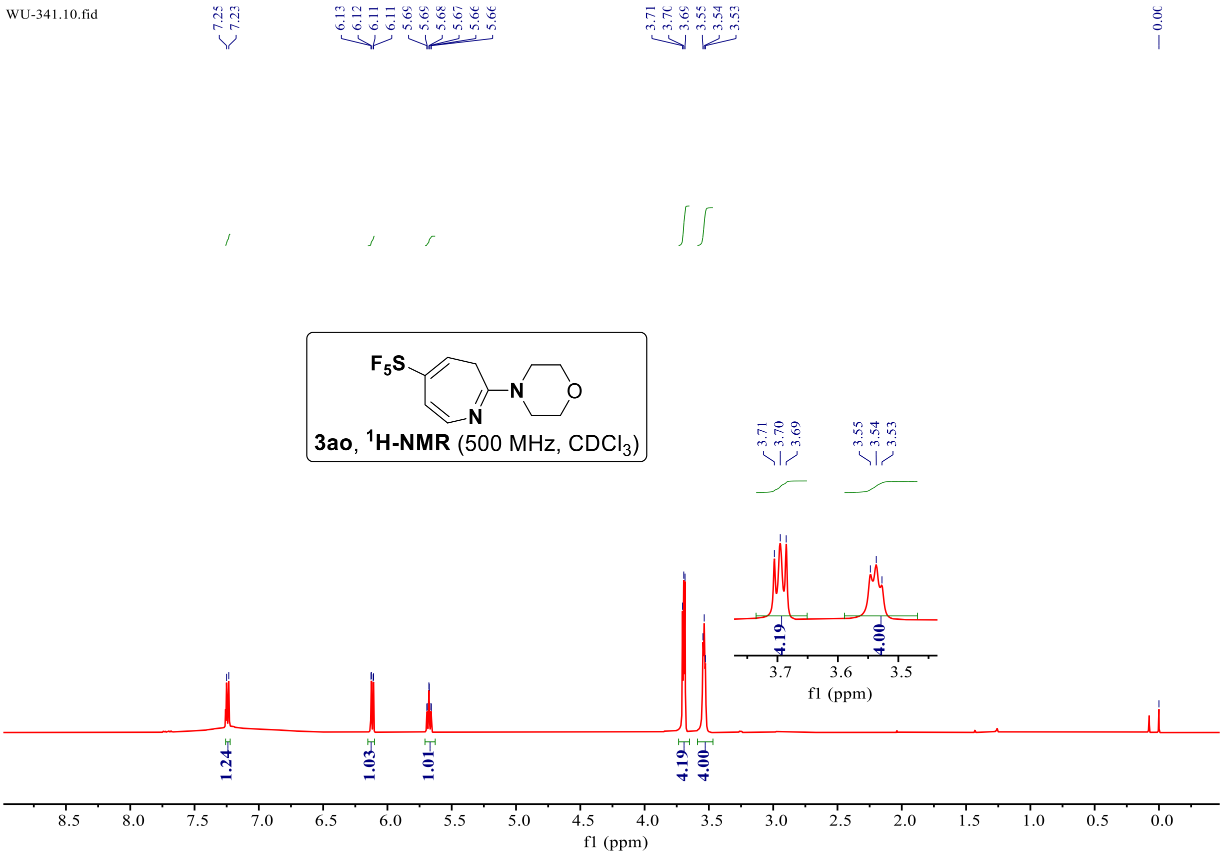
WU-341.10.fid
3ao, ¹H-NMR (500 MHz, CDCl₃)
f1 (ppm)

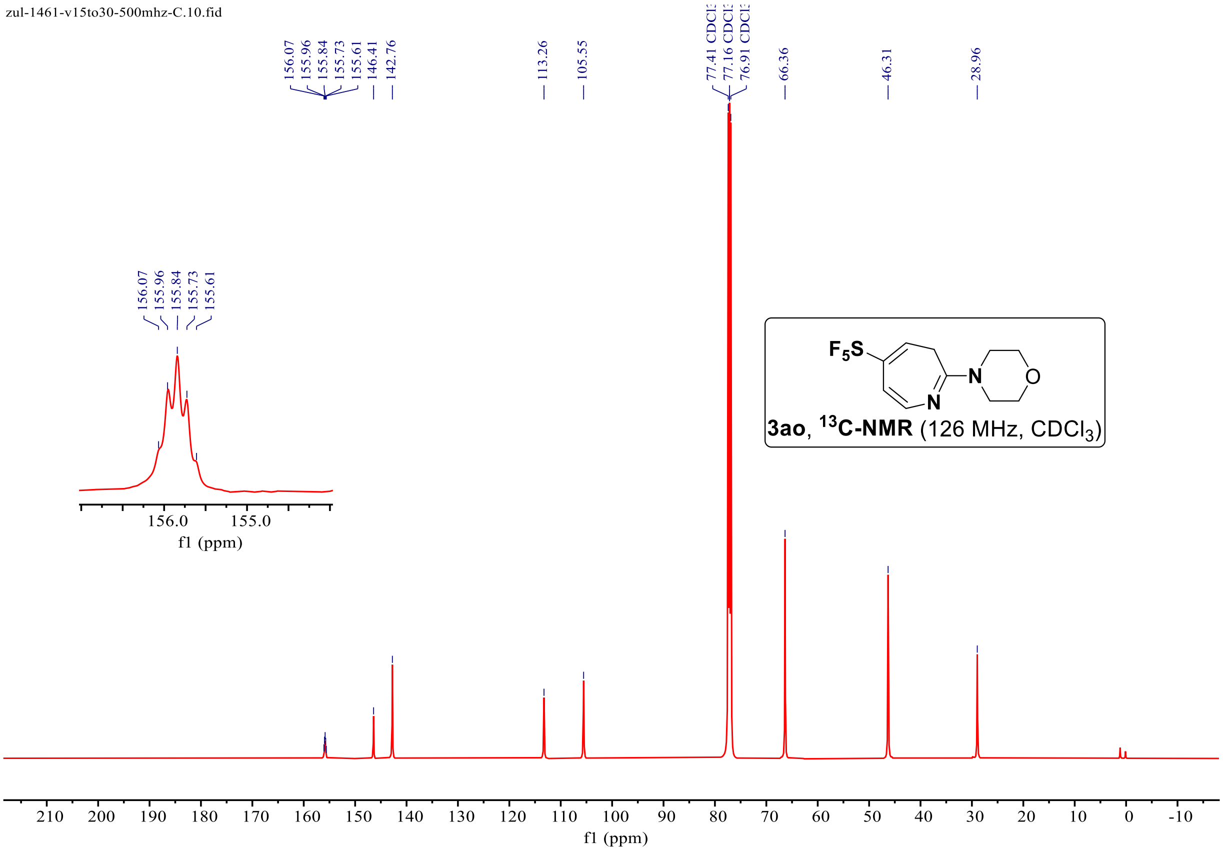
zul-1461-v15to30-500mhz-C.10.fid
3ao, ¹³C-NMR (126 MHz, CDCl₃)
f1 (ppm)

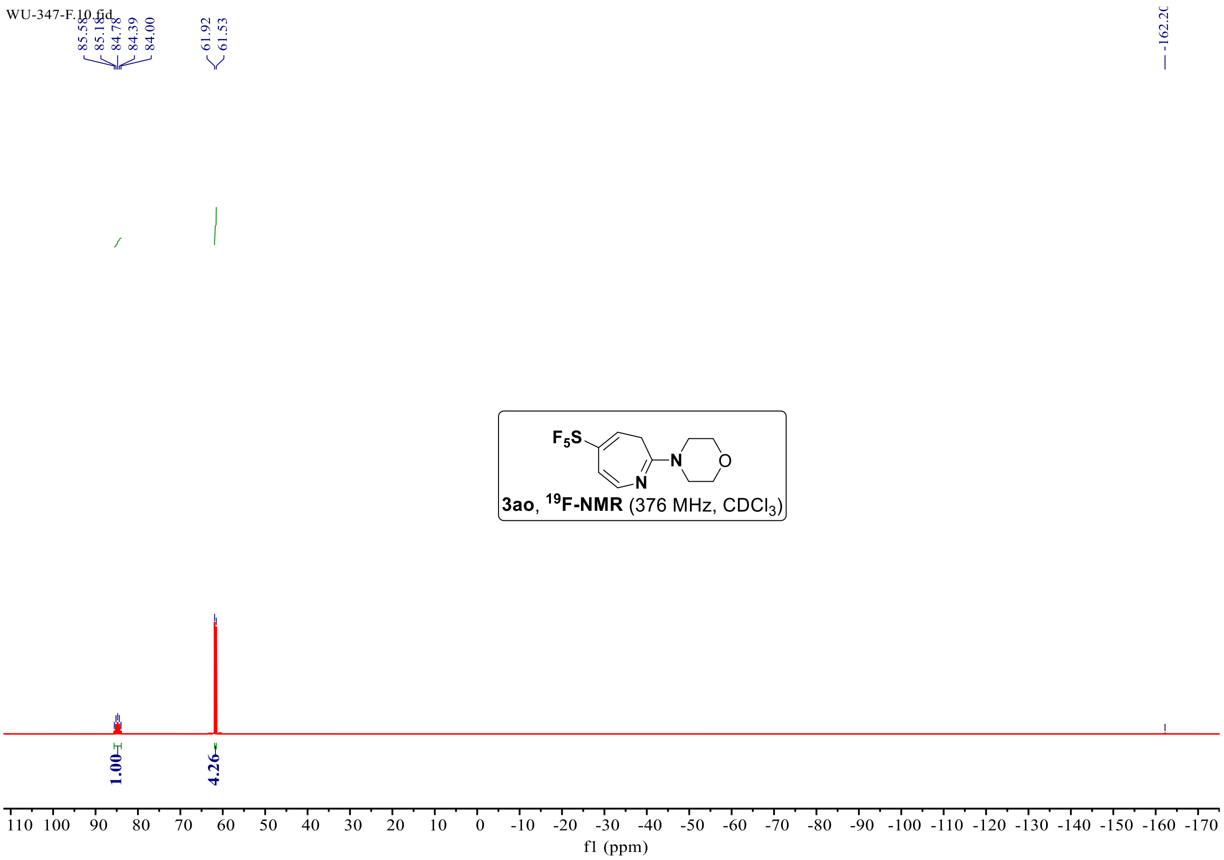
WU-347-F.10.fid
85.58
85.18
84.78
84.39
84.00
61.92
61.53
-162.20
F5S
N
N
O
3ao, 19F-NMR (376 MHz, CDCl3)
1.00
4.26
110 100 90 80 70 60 50 40 30 20 10 0 -10 -20 -30 -40 -50 -60 -70 -80 -90 -100 -110 -120 -130 -140 -150 -160 -170
f1 (ppm)

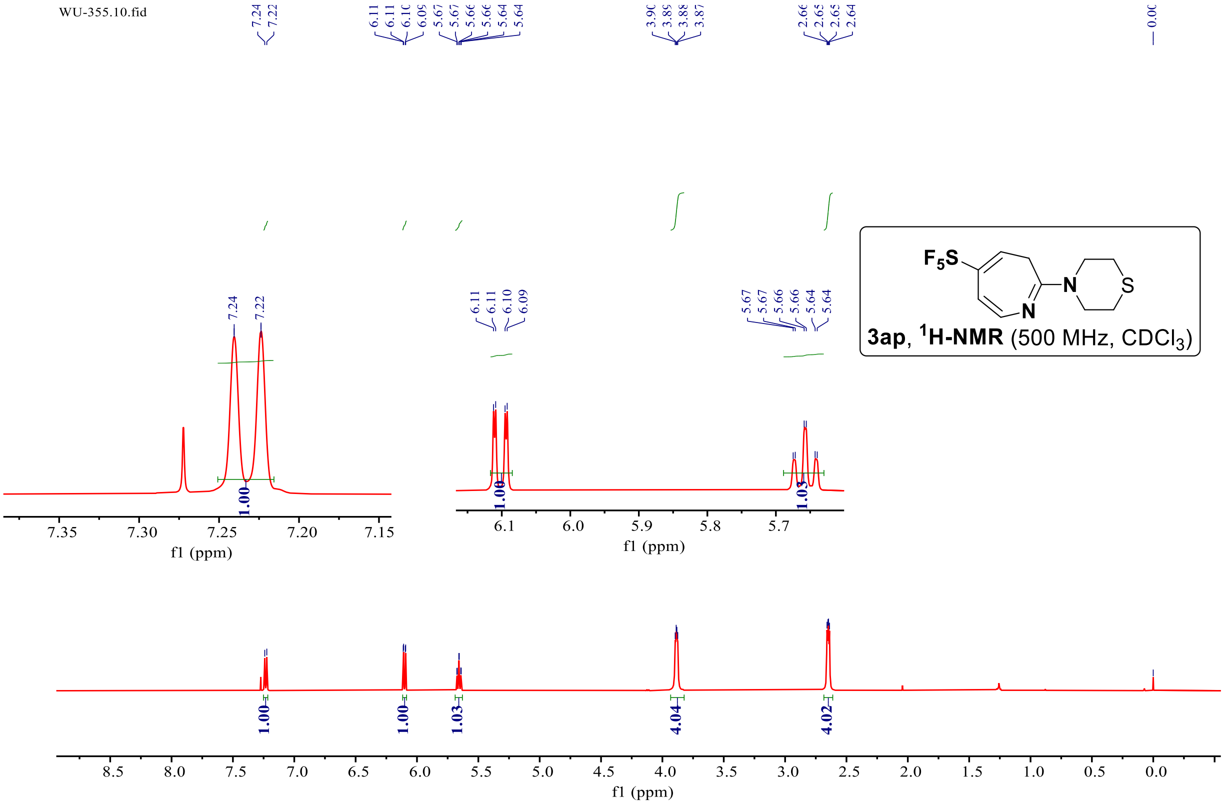
WU-355.10.fid
7.24
7.22
6.11
6.11
6.09
5.67
5.67
5.66
5.66
5.64
5.64
3.89
3.88
3.87
2.65
2.64
0.00
F5S
N
N
S
3ap, 1H-NMR (500 MHz, CDCl3)
1.00
7.35 7.30 7.25 7.20 7.15
f1 (ppm)
6.11
6.11
6.10
6.09
5.67
5.67
5.66
5.66
5.64
5.64
1.00
1.03
6.1 6.0 5.9 5.8 5.7
f1 (ppm)
1.00
1.00
1.03
4.04
4.02
8.5 8.0 7.5 7.0 6.5 6.0 5.5 5.0 4.5 4.0 3.5 3.0 2.5 2.0 1.5 1.0 0.5 0.0
f1 (ppm)

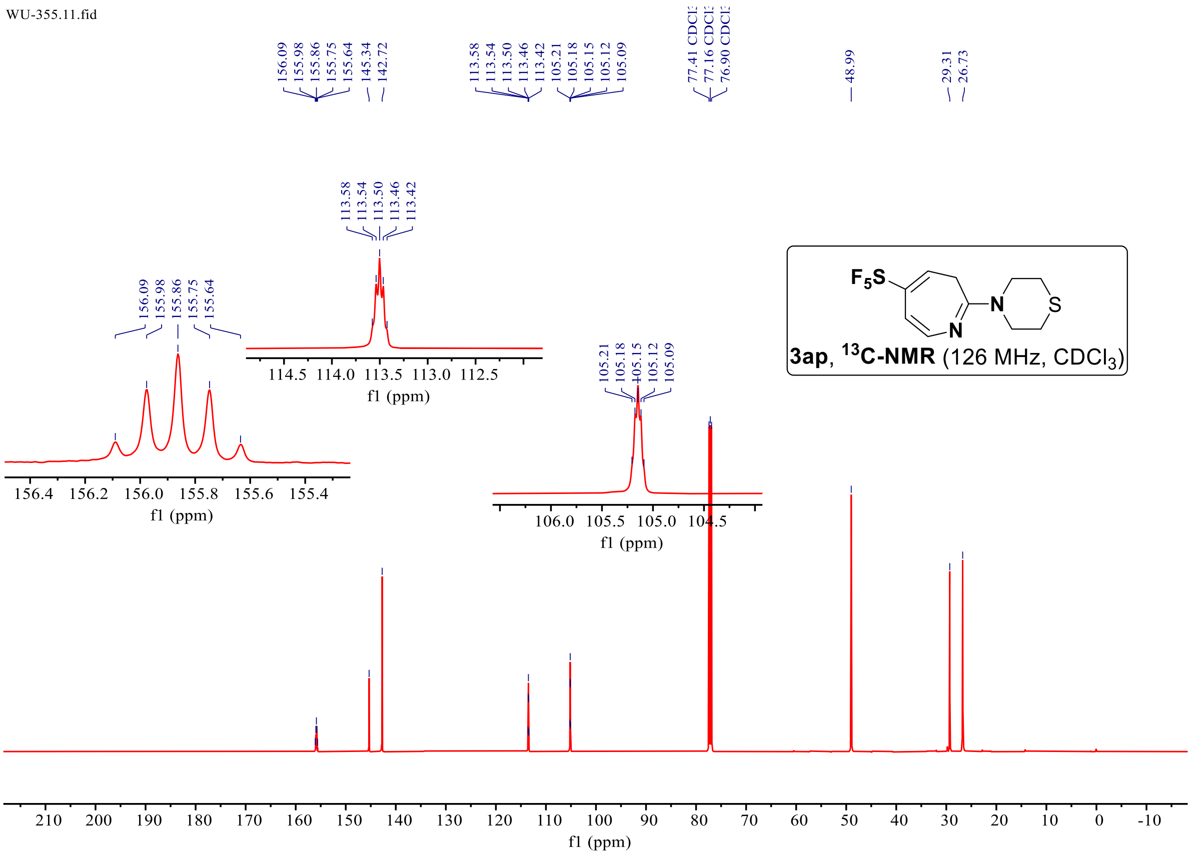

WU-355.11.fid
156.09
155.98
155.86
155.75
155.64
145.34
142.72
113.58
113.54
113.50
113.46
113.42
105.21
105.18
105.15
105.12
105.09
77.41 CDCl3
77.16 CDCl3
76.90 CDCl3
48.99
29.31
26.73
3ap, 13C-NMR (126 MHz, CDCl3)
f1 (ppm)

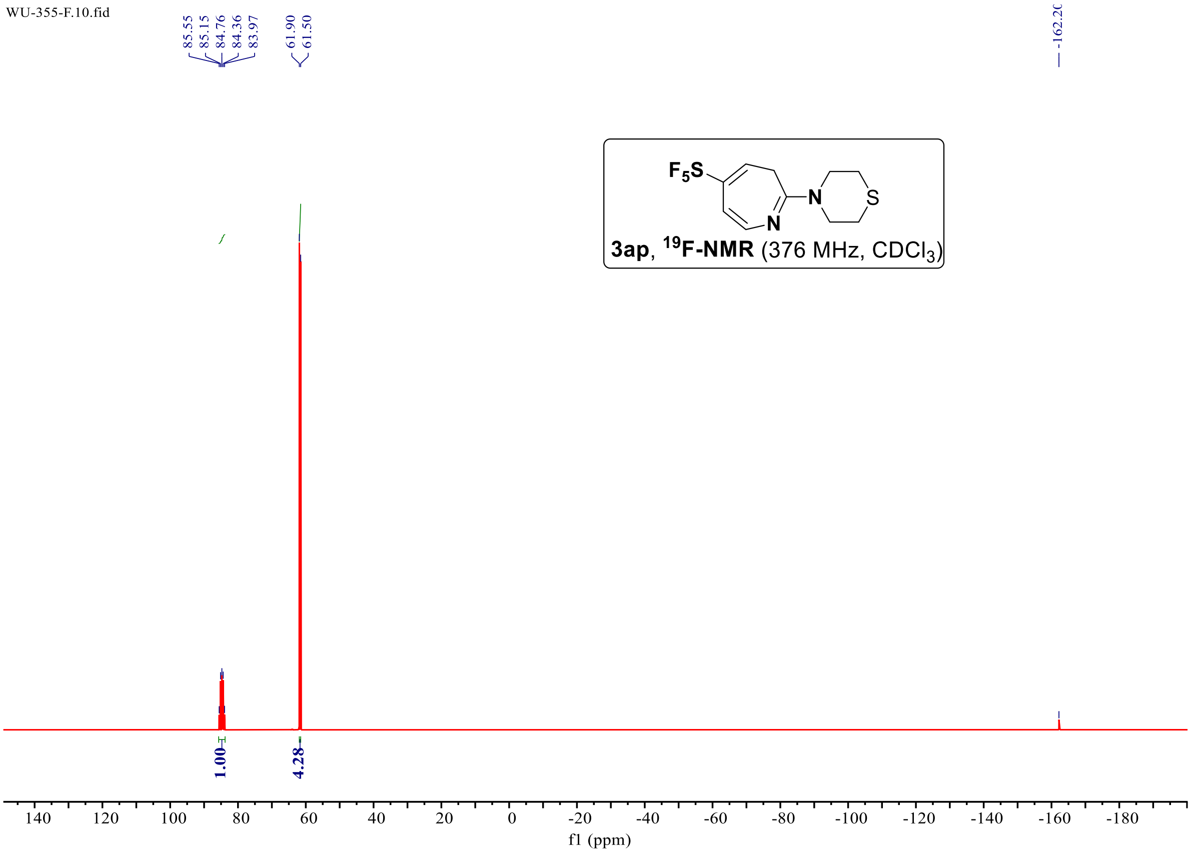

WU-355-F.10.fid
85.55
85.15
84.76
84.36
83.97
61.90
61.50
-162.20
1.00
4.28
3ap, 19F-NMR (376 MHz, CDCl3)
f1 (ppm)

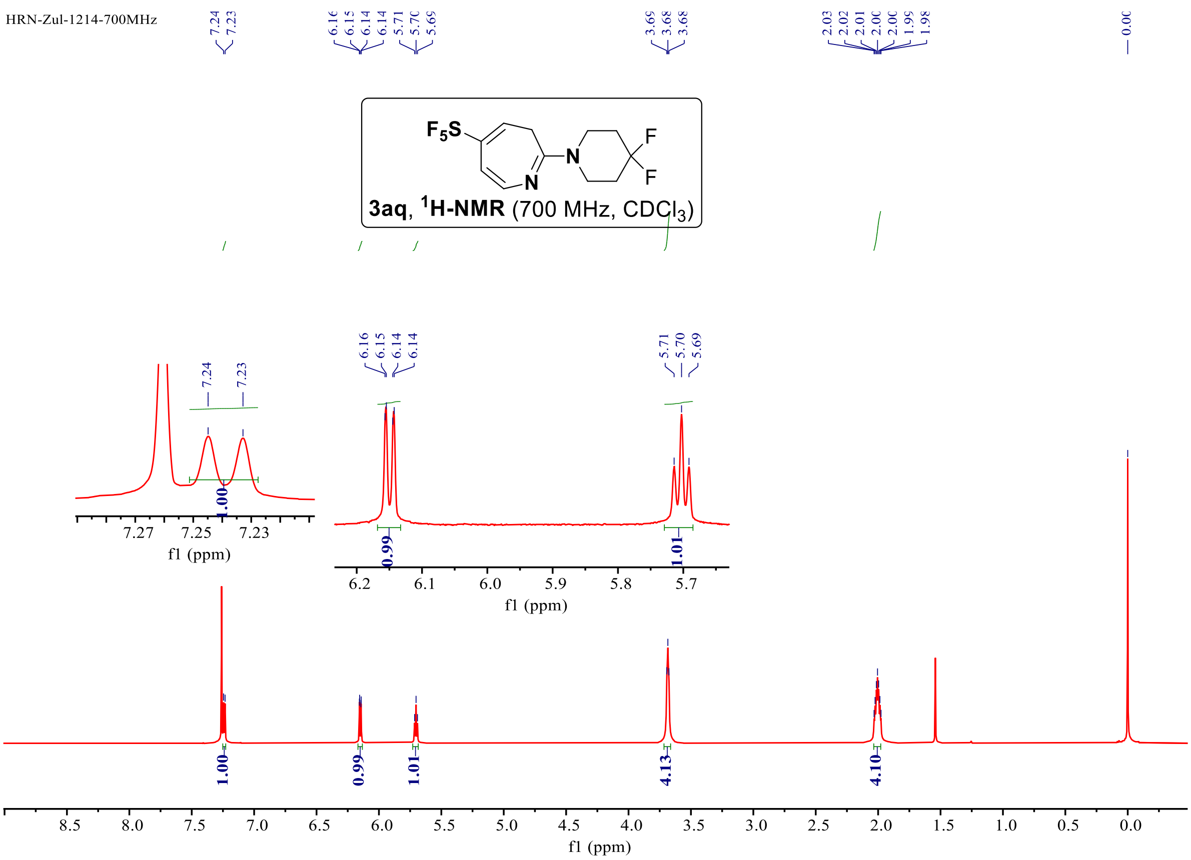
HRN-Zul-1214-700MHz
3aq, 1H-NMR (700 MHz, CDCl3)
f1 (ppm)

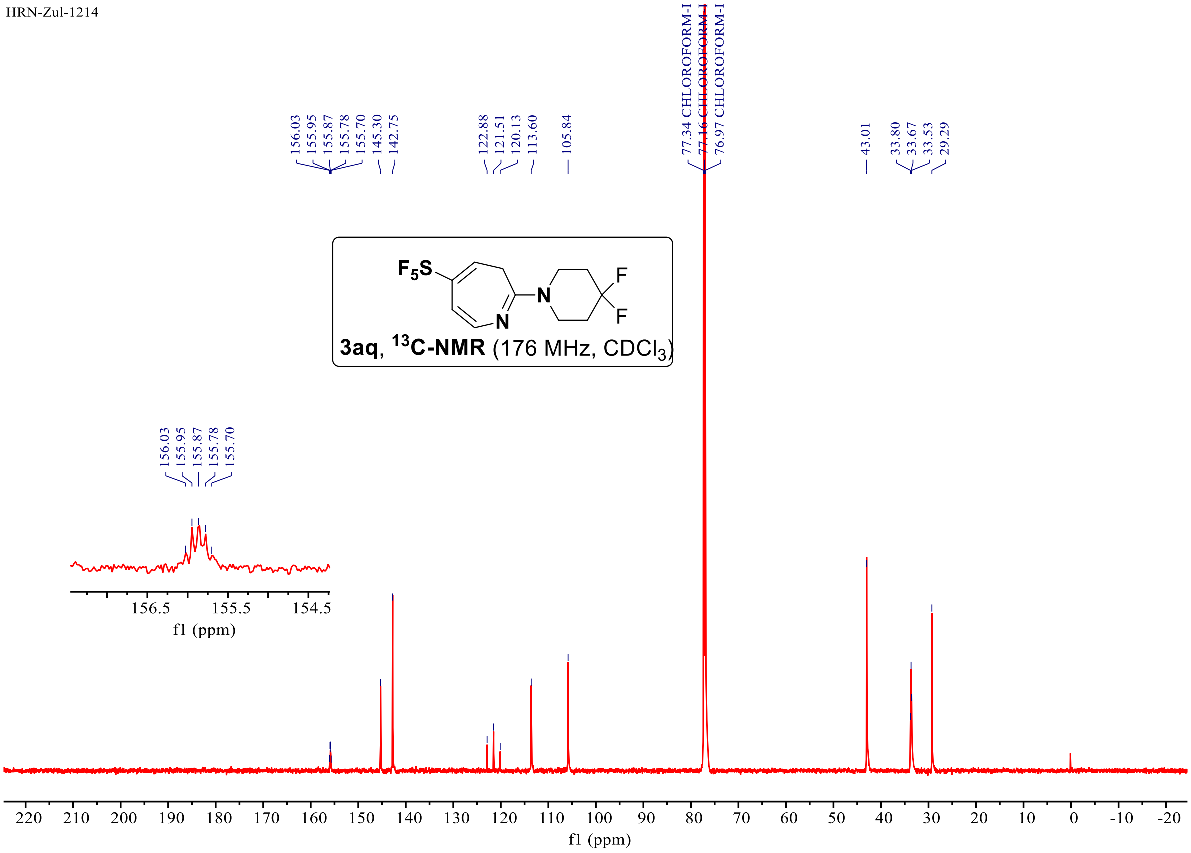
HRN-Zul-1214
3aq, 13C-NMR (176 MHz, CDCl3)
f1 (ppm)

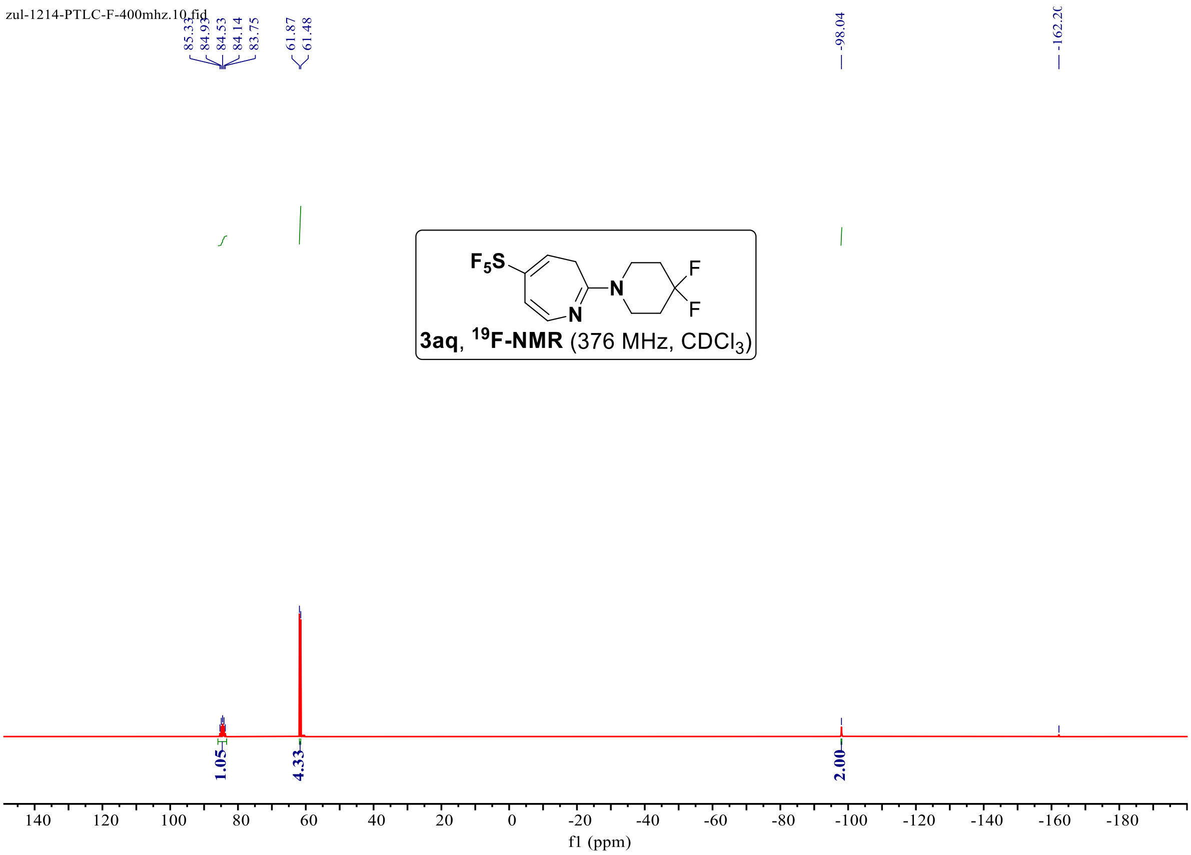
zul-1214-PTLC-F-400mhz.10.fid
85.33
84.93
84.53
84.14
83.75
61.87
61.48
-98.04
F5S
N
N
F
F
3aq, 19F-NMR (376 MHz, CDCl3)
1.05
4.33
2.00
140 120 100 80 60 40 20 0 -20 -40 -60 -80 -100 -120 -140 -160 -180
f1 (ppm)

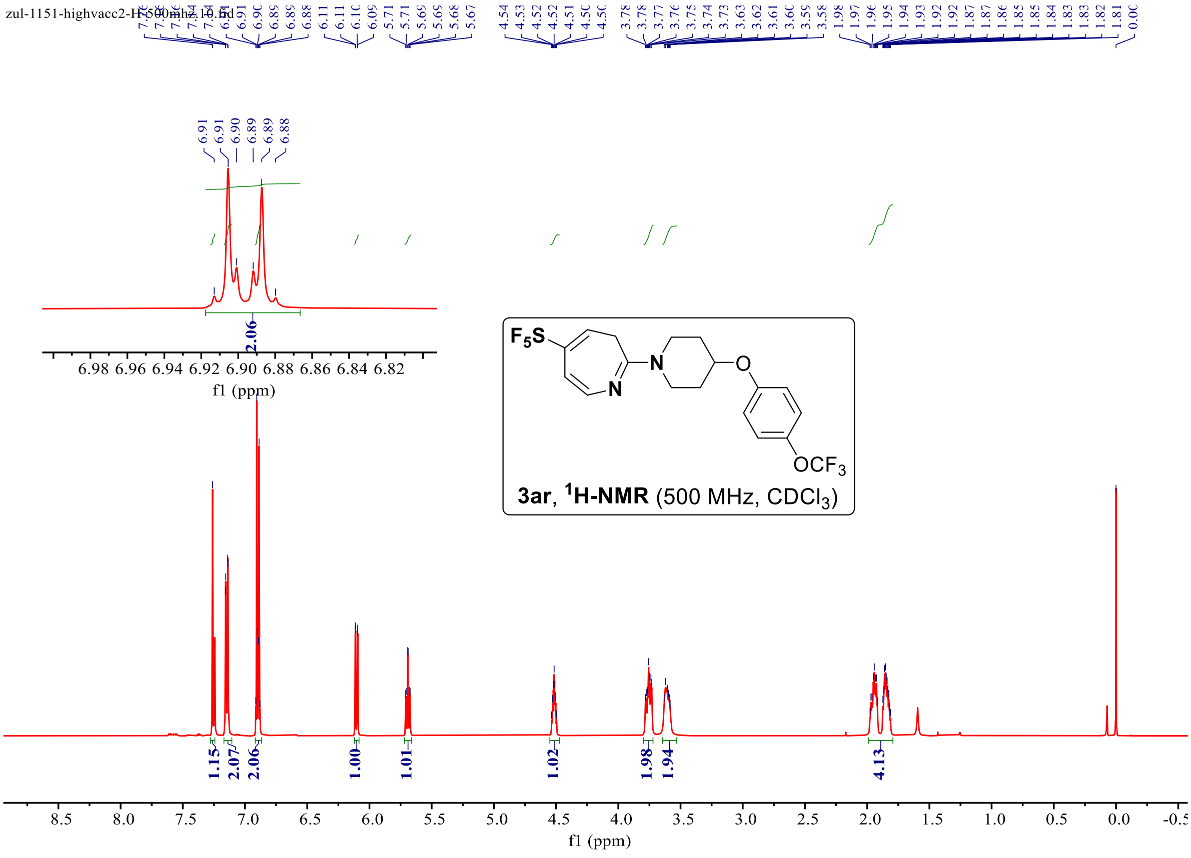
zul-1151-highvacc2-H-500mhz.10.fid
6.91
6.91
6.90
6.89
6.89
6.88
2.06
6.98 6.96 6.94 6.92 6.90 6.88 6.86 6.84 6.82
f1 (ppm)
F5S
N
N
O
OCF3
3ar, 1H-NMR (500 MHz, CDCl3)
1.15
2.07
2.06
1.00
1.01
1.02
1.98
1.94
4.13
8.5 8.0 7.5 7.0 6.5 6.0 5.5 5.0 4.5 4.0 3.5 3.0 2.5 2.0 1.5 1.0 0.5 0.0
f1 (ppm)

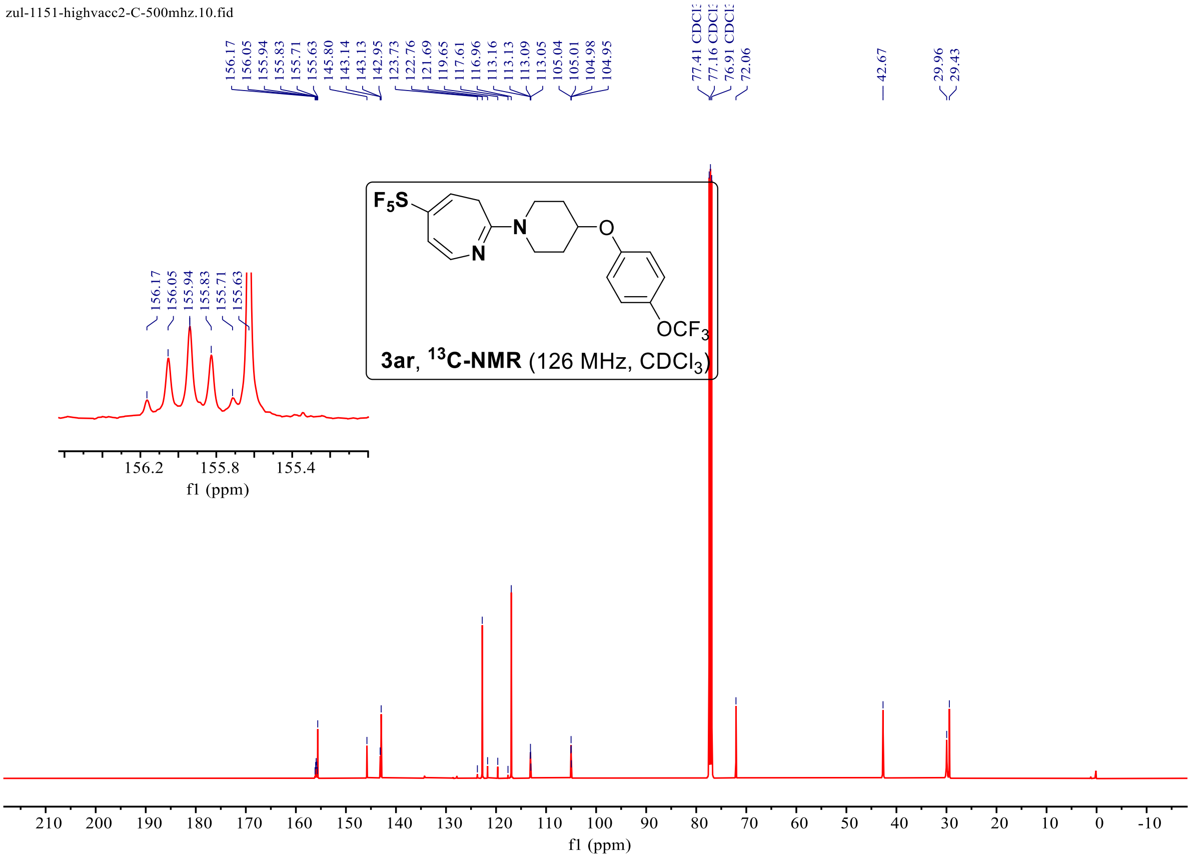
zul-1151-highvacc2-C-500mhz.10.fid
156.17 156.05 155.94 155.83 155.71 155.63 145.80 143.14 143.13 142.95 123.73 122.76 121.69 119.65 117.61 116.96 113.16 113.13 113.09 113.05 105.04 105.01 104.98 104.95
77.41 CDCl3 77.16 CDCl3 76.91 CDCl3 72.06
42.67
29.96 29.43
F5S
N
N
O
OCF3
3ar, 13C-NMR (126 MHz, CDCl3)
156.2 155.8 155.4
f1 (ppm)
210 200 190 180 170 160 150 140 130 120 110 100 90 80 70 60 50 40 30 20 10 0 -10
f1 (ppm)

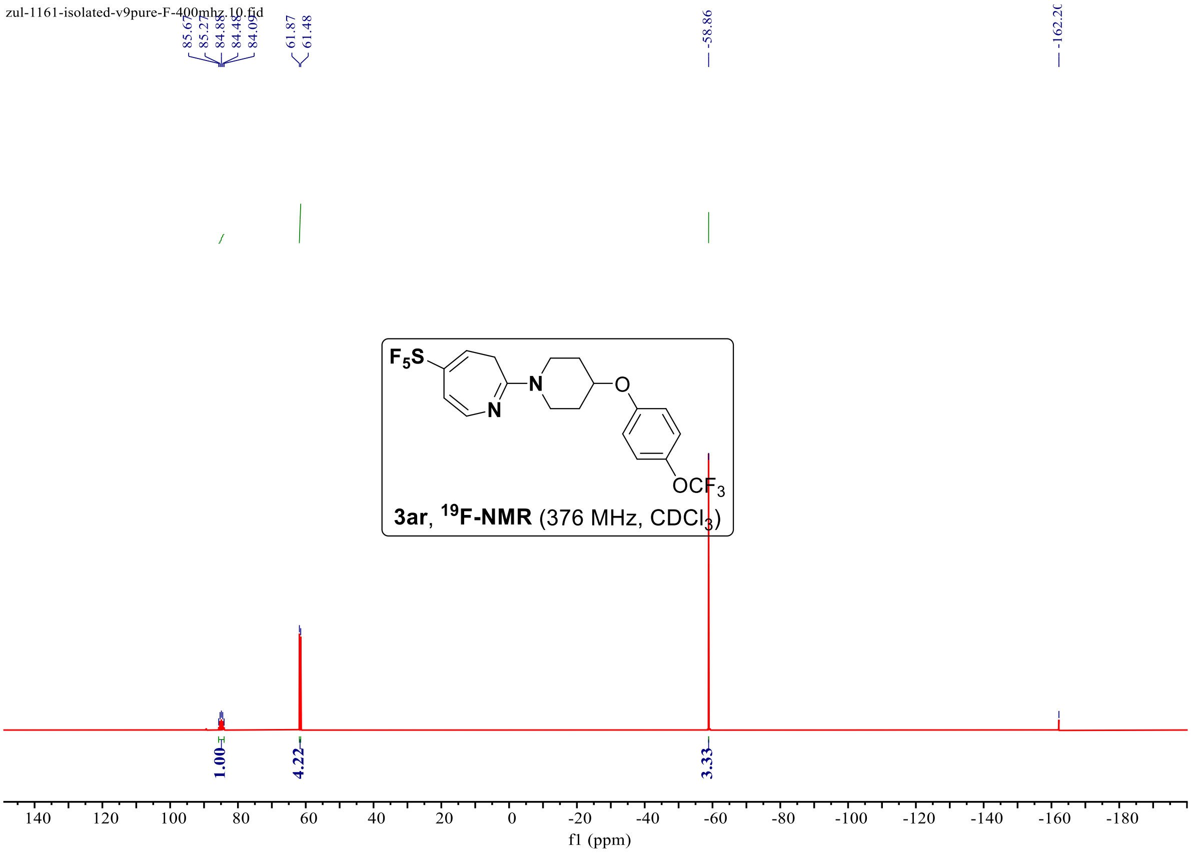
zul-1161-isolated-v9pure-F-400mhz.10.fid
85.67 85.27 84.88 84.48 84.09
61.87 61.48
-58.86
-162.20
F5S
N
N
O
OCF3
3ar, 19F-NMR (376 MHz, CDCl3)
1.00
4.22
3.33
140 120 100 80 60 40 20 0 -20 -40 -60 -80 -100 -120 -140 -160 -180
f1 (ppm)

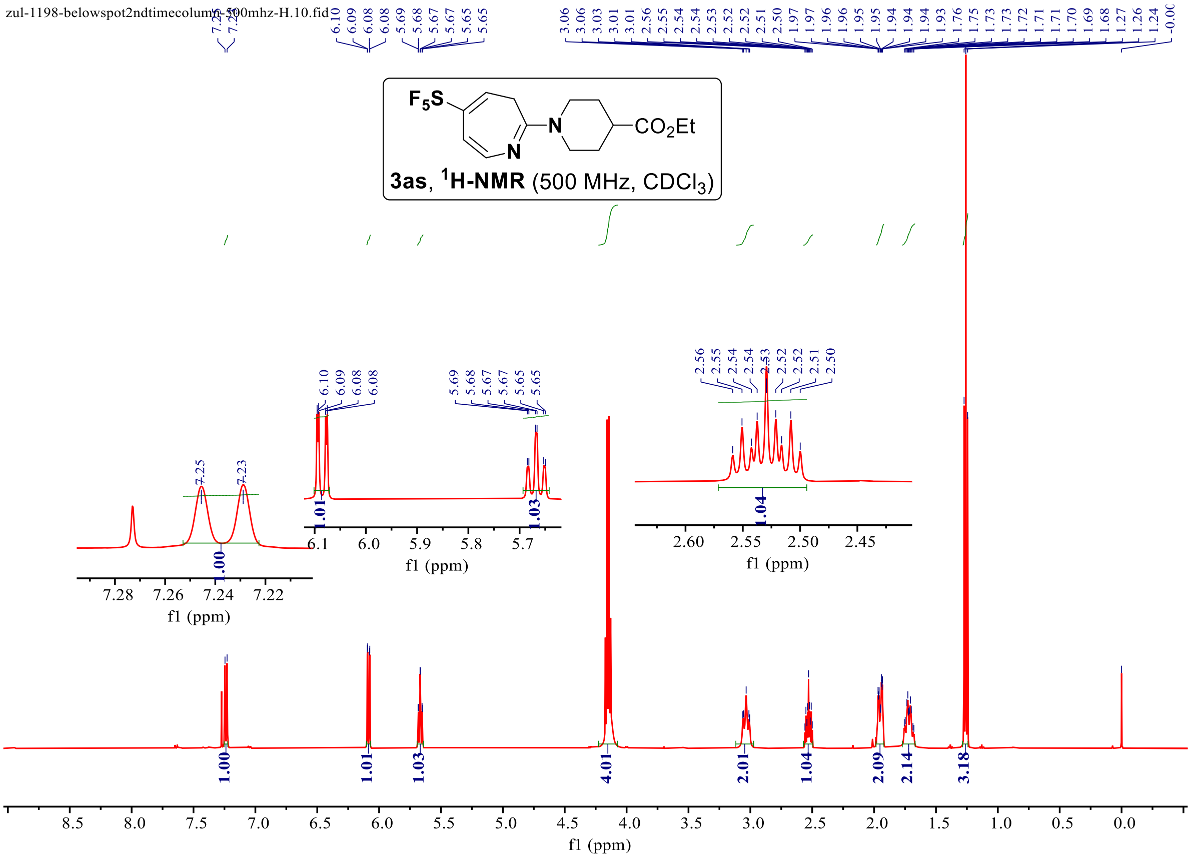
zul-1198-belowspot2ndtimecolumn-500mhz-H.10.fid
7.25 7.23 6.10 6.09 6.08 6.08 5.69 5.68 5.67 5.67 5.65 5.65
3.06 3.06 3.03 3.01 3.01 2.56 2.55 2.54 2.54 2.53 2.52 2.52 2.51 2.50 1.97 1.97 1.96 1.96 1.95 1.95 1.94 1.94 1.94 1.93 1.76 1.75 1.73 1.73 1.72 1.71 1.71 1.70 1.69 1.68 1.27 1.26 1.24 -0.00
F5S
N
N
CO2Et
3as, 1H-NMR (500 MHz, CDCl3)
1.00
1.01
1.03
4.01
2.01
1.04
2.09
2.14
3.18
f1 (ppm)

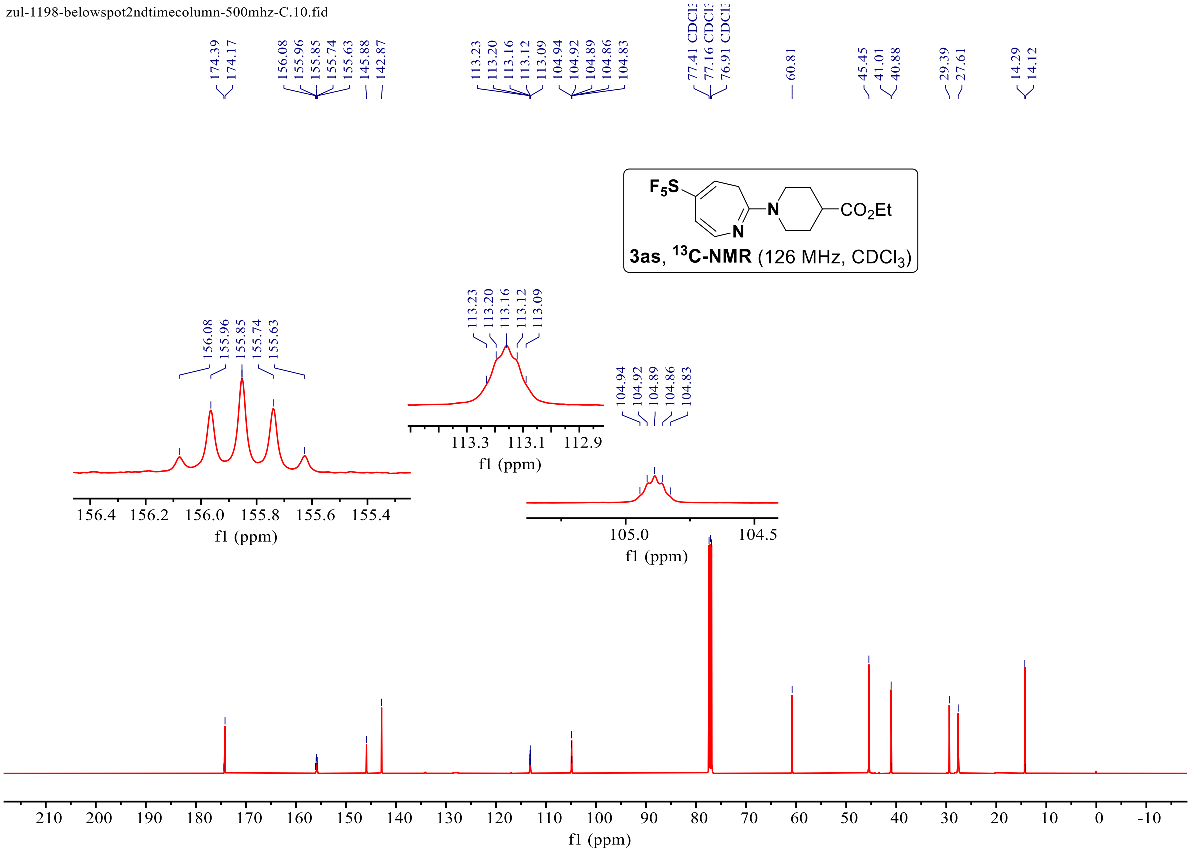
zul-1198-belowspot2ndtimecolumn-500mhz-C.10.fid
174.39 174.17 156.08 155.96 155.85 155.74 155.63 145.88 142.87
113.23 113.20 113.16 113.12 113.09 104.94 104.92 104.89 104.86 104.83
77.41 CDCl3 77.16 CDCl3 76.91 CDCl3
60.81 45.45 41.01 40.88 29.39 27.61 14.29 14.12
F5S
N
N
CO2Et
3as, 13C-NMR (126 MHz, CDCl3)
f1 (ppm)

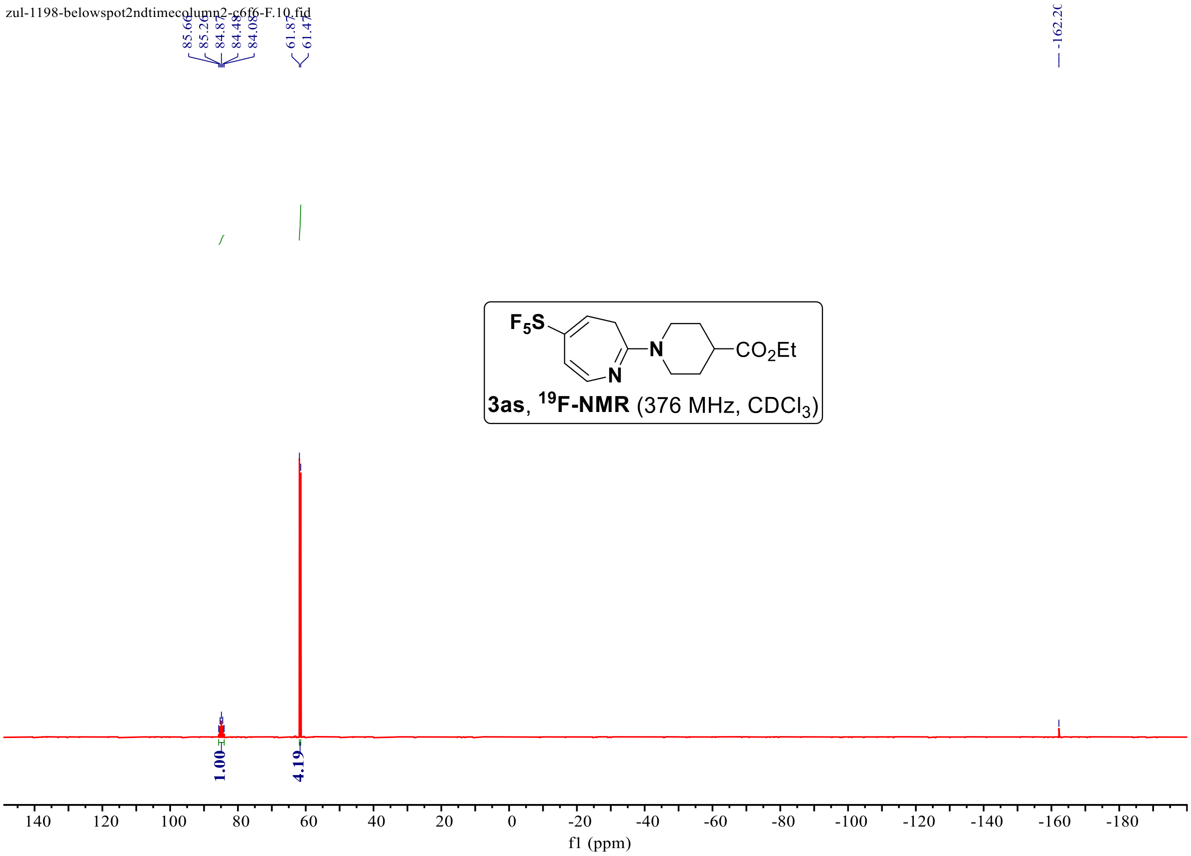
zul-1198-belowspot2ndtimecolumn2-c6f6-F.10.fid
3as, 19F-NMR (376 MHz, CDCl3)
f1 (ppm)

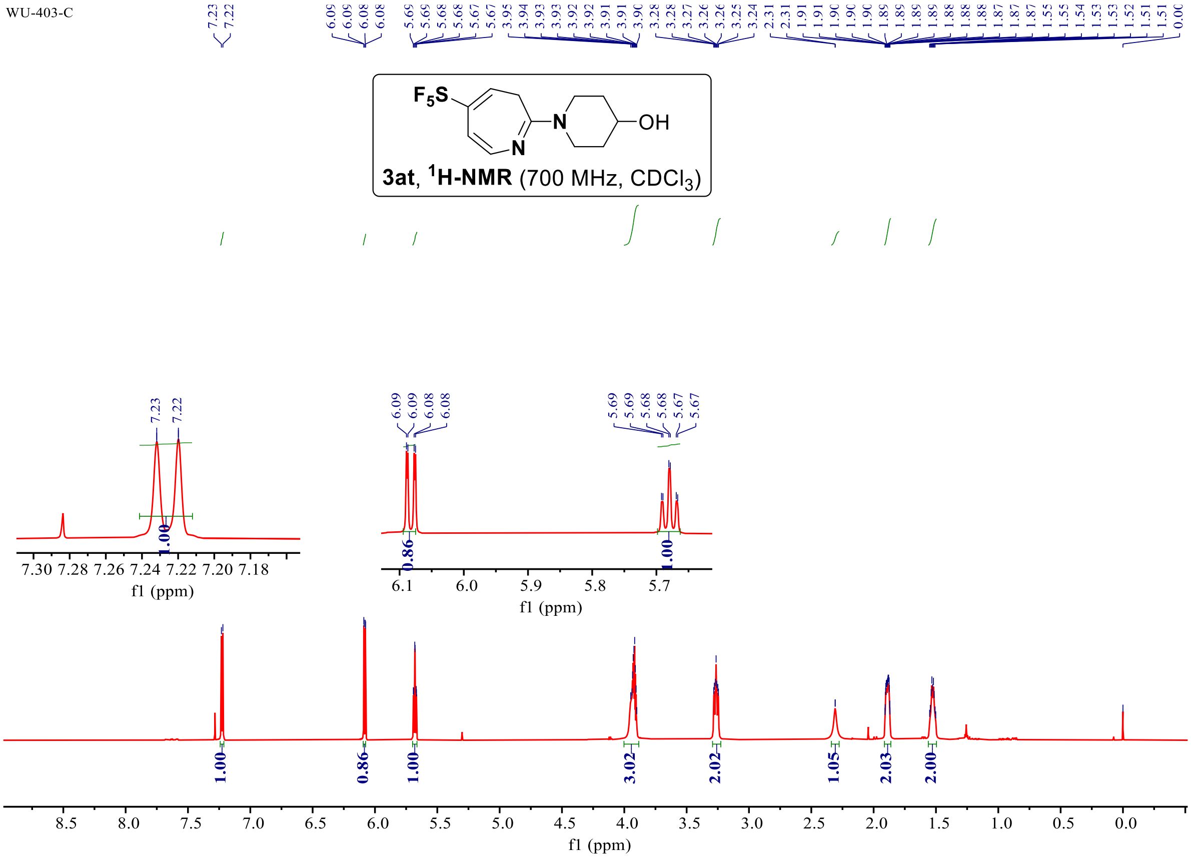
WU-403-C
3at, 1H-NMR (700 MHz, CDCl3)
f1 (ppm)

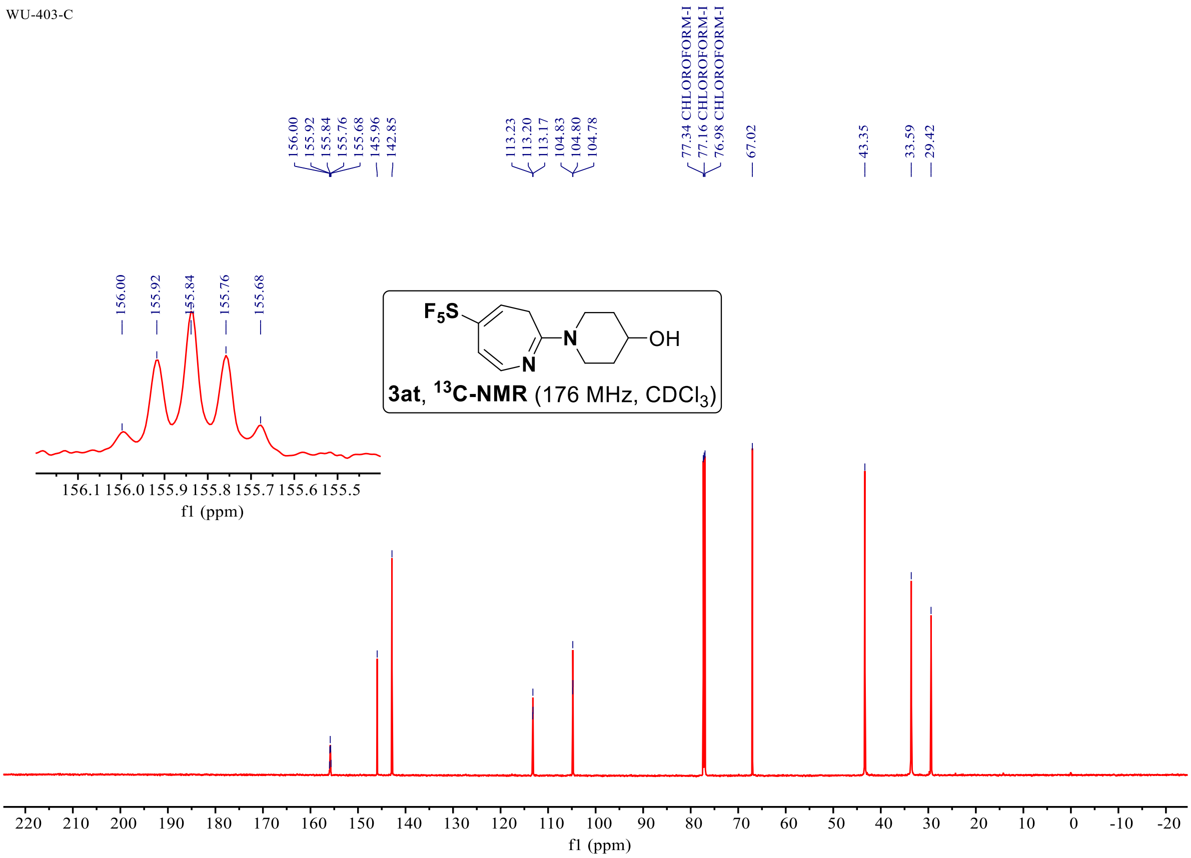
WU-403-C
156.00
155.92
155.84
155.76
155.68
145.96
142.85
113.23
113.20
113.17
104.83
104.80
104.78
77.34 CHLOROFORM-D
77.16 CHLOROFORM-D
76.98 CHLOROFORM-D
67.02
43.35
33.59
29.42
F_5S
N
OH
3at, ^{13}C-NMR (176 MHz, $CDCl_3$)
156.1 156.0 155.9 155.8 155.7 155.6 155.5
f1 (ppm)
220 210 200 190 180 170 160 150 140 130 120 110 100 90 80 70 60 50 40 30 20 10 0 -10 -20
f1 (ppm)

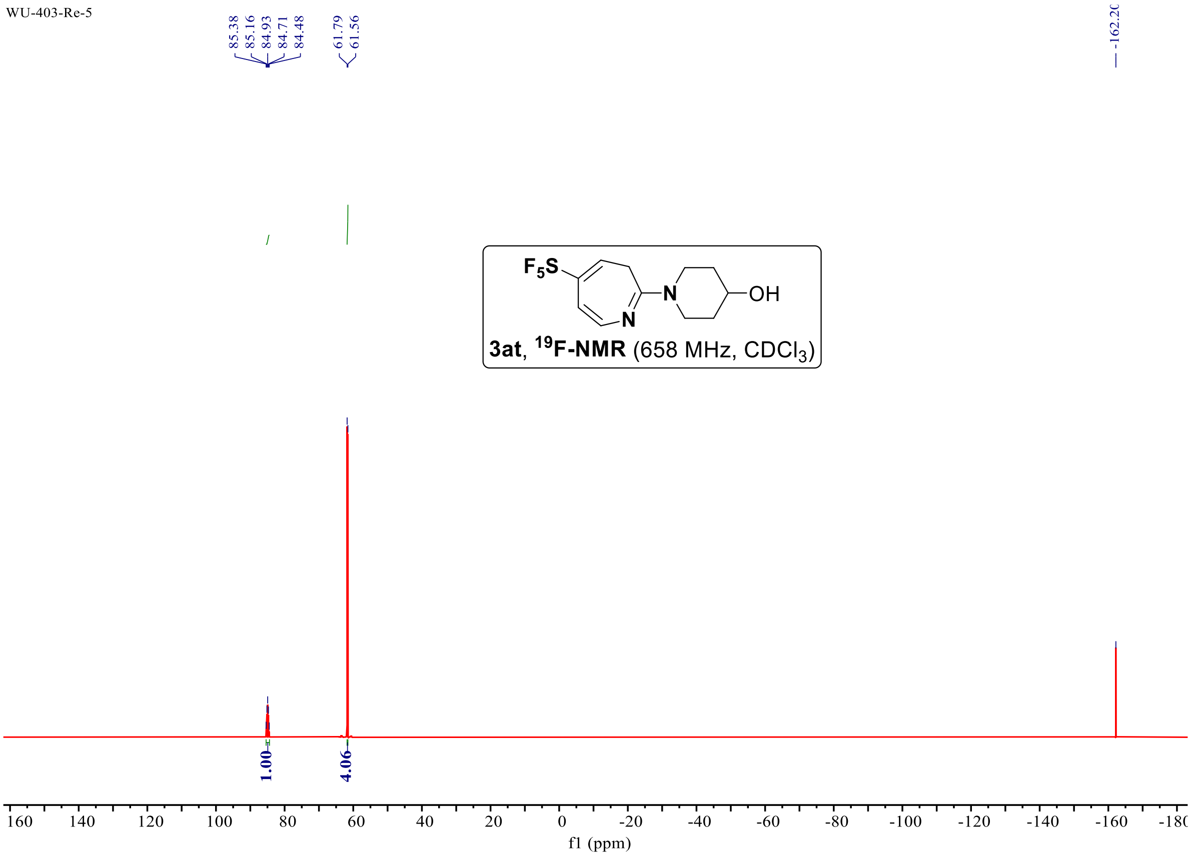
WU-403-Re-5
85.38
85.16
84.93
84.71
84.48
61.79
61.56
-162.20
F_5S
N
OH
3at, ^{19}F-NMR (658 MHz, $CDCl_3$)
1.00
4.06
160 140 120 100 80 60 40 20 0 -20 -40 -60 -80 -100 -120 -140 -160 -180
f1 (ppm)

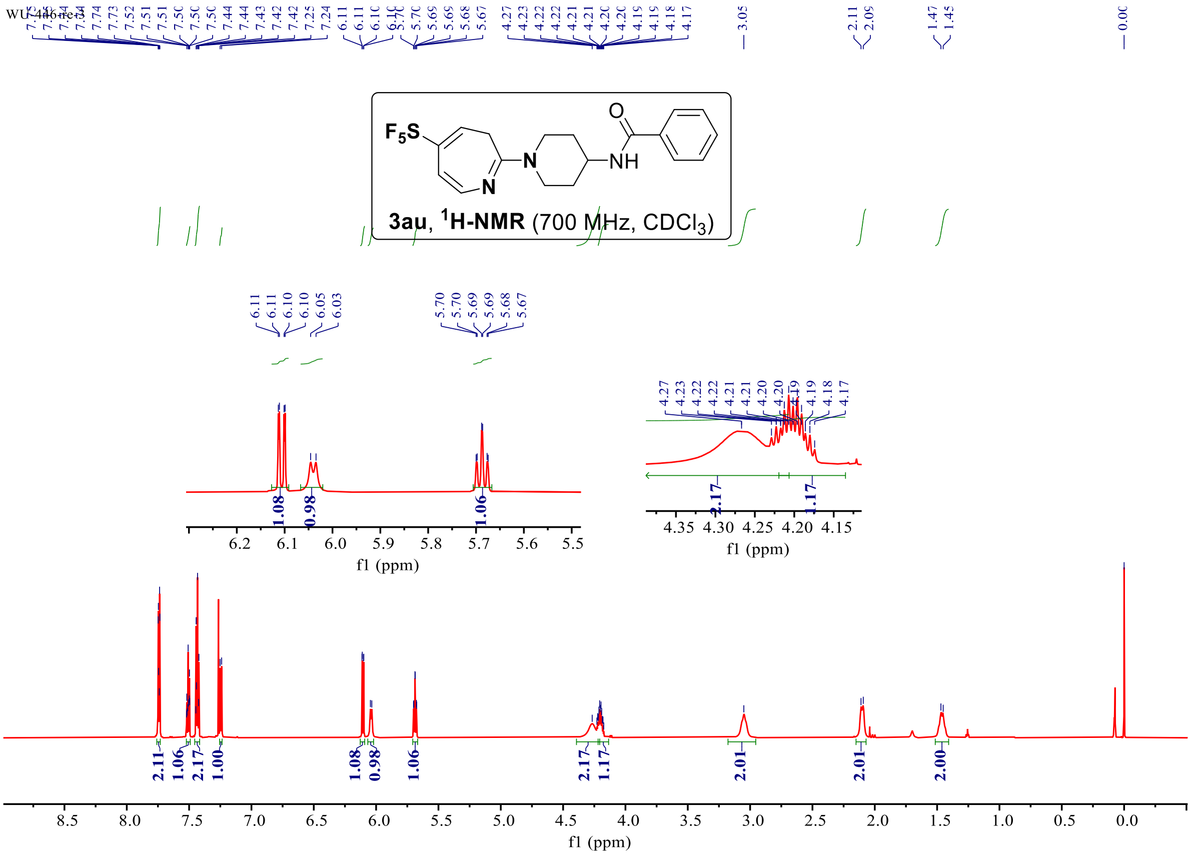
WU-446-re-3
3au, ¹H-NMR (700 MHz, CDCl₃)
f1 (ppm)

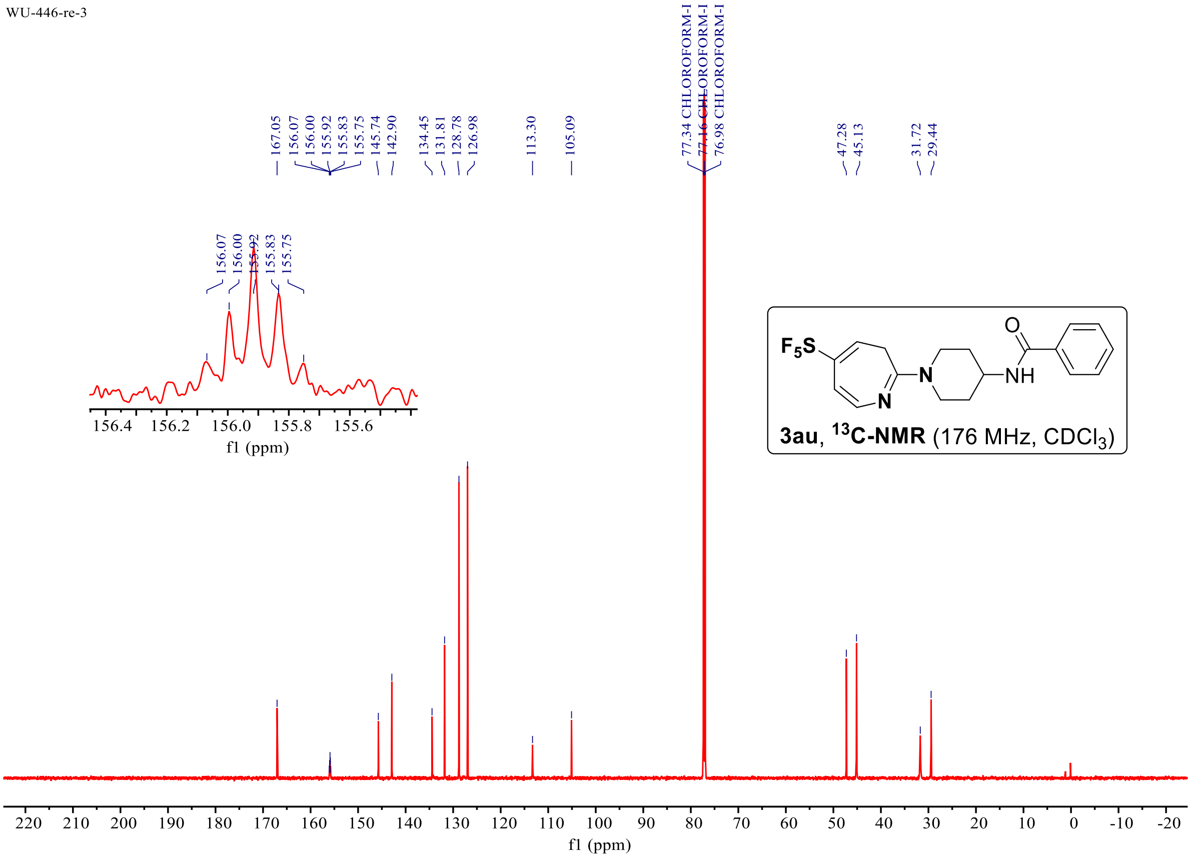
WU-446-re-3
167.05
156.07
156.00
155.92
155.83
155.75
145.74
142.90
134.45
131.81
128.78
126.98
113.30
105.09
77.34 CHLOROFORM-1
77.16 CHLOROFORM-1
76.98 CHLOROFORM-1
47.28
45.13
31.72
29.44
3au, ¹³C-NMR (176 MHz, CDCl₃)
f1 (ppm)

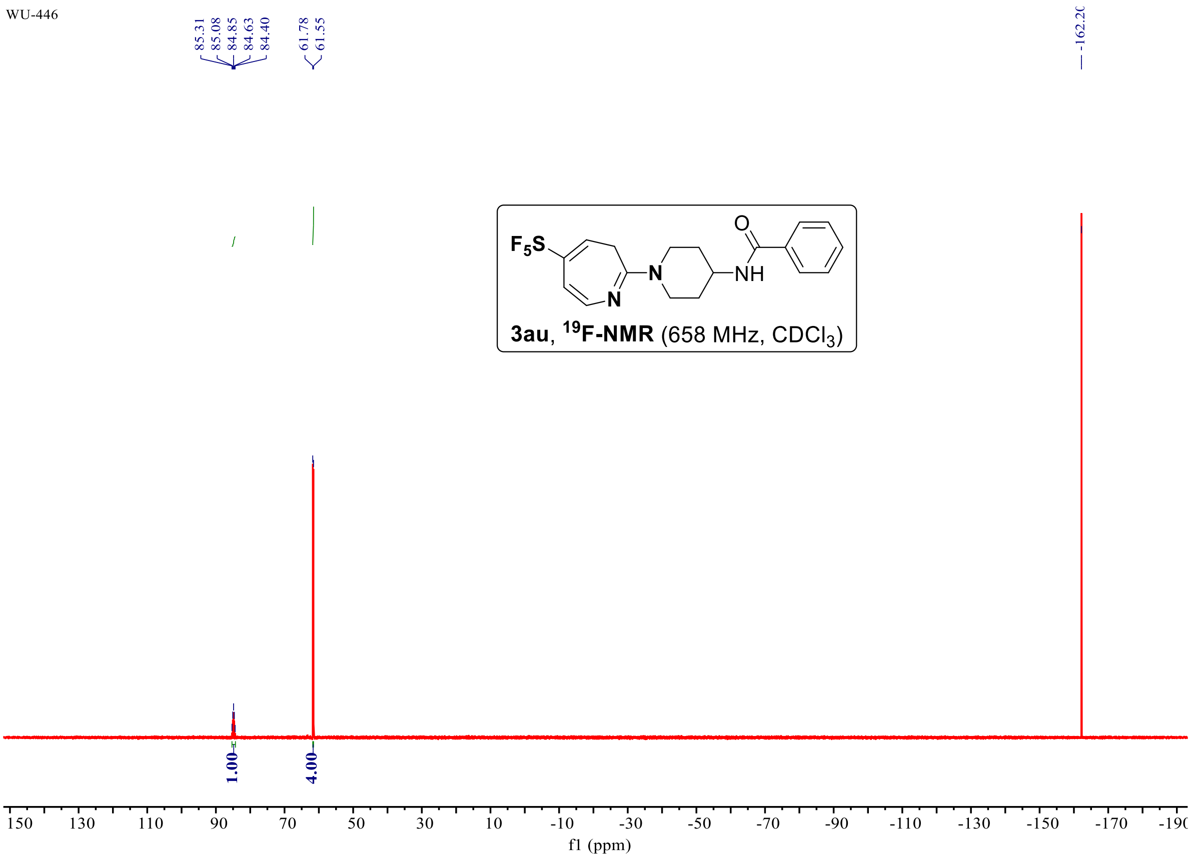
WU-446
85.31
85.08
84.85
84.63
84.40
61.78
61.55
-162.20
F5S
O
N
NH
N
3au, 19F-NMR (658 MHz, CDCl3)
1.00
4.00
f1 (ppm)

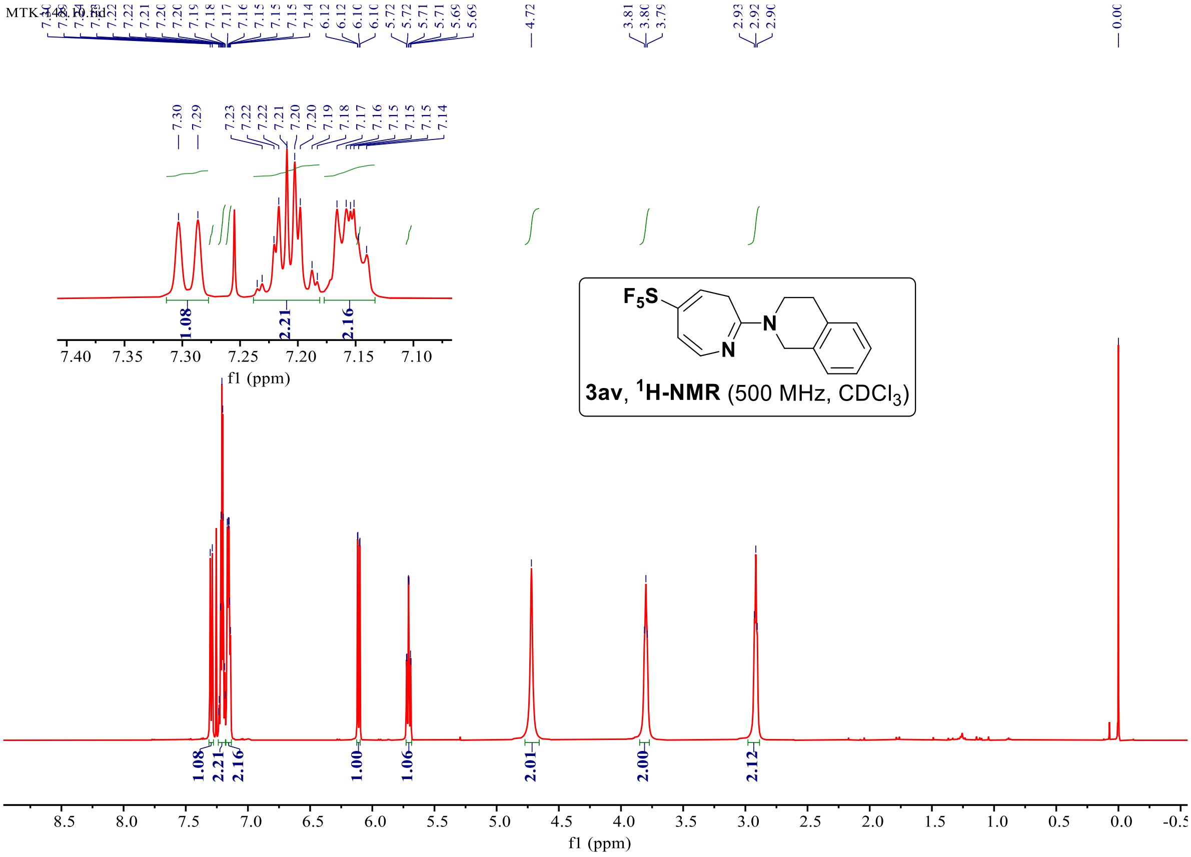
4.72
3.81
2.93
2.92
0.00
F5S
N
N
3av, 1H-NMR (500 MHz, CDCl3)
1.08
2.21
2.16
1.00
1.06
2.01
2.00
2.12
f1 (ppm)

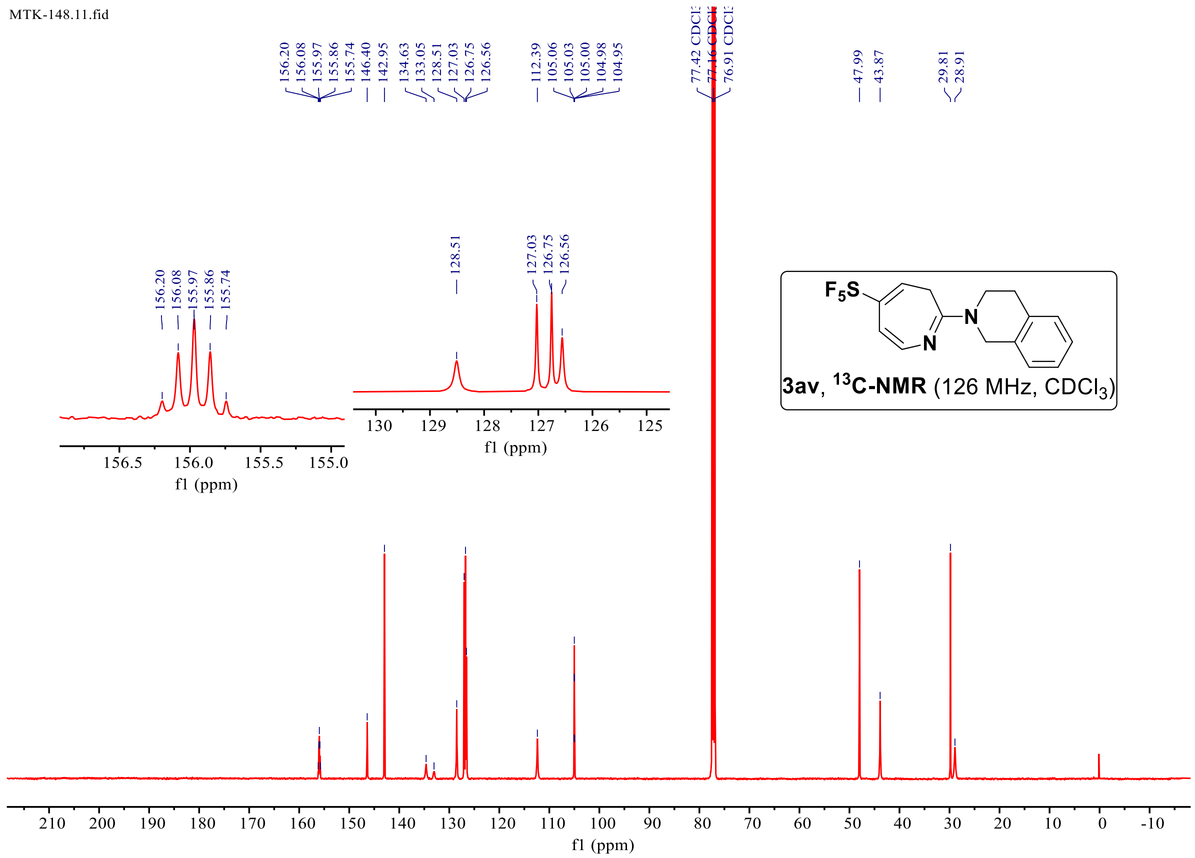
MTK-148.11.fid
156.20
156.08
155.97
155.86
155.74
146.40
142.95
134.63
133.05
128.51
127.03
126.75
126.56
112.39
105.06
105.03
105.00
104.98
104.95
77.42 CDCl3
77.16 CDCl3
76.91 CDCl3
47.99
43.87
29.81
28.91
F5S
N
3av, 13C-NMR (126 MHz, CDCl3)
f1 (ppm)

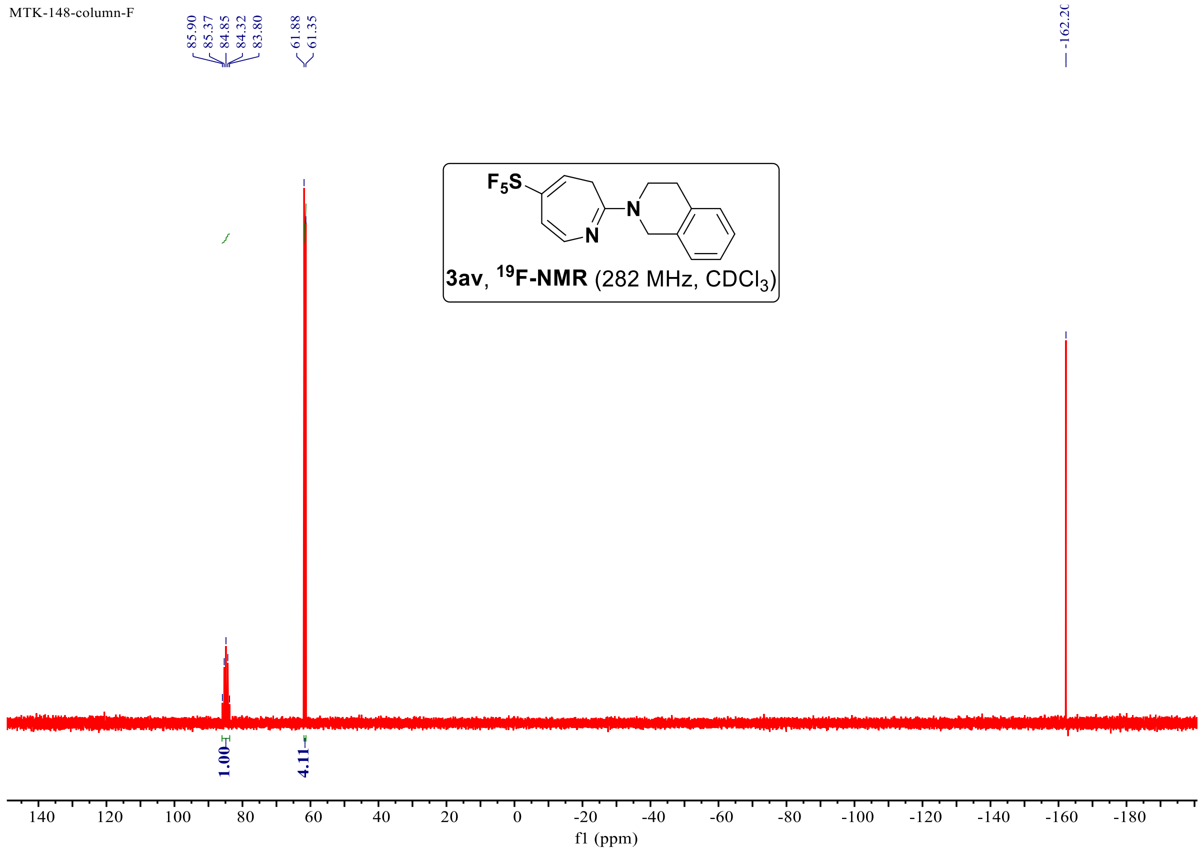
MTK-148-column-F
85.90
85.37
84.85
84.32
83.80
61.88
61.35
-162.20
F5S
N
3av, 19F-NMR (282 MHz, CDCl3)
1.00
4.11
f1 (ppm)

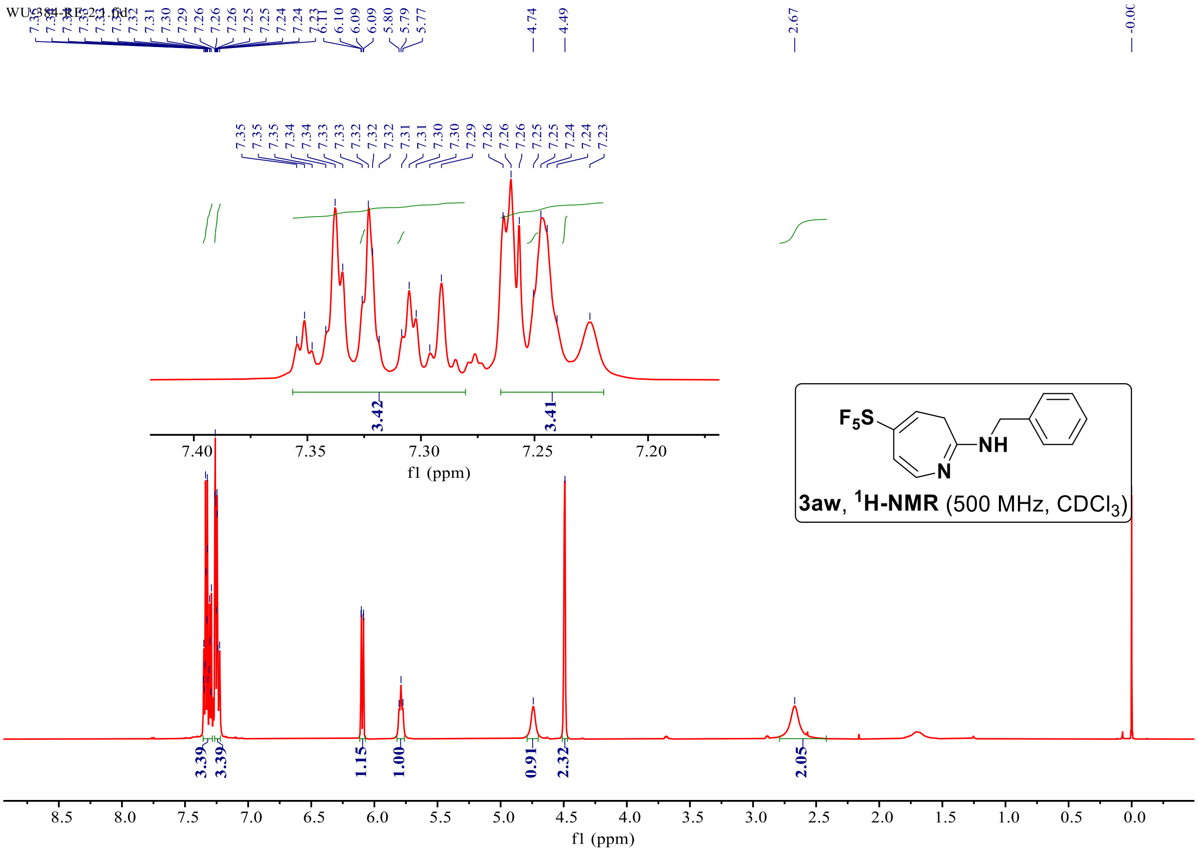

7.35 7.35 7.35 7.34 7.34 7.33 7.33 7.32 7.32 7.32 7.31 7.31 7.30 7.30 7.29 7.26 7.26 7.26 7.25 7.25 7.24 7.24 7.23
6.11 6.10 6.09 6.09 5.80 5.79 5.77
4.74
4.49
2.67
-0.00
3.42
3.41
3.39
3.39
1.15
1.00
0.91
2.32
2.05
f1 (ppm)
F5S
NH
N
3aw, 1H-NMR (500 MHz, CDCl3)

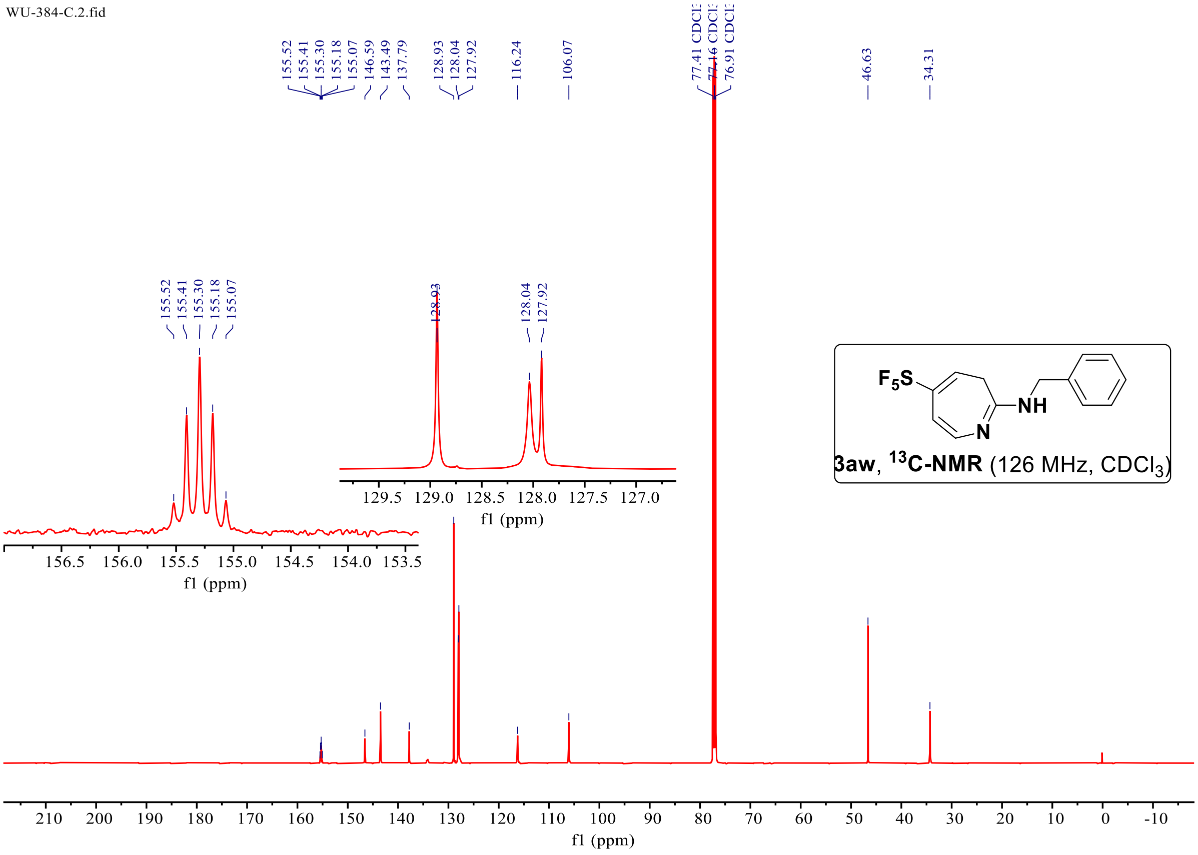

WU-384-C.2.fid
155.52 155.41 155.30 155.18 155.07 146.59 143.49 137.79 128.93 128.04 127.92 116.24 106.07
77.41 CDCl3 77.16 CDCl3 76.91 CDCl3
46.63
34.31
f1 (ppm)
F5S
NH
N
3aw, 13C-NMR (126 MHz, CDCl3)

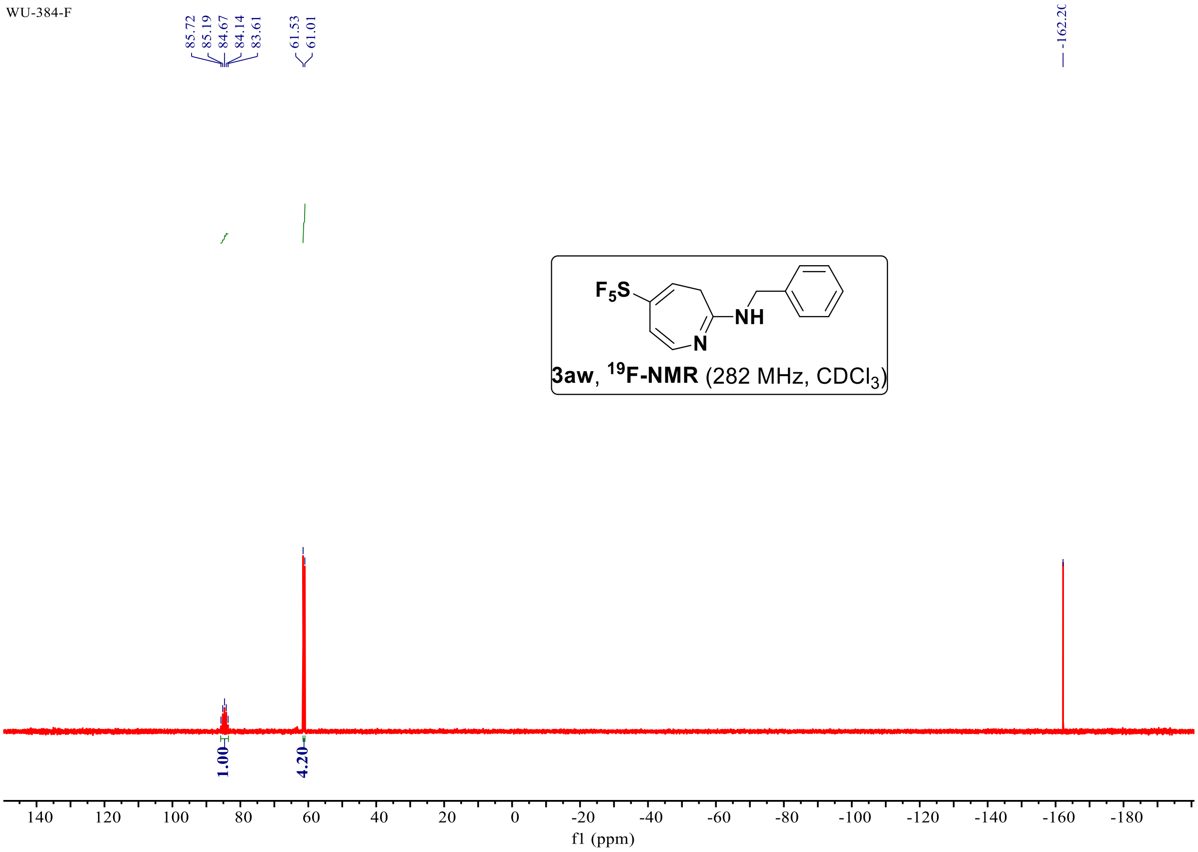
WU-384-F
85.72
85.19
84.67
84.14
83.61
61.53
61.01
-162.20
F5S
NH
N
3aw, 19F-NMR (282 MHz, CDCl3)
1.00
4.20
140 120 100 80 60 40 20 0 -20 -40 -60 -80 -100 -120 -140 -160 -180
f1 (ppm)

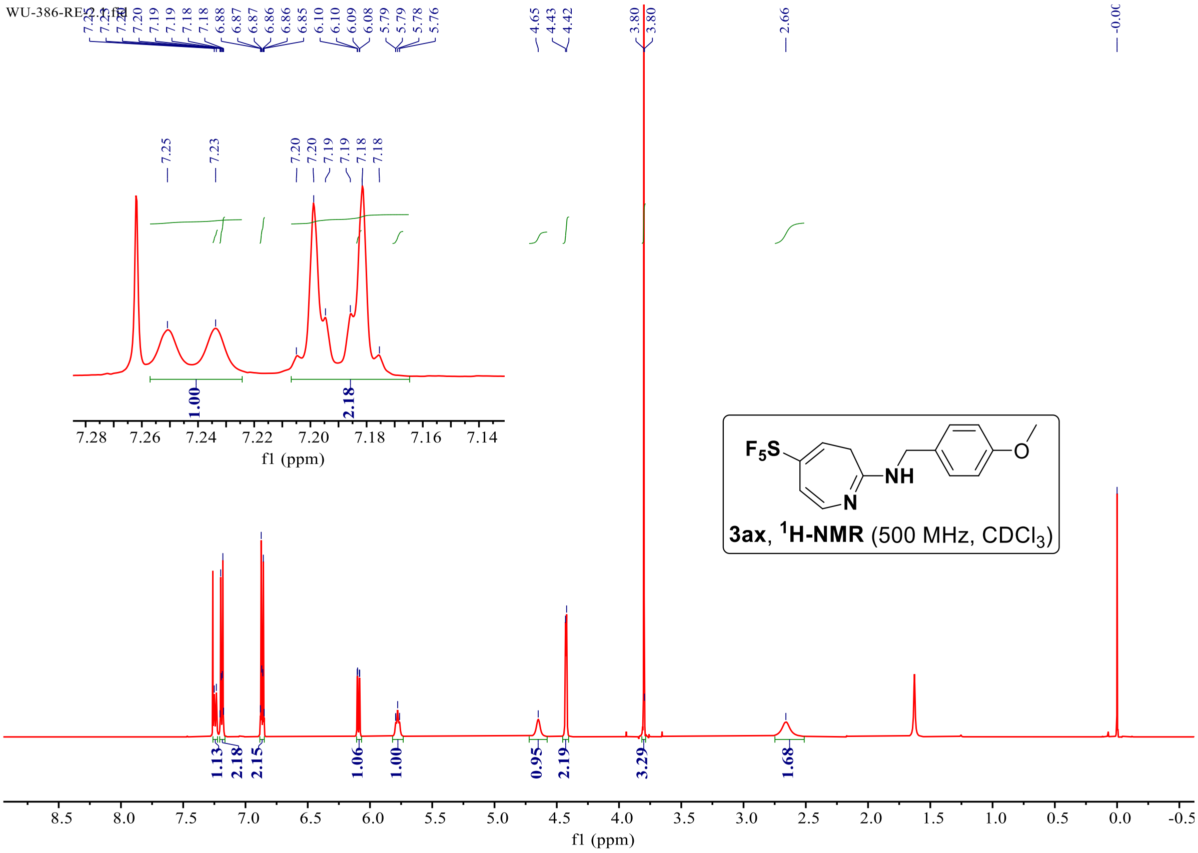
7.20
7.19
7.19
7.18
7.18
6.88
6.87
6.87
6.86
6.86
6.85
6.10
6.10
6.09
6.08
5.79
5.79
5.78
5.76
4.65
4.43
4.42
3.80
3.80
2.66
-0.00
7.25
7.23
7.20
7.20
7.19
7.19
7.18
7.18
1.00
2.18
7.28 7.26 7.24 7.22 7.20 7.18 7.16 7.14
f1 (ppm)
F5S
NH
N
O
3ax, 1H-NMR (500 MHz, CDCl3)
1.13
2.18
2.15
1.06
1.00
0.95
2.19
3.29
1.68
8.5 8.0 7.5 7.0 6.5 6.0 5.5 5.0 4.5 4.0 3.5 3.0 2.5 2.0 1.5 1.0 0.5 0.0 -0.5
f1 (ppm)

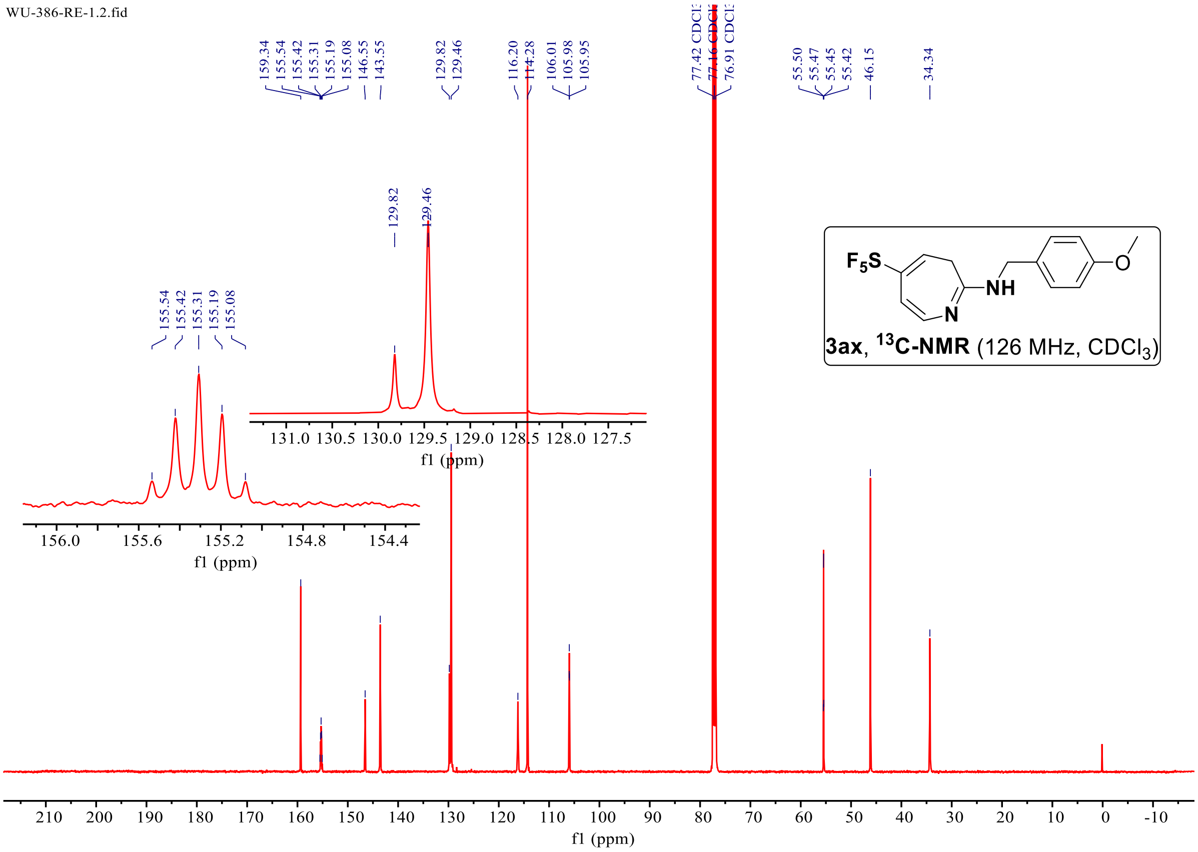

WU-386-RE-1.2.fid
3ax, 13C-NMR (126 MHz, CDCl3)
f1 (ppm)

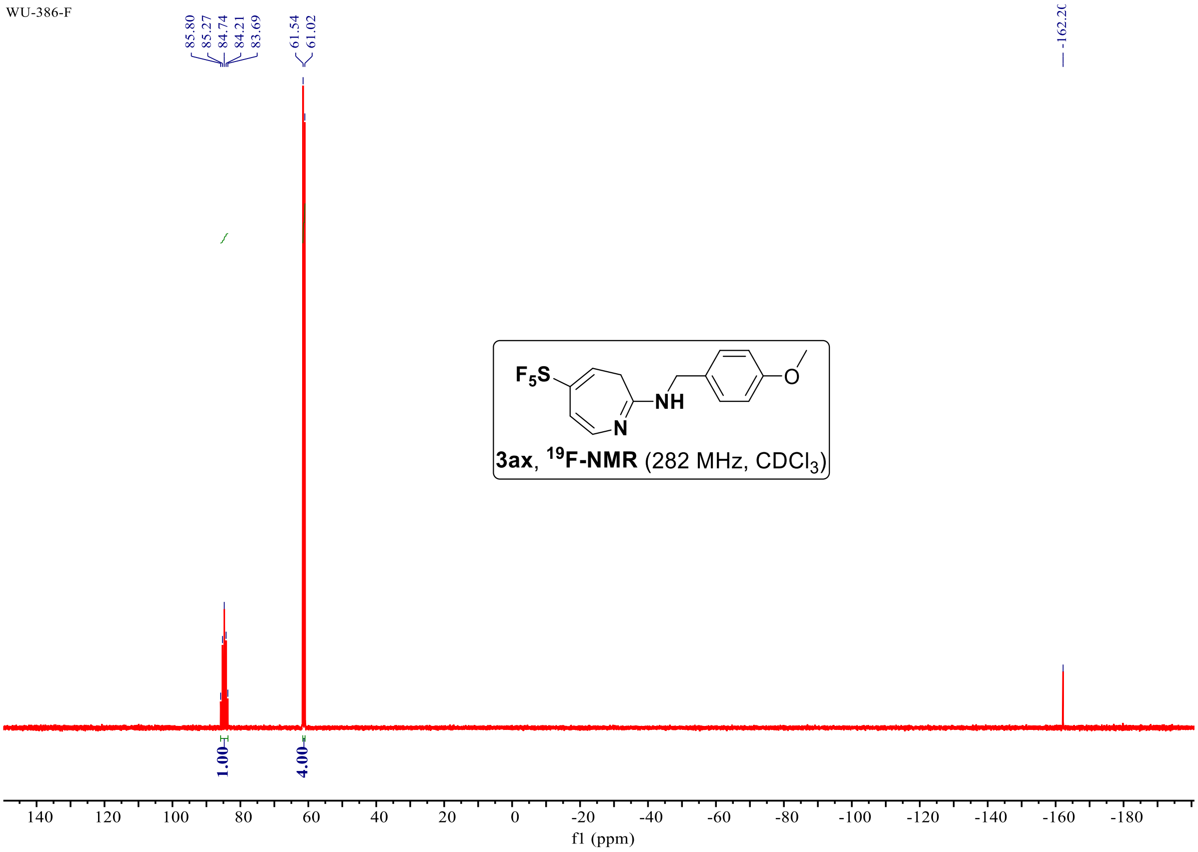

WU-386-F
3ax, 19F-NMR (282 MHz, CDCl3)
f1 (ppm)

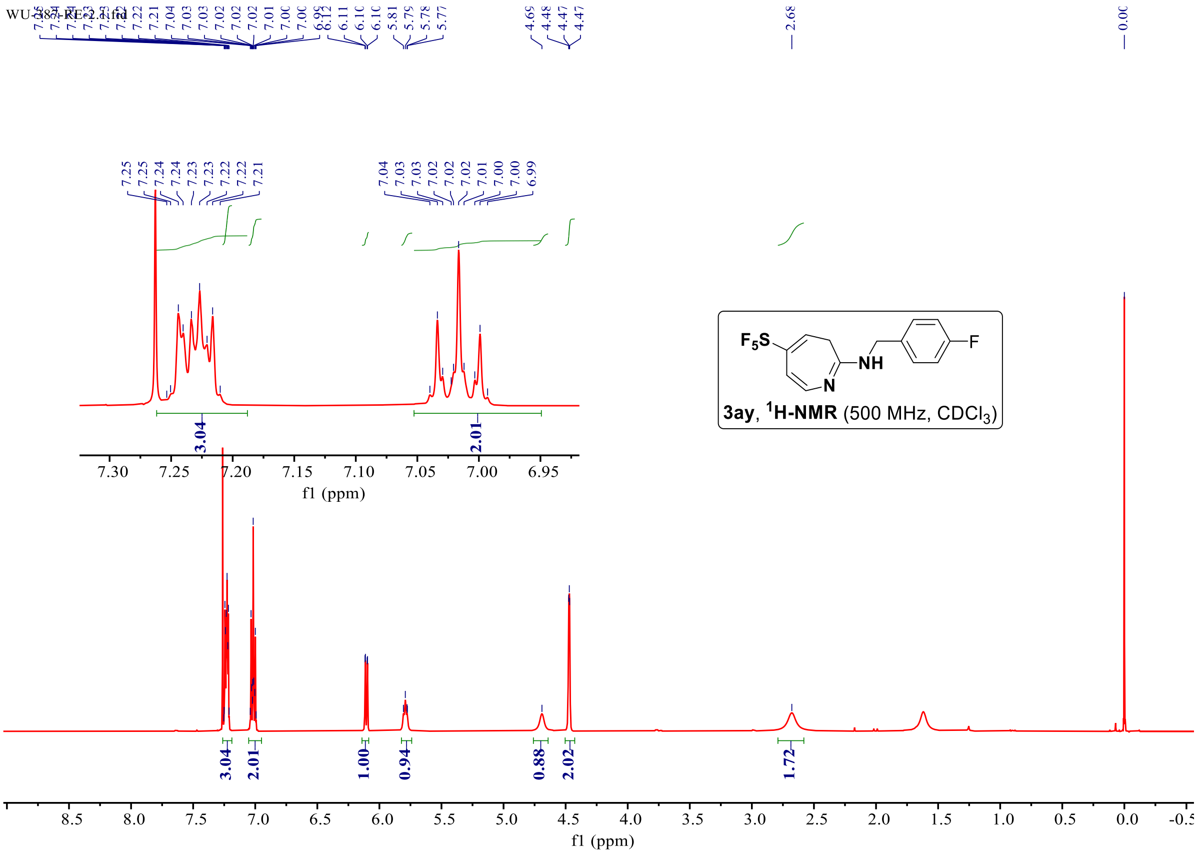

WU-387-RE-2.1.fid
3ay, 1H-NMR (500 MHz, CDCl3)
F5S
NH
F
N
3.04
2.01
1.00
0.94
0.88
2.02
1.72
f1 (ppm)

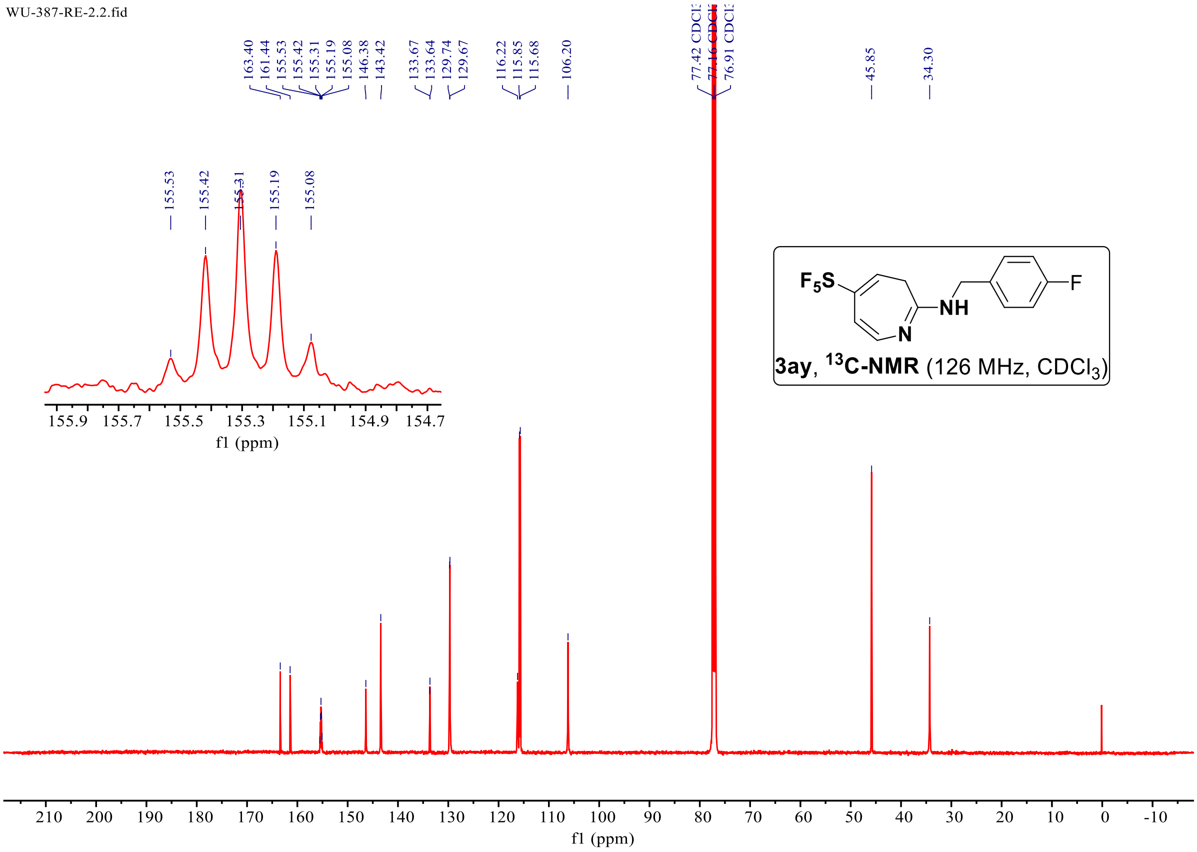

WU-387-RE-2.2.fid
163.40
161.44
155.53
155.42
155.31
155.19
155.08
146.38
143.42
133.67
133.64
129.74
129.67
116.22
115.85
115.68
106.20
45.85
34.30
3ay, 13C-NMR (126 MHz, CDCl3)
F5S
NH
F
N
f1 (ppm)

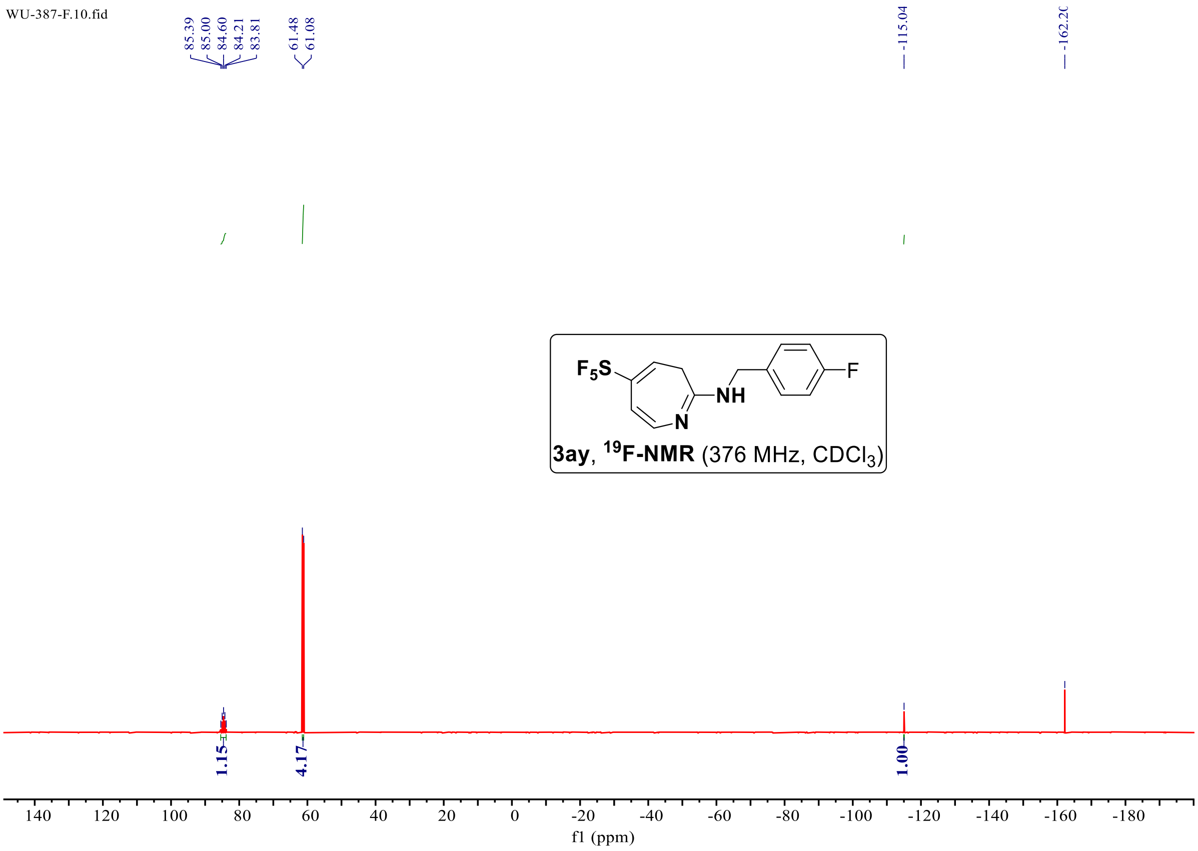
WU-387-F.10.fid
3ay, 19F-NMR (376 MHz, CDCl3)
f1 (ppm)

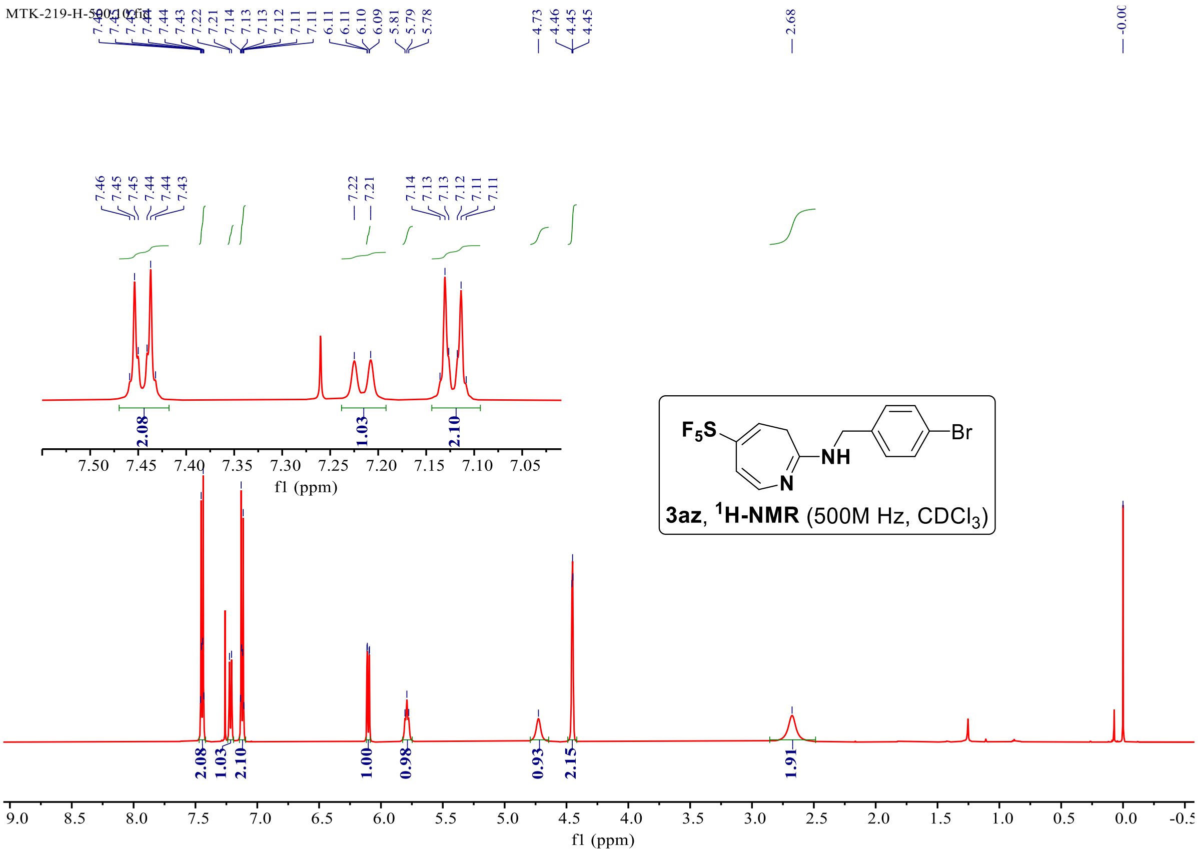
MTK-219-H-500.10.fid
3az, 1H-NMR (500M Hz, CDCl3)
f1 (ppm)

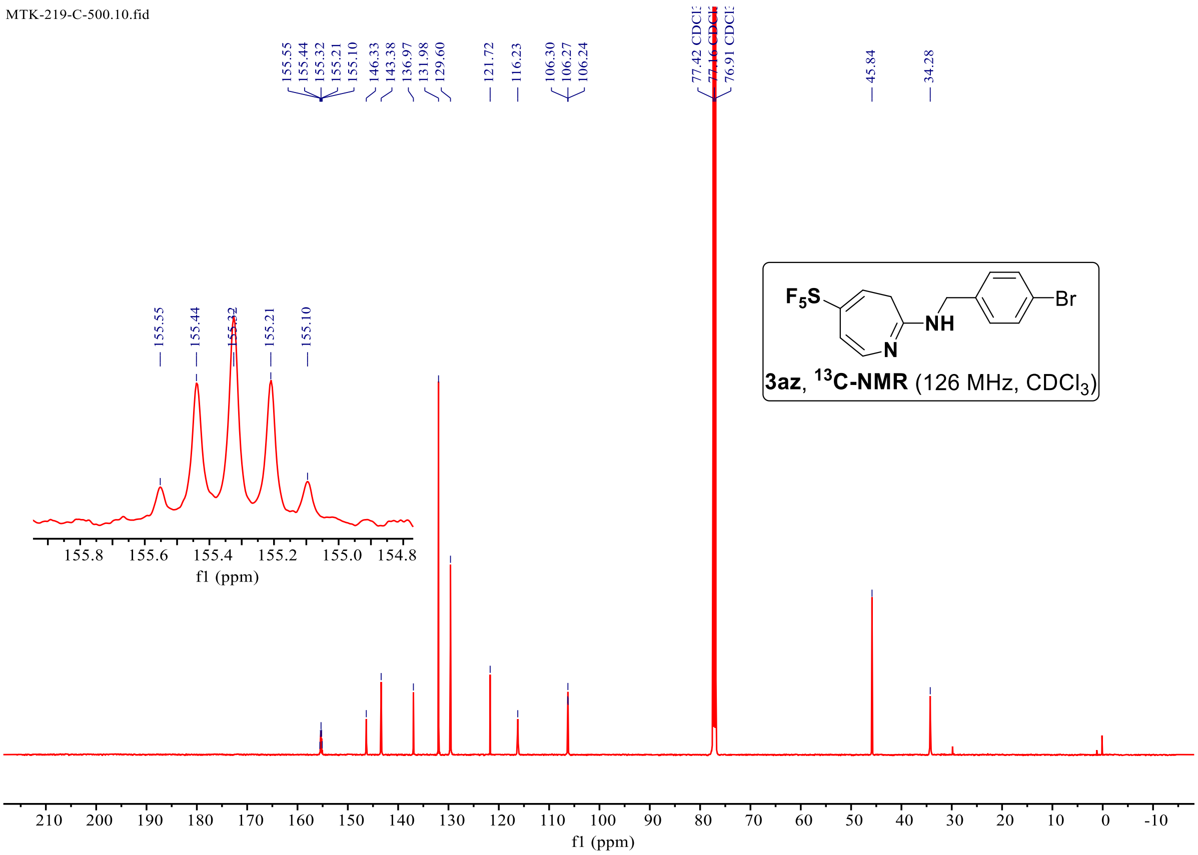
MTK-219-C-500.10.fid
155.55
155.44
155.32
155.21
155.10
146.33
143.38
136.97
131.98
129.60
121.72
116.23
106.30
106.27
106.24
77.42 CDCl3
77.16 CDCl3
76.91 CDCl3
45.84
34.28
3az, 13C-NMR (126 MHz, CDCl3)
f1 (ppm)

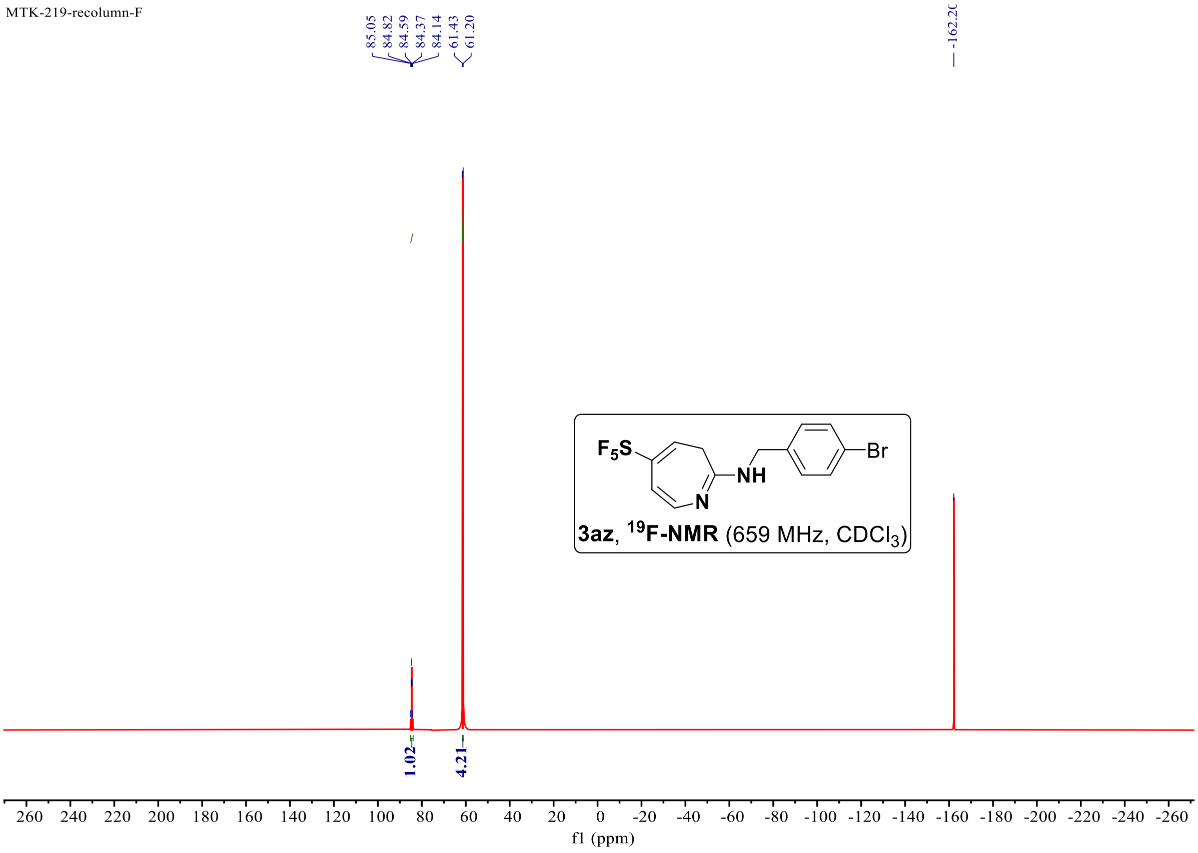
MTK-219-recolumn-F
85.05
84.82
84.59
84.37
84.14
61.43
61.20
-162.20
1.02
4.21
3az, 19F-NMR (659 MHz, CDCl3)
f1 (ppm)

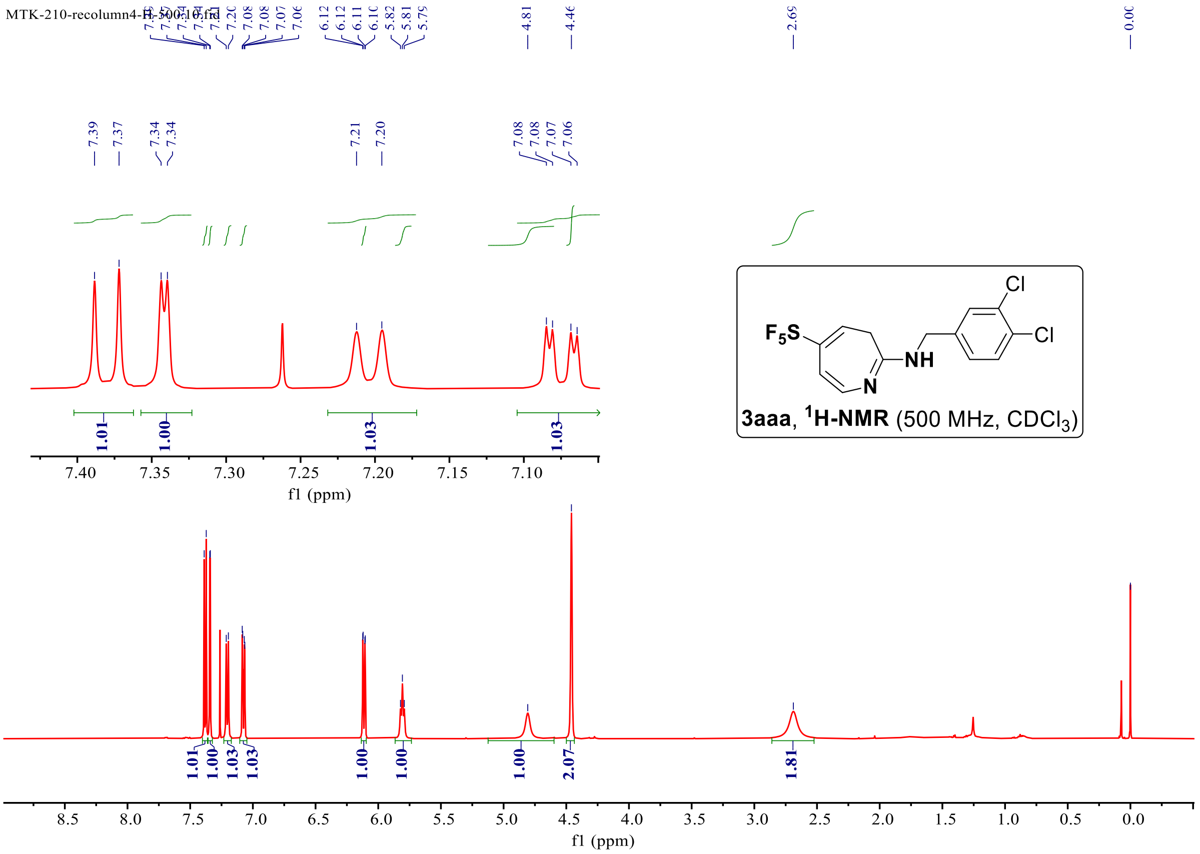
MTK-210-recolumn4-H-500.10.fid
3aaa, ^{1}H-NMR (500 MHz, CDCl$_3$)
f1 (ppm)

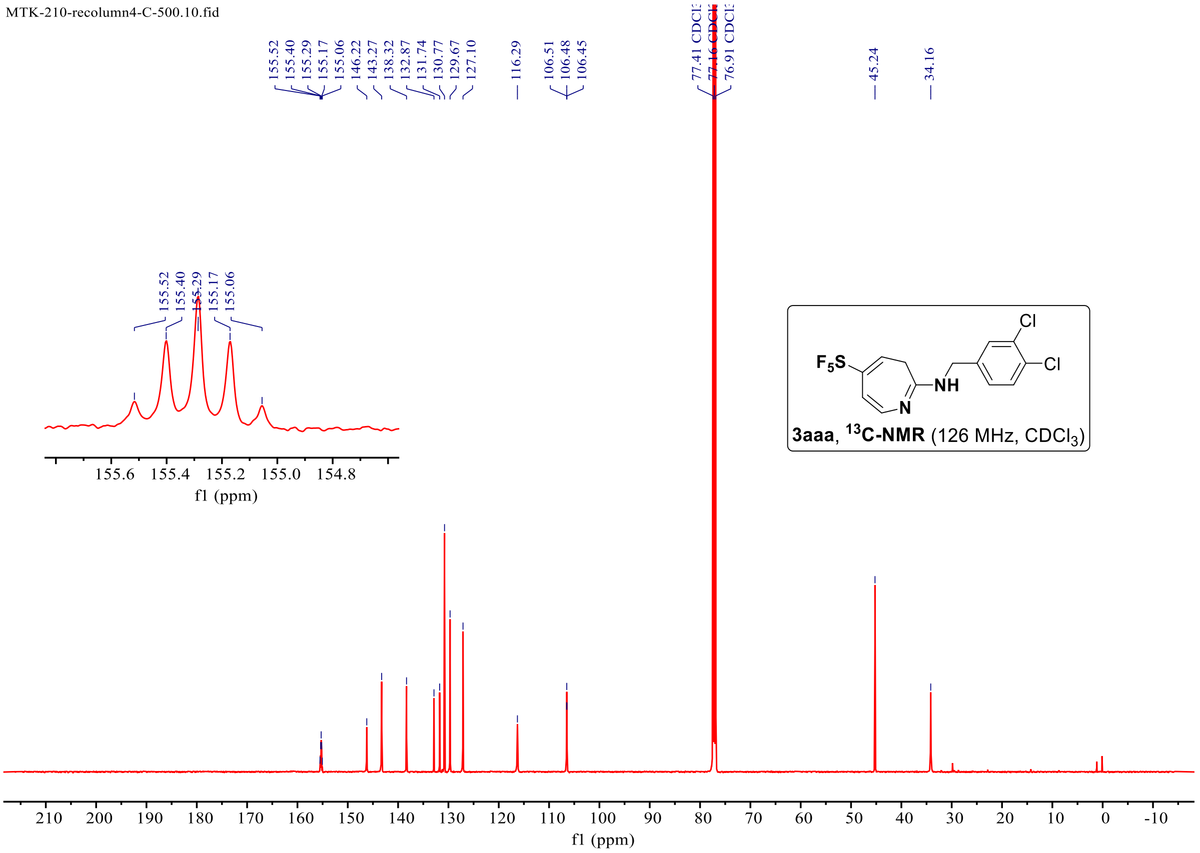
MTK-210-recolumn4-C-500.10.fid
155.52
155.40
155.29
155.17
155.06
146.22
143.27
138.32
132.87
131.74
130.77
129.67
127.10
116.29
106.51
106.48
106.45
45.24
34.16
3aaa, ^{13}C-NMR (126 MHz, CDCl$_3$)
f1 (ppm)

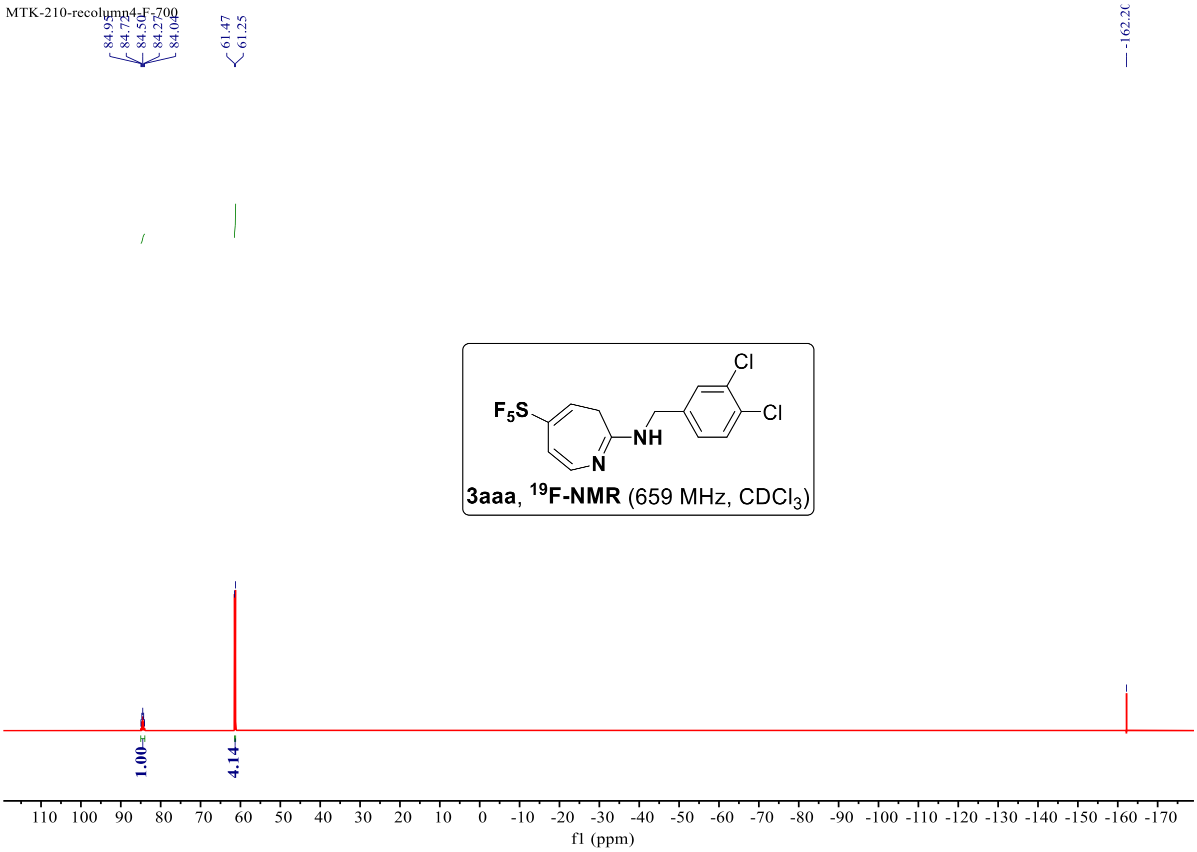
MTK-210-recolumn4-F-700
84.95 84.72 84.50 84.27 84.04
61.47 61.25
-162.20
F5S
Cl
Cl
NH
N
3aaa, 19F-NMR (659 MHz, CDCl3)
1.00
4.14
110 100 90 80 70 60 50 40 30 20 10 0 -10 -20 -30 -40 -50 -60 -70 -80 -90 -100 -110 -120 -130 -140 -150 -160 -170
f1 (ppm)

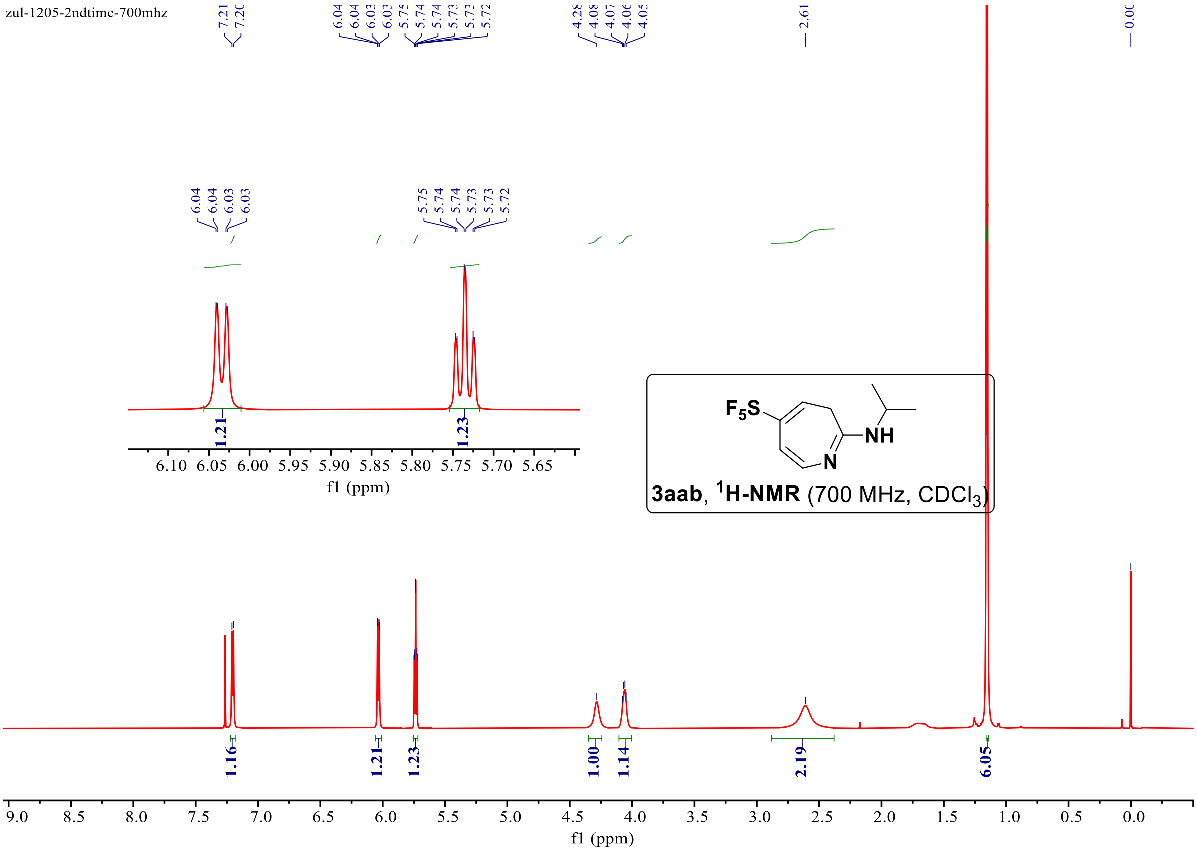
zul-1205-2ndtime-700mhz
7.21 7.20
6.04 6.04 6.03 6.03 5.75 5.74 5.74 5.73 5.73 5.72
4.28 4.08 4.07 4.06 4.05
2.61
0.00
F5S
NH
N
3aab, 1H-NMR (700 MHz, CDCl3)
1.21
1.23
6.10 6.05 6.00 5.95 5.90 5.85 5.80 5.75 5.70 5.65
f1 (ppm)
1.16
1.21
1.23
1.00
1.14
2.19
6.05
9.0 8.5 8.0 7.5 7.0 6.5 6.0 5.5 5.0 4.5 4.0 3.5 3.0 2.5 2.0 1.5 1.0 0.5 0.0
f1 (ppm)

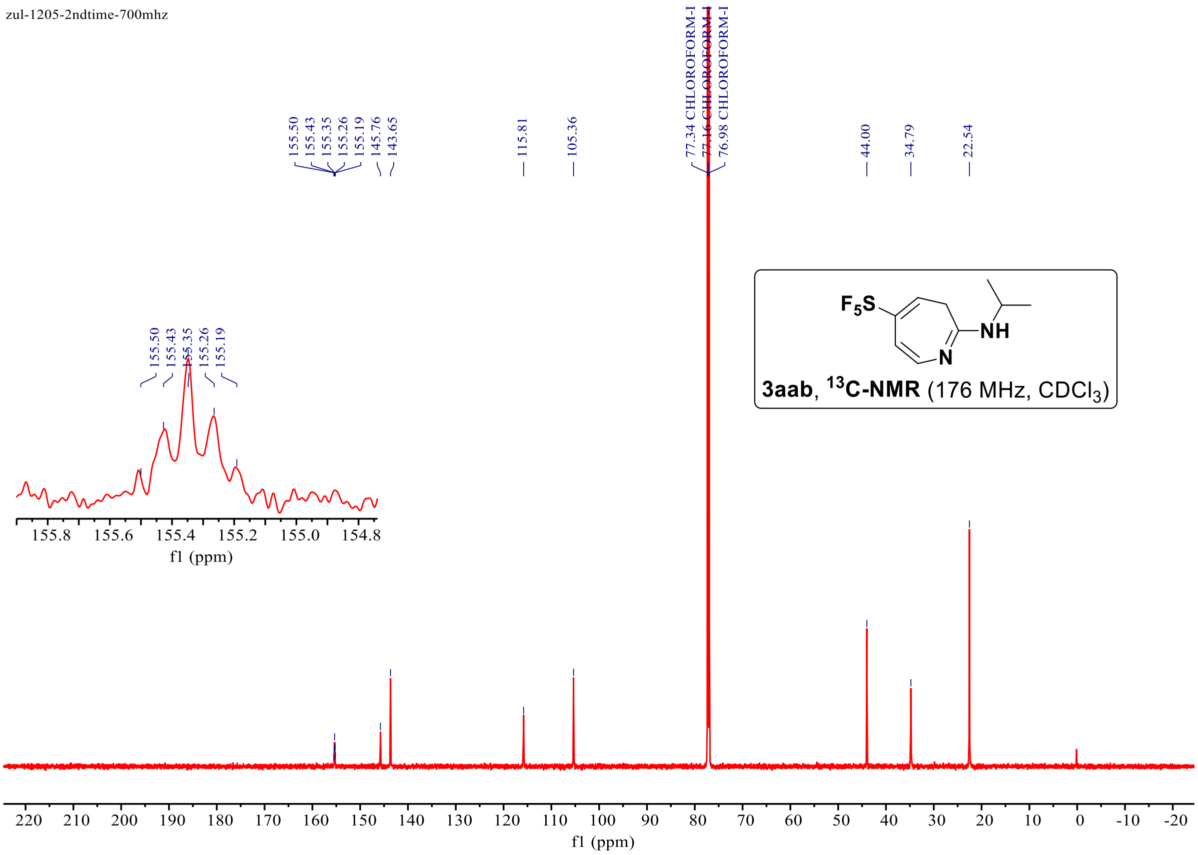
zul-1205-2ndtime-700mhz
155.50
155.43
155.35
155.26
155.19
145.76
143.65
115.81
105.36
77.34 CHLOROFORM-D
77.16 CHLOROFORM-D
76.98 CHLOROFORM-D
44.00
34.79
22.54
3aab, 13C-NMR (176 MHz, CDCl3)
f1 (ppm)

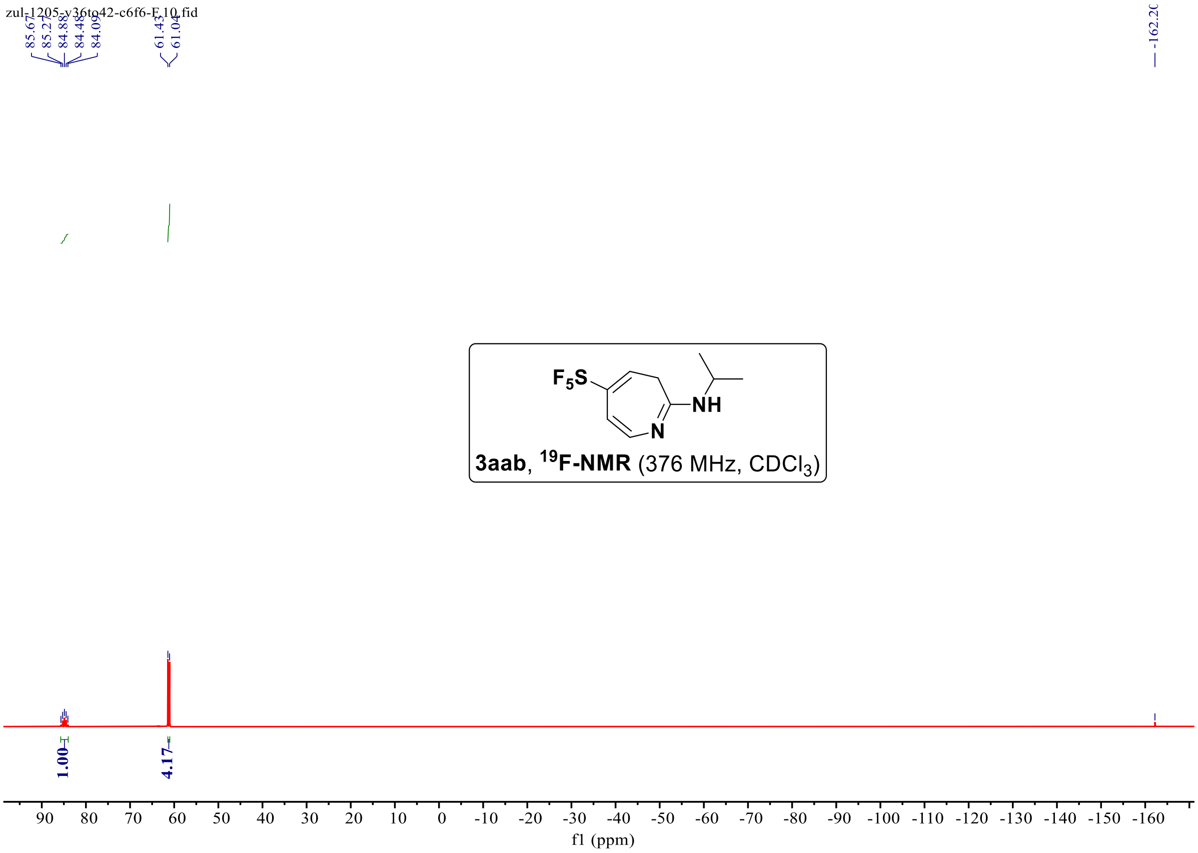
zul-1205-v36to42-c6f6-F.10.fid
85.67
85.27
84.88
84.48
84.09
61.43
61.04
-162.20
3aab, 19F-NMR (376 MHz, CDCl3)
1.00
4.17
f1 (ppm)

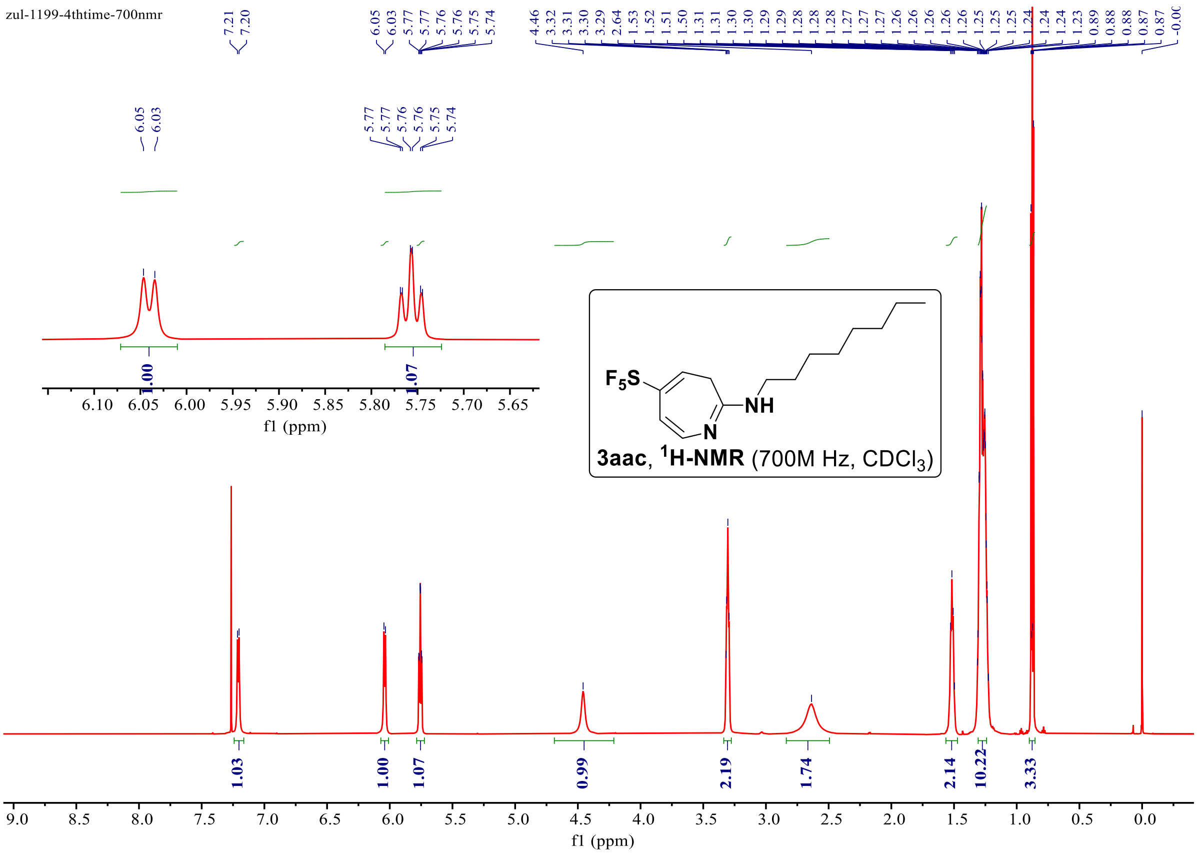
zul-1199-4thtime-700nmr
3aac, ¹H-NMR (700M Hz, CDCl₃)
F₅S
NH
N
f1 (ppm)

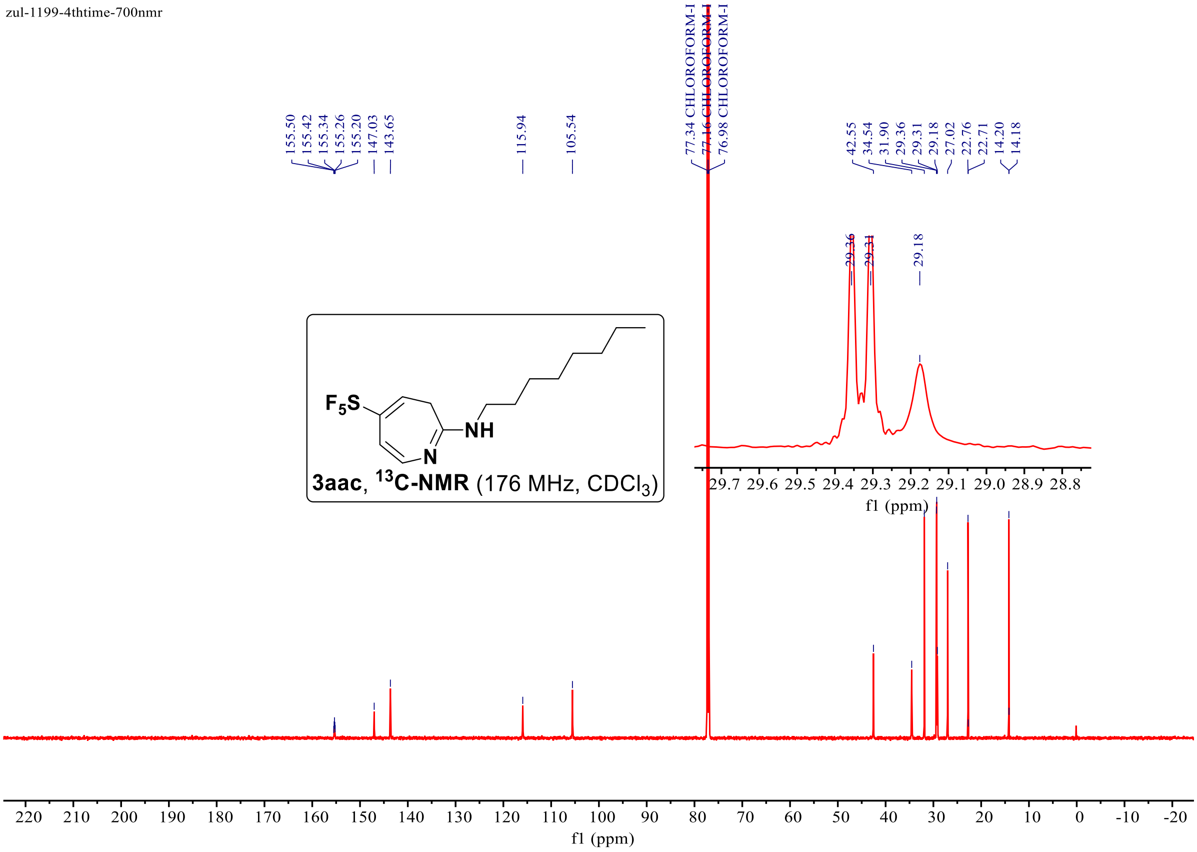
zul-1199-4thtime-700nmr
3aac, ¹³C-NMR (176 MHz, CDCl₃)
F₅S
NH
N
77.34 CHLOROFORM-D
77.16 CHLOROFORM-D
76.98 CHLOROFORM-D
f1 (ppm)

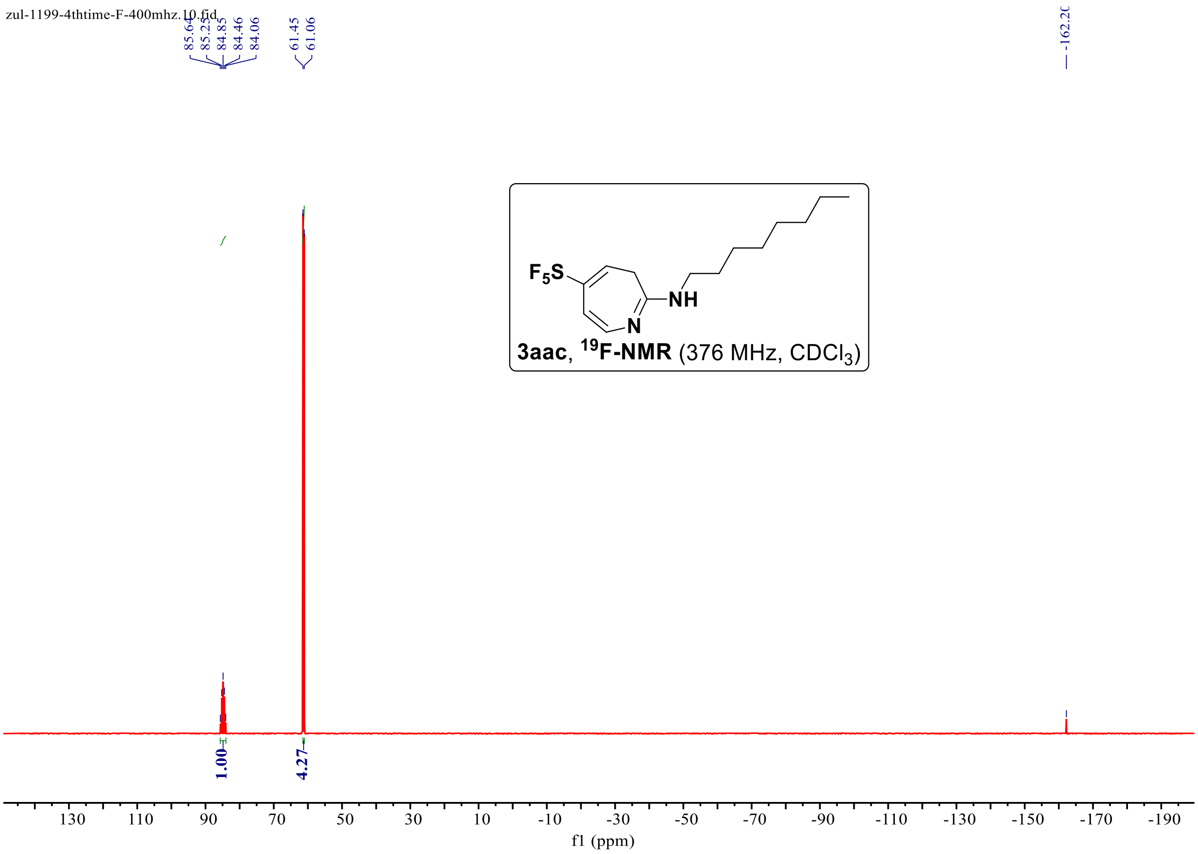
zul-1199-4thtime-F-400mhz.10.fid
85.64
85.23
84.85
84.46
84.06
61.45
61.06
-162.20
F_5S
NH
N
3aac, ^{19}F-NMR (376 MHz, $CDCl_3$)
1.00
4.27
130 110 90 70 50 30 10 -10 -30 -50 -70 -90 -110 -130 -150 -170 -190
f1 (ppm)

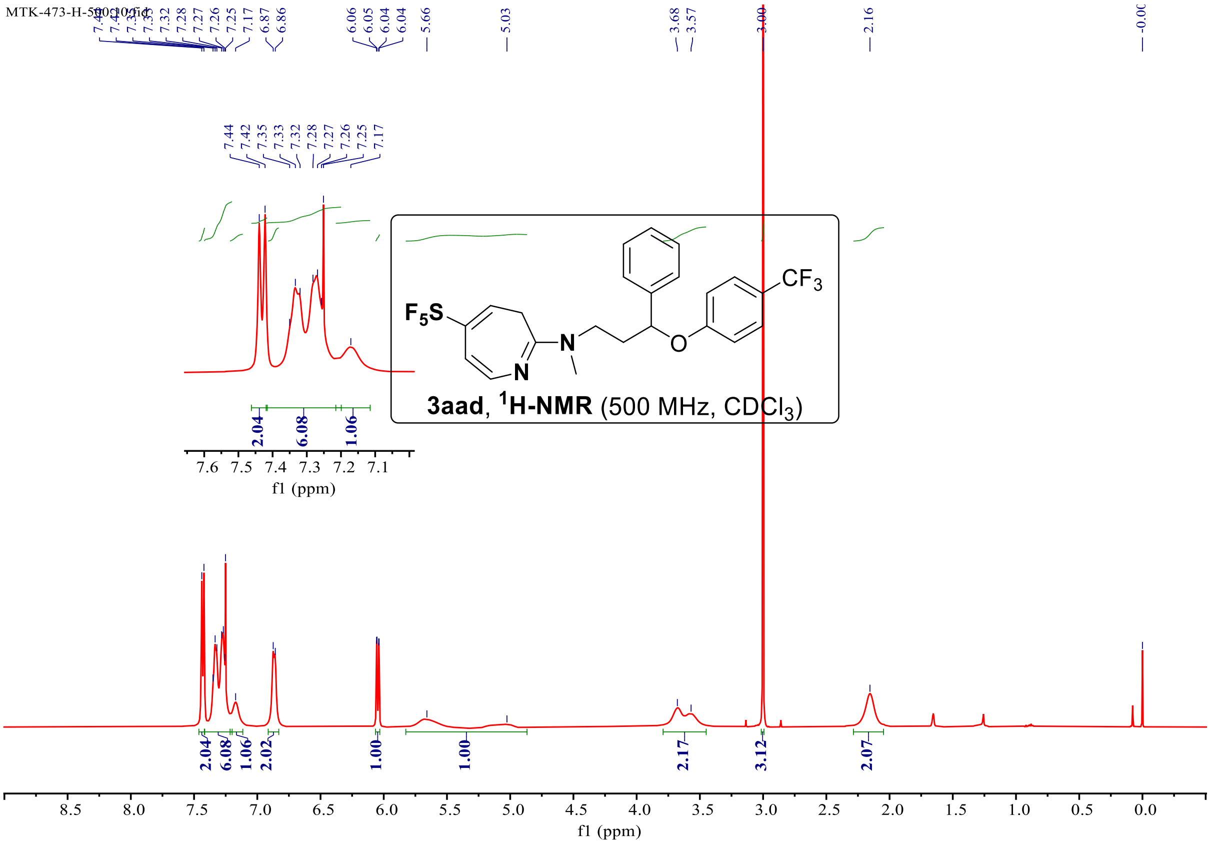
MTK-473-H-500.10.fid
6.87
6.86
6.06
6.05
6.04
6.04
5.66
5.03
3.68
3.57
3.00
2.16
-0.00
7.44
7.42
7.35
7.33
7.32
7.28
7.27
7.26
7.25
7.17
2.04
6.08
1.06
7.6 7.5 7.4 7.3 7.2 7.1
f1 (ppm)
F_5S
N
N
O
CF_3
3aad, ^{1}H-NMR (500 MHz, $CDCl_3$)
2.04
6.08
1.06
2.02
1.00
1.00
2.17
3.12
2.07
8.5 8.0 7.5 7.0 6.5 6.0 5.5 5.0 4.5 4.0 3.5 3.0 2.5 2.0 1.5 1.0 0.5 0.0
f1 (ppm)

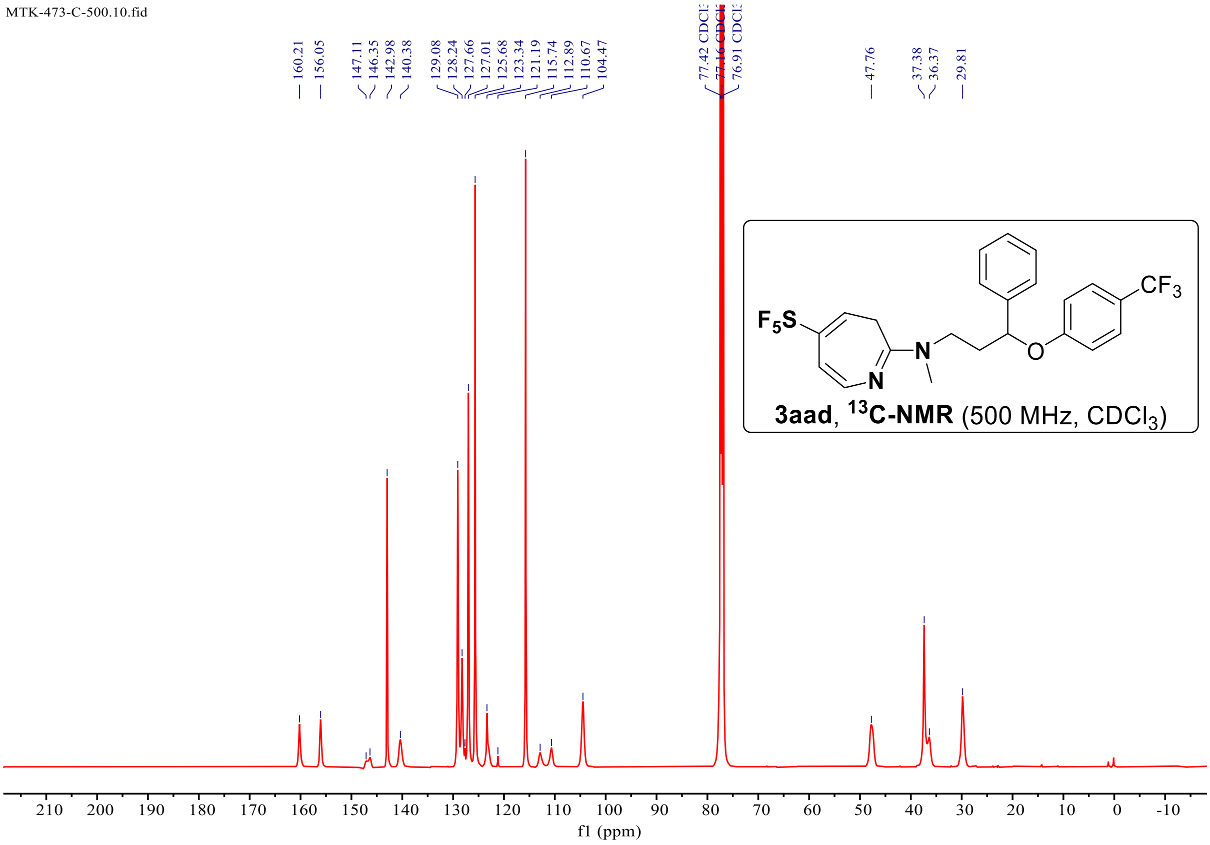
MTK-473-C-500.10.fid
160.21
156.05
147.11
146.35
142.98
140.38
129.08
128.24
127.66
127.01
125.68
123.34
121.19
115.74
112.89
110.67
104.47
77.42 CDCl3
77.16 CDCl3
76.91 CDCl3
47.76
37.38
36.37
29.81
F5S
N
N
O
CF3
3aad, 13C-NMR (500 MHz, CDCl3)
f1 (ppm)

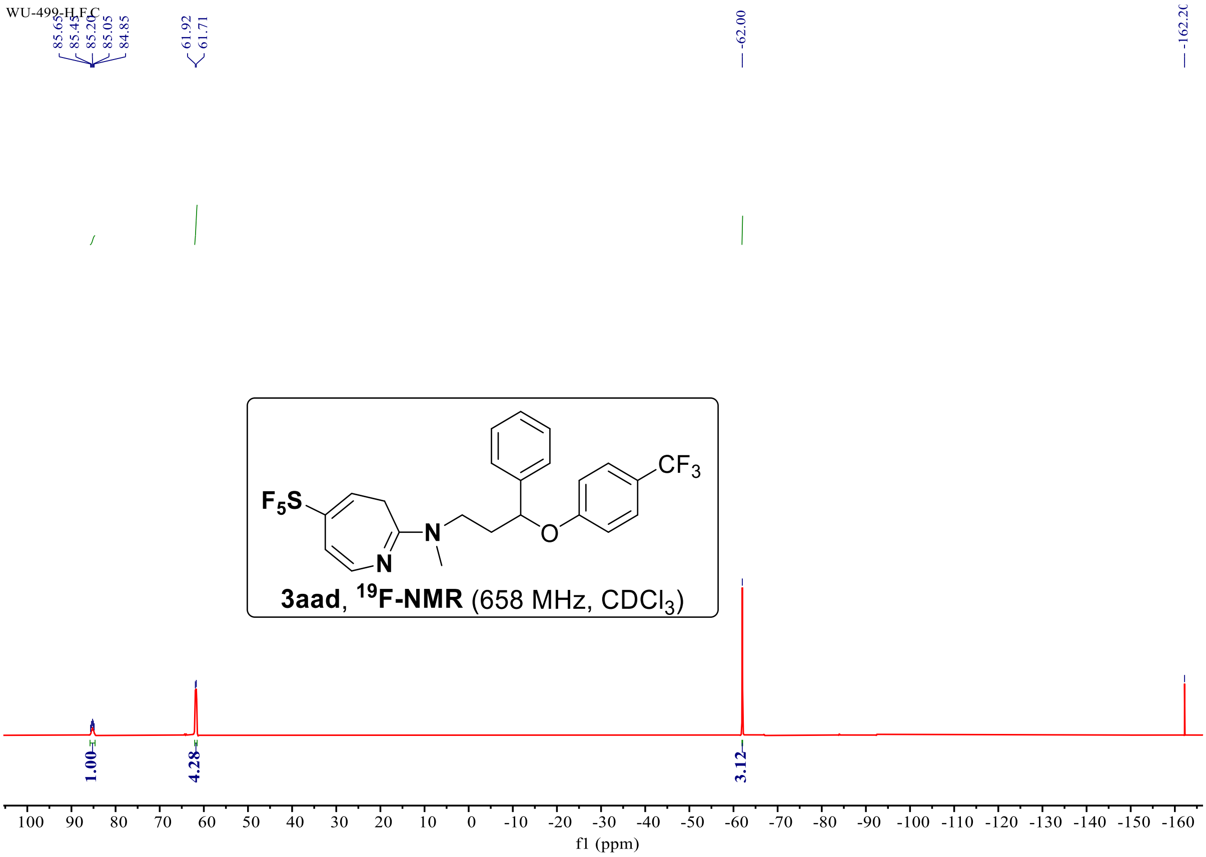
WU-499-H,F,C
85.65
85.45
85.20
85.05
84.85
61.92
61.71
-62.00
-162.20
F5S
N
N
O
CF3
3aad, 19F-NMR (658 MHz, CDCl3)
1.00
4.28
3.12
f1 (ppm)

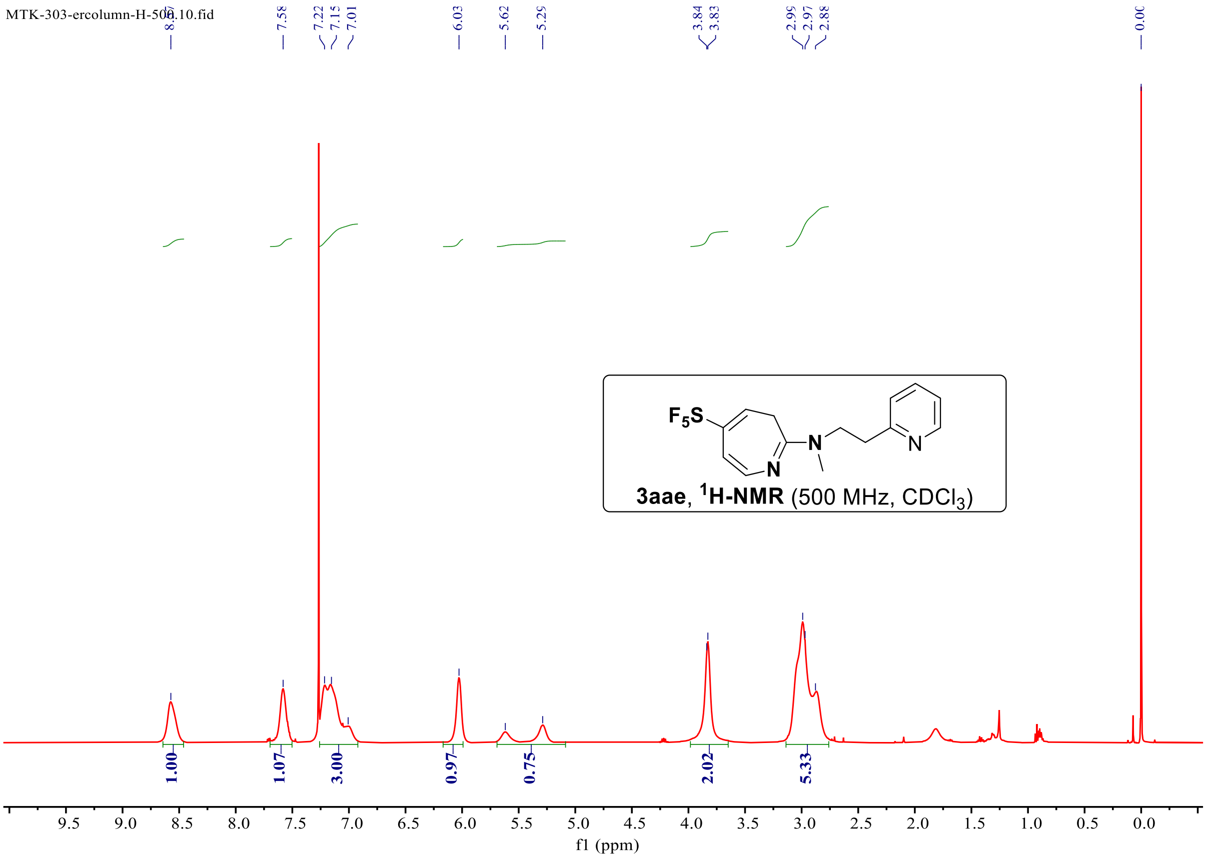
MTK-303-ercolumn-H-500.10.fid
7.58
7.22
7.15
7.01
6.03
5.62
3.84
3.83
2.97
2.88
0.00
F5S
N
N
3aae, 1H-NMR (500 MHz, CDCl3)
1.00
1.07
3.00
0.97
0.75
2.02
5.33
9.5 9.0 8.5 8.0 7.5 7.0 6.5 6.0 5.5 5.0 4.5 4.0 3.5 3.0 2.5 2.0 1.5 1.0 0.5 0.0
f1 (ppm)

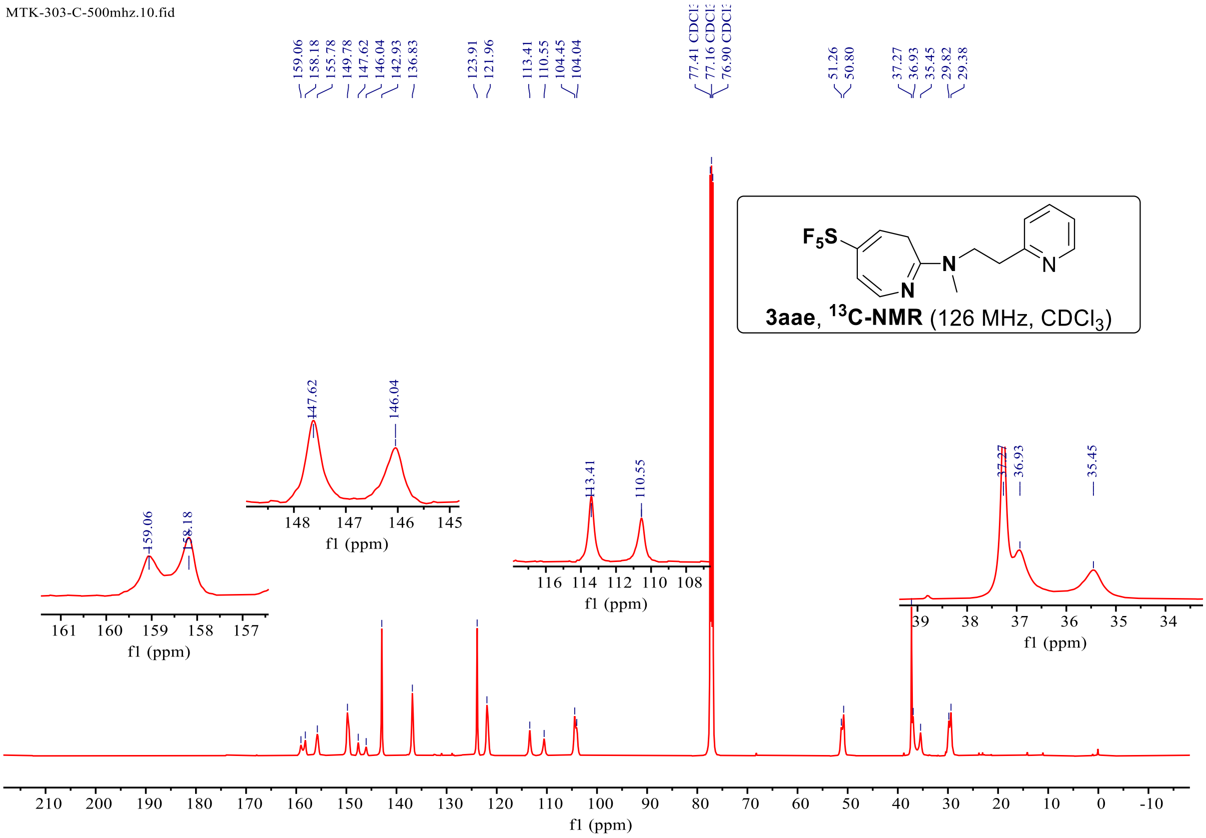
MTK-303-C-500mhz.10.fid
159.06
158.18
155.78
149.78
147.62
146.04
142.93
136.83
123.91
121.96
113.41
110.55
104.45
104.04
51.26
50.80
37.27
36.93
35.45
29.82
29.38
F5S
N
N
3aae, 13C-NMR (126 MHz, CDCl3)
210 200 190 180 170 160 150 140 130 120 110 100 90 80 70 60 50 40 30 20 10 0 -10
f1 (ppm)

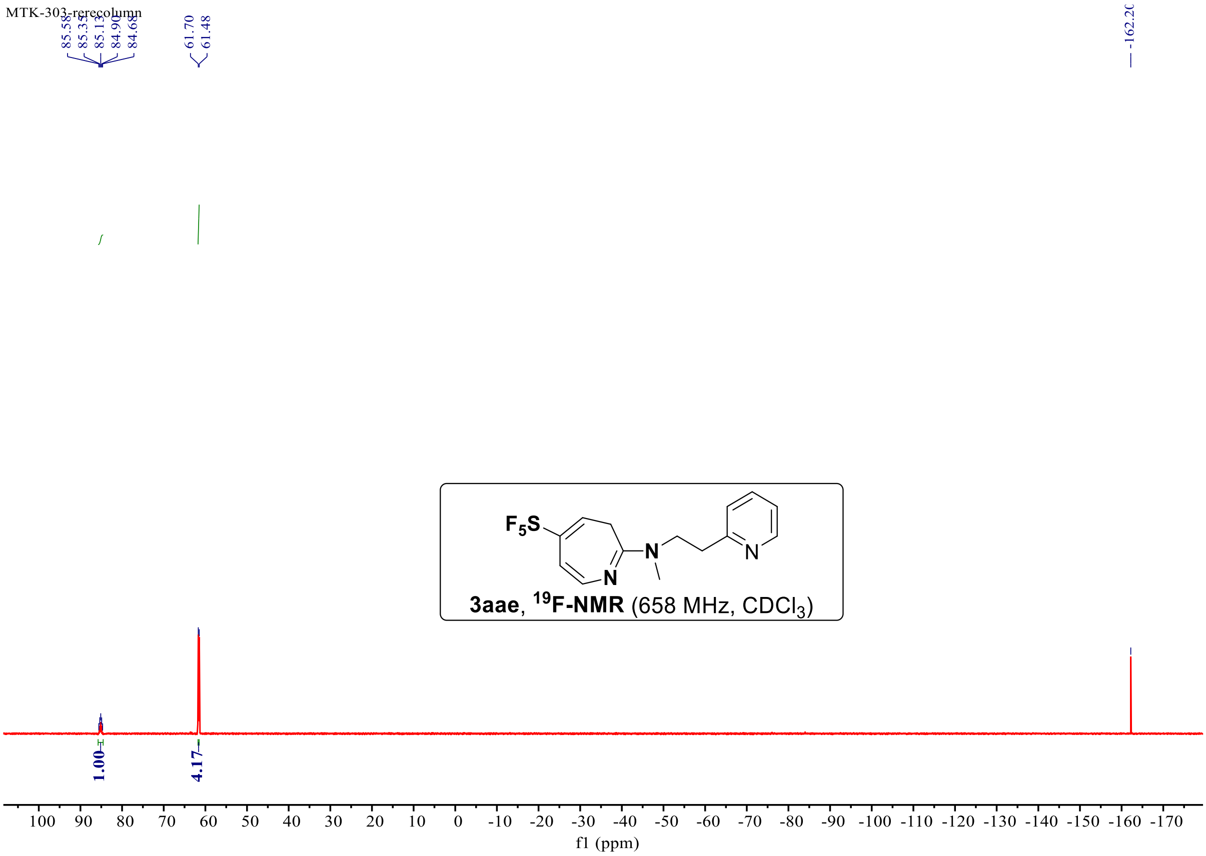
MTK-303-rerecolumn
3aae, ^{19}F-NMR (658 MHz, $CDCl_3$)
f1 (ppm)

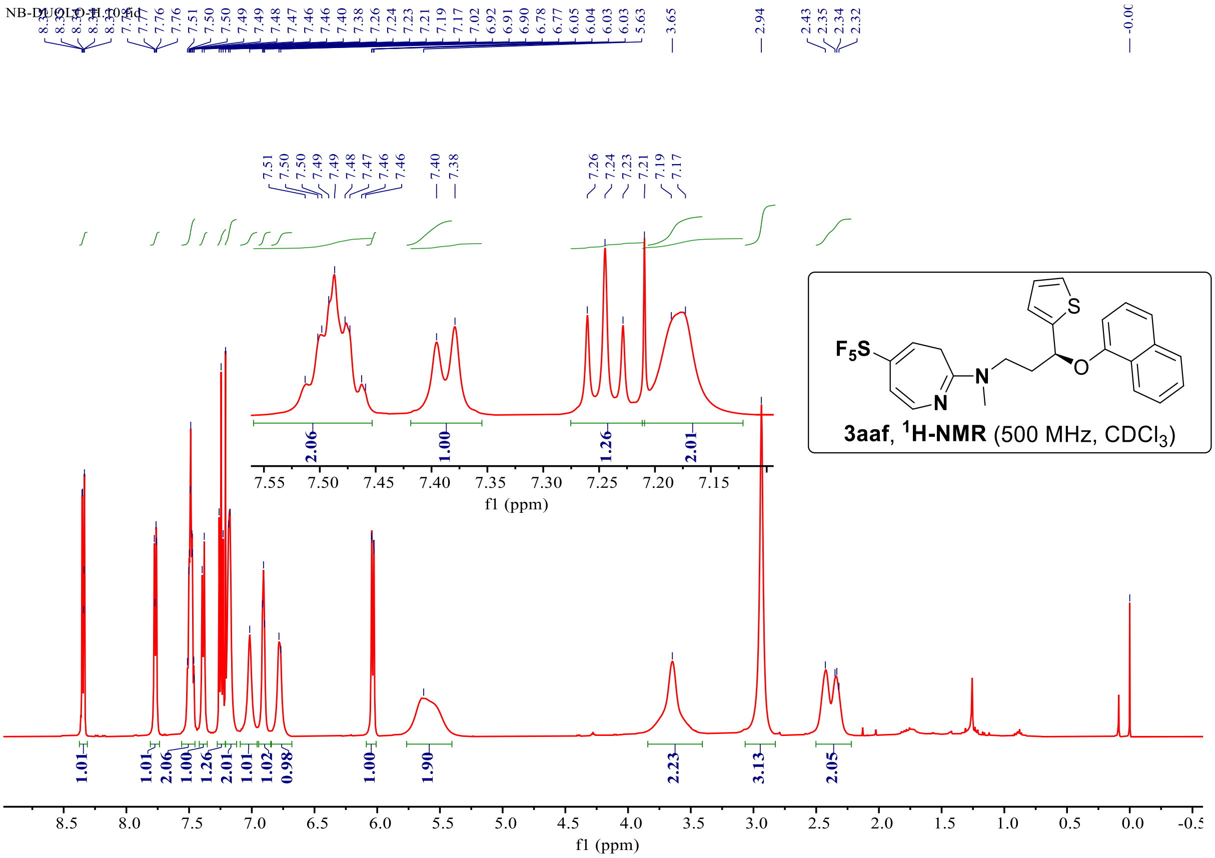
3aaf, ^{1}H-NMR (500 MHz, $CDCl_3$)
f1 (ppm)

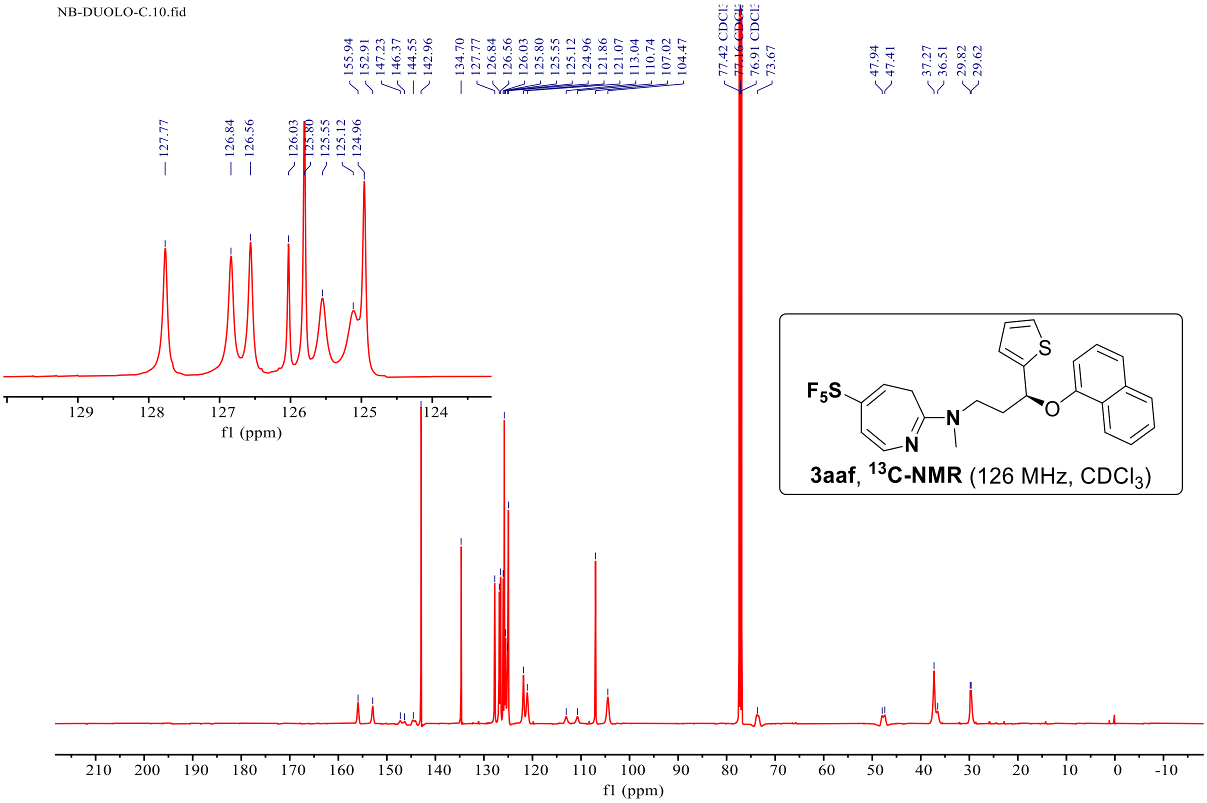
NB-DUOLO-C.10.fid
155.94
152.91
147.23
146.37
144.55
142.96
134.70
127.77
126.84
126.56
126.03
125.80
125.55
125.12
124.96
121.86
121.07
113.04
110.74
107.02
104.47
76.91 CDCl3
73.67
47.94
47.41
37.27
36.51
29.82
29.62
f1 (ppm)
F5S
N
N
O
S
3aaf, 13C-NMR (126 MHz, CDCl3)

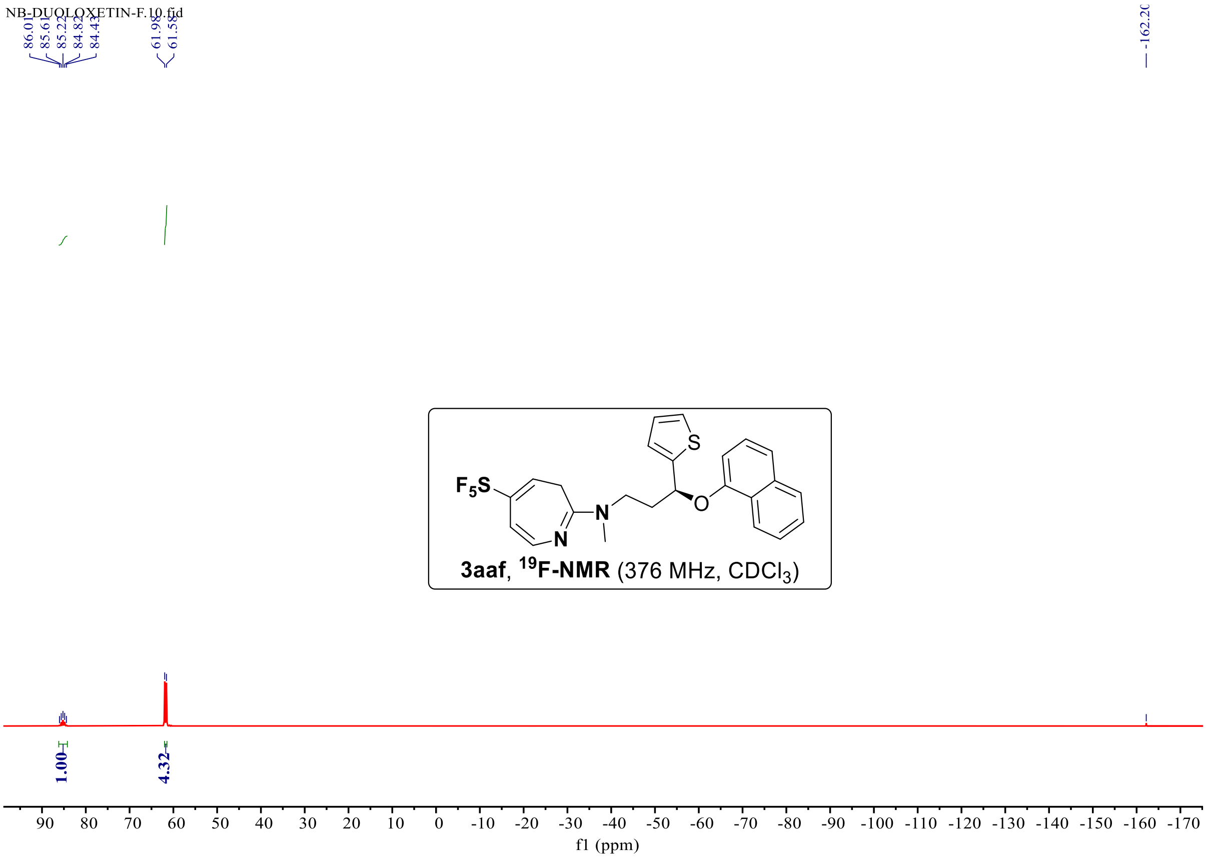
NB-DUOLOXETIN-F.10.fid
84.82
84.43
-162.20
F5S
N
N
O
S
3aaf, 19F-NMR (376 MHz, CDCl3)
1.00
4.32
f1 (ppm)

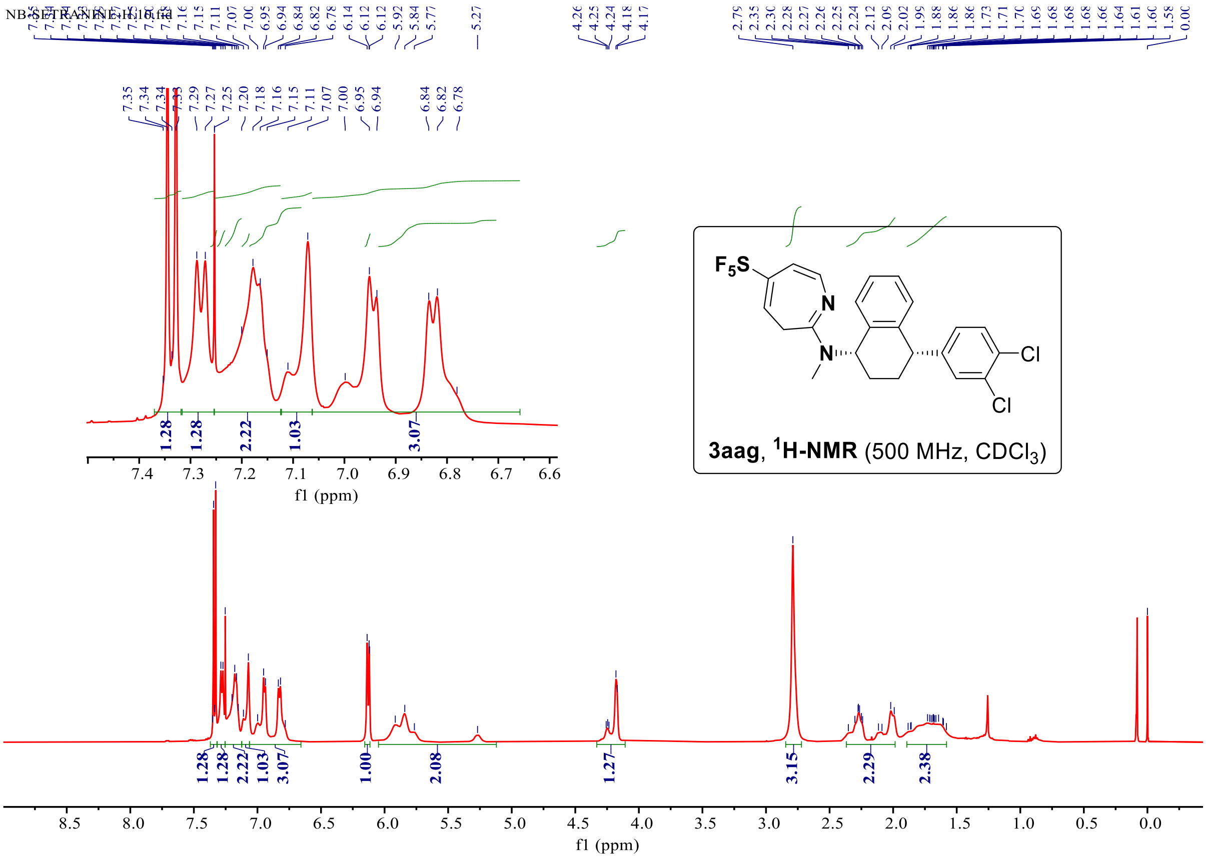
NB-SETRANINE-H.10.fid
7.35
7.34
7.34
7.33
7.29
7.27
7.25
7.20
7.18
7.16
7.15
7.11
7.07
7.00
6.95
6.94
6.84
6.82
6.78
6.14
6.12
6.12
5.92
5.84
5.77
5.27
4.18
4.17
2.79
2.12
2.09
2.02
1.99
1.88
1.86
1.86
1.73
1.71
1.70
1.69
1.68
1.68
1.68
1.66
1.64
1.61
1.60
1.58
0.00
1.28
1.28
2.22
1.03
3.07
1.00
2.08
1.27
3.15
2.29
2.38
f1 (ppm)
F5S
N
N
Cl
Cl
3aag, 1H-NMR (500 MHz, CDCl3)

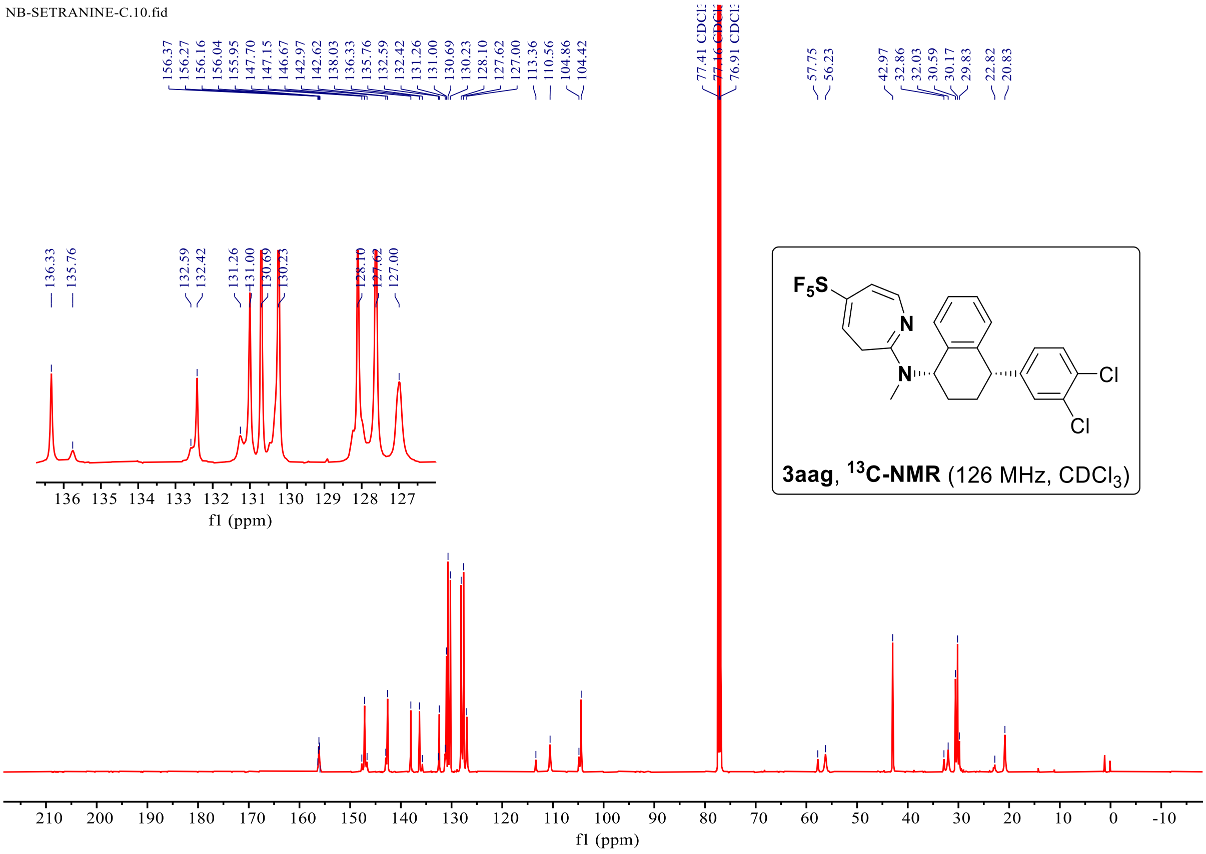
NB-SETRANINE-C.10.fid
156.37
156.27
156.16
156.04
155.95
147.70
147.15
146.67
142.97
142.62
138.03
136.33
135.76
132.59
132.42
131.26
131.00
130.69
130.23
128.10
127.62
127.00
113.36
110.56
104.86
104.42
77.41 CDCl3
77.16 CDCl3
76.91 CDCl3
57.75
56.23
42.97
32.86
32.03
30.59
30.17
29.83
22.82
20.83
f1 (ppm)
F5S
N
N
Cl
Cl
3aag, 13C-NMR (126 MHz, CDCl3)

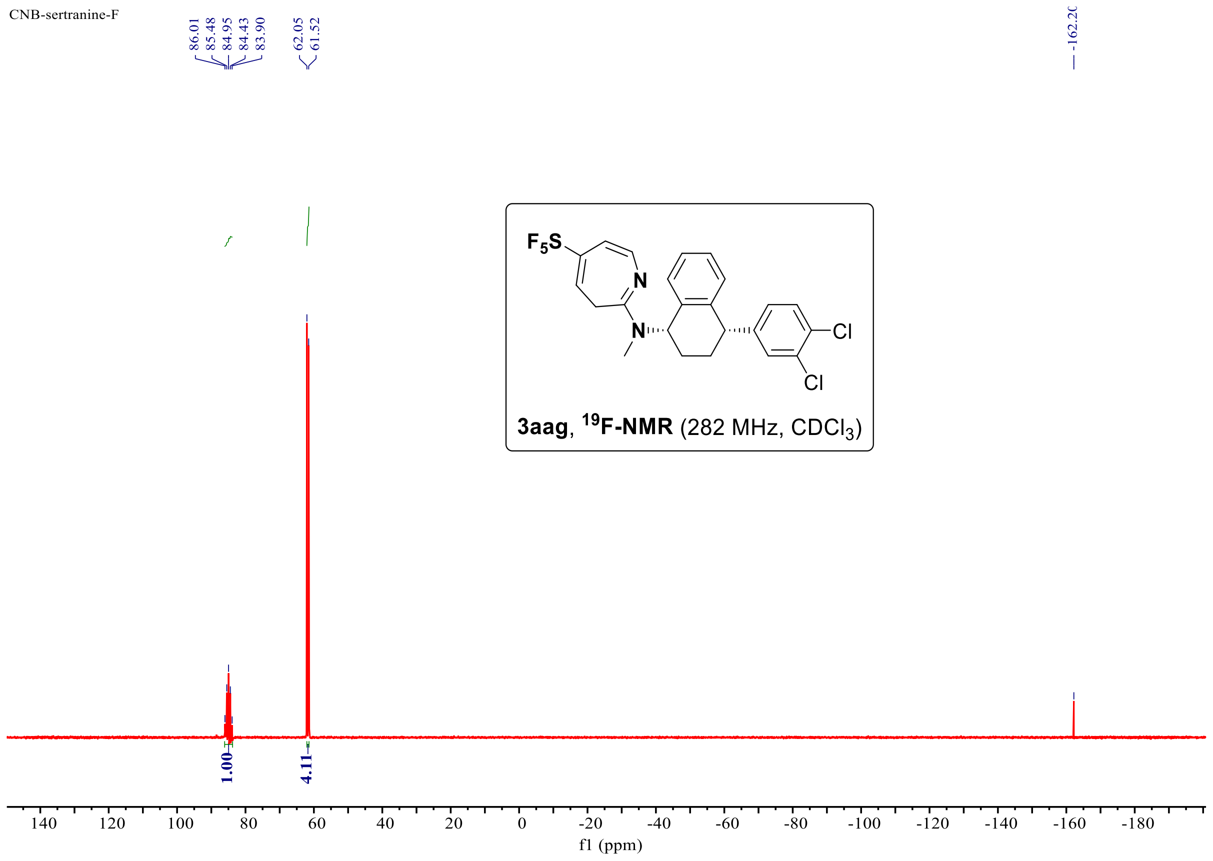
CNB-sertranine-F
86.01
85.48
84.95
84.43
83.90
62.05
61.52
-162.20
F_5S
N
Cl
Cl
3aag, ^{19}F-NMR (282 MHz, $CDCl_3$)
1.00
4.11
140 120 100 80 60 40 20 0 -20 -40 -60 -80 -100 -120 -140 -160 -180
f1 (ppm)

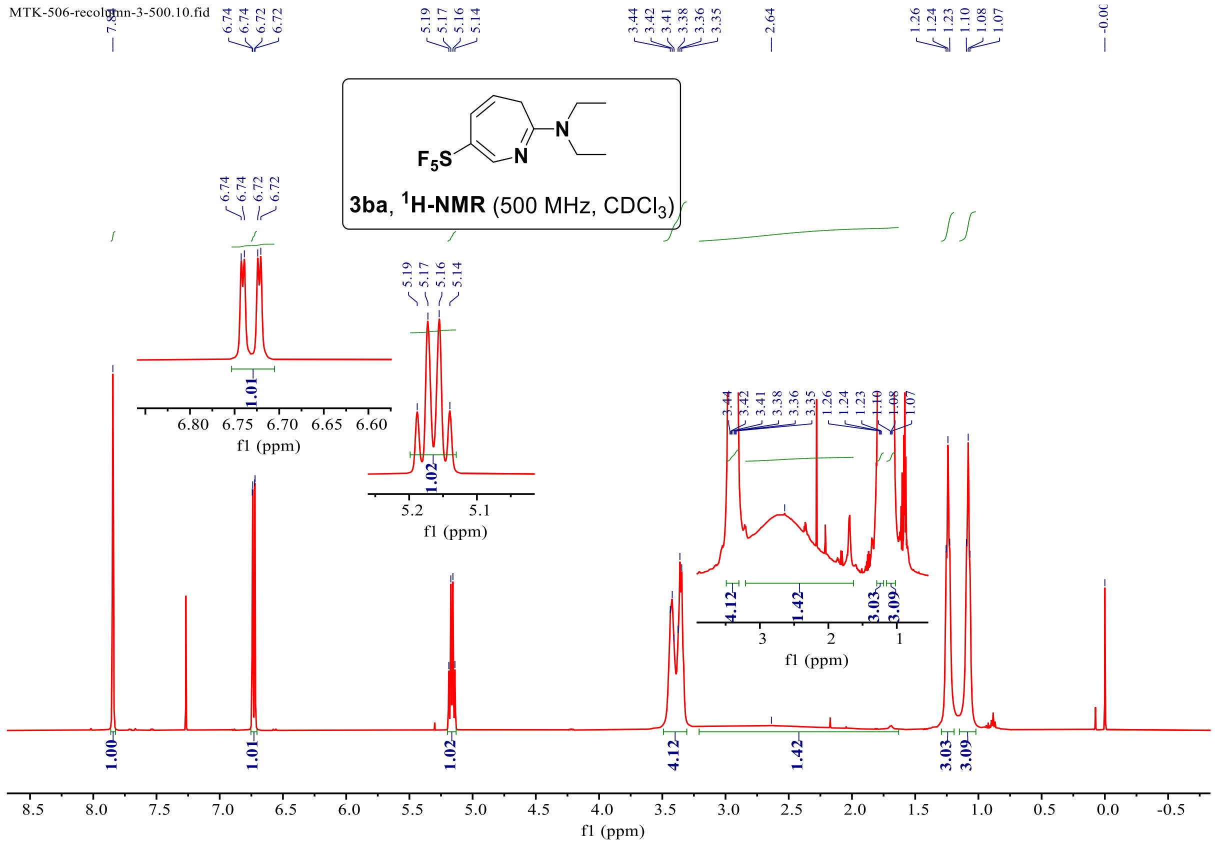
MTK-506-recolumn-3-500.10.fid
7.85
6.74
6.74
6.72
6.72
5.19
5.17
5.16
5.14
3.44
3.42
3.41
3.38
3.36
3.35
2.64
1.26
1.24
1.23
1.10
1.08
1.07
-0.00
F_5S
N
N
3ba, ^{1}H-NMR (500 MHz, $CDCl_3$)
1.00
1.01
1.02
4.12
1.42
3.03
3.09
8.5 8.0 7.5 7.0 6.5 6.0 5.5 5.0 4.5 4.0 3.5 3.0 2.5 2.0 1.5 1.0 0.5 0.0 -0.5
f1 (ppm)

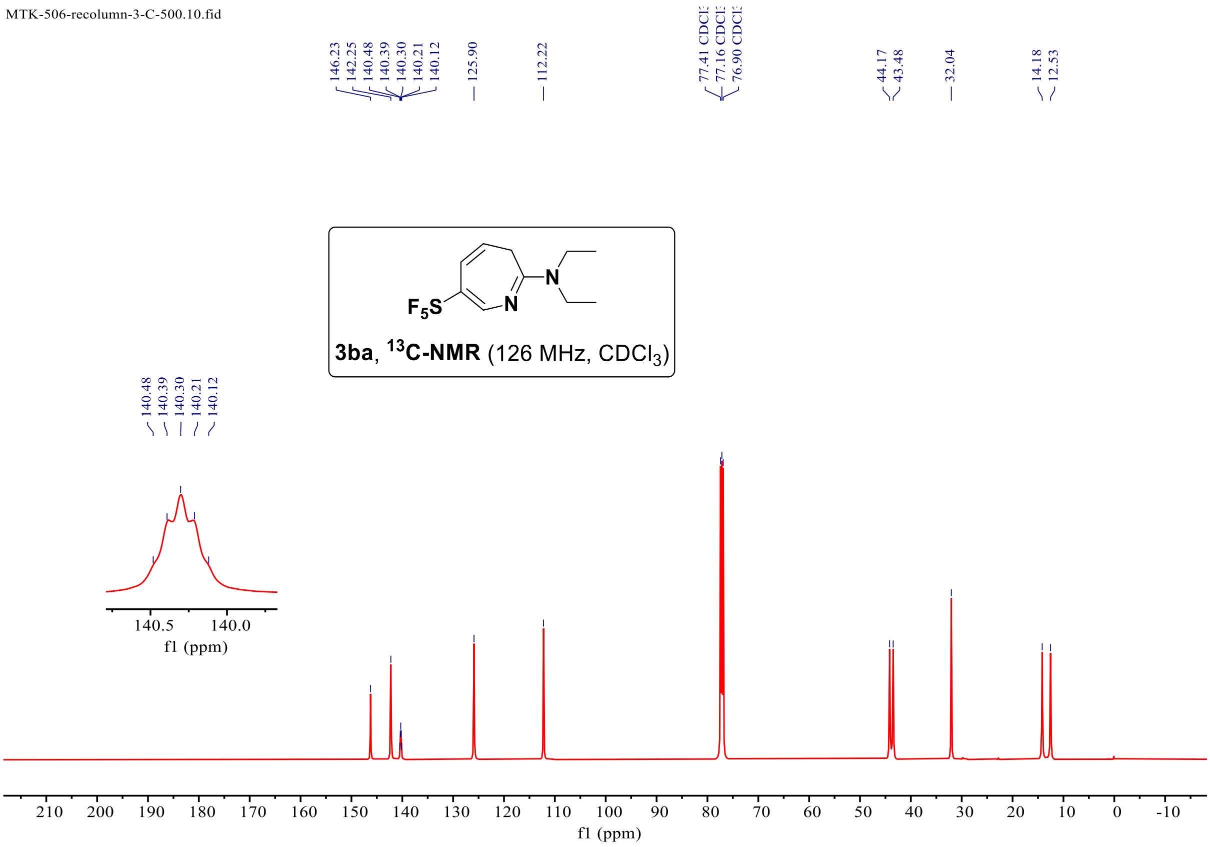
MTK-506-recolumn-3-C-500.10.fid
146.23
142.25
140.48
140.39
140.30
140.21
140.12
125.90
112.22
77.41 CDCl3
77.16 CDCl3
76.90 CDCl3
44.17
43.48
32.04
14.18
12.53
F5S
N
N
3ba, 13C-NMR (126 MHz, CDCl3)
140.48
140.39
140.30
140.21
140.12
140.5
140.0
f1 (ppm)
210 200 190 180 170 160 150 140 130 120 110 100 90 80 70 60 50 40 30 20 10 0 -10
f1 (ppm)

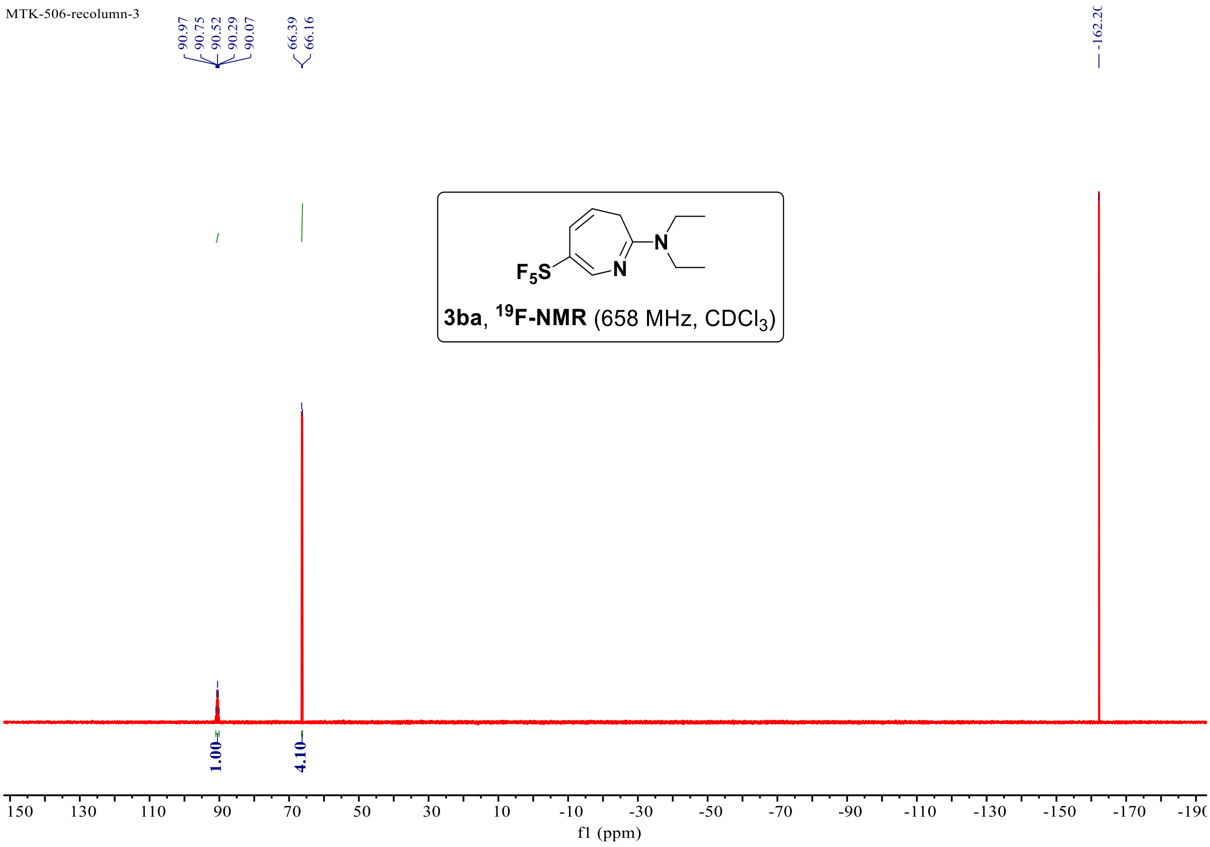
MTK-506-recolumn-3
90.97
90.75
90.52
90.29
90.07
66.39
66.16
-162.20
F5S
N
N
3ba, 19F-NMR (658 MHz, CDCl3)
1.00
4.10
150 130 110 90 70 50 30 10 -10 -30 -50 -70 -90 -110 -130 -150 -170 -190
f1 (ppm)

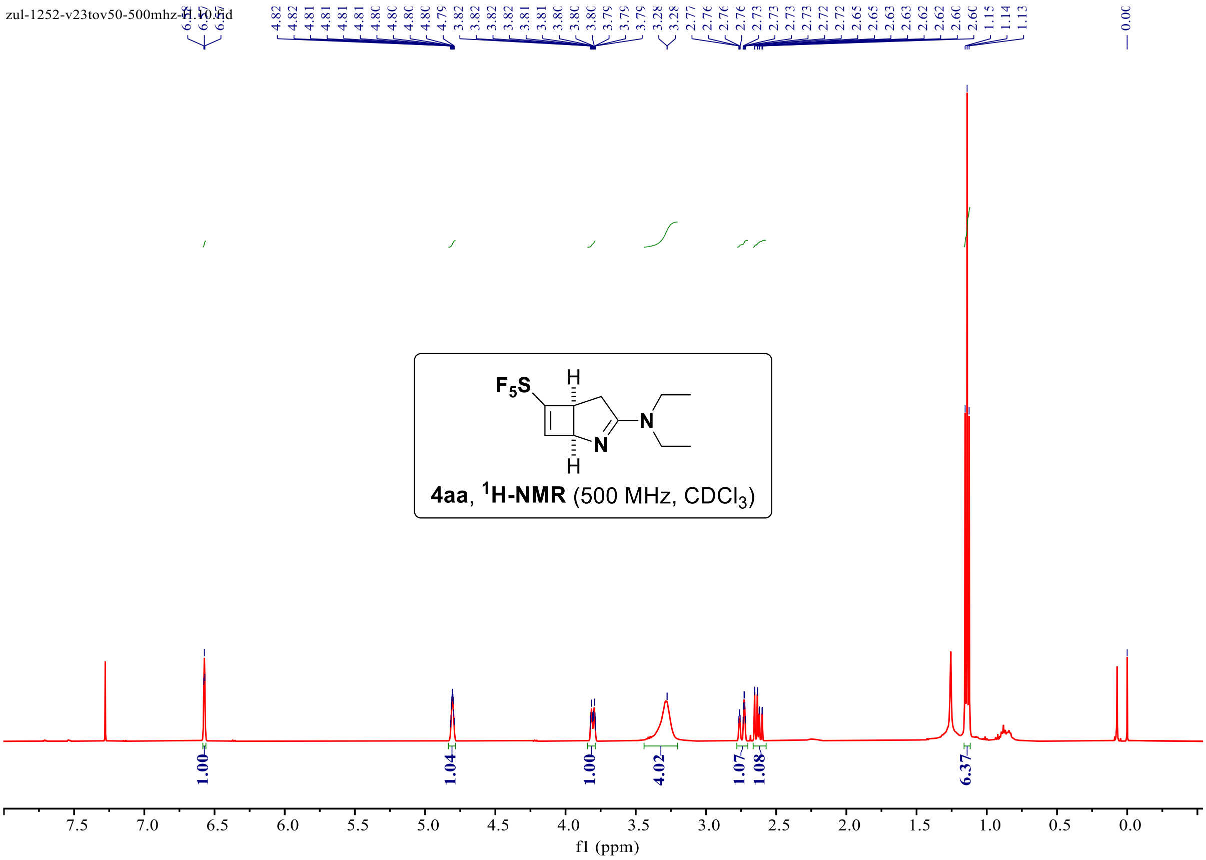
zul-1252-v23tov50-500mhz-H.10.fid
4.82 4.82 4.81 4.81 4.81 4.81 4.80 4.80 4.80 4.80 4.79
3.82 3.82 3.82 3.81 3.81 3.81 3.80 3.80 3.80 3.79 3.79 3.79
3.28 3.28
2.77 2.76 2.76 2.76 2.73 2.73 2.73 2.72 2.72 2.72 2.65 2.65 2.63 2.63 2.62 2.62 2.60 2.60 1.15 1.14 1.13
0.00
F5S
H
N
N
H
4aa, 1H-NMR (500 MHz, CDCl3)
1.00
1.04
1.00
4.02
1.07
1.08
6.37
7.5 7.0 6.5 6.0 5.5 5.0 4.5 4.0 3.5 3.0 2.5 2.0 1.5 1.0 0.5 0.0
f1 (ppm)

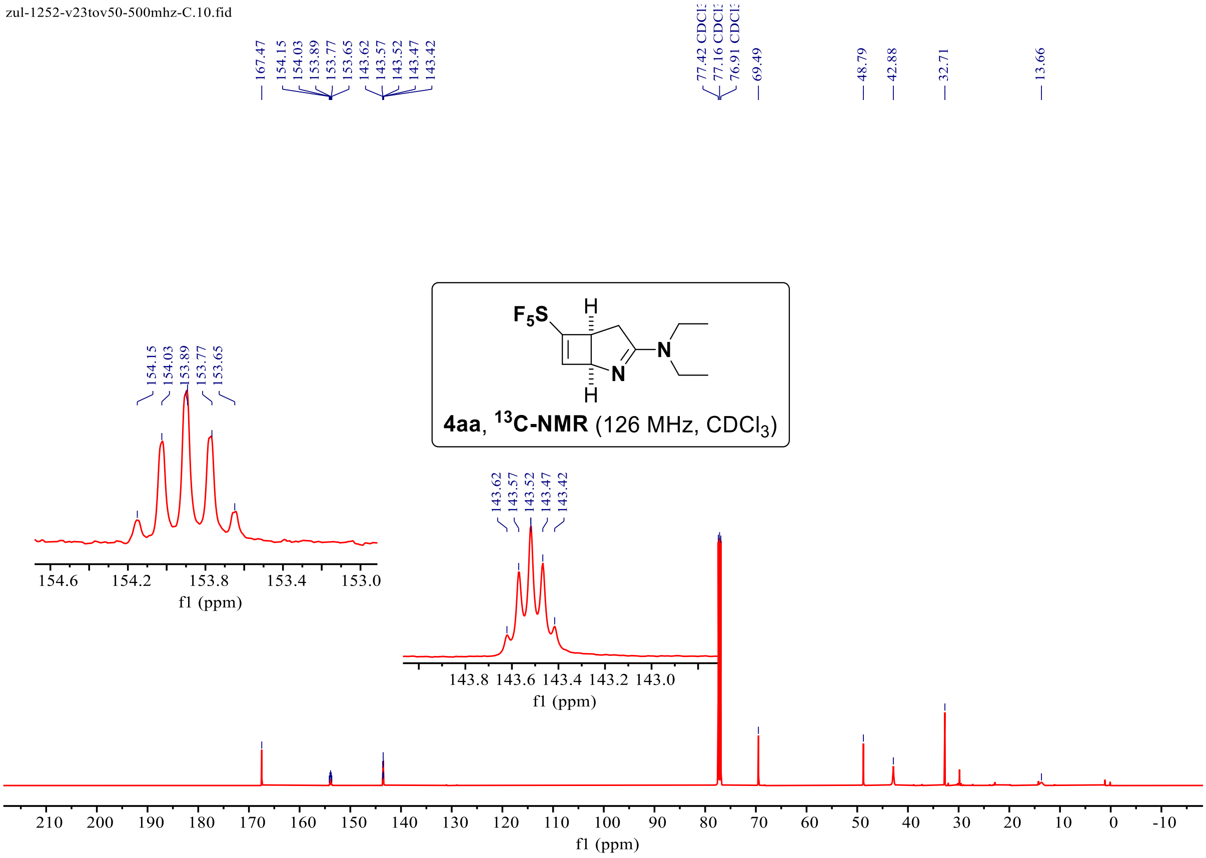
zul-1252-v23tov50-500mhz-C.10.fid
167.47
154.15 154.03 153.89 153.77 153.65
143.62 143.57 143.52 143.47 143.42
77.42 CDCl3
77.16 CDCl3
76.91 CDCl3
69.49
48.79
42.88
32.71
13.66
F5S
H
N
N
H
4aa, 13C-NMR (126 MHz, CDCl3)
154.6 154.2 153.8 153.4 153.0
f1 (ppm)
143.8 143.6 143.4 143.2 143.0
f1 (ppm)
210 200 190 180 170 160 150 140 130 120 110 100 90 80 70 60 50 40 30 20 10 0 -10
f1 (ppm)

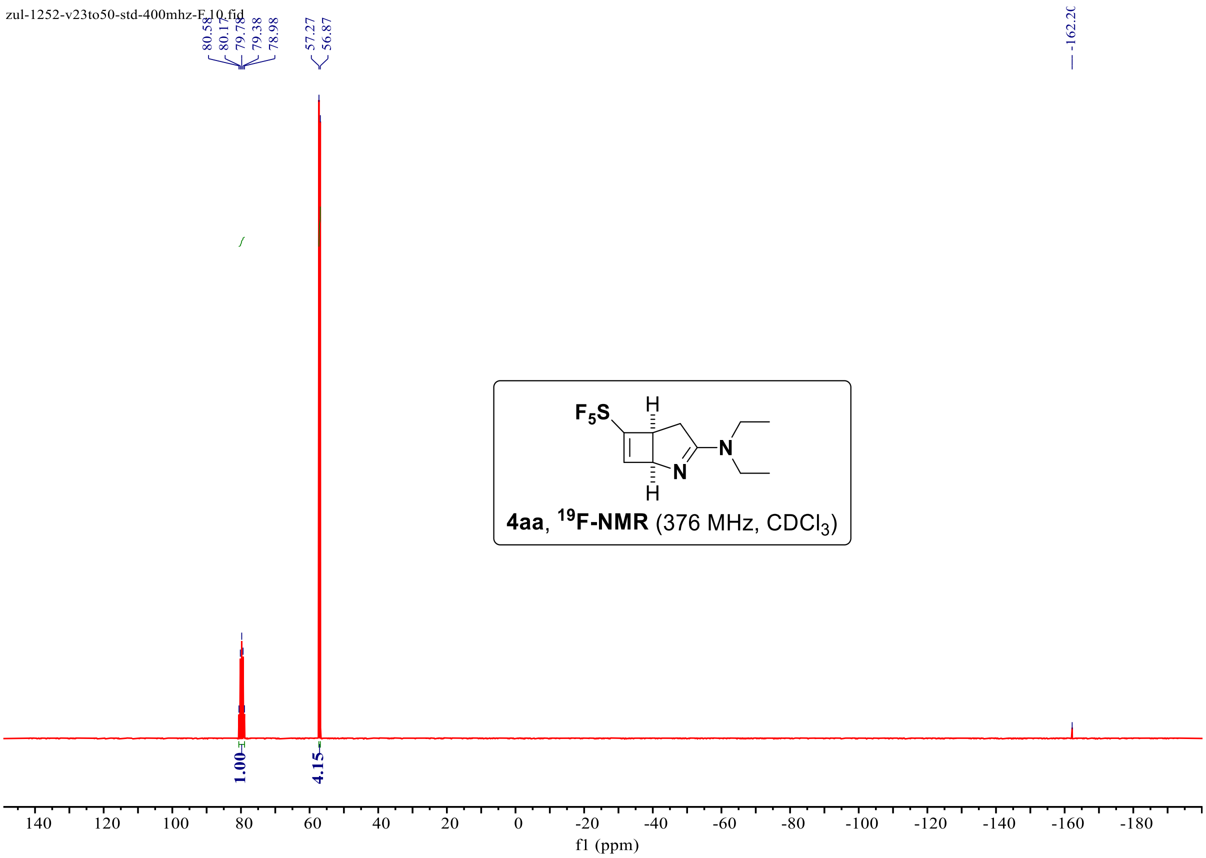
zul-1252-v23to50-std-400mhz-F.10.fid
80.58
80.17
79.78
79.38
78.98
57.27
56.87
-162.20
F5S
H
N
N
H
4aa, 19F-NMR (376 MHz, CDCl3)
1.00
4.15
f1 (ppm)

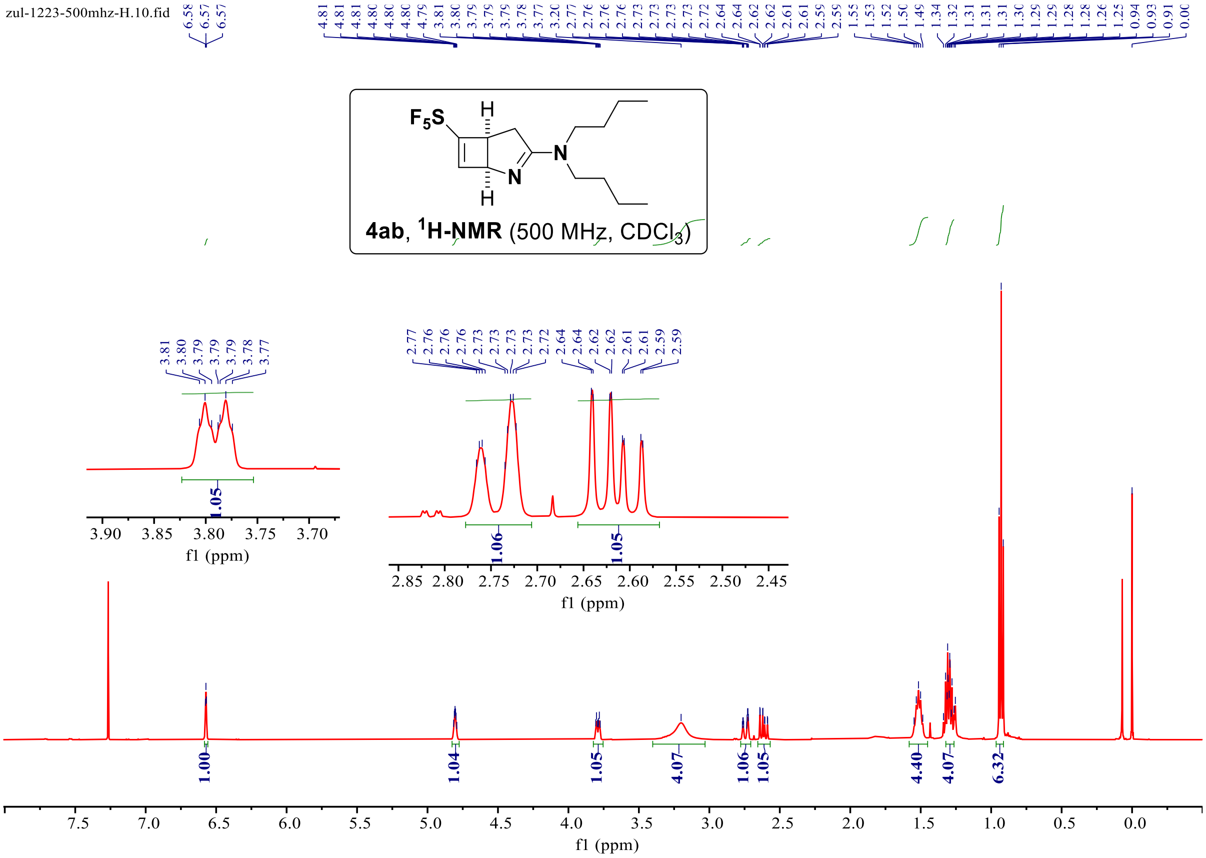
zul-1223-500mhz-H.10.fid
F5S
H
N
N
H
4ab, 1H-NMR (500 MHz, CDCl3)
f1 (ppm)

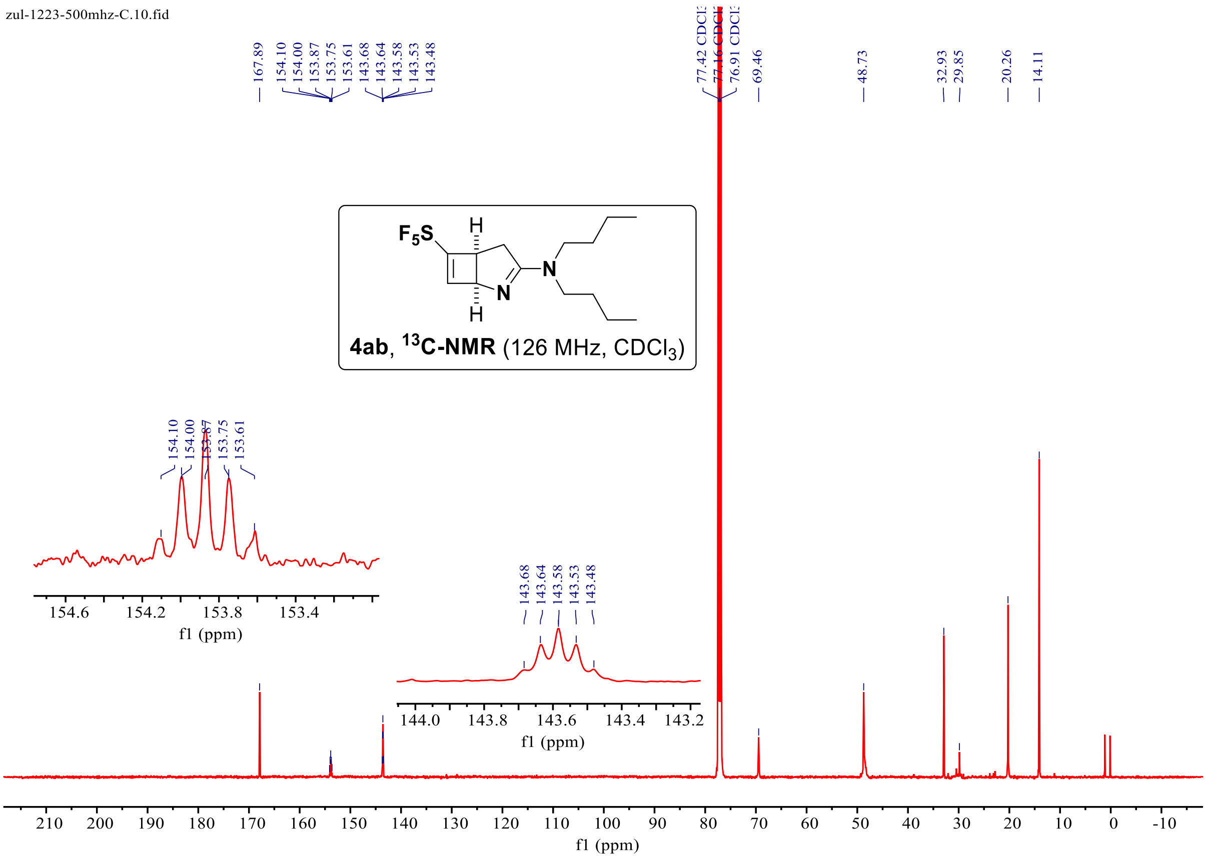
zul-1223-500mhz-C.10.fid
167.89
154.10
154.00
153.87
153.75
153.61
143.68
143.64
143.58
143.53
143.48
77.42 CDCl3
77.16 CDCl3
76.91 CDCl3
69.46
48.73
32.93
29.85
20.26
14.11
F5S
H
N
N
H
4ab, 13C-NMR (126 MHz, CDCl3)
154.6
154.2
153.8
153.4
f1 (ppm)
144.0
143.8
143.6
143.4
143.2
f1 (ppm)
210
200
190
180
170
160
150
140
130
120
110
100
90
80
70
60
50
40
30
20
10
0
-10
f1 (ppm)

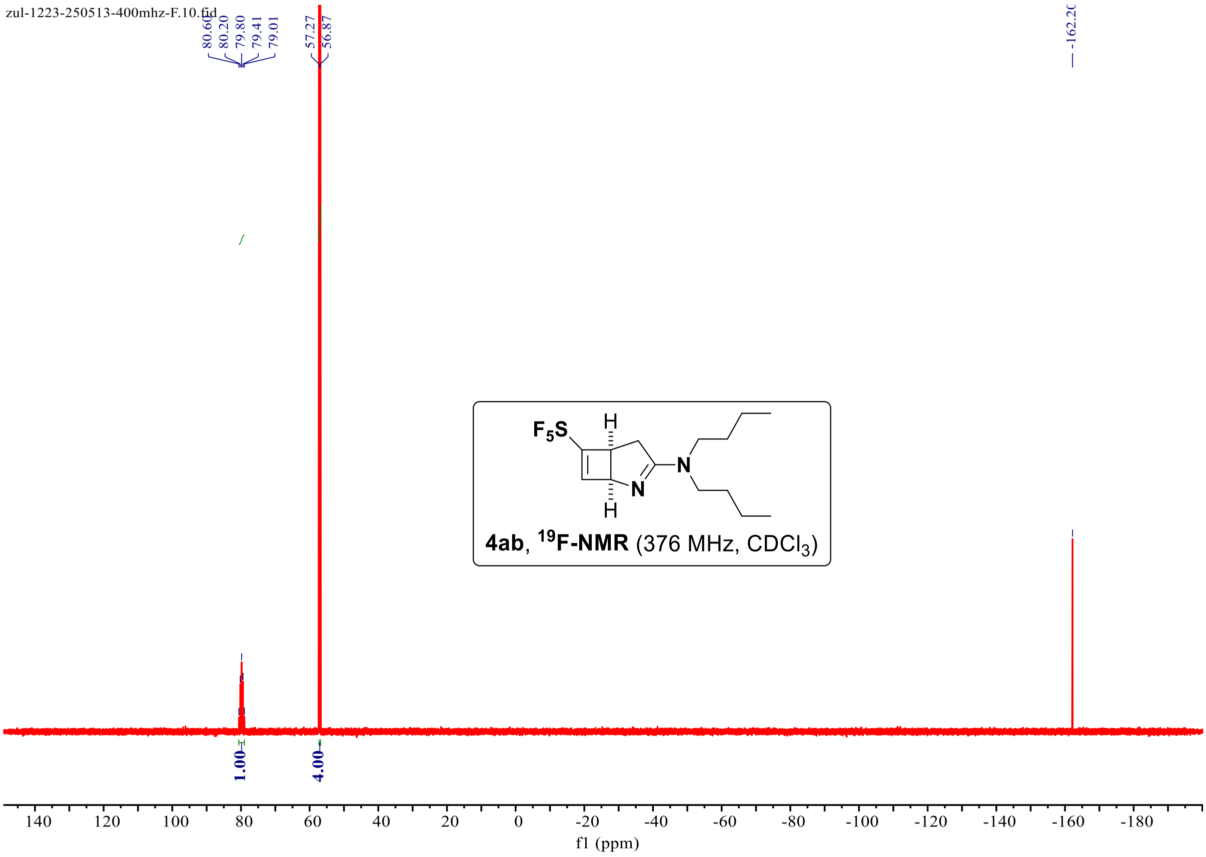
zul-1223-250513-400mhz-F.10.fid
80.60
80.20
79.80
79.41
79.01
57.27
56.87
-162.20
F5S
H
N
N
H
4ab, 19F-NMR (376 MHz, CDCl3)
1.00
4.00
140
120
100
80
60
40
20
0
-20
-40
-60
-80
-100
-120
-140
-160
-180
f1 (ppm)

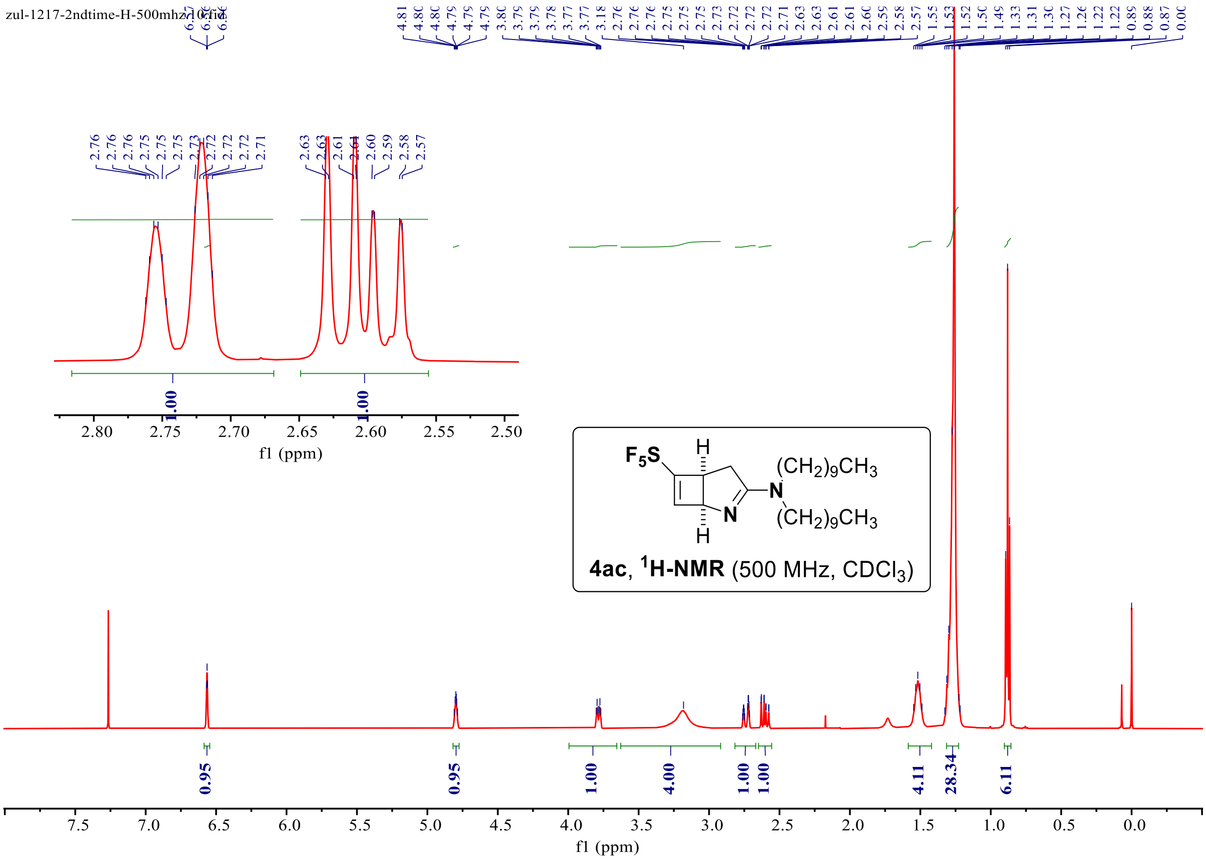
zul-1217-2ndtime-H-500mhz.10.fid
4ac, 1H-NMR (500 MHz, CDCl3)
F5S
H
N
(CH2)9CH3
N
(CH2)9CH3
H
0.95
0.95
1.00
4.00
1.00
1.00
4.11
28.34
6.11
f1 (ppm)

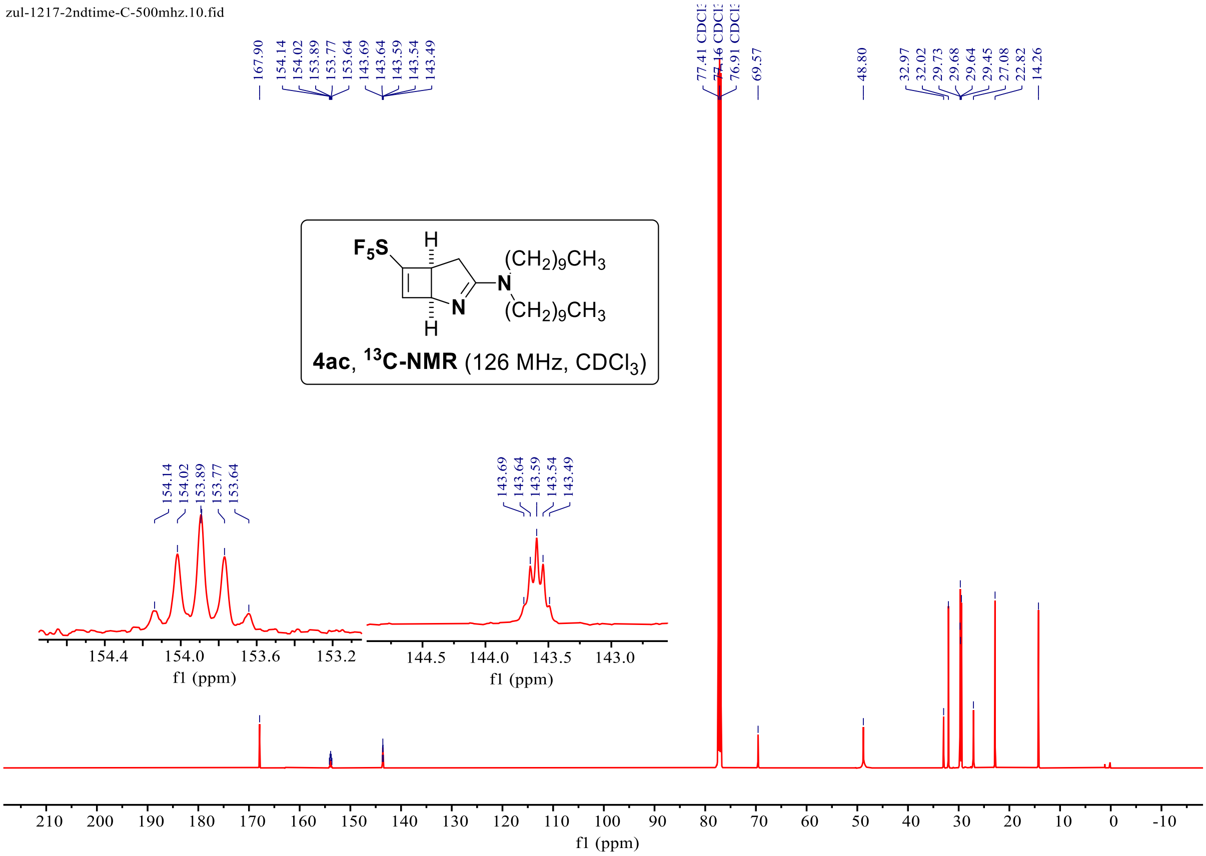
zul-1217-2ndtime-C-500mhz.10.fid
167.90
154.14
154.02
153.89
153.77
153.64
143.69
143.64
143.59
143.54
143.49
69.57
48.80
32.97
32.02
29.73
29.68
29.64
29.45
27.08
22.82
14.26
4ac, 13C-NMR (126 MHz, CDCl3)
F5S
H
N
(CH2)9CH3
N
(CH2)9CH3
H
f1 (ppm)

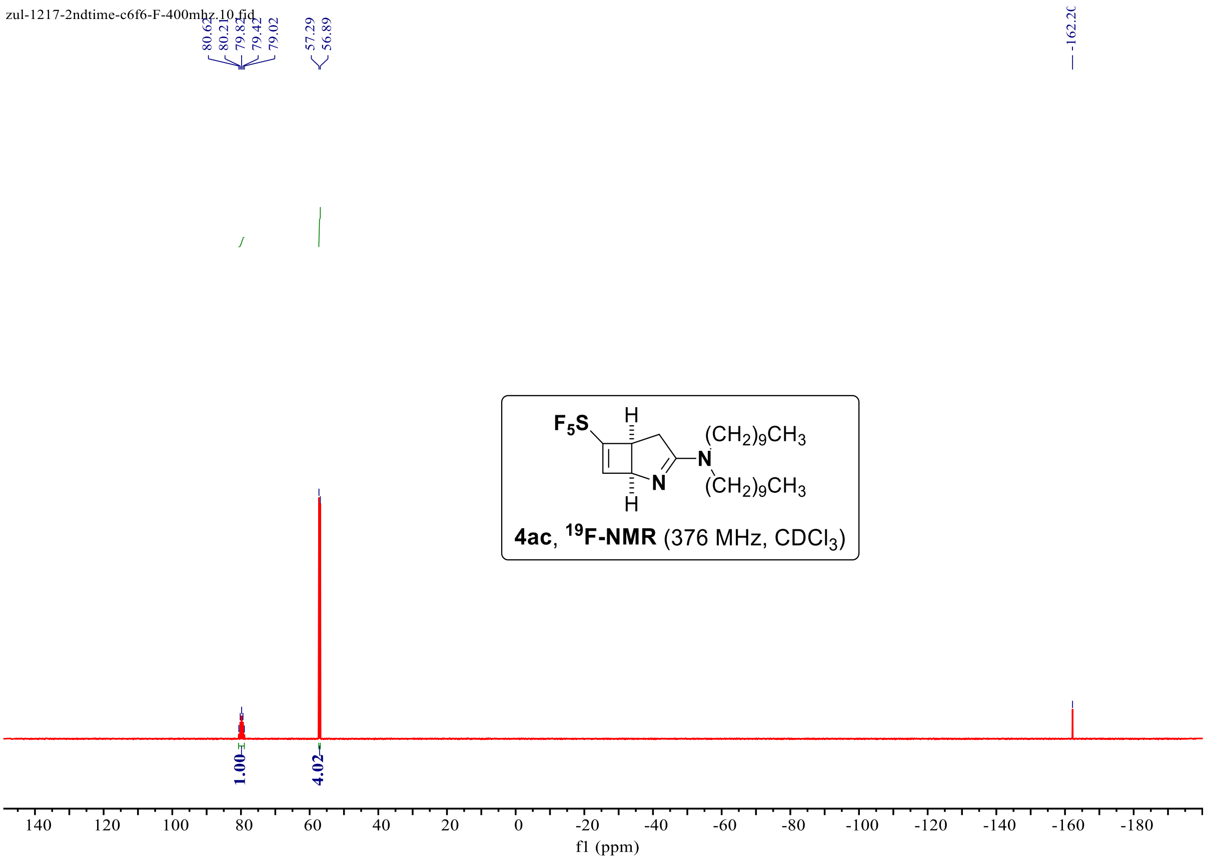
zul-1217-2ndtime-c6f6-F-400mhz.10.fid
4ac, 19F-NMR (376 MHz, CDCl3)
f1 (ppm)

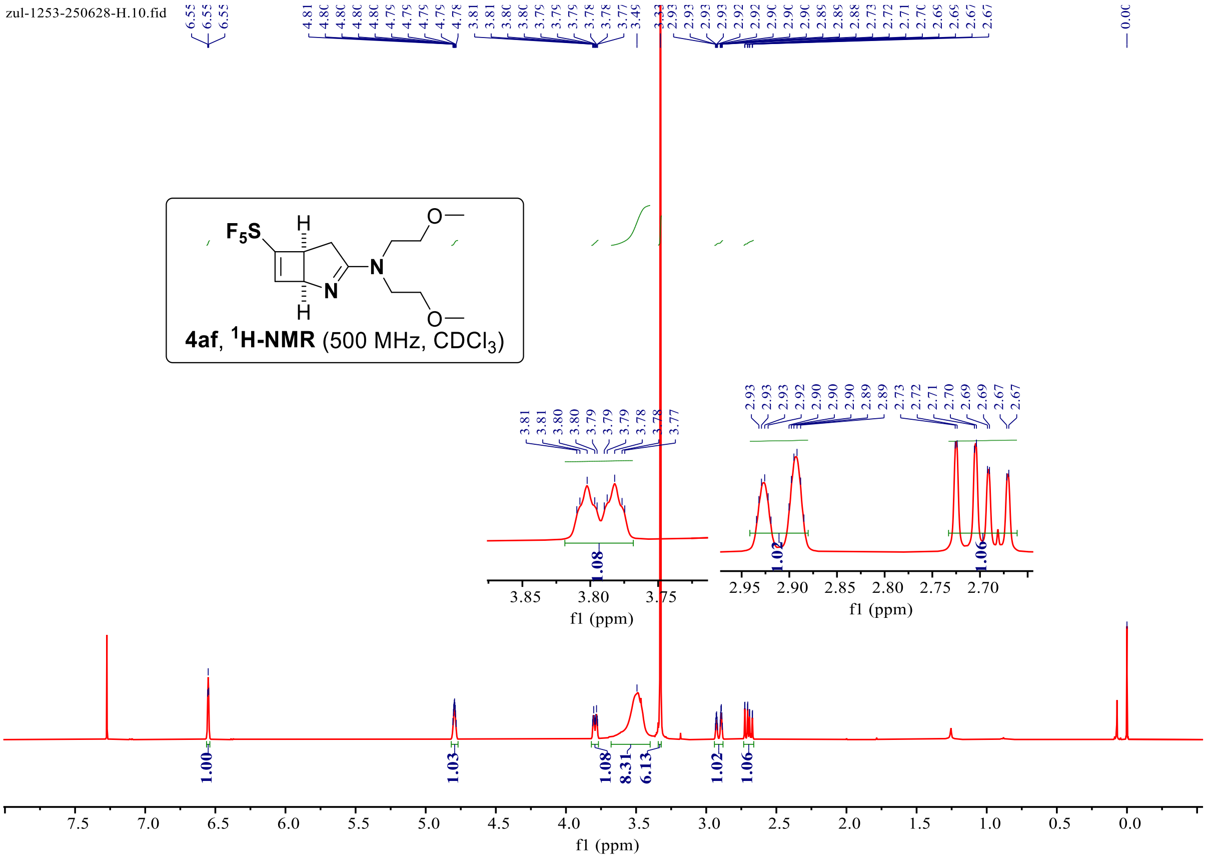
zul-1253-250628-H.10.fid
4af, 1H-NMR (500 MHz, CDCl3)
f1 (ppm)

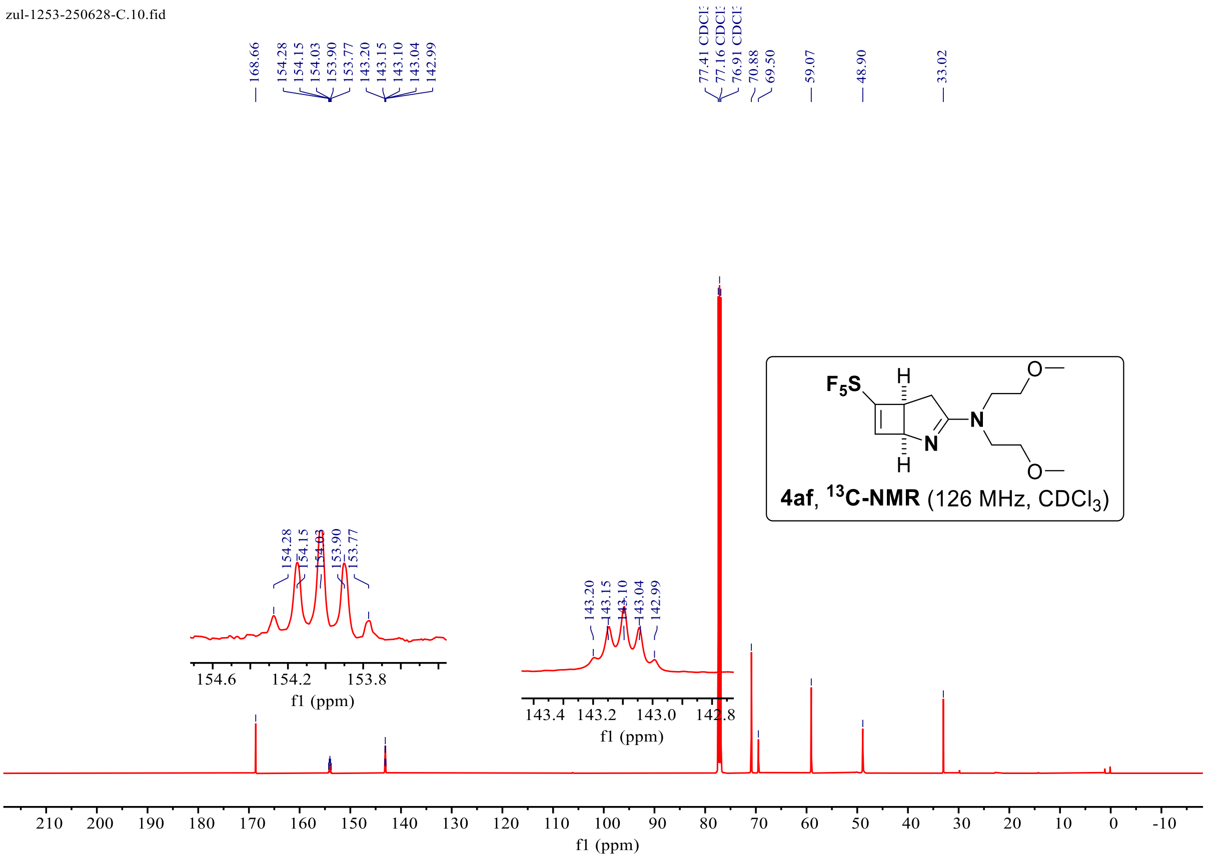

zul-1253-250628-C.10.fid
168.66
154.28
154.15
154.03
153.90
153.77
143.20
143.15
143.10
143.04
142.99
77.41 CDCl3
77.16 CDCl3
76.91 CDCl3
70.88
69.50
59.07
48.90
33.02
4af, 13C-NMR (126 MHz, CDCl3)
f1 (ppm)

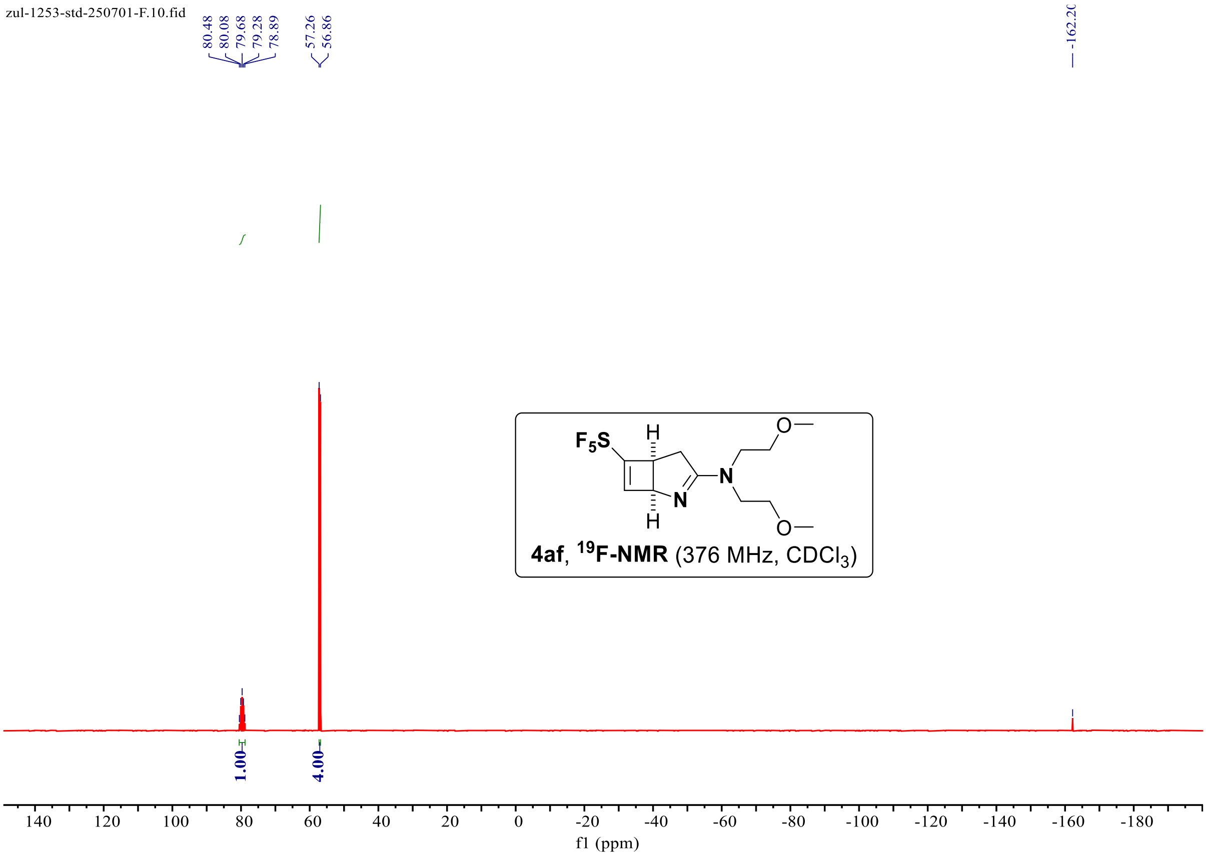

zul-1253-std-250701-F.10.fid
80.48
80.08
79.68
79.28
78.89
57.26
56.86
-162.20
1.00
4.00
4af, 19F-NMR (376 MHz, CDCl3)
f1 (ppm)

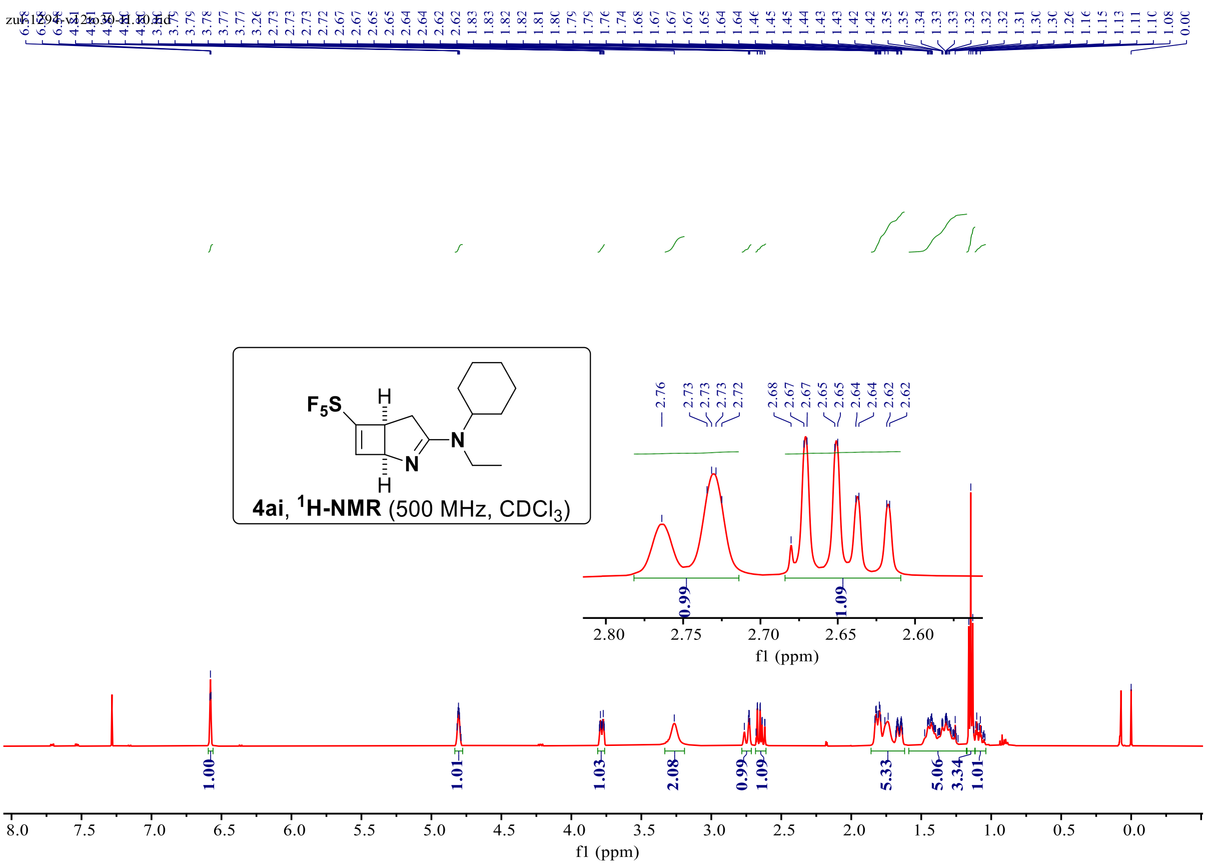
F5S
H
N
N
H
4ai, 1H-NMR (500 MHz, CDCl3)
2.76
2.73
2.73
2.73
2.72
2.68
2.67
2.67
2.65
2.65
2.64
2.64
2.62
2.62
0.99
1.09
2.80 2.75 2.70 2.65 2.60
f1 (ppm)
1.00
1.01
1.03
2.08
0.99
1.09
5.33
5.06
3.34
1.01
8.0 7.5 7.0 6.5 6.0 5.5 5.0 4.5 4.0 3.5 3.0 2.5 2.0 1.5 1.0 0.5 0.0
f1 (ppm)

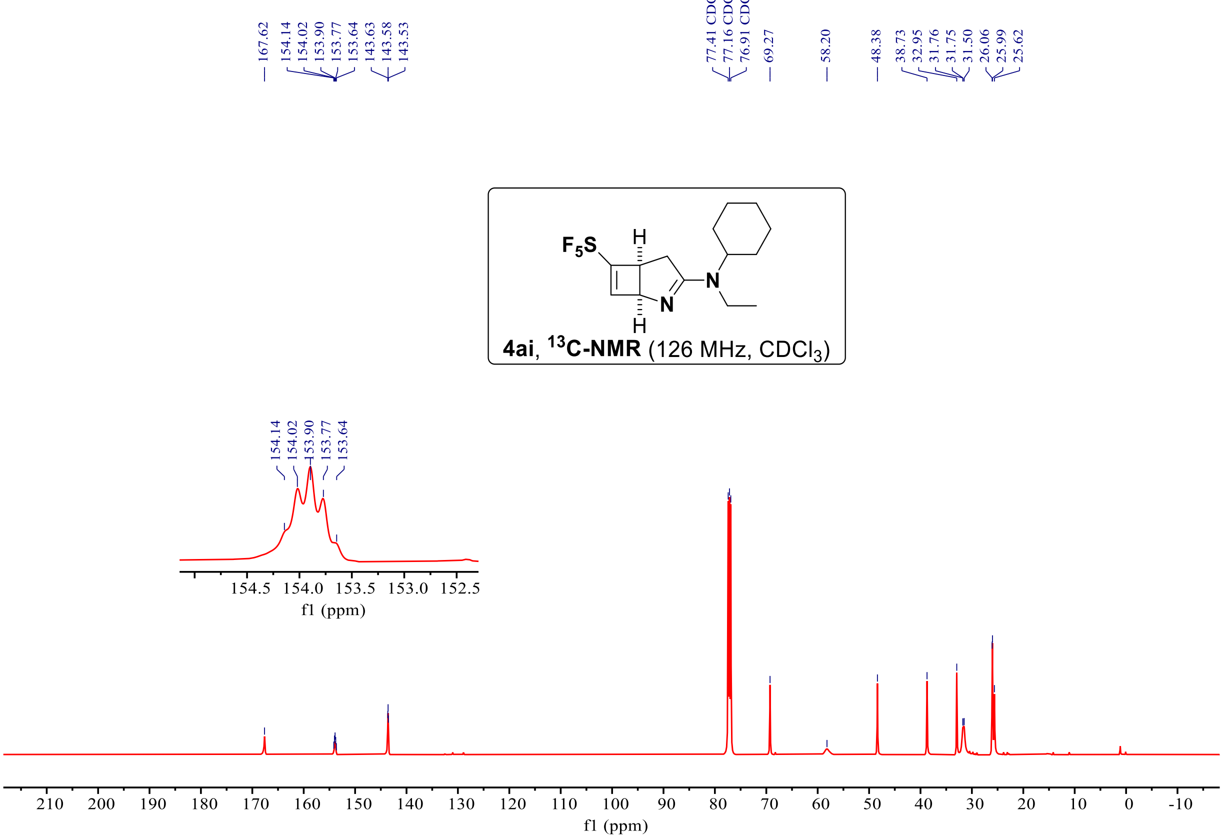
zul-1294-v12to30-C.10.fid
167.62
154.14
154.02
153.90
153.77
153.64
143.63
143.58
143.53
77.41 CDCl3
77.16 CDCl3
76.91 CDCl3
69.27
58.20
48.38
38.73
32.95
31.76
31.75
31.50
26.06
25.99
25.62
F5S
H
N
N
H
4ai, 13C-NMR (126 MHz, CDCl3)
154.14
154.02
153.90
153.77
153.64
154.5 154.0 153.5 153.0 152.5
f1 (ppm)
210 200 190 180 170 160 150 140 130 120 110 100 90 80 70 60 50 40 30 20 10 0 -10
f1 (ppm)

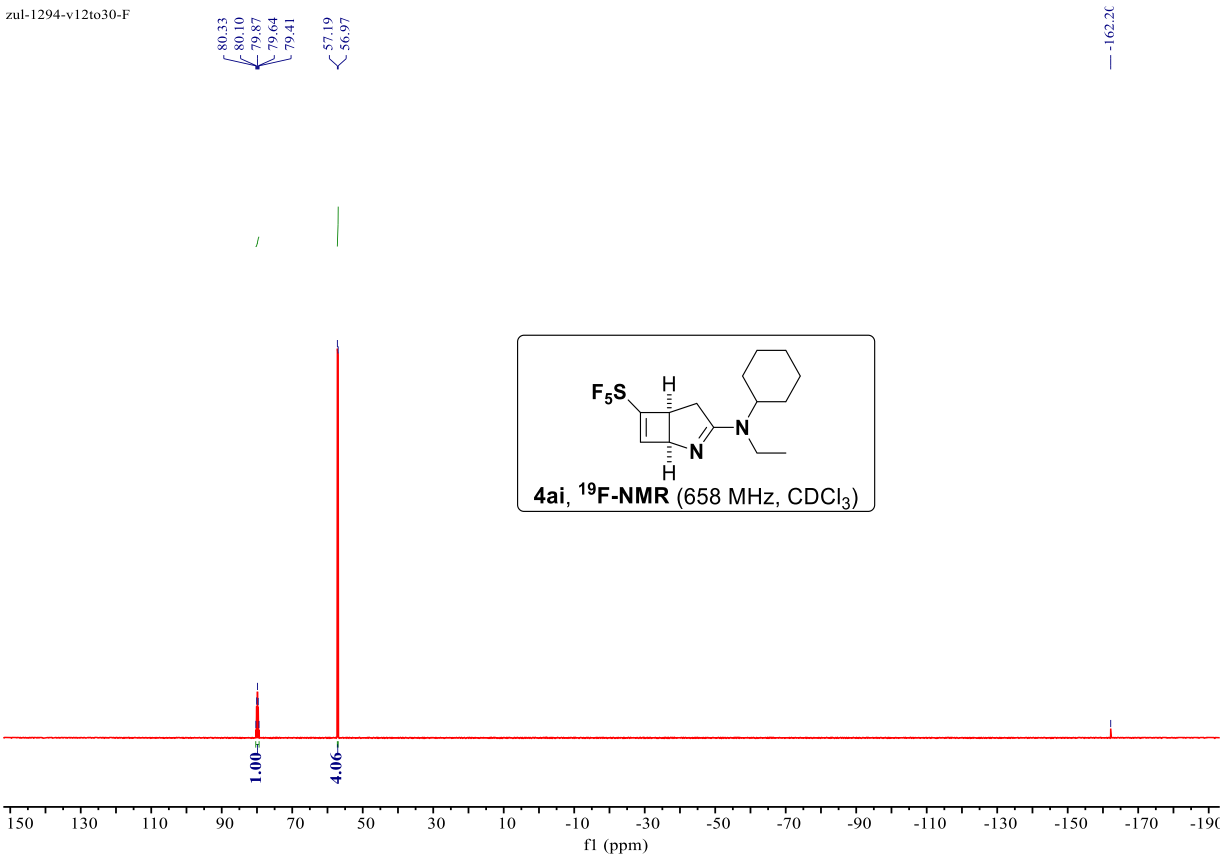
zul-1294-v12to30-F
80.33
80.10
79.87
79.64
79.41
57.19
56.97
-162.20
F5S
H
N
H
4ai, 19F-NMR (658 MHz, CDCl3)
1.00
4.06
150 130 110 90 70 50 30 10 -10 -30 -50 -70 -90 -110 -130 -150 -170
f1 (ppm)

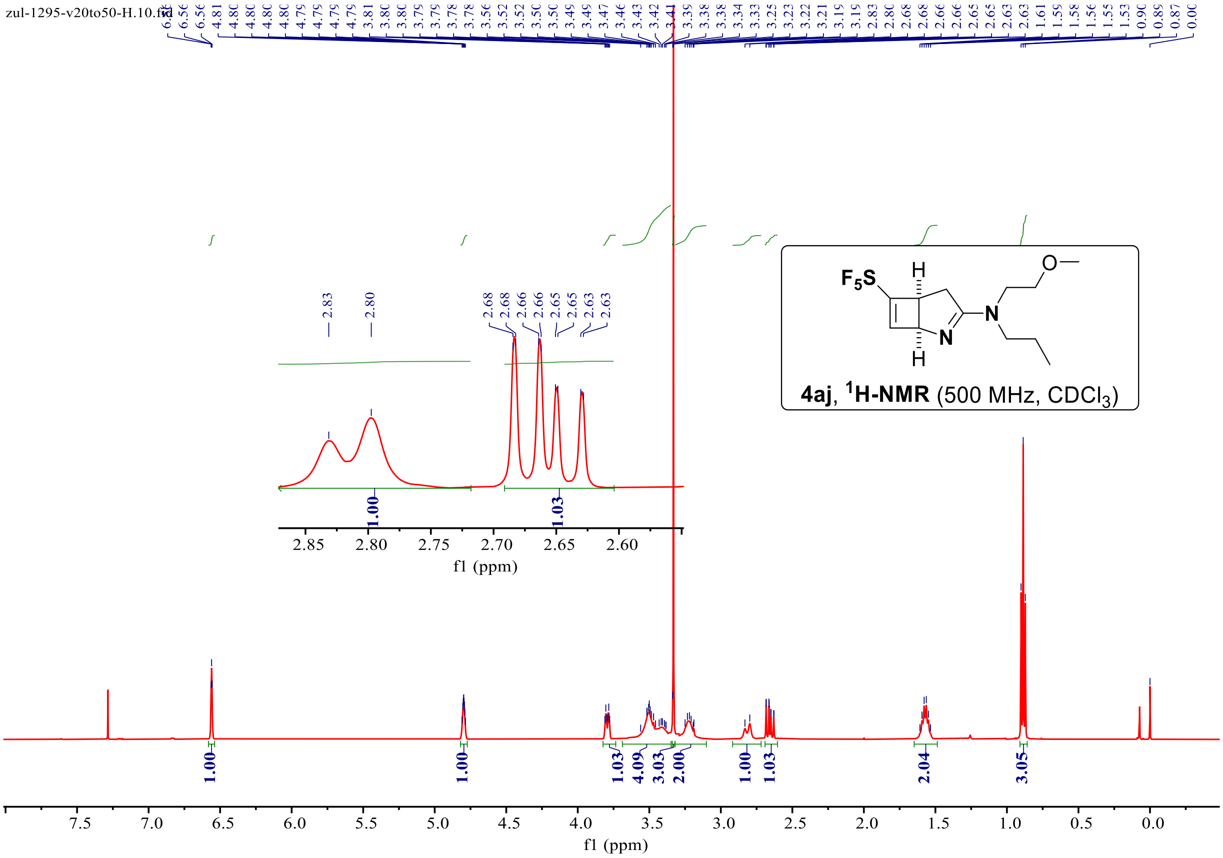
zul-1295-v20to50-H.10.fid
F5S
H
N
O
H
4aj, 1H-NMR (500 MHz, CDCl3)
2.83
2.80
2.68
2.68
2.66
2.66
2.65
2.65
2.63
2.63
1.00
1.03
2.85 2.80 2.75 2.70 2.65 2.60
f1 (ppm)
1.00
1.00
1.03
4.09
3.03
2.00
1.00
1.03
2.04
3.05
7.5 7.0 6.5 6.0 5.5 5.0 4.5 4.0 3.5 3.0 2.5 2.0 1.5 1.0 0.5 0.0
f1 (ppm)

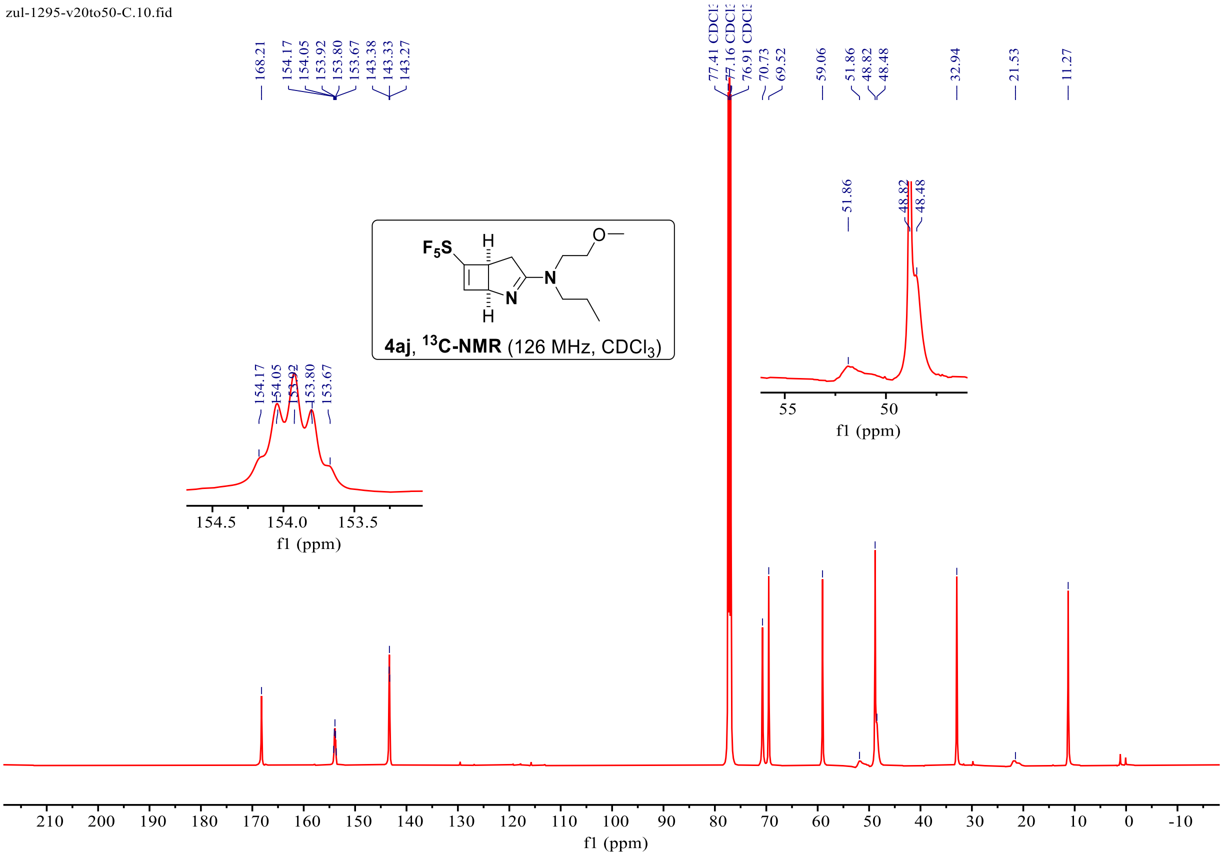
zul-1295-v20to50-C.10.fid
168.21
154.17
154.05
153.92
153.80
153.67
143.38
143.33
143.27
77.41 CDCl3
77.16 CDCl3
76.91 CDCl3
70.73
69.52
59.06
51.86
48.82
48.48
32.94
21.53
11.27
F5S
H
H
N
N
O
4aj, 13C-NMR (126 MHz, CDCl3)
154.5
154.0
153.5
f1 (ppm)
55
50
f1 (ppm)
210 200 190 180 170 160 150 140 130 120 110 100 90 80 70 60 50 40 30 20 10 0 -10
f1 (ppm)

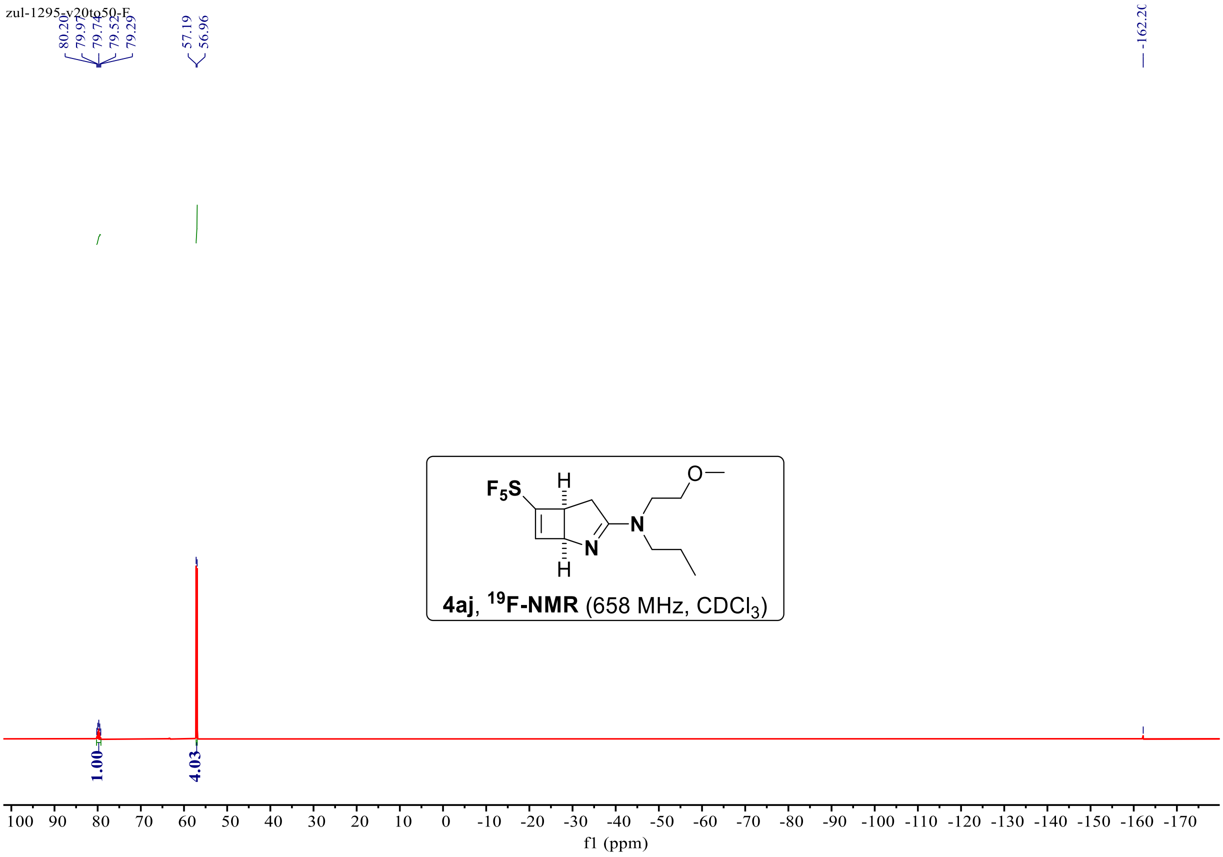
zul-1295-v20to50-F
80.20
79.97
79.74
79.52
79.29
57.19
56.96
-162.20
F5S
H
H
N
N
O
4aj, 19F-NMR (658 MHz, CDCl3)
1.00
4.03
100 90 80 70 60 50 40 30 20 10 0 -10 -20 -30 -40 -50 -60 -70 -80 -90 -100 -110 -120 -130 -140 -150 -160 -170
f1 (ppm)

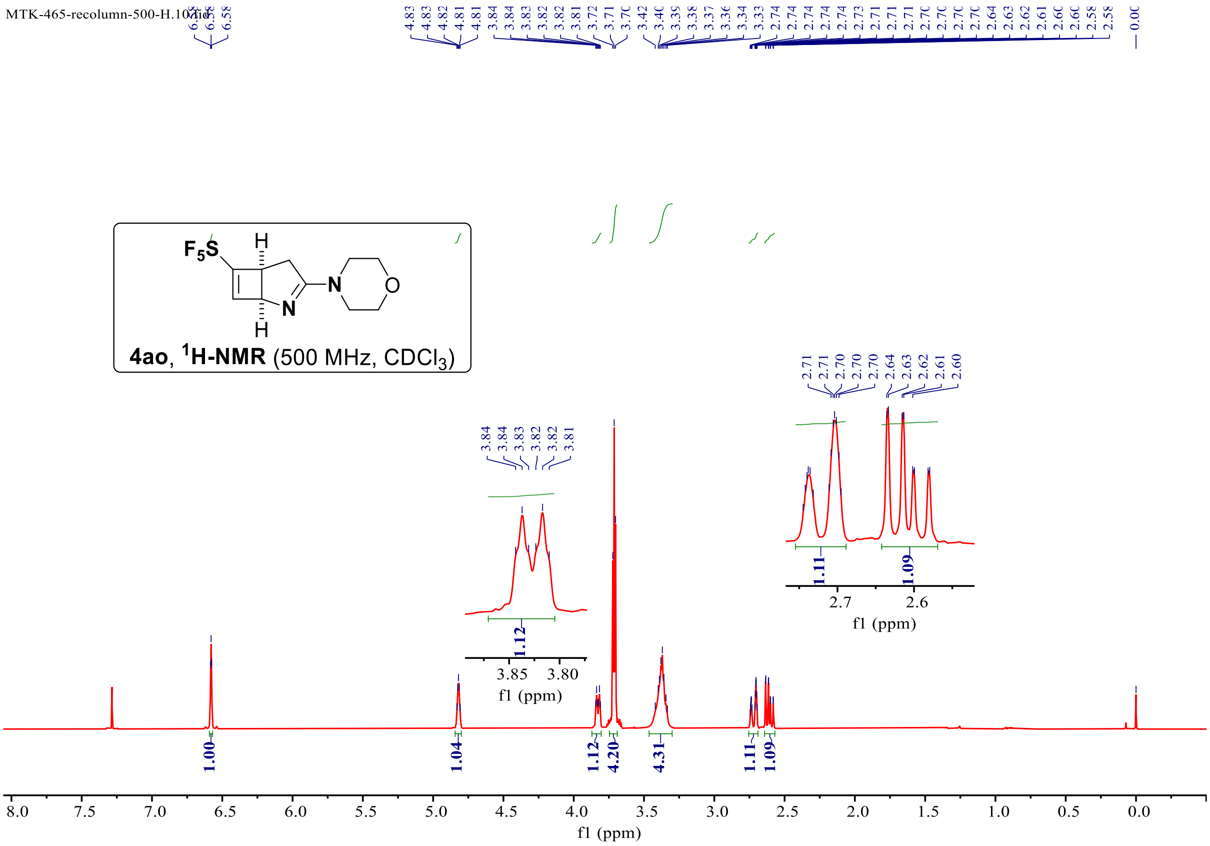
MTK-465-recolumn-500-H.10.fid
4ao, 1H-NMR (500 MHz, CDCl3)
F5S
N
O
H
1.00
1.04
1.12
4.20
4.31
1.11
1.09
f1 (ppm)

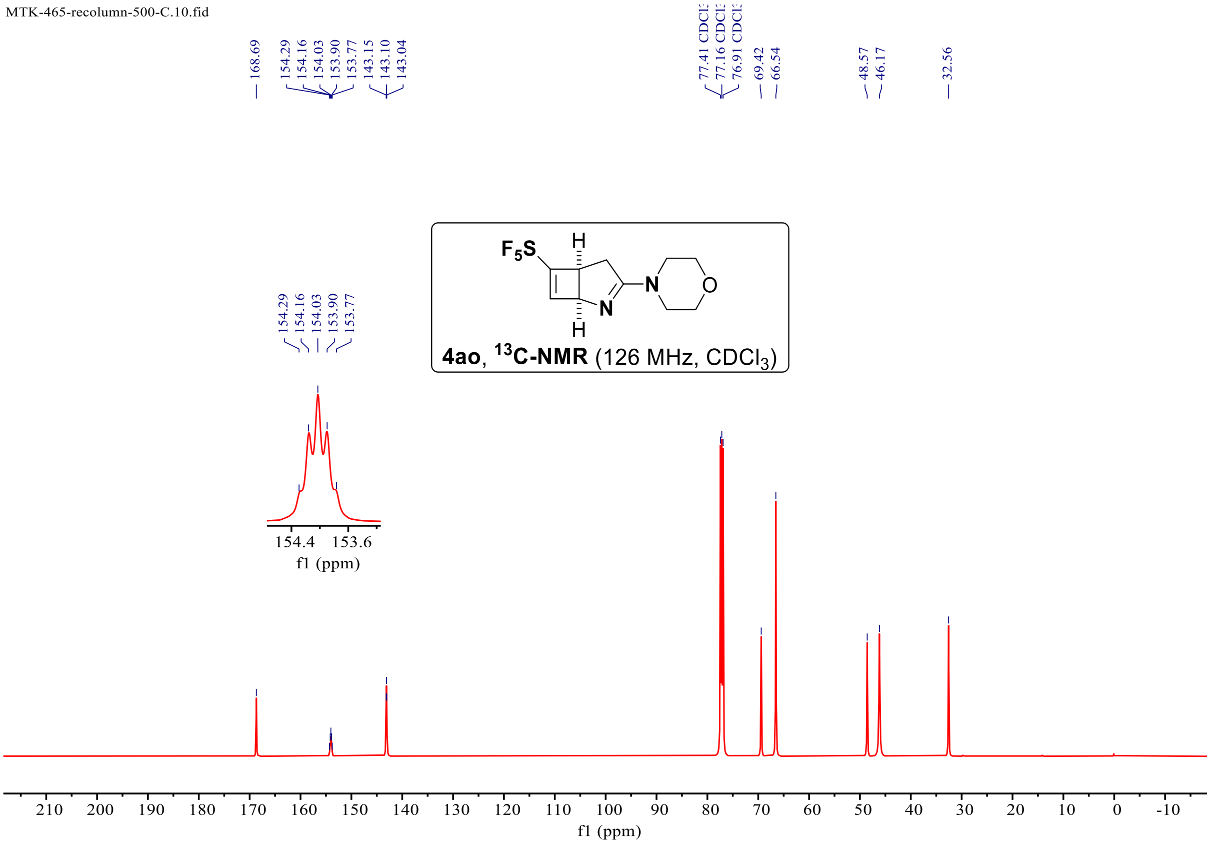
MTK-465-recolumn-500-C.10.fid
168.69
154.29
154.16
154.03
153.90
153.77
143.15
143.10
143.04
69.42
66.54
48.57
46.17
32.56
4ao, 13C-NMR (126 MHz, CDCl3)
F5S
N
O
H
f1 (ppm)

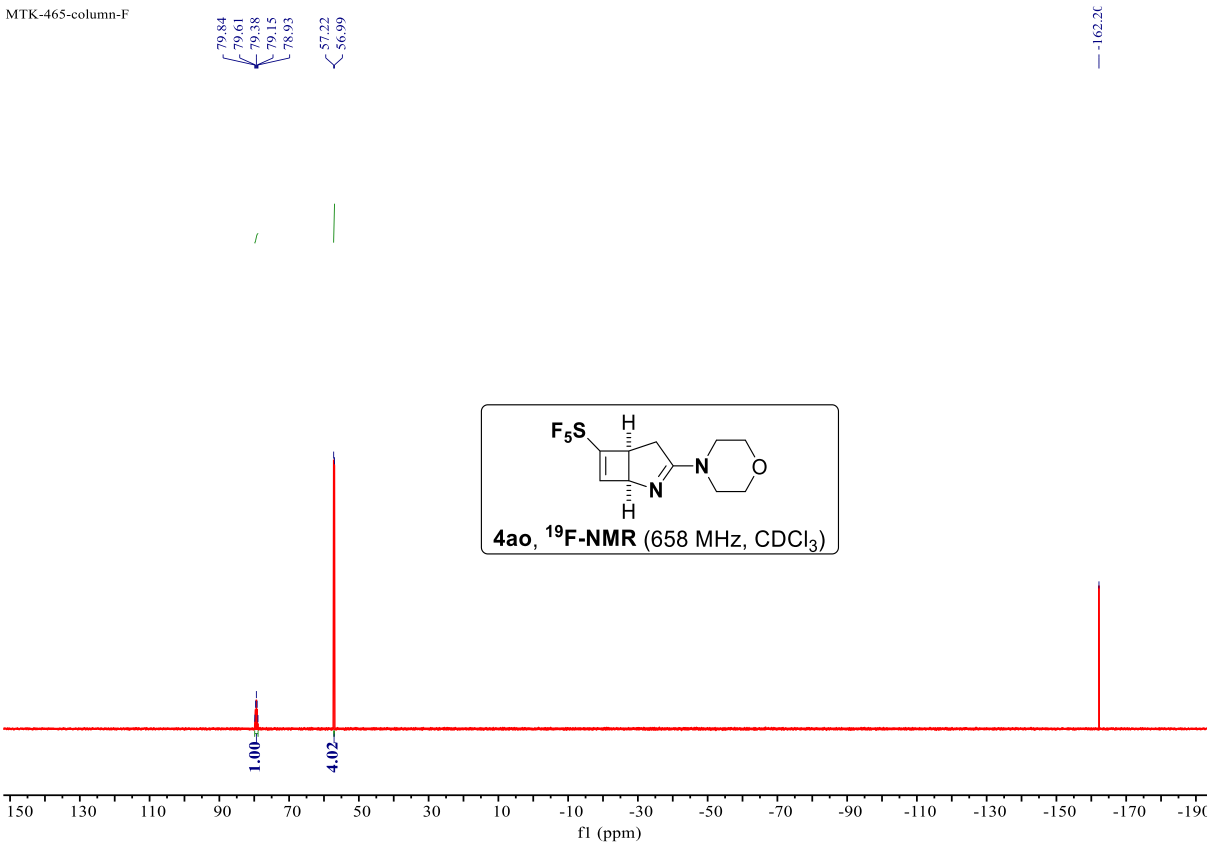
MTK-465-column-F
4ao, 19F-NMR (658 MHz, CDCl3)
f1 (ppm)

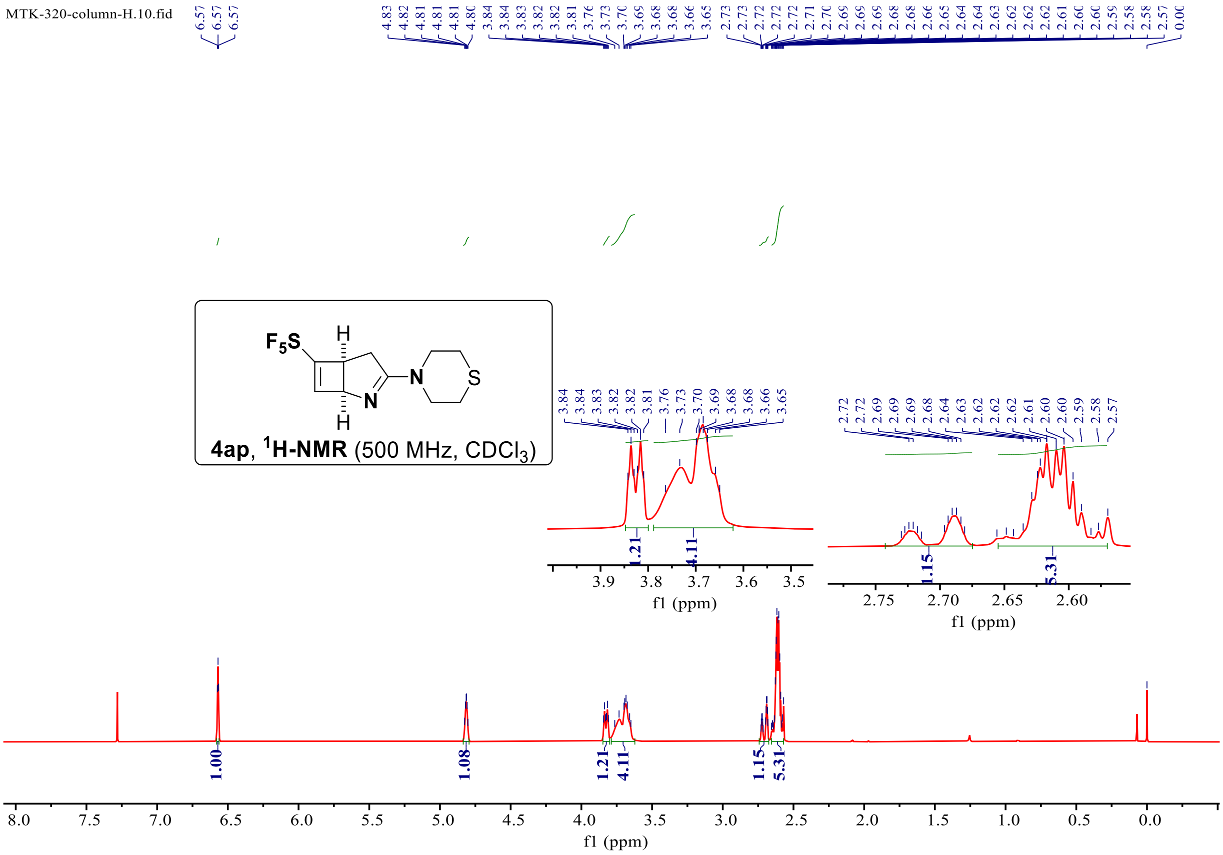
MTK-320-column-H.10.fid
4ap, 1H-NMR (500 MHz, CDCl3)
f1 (ppm)

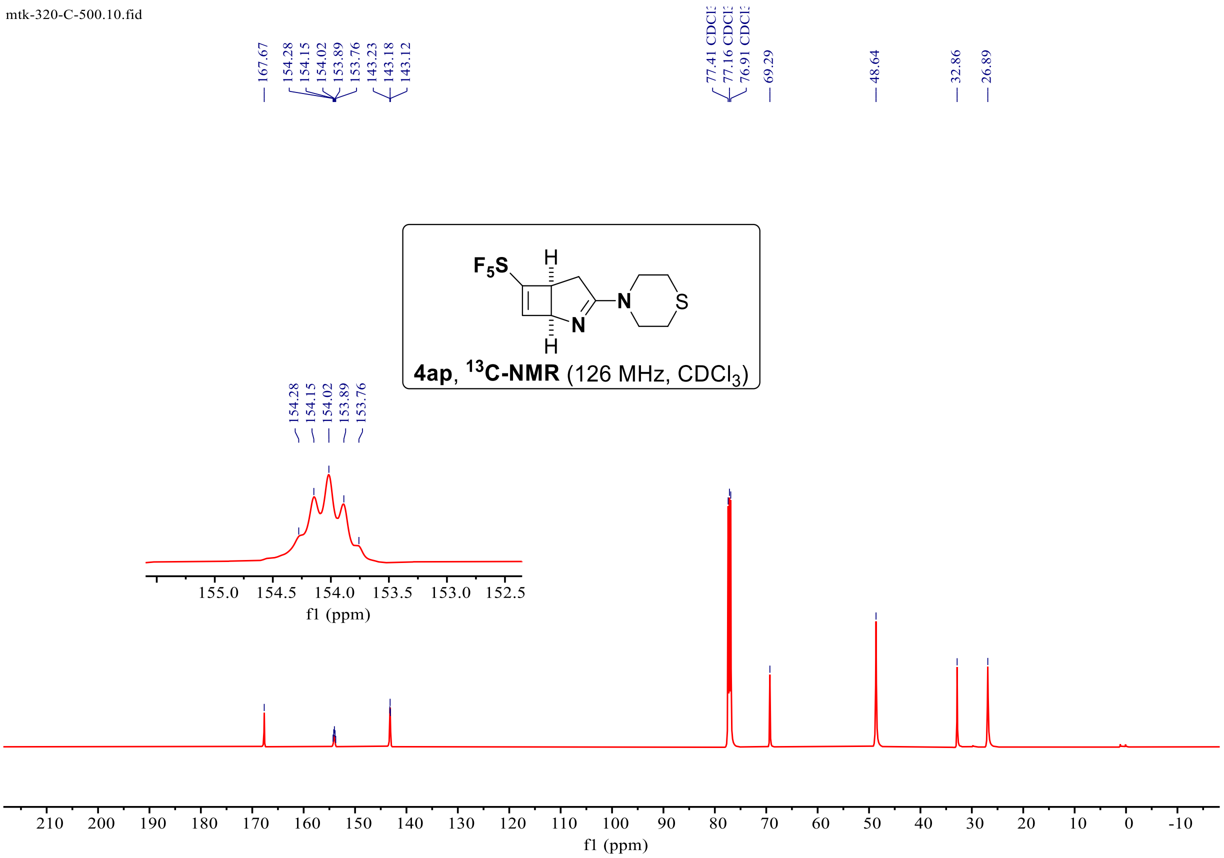
mtk-320-C-500.10.fid
167.67
154.28
154.15
154.02
153.89
153.76
143.23
143.18
143.12
77.41 CDCl
77.16 CDCl
76.91 CDCl
69.29
48.64
32.86
26.89
F5S
H
N
N
S
H
4ap, 13C-NMR (126 MHz, CDCl3)
155.0 154.5 154.0 153.5 153.0 152.5
f1 (ppm)
210 200 190 180 170 160 150 140 130 120 110 100 90 80 70 60 50 40 30 20 10 0 -10
f1 (ppm)

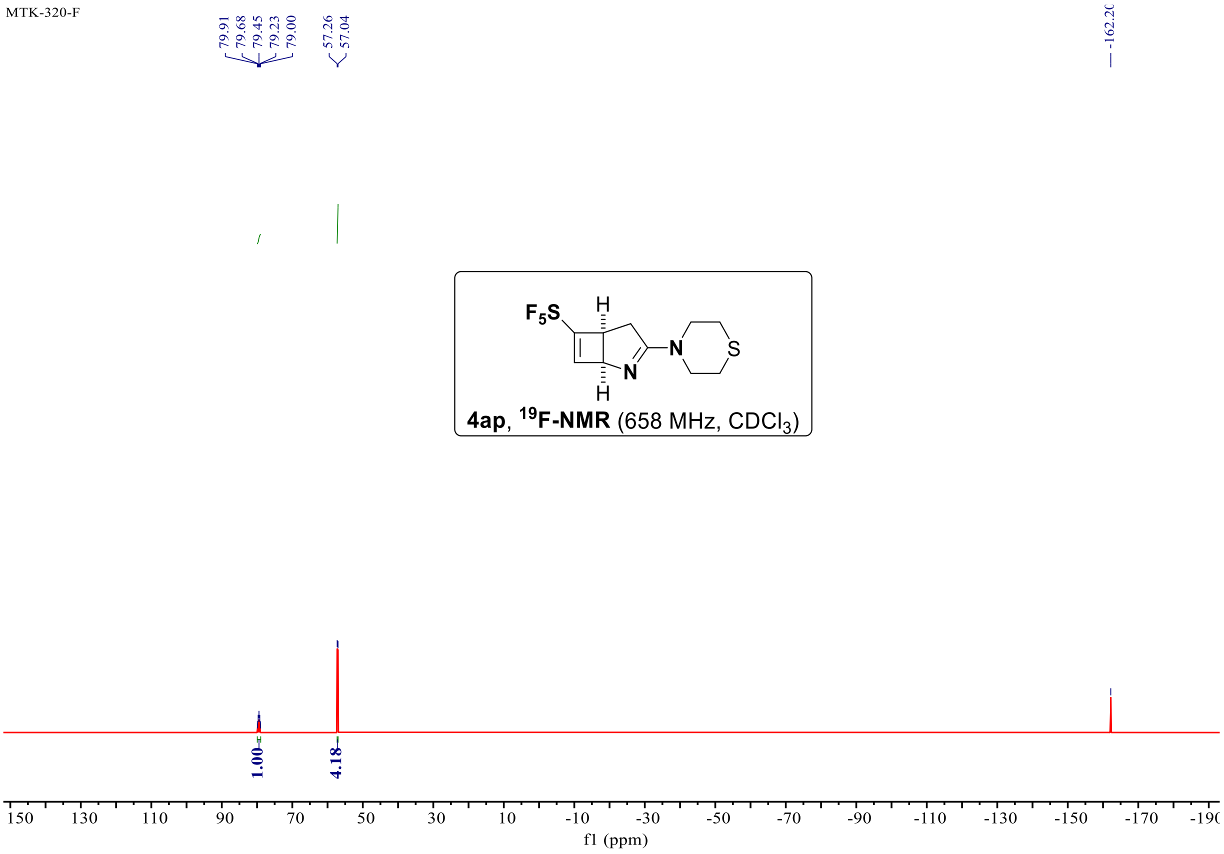
MTK-320-F
79.91
79.68
79.45
79.23
79.00
57.26
57.04
-162.2
F5S
H
N
N
S
H
4ap, 19F-NMR (658 MHz, CDCl3)
1.00
4.18
150 130 110 90 70 50 30 10 -10 -30 -50 -70 -90 -110 -130 -150 -170 -19
f1 (ppm)

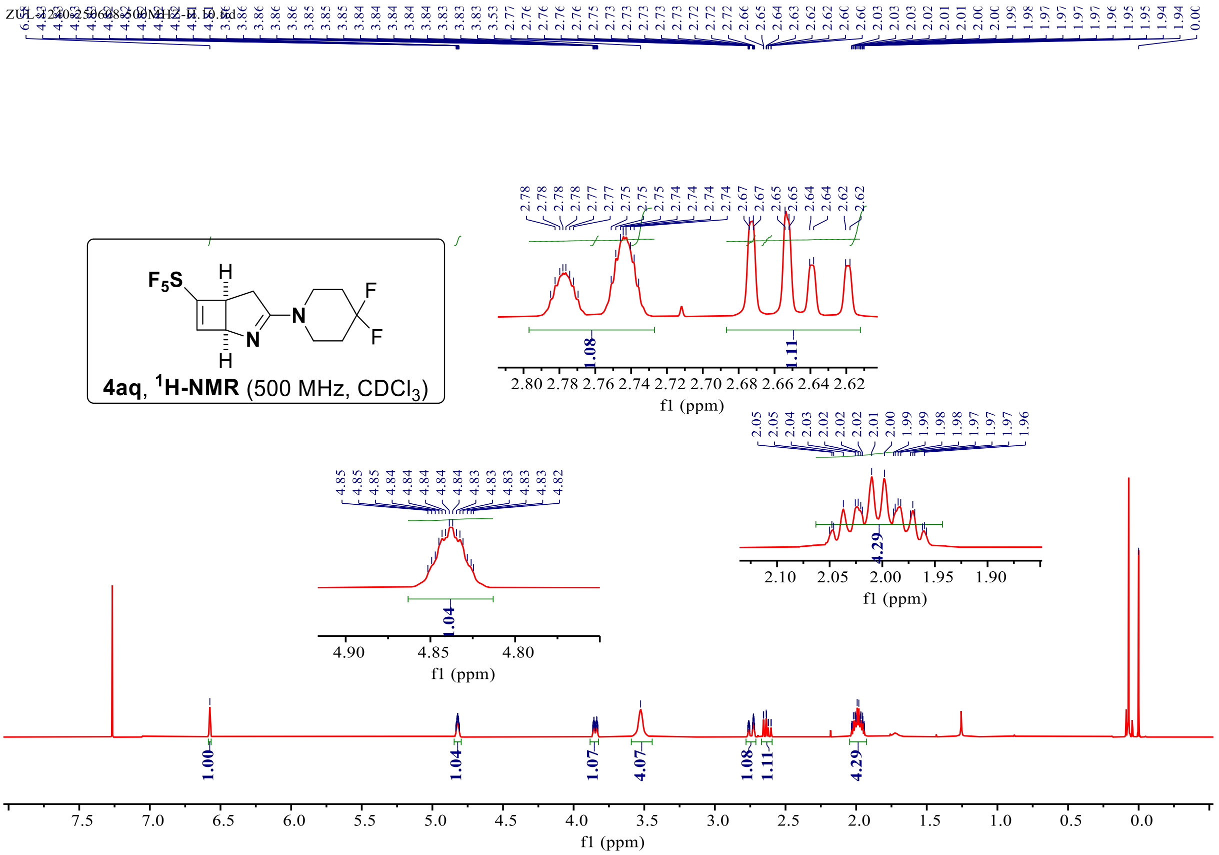
ZUL-1240-250608-500MHZ-H.10.fid
4aq, 1H-NMR (500 MHz, CDCl3)
f1 (ppm)

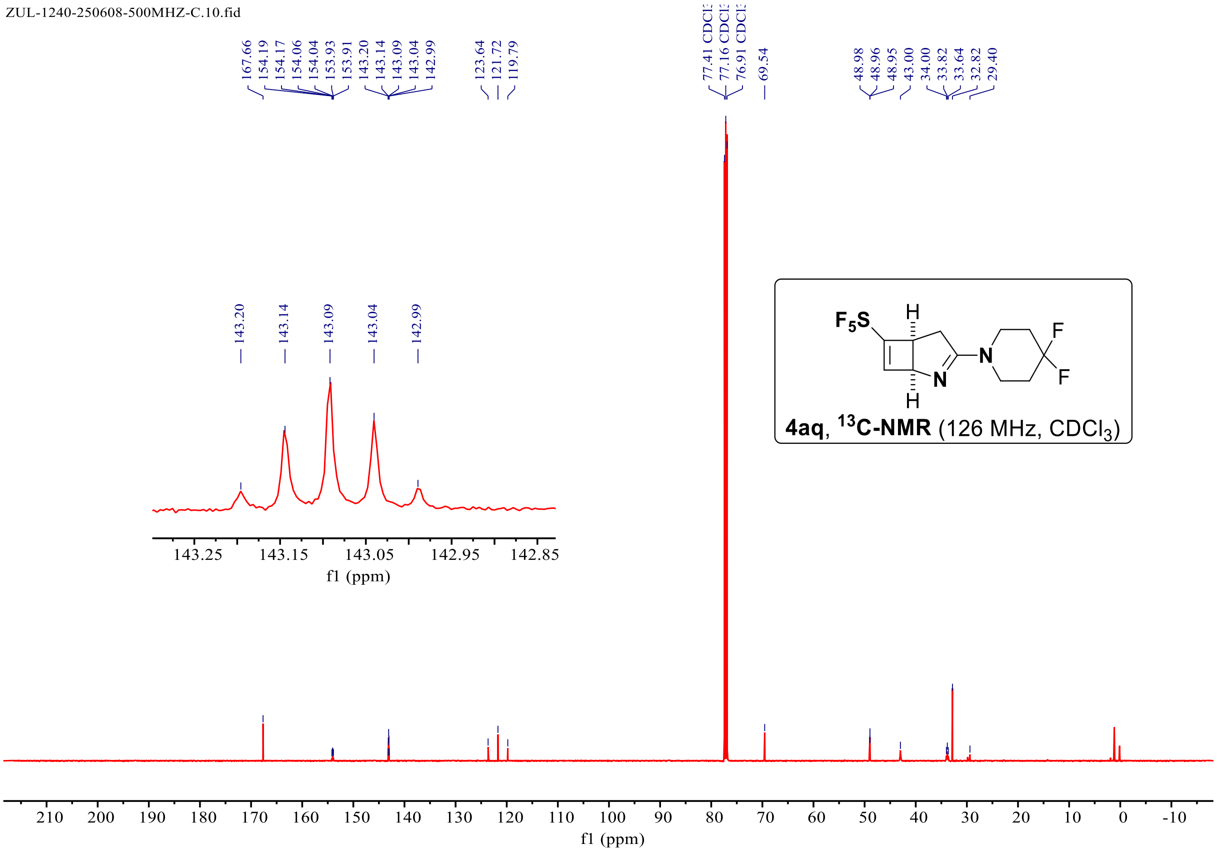
ZUL-1240-250608-500MHZ-C.10.fid
167.66
154.19
154.17
154.06
154.04
153.93
153.91
143.20
143.14
143.09
143.04
142.99
123.64
121.72
119.79
77.41 CDCl3
77.16 CDCl3
76.91 CDCl3
69.54
48.98
48.96
48.95
43.00
34.00
33.82
33.64
32.82
29.40
4aq, 13C-NMR (126 MHz, CDCl3)
f1 (ppm)

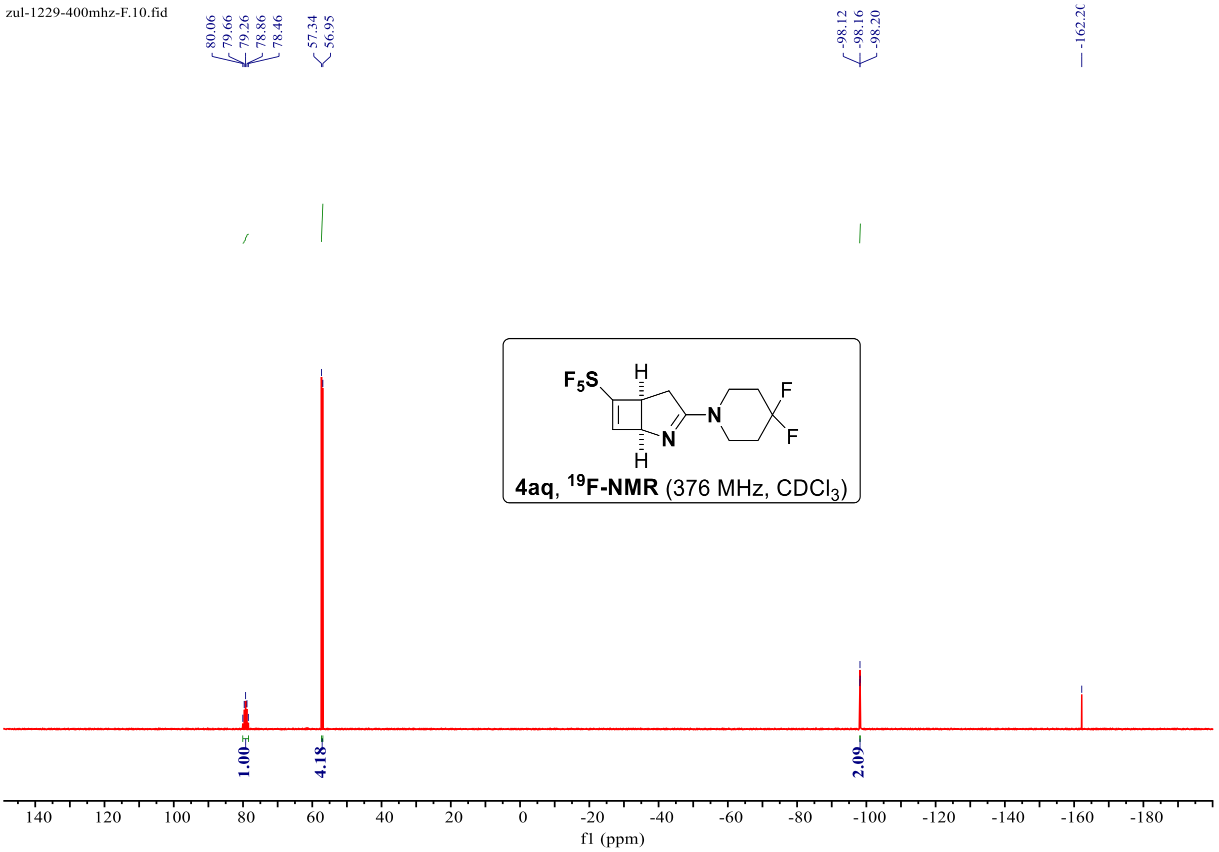
zul-1229-400mhz-F.10.fid
80.06
79.66
79.26
78.86
78.46
57.34
56.95
-98.12
-98.16
-98.20
-162.20
F5S
H
N
N
H
F
F
4aq, 19F-NMR (376 MHz, CDCl3)
1.00
4.18
2.09
f1 (ppm)

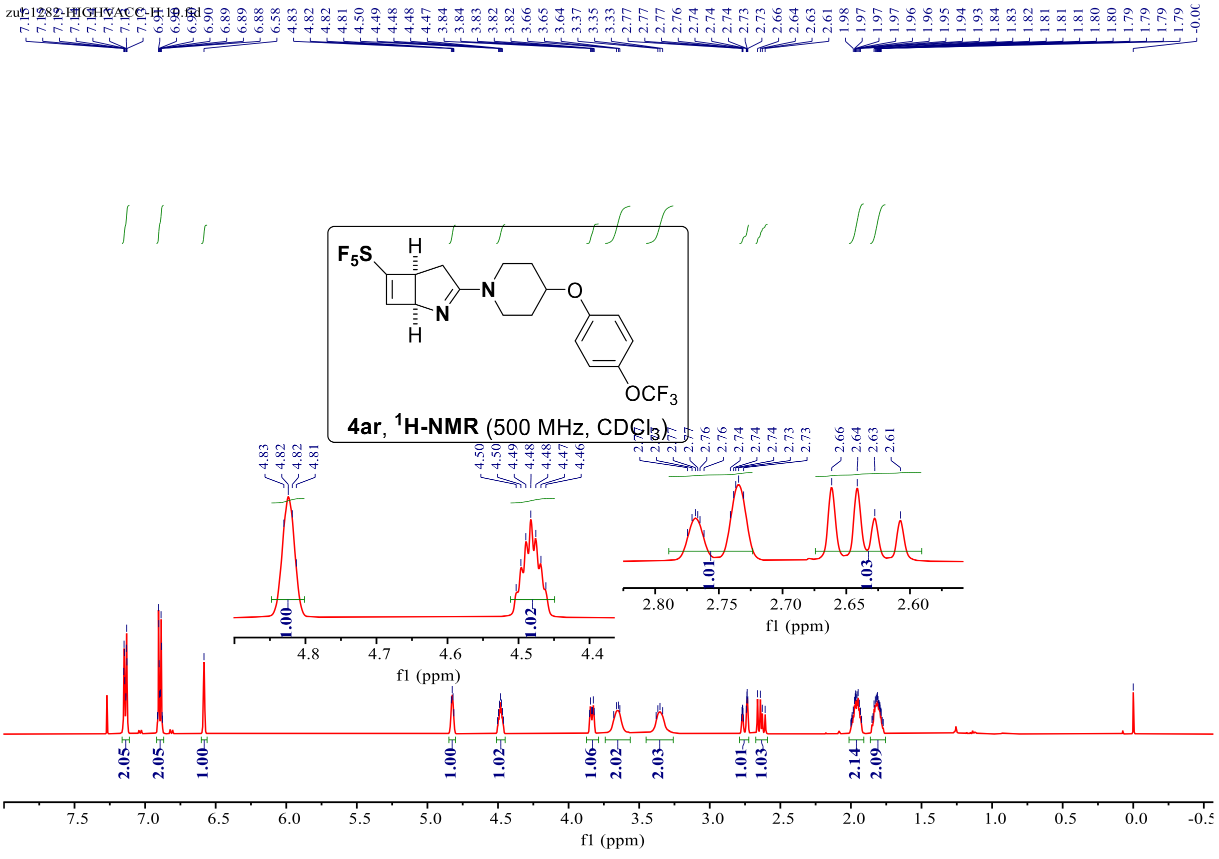
F5S
H
N
N
H
O
OCF3
4ar, 1H-NMR (500 MHz, CDCl3)
1.00
1.02
1.01
1.03
2.05
2.05
1.00
1.00
1.02
1.06
2.02
2.03
1.01
1.03
2.14
2.09
f1 (ppm)

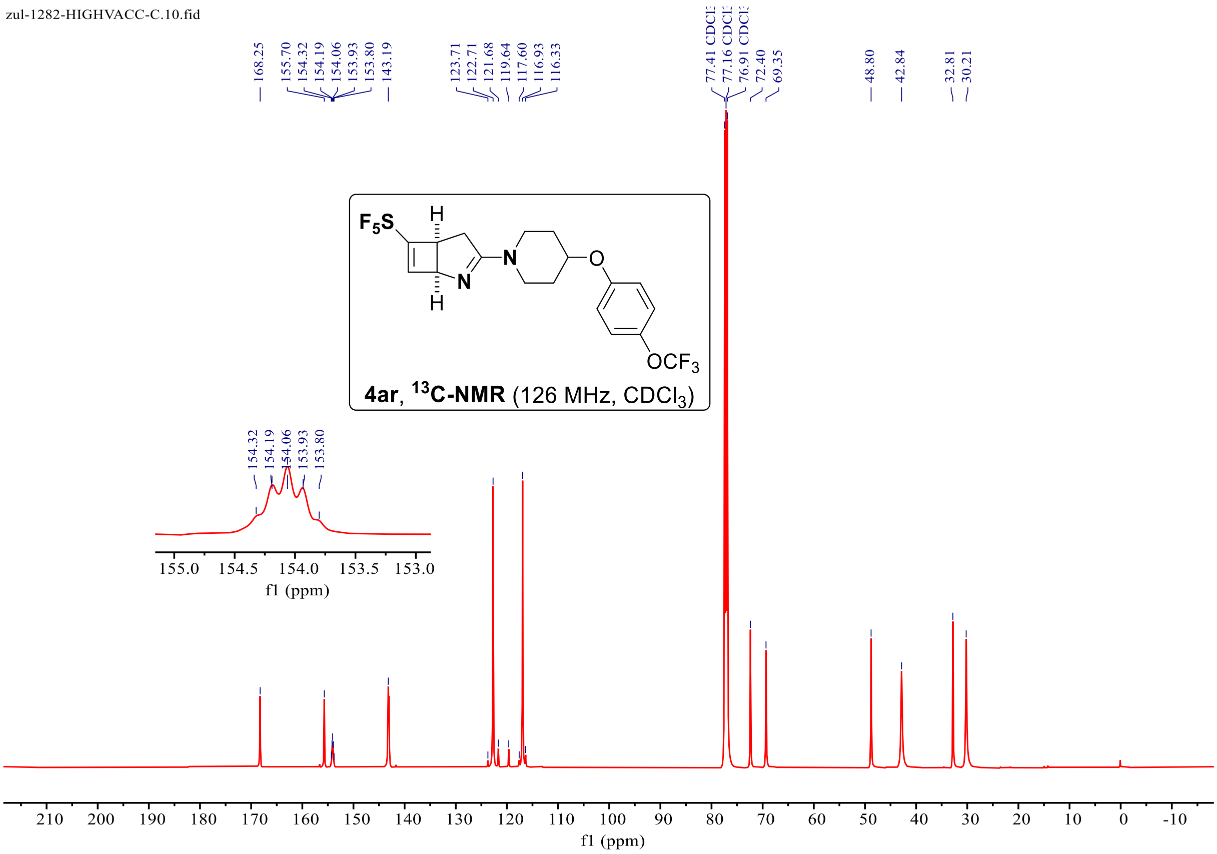
zul-1282-HIGHVACC-C.10.fid
4ar, 13C-NMR (126 MHz, CDCl3)
f1 (ppm)

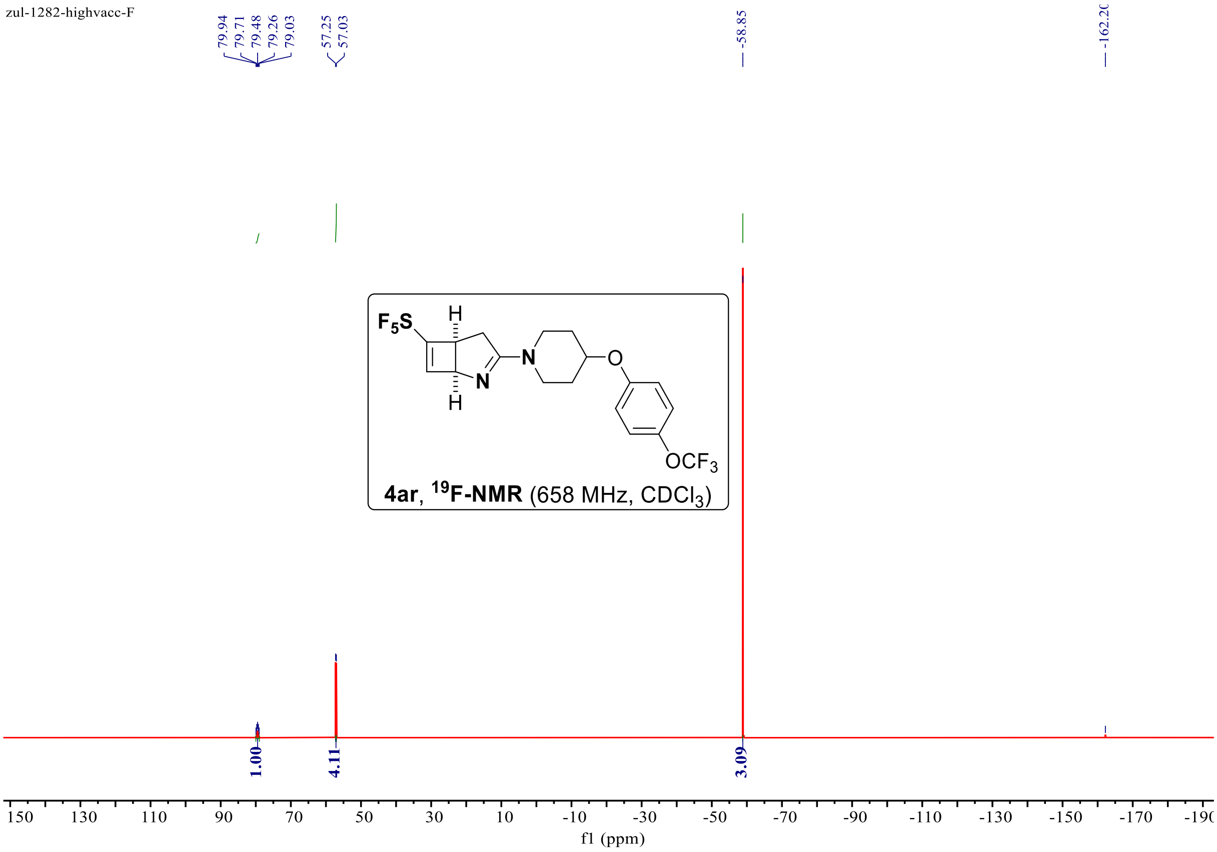
zul-1282-highvacc-F
4ar, 19F-NMR (658 MHz, CDCl3)
f1 (ppm)

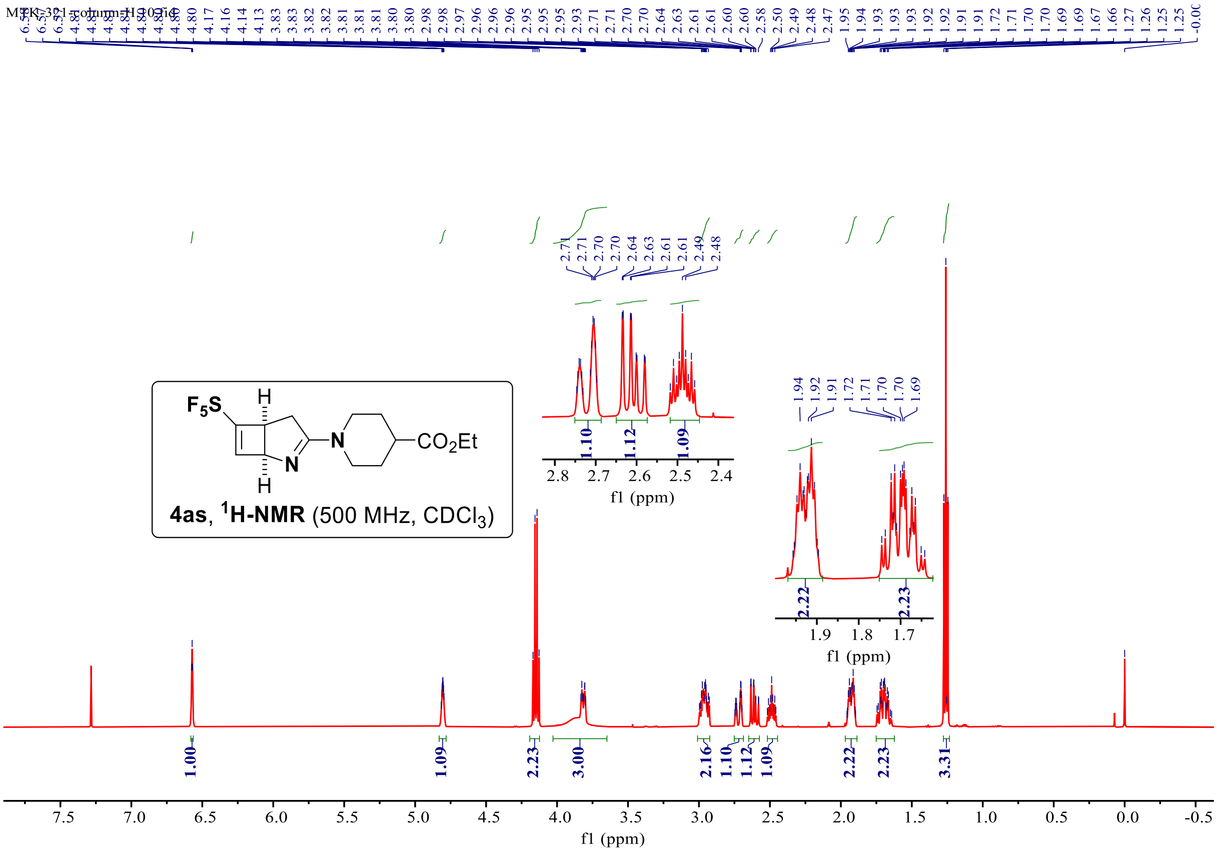
MTK-321-column-H-10.fid
4.80
4.17
4.16
4.14
4.13
3.83
3.83
3.82
3.82
3.81
3.81
3.81
3.80
3.80
2.98
2.98
2.97
2.96
2.96
2.96
2.95
2.95
2.95
2.93
2.71
2.71
2.71
2.70
2.70
2.64
2.63
2.61
2.61
2.60
2.60
2.58
2.50
2.49
2.48
2.47
1.95
1.94
1.93
1.93
1.93
1.92
1.92
1.91
1.91
1.72
1.71
1.70
1.70
1.69
1.69
1.67
1.66
1.27
1.26
1.25
1.25
-0.00
F_5S
H
N
H
N
CO_2Et
4as, ^{1}H-NMR (500 MHz, $CDCl_3$)
1.10
1.12
1.09
2.8
2.7
2.6
2.5
2.4
f1 (ppm)
1.94
1.92
1.91
1.72
1.71
1.70
1.70
1.69
2.22
2.23
1.9
1.8
1.7
f1 (ppm)
1.00
1.09
2.23
3.00
2.16
1.10
1.12
1.09
2.22
2.23
3.31
7.5
7.0
6.5
6.0
5.5
5.0
4.5
4.0
3.5
3.0
2.5
2.0
1.5
1.0
0.5
0.0
-0.5
f1 (ppm)

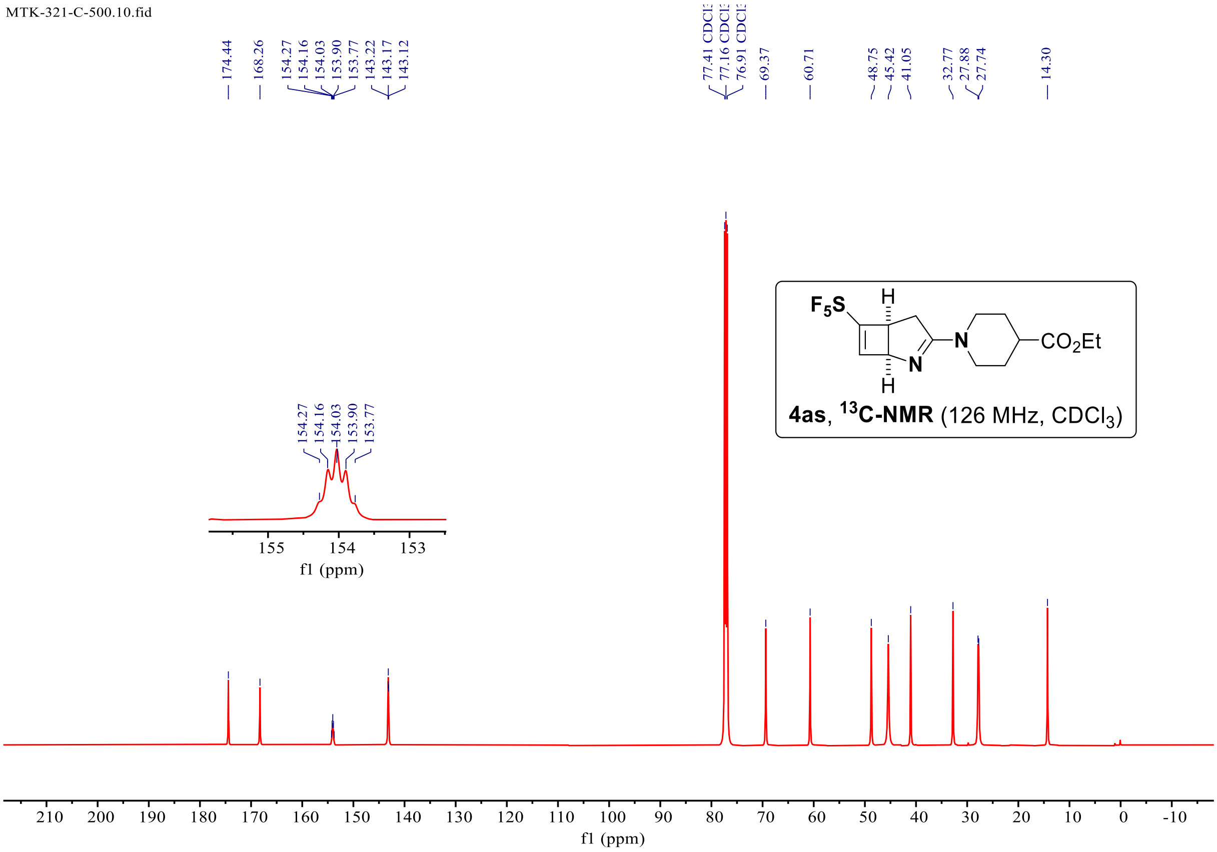
MTK-321-C-500.10.fid
174.44
168.26
154.27
154.16
154.03
153.90
153.77
143.22
143.17
143.12
77.41 CDCl3
77.16 CDCl3
76.91 CDCl3
69.37
60.71
48.75
45.42
41.05
32.77
27.88
27.74
14.30
F_5S
H
N
H
N
CO_2Et
4as, ^{13}C-NMR (126 MHz, $CDCl_3$)
154.27
154.16
154.03
153.90
153.77
155
154
153
f1 (ppm)
210
200
190
180
170
160
150
140
130
120
110
100
90
80
70
60
50
40
30
20
10
0
-10
f1 (ppm)

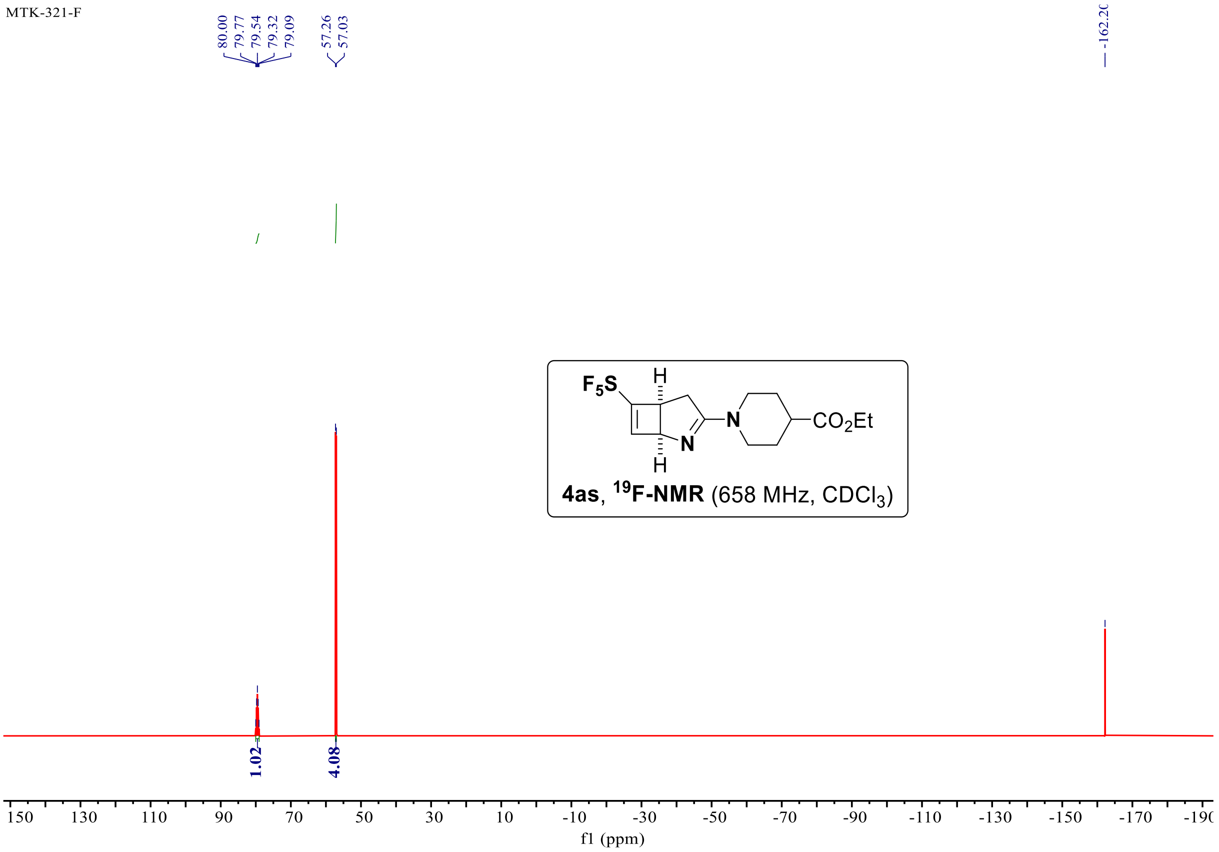
MTK-321-F
80.00
79.77
79.54
79.32
79.09
57.26
57.03
-162.20
4as, 19F-NMR (658 MHz, CDCl3)
1.02
4.08
f1 (ppm)

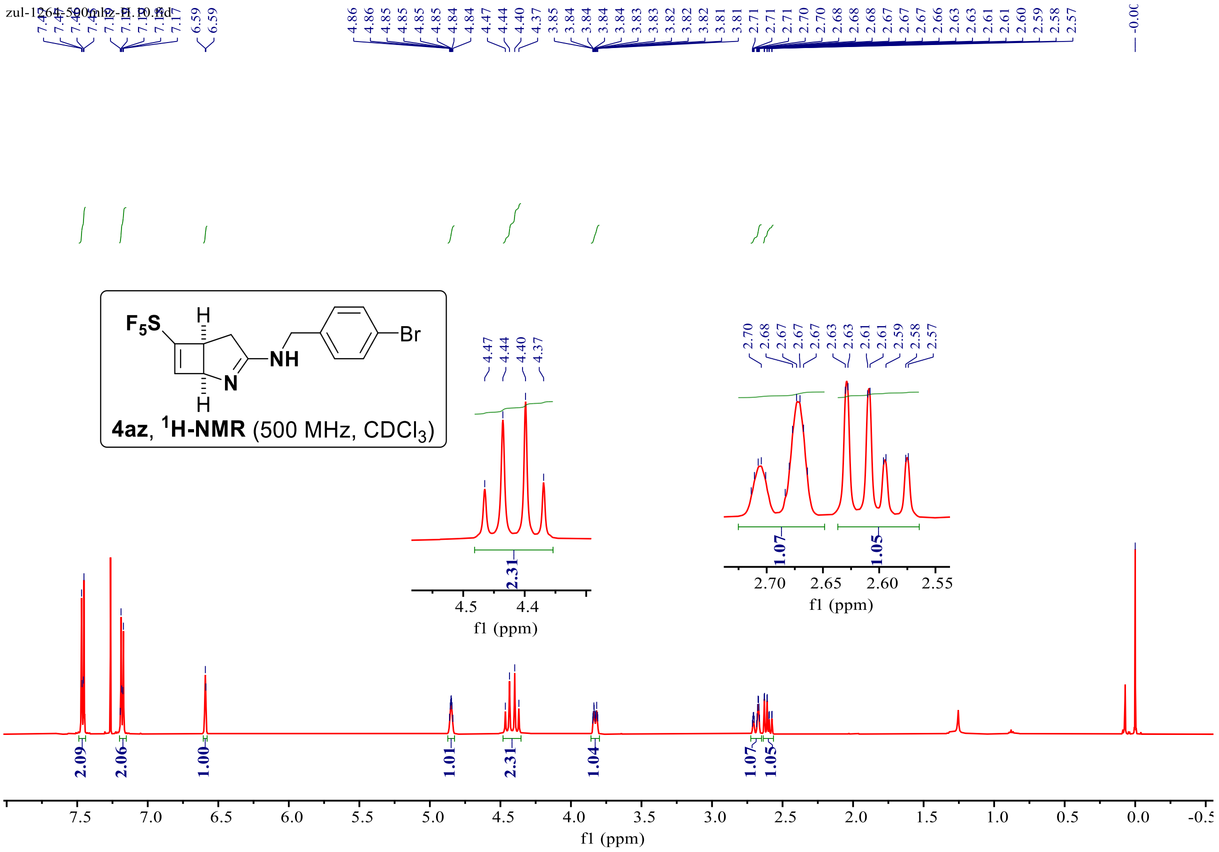
4az, 1H-NMR (500 MHz, CDCl3)
2.09
2.06
1.00
1.01
2.31
1.04
1.07
1.05
f1 (ppm)

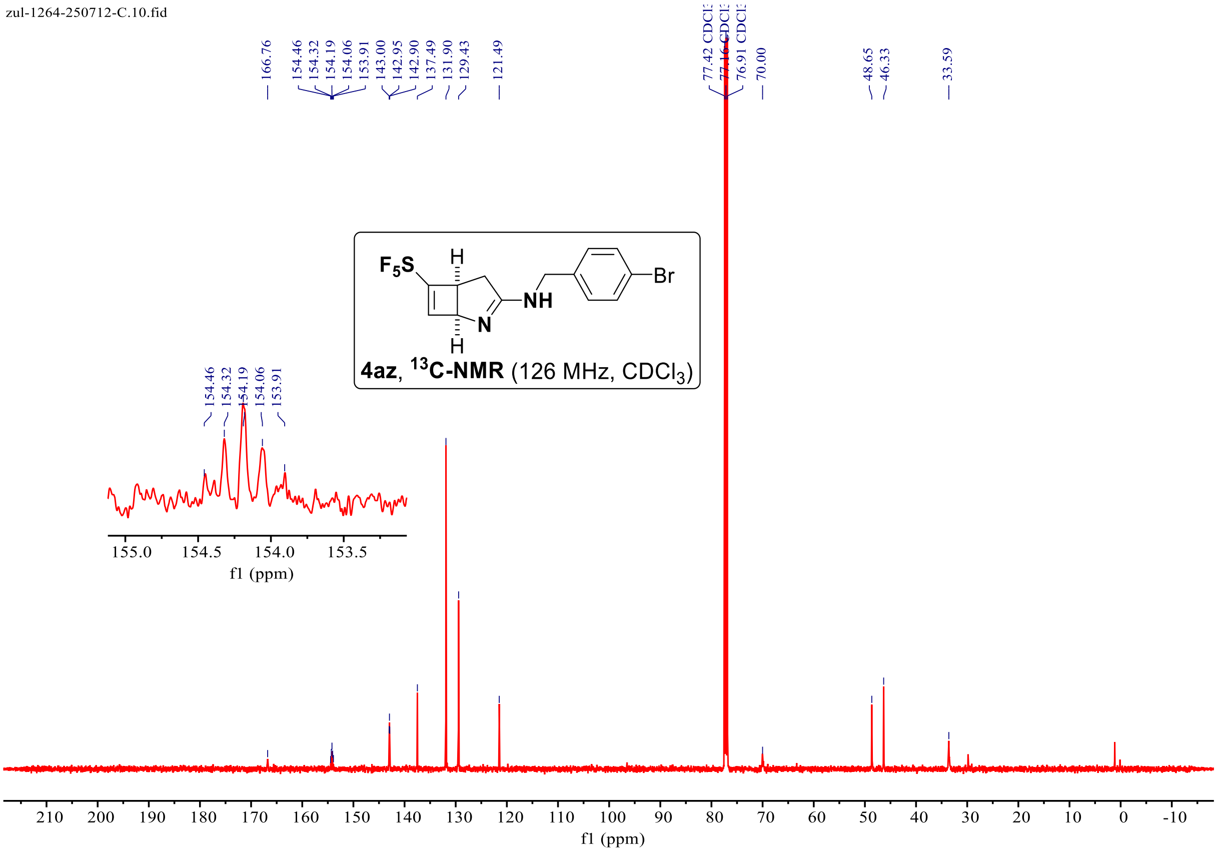
zul-1264-250712-C.10.fid
166.76
154.46
154.32
154.19
154.06
153.91
143.00
142.95
142.90
137.49
131.90
129.43
121.49
77.42 CDCl3
77.16 CDCl3
76.91 CDCl3
70.00
48.65
46.33
33.59
F5S
H
NH
Br
N
H
4az, 13C-NMR (126 MHz, CDCl3)
f1 (ppm)

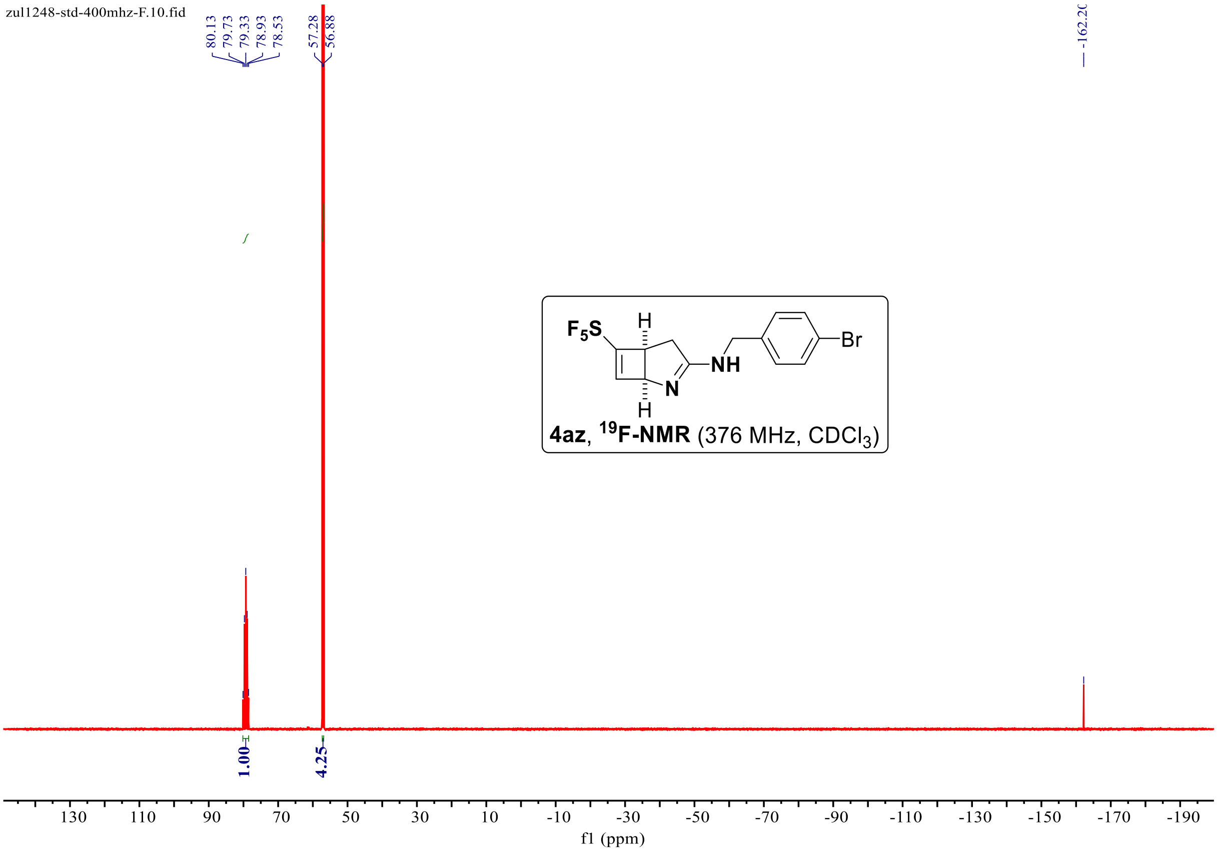
zul1248-std-400mhz-F.10.fid
80.13
79.73
79.33
78.93
78.53
57.28
56.88
-162.20
F5S
H
NH
Br
N
H
4az, 19F-NMR (376 MHz, CDCl3)
1.00
4.25
f1 (ppm)

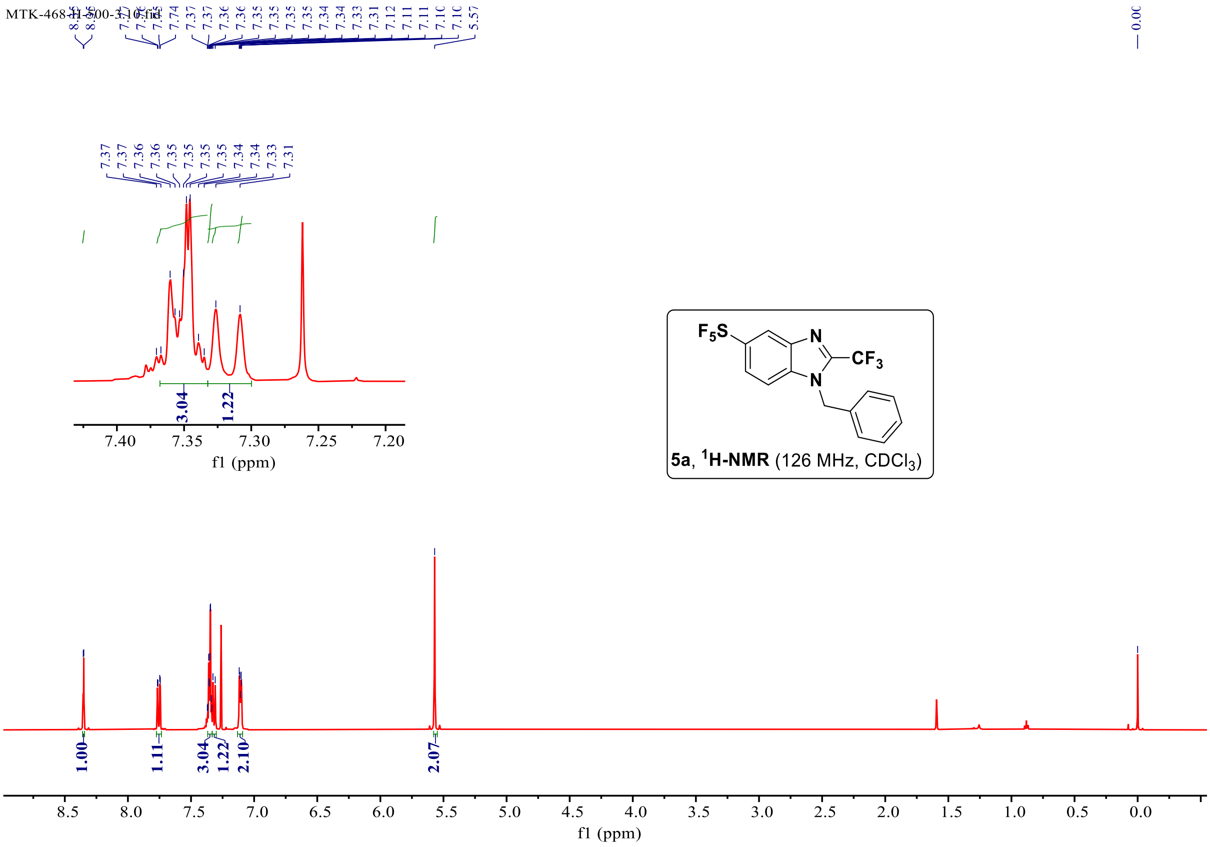
MTK-468-f1-500-3.10.fid
5a, ¹H-NMR (126 MHz, CDCl₃)
3.04
1.22
1.00
1.11
2.10
2.07
f1 (ppm)

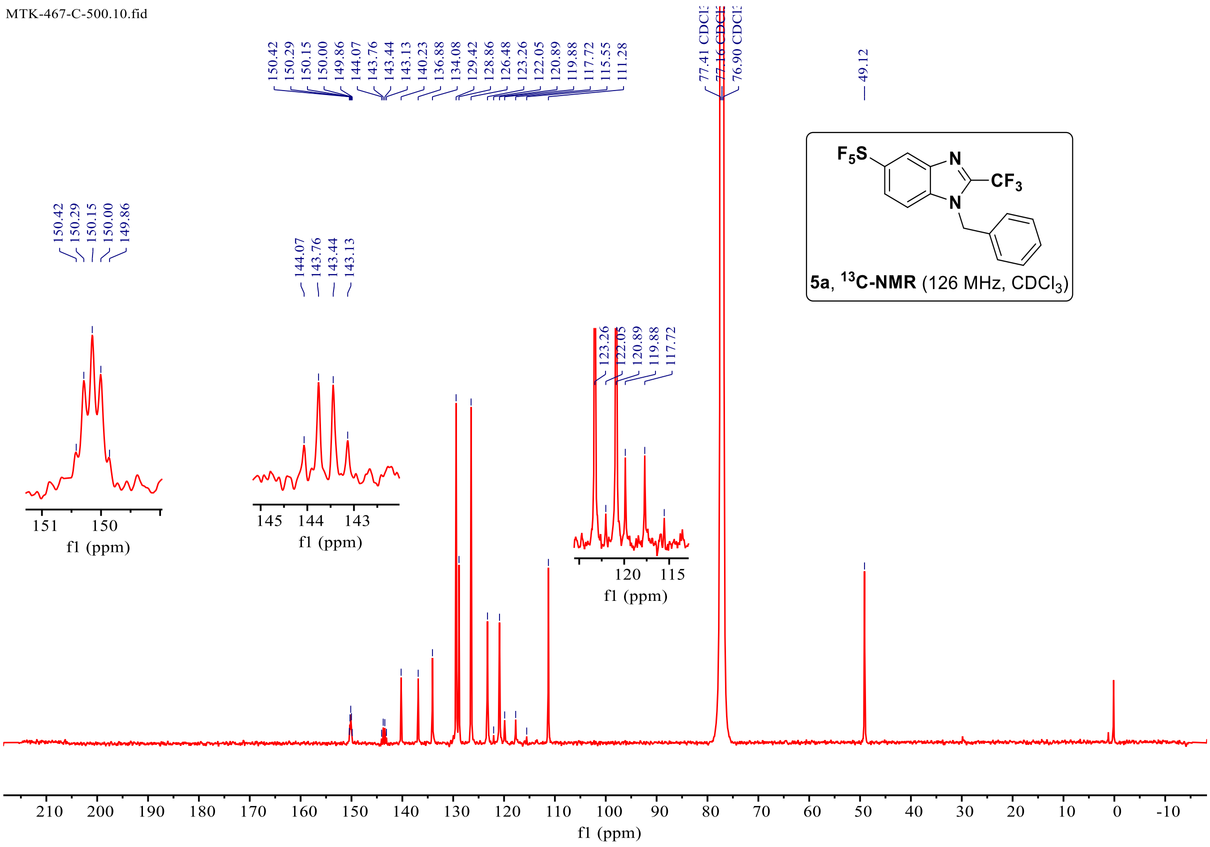
MTK-467-C-500.10.fid
150.42
150.29
150.15
150.00
149.86
144.07
143.76
143.44
143.13
140.23
136.88
134.08
129.42
128.86
126.48
123.26
122.05
120.89
119.88
117.72
115.55
111.28
76.90 CDCl3
49.12
5a, ¹³C-NMR (126 MHz, CDCl₃)
f1 (ppm)

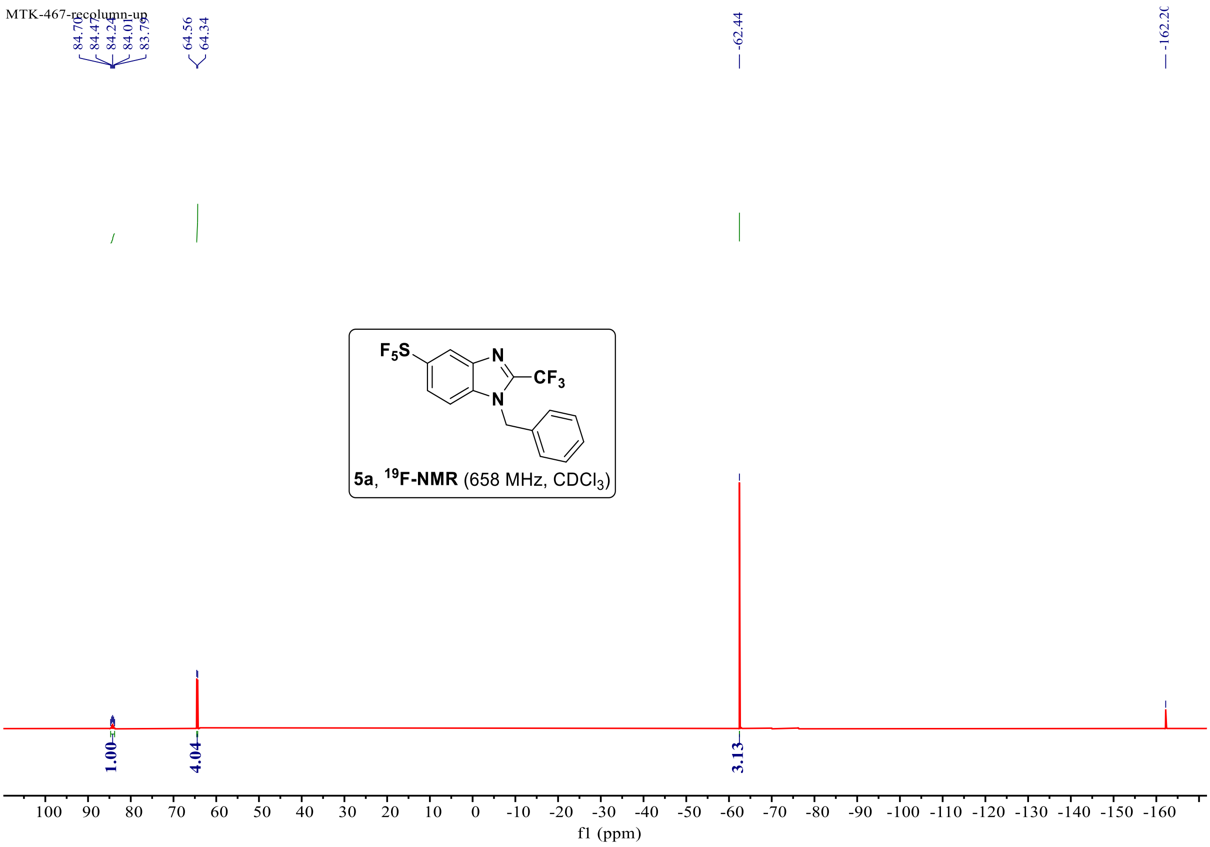
MTK-467-recolumn-up
5a, 19F-NMR (658 MHz, CDCl3)
f1 (ppm)

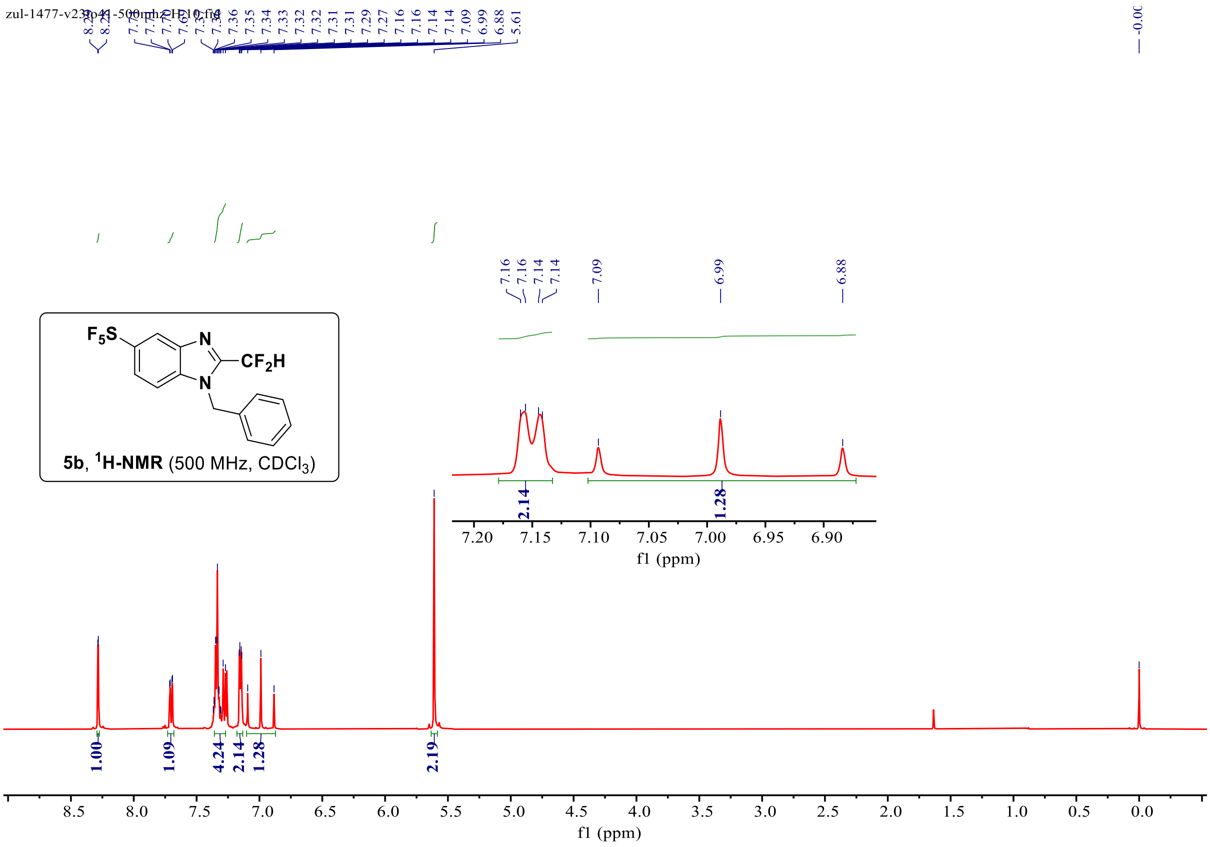
5b, 1H-NMR (500 MHz, CDCl3)
f1 (ppm)

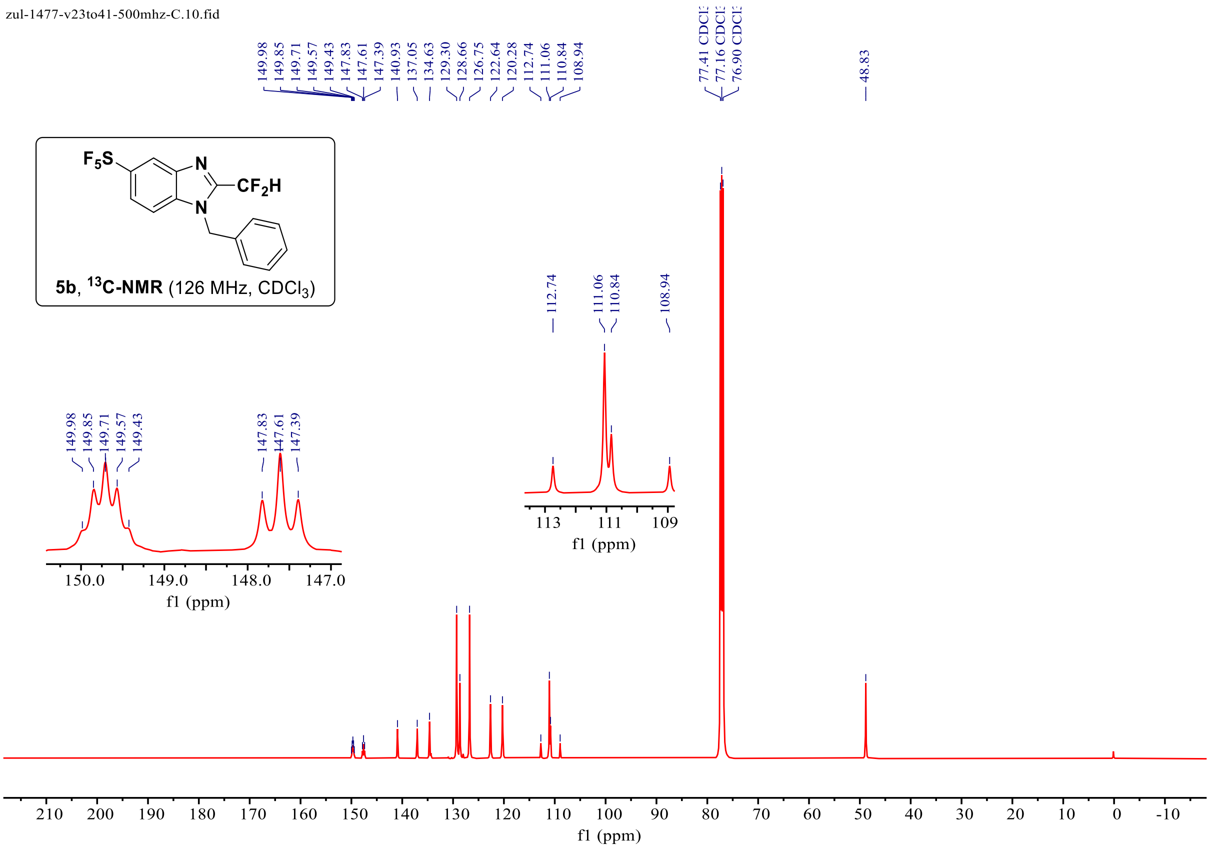

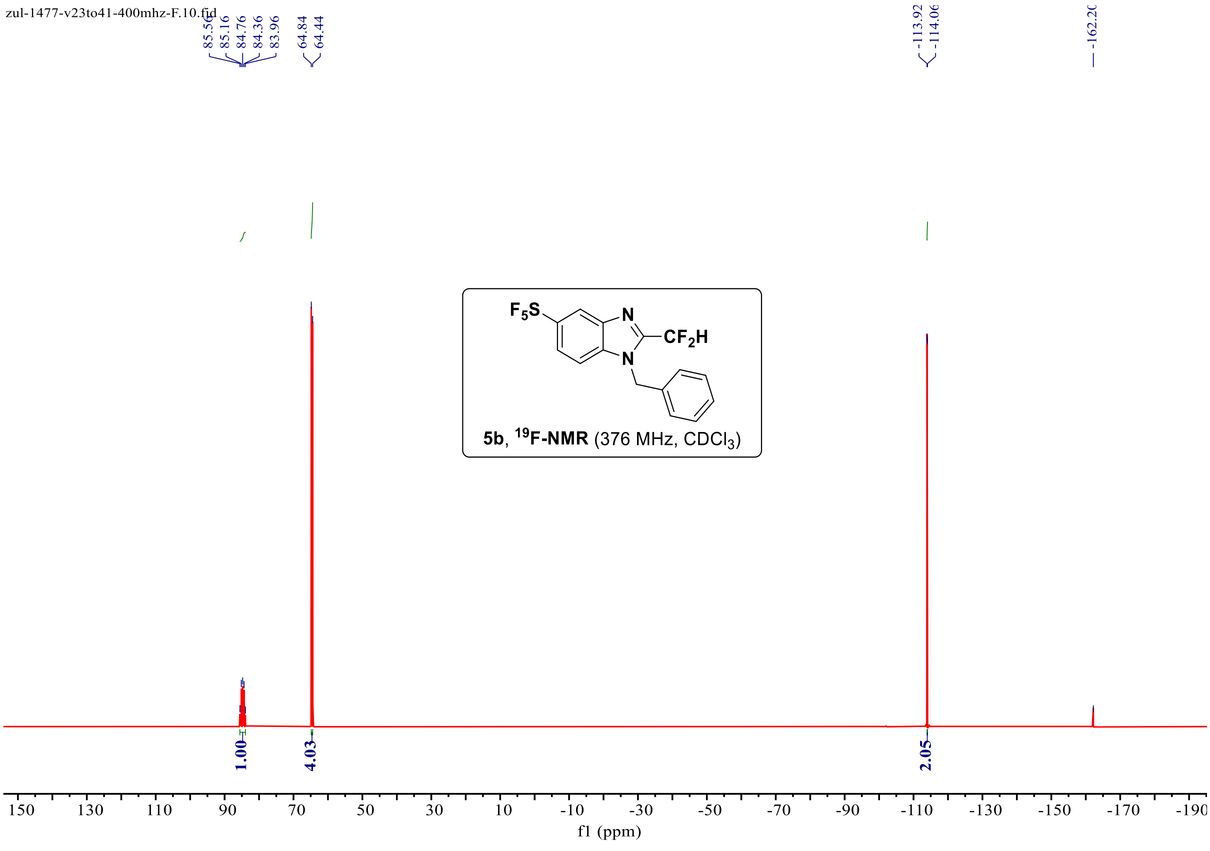

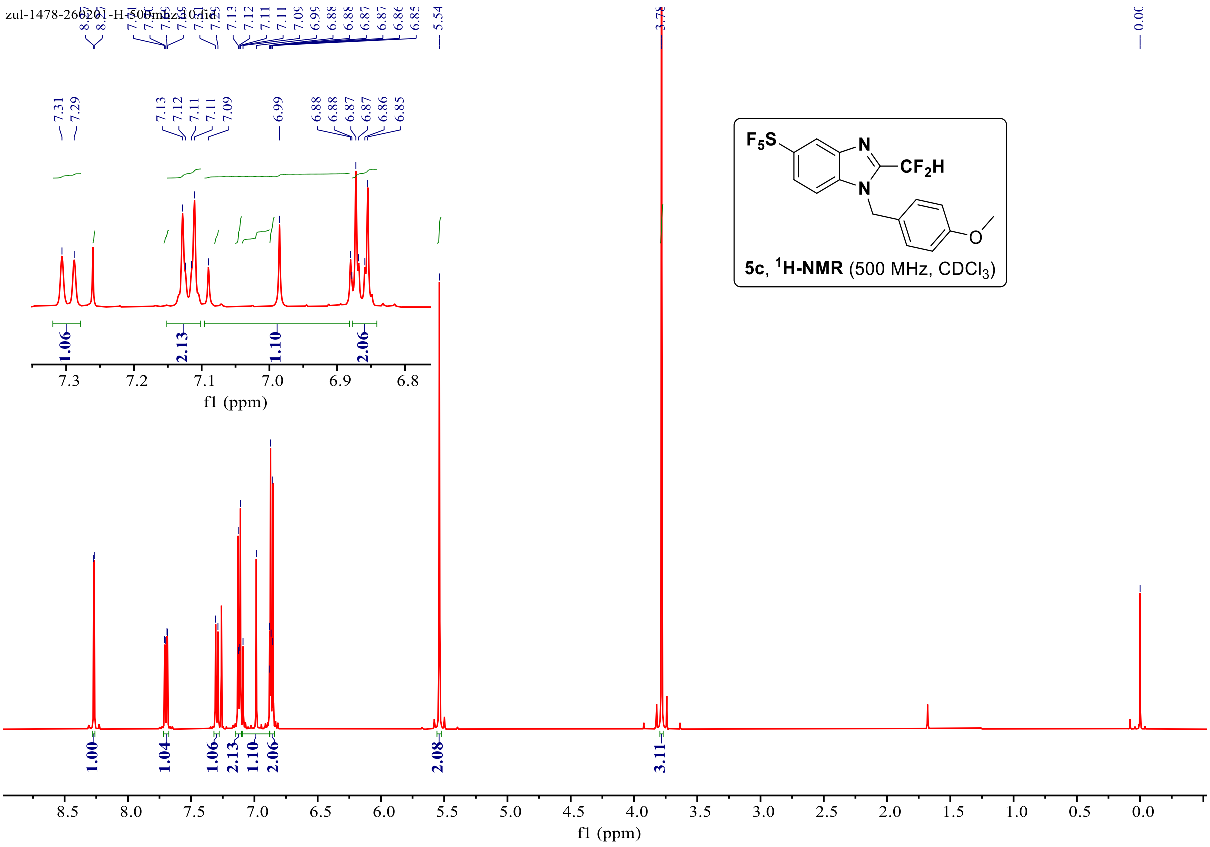
zul-1478-260201-H-500mhz.10.fid
5c, 1H-NMR (500 MHz, CDCl3)
f1 (ppm)

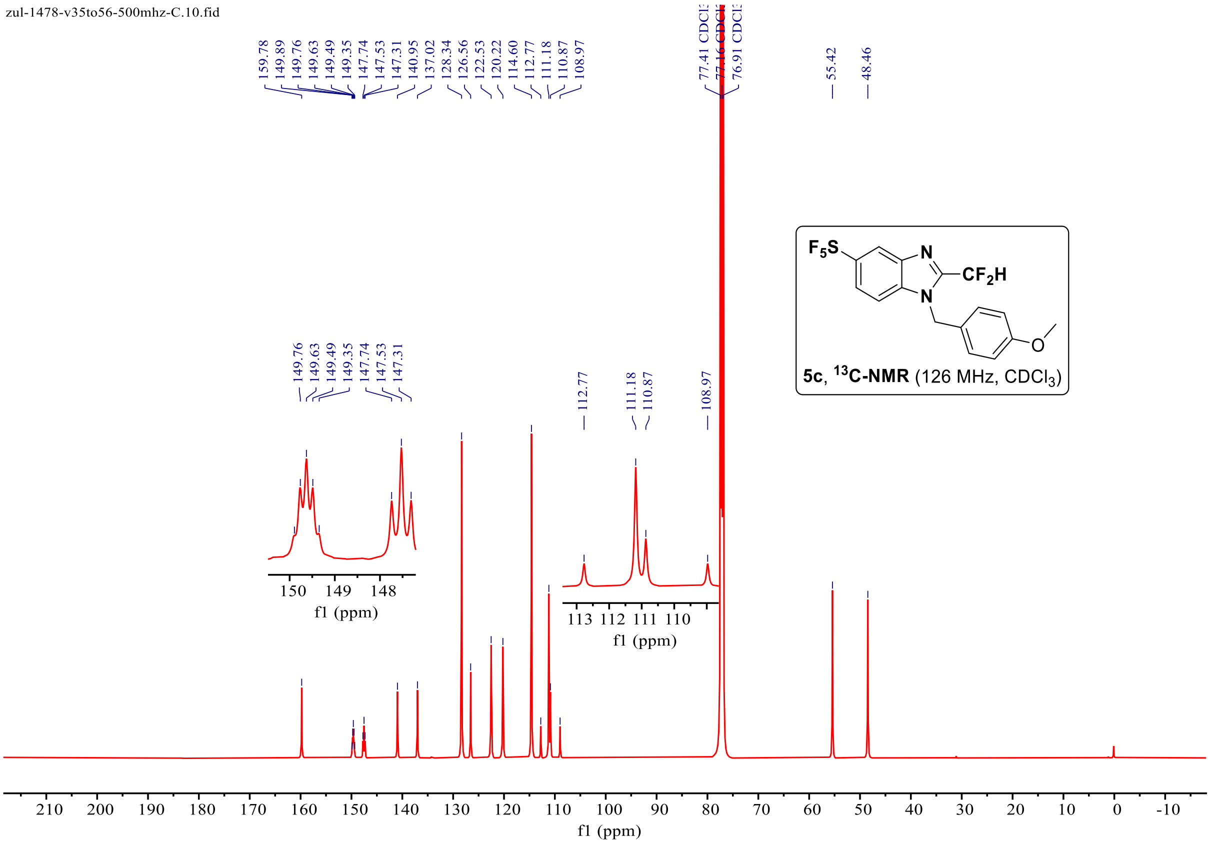
zul-1478-v35to56-500mhz-C.10.fid
5c, 13C-NMR (126 MHz, CDCl3)
f1 (ppm)

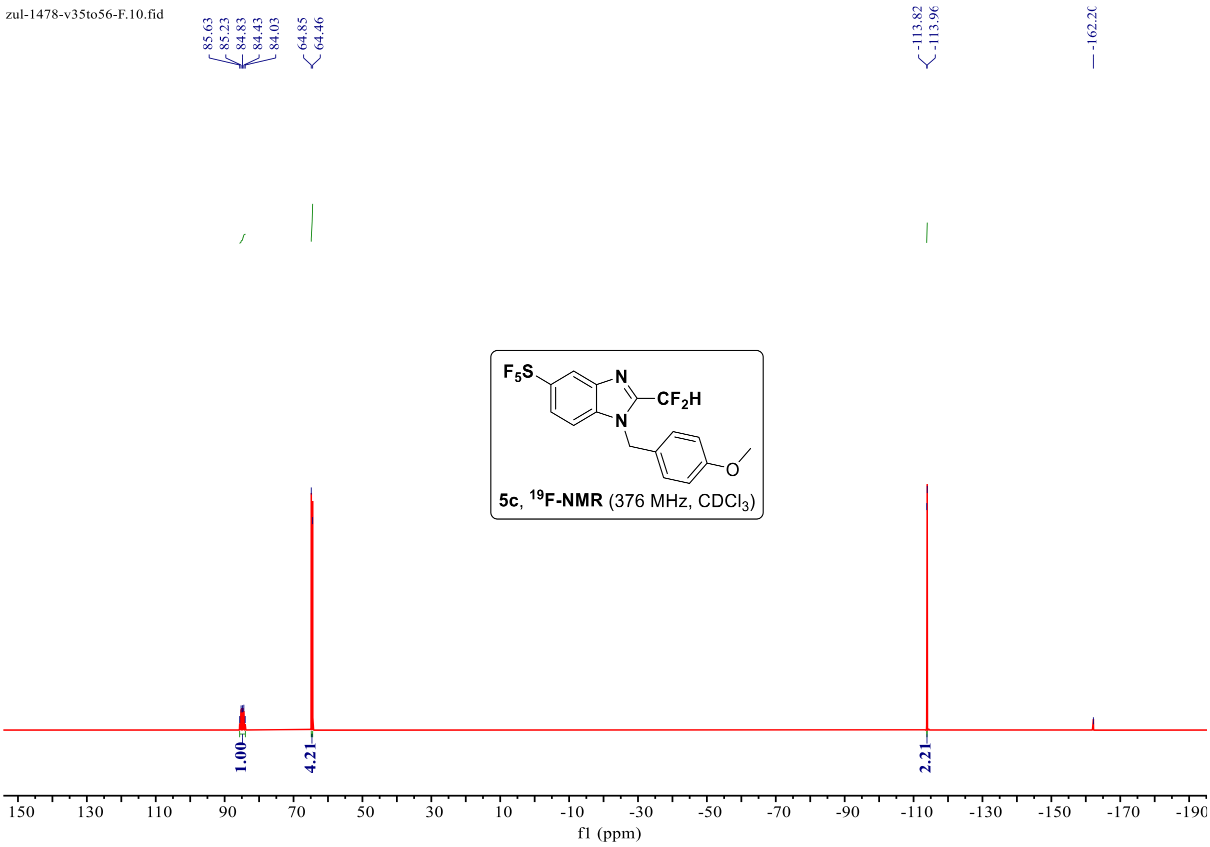

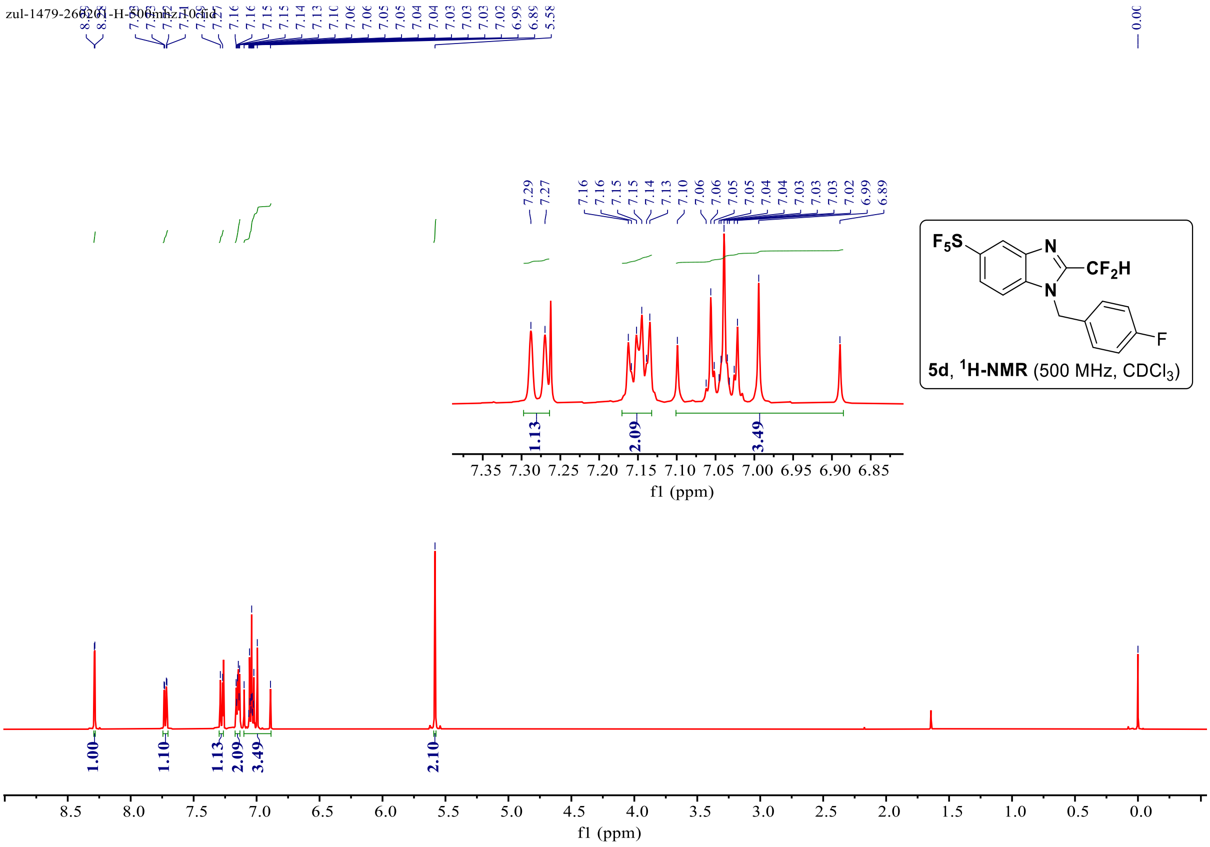

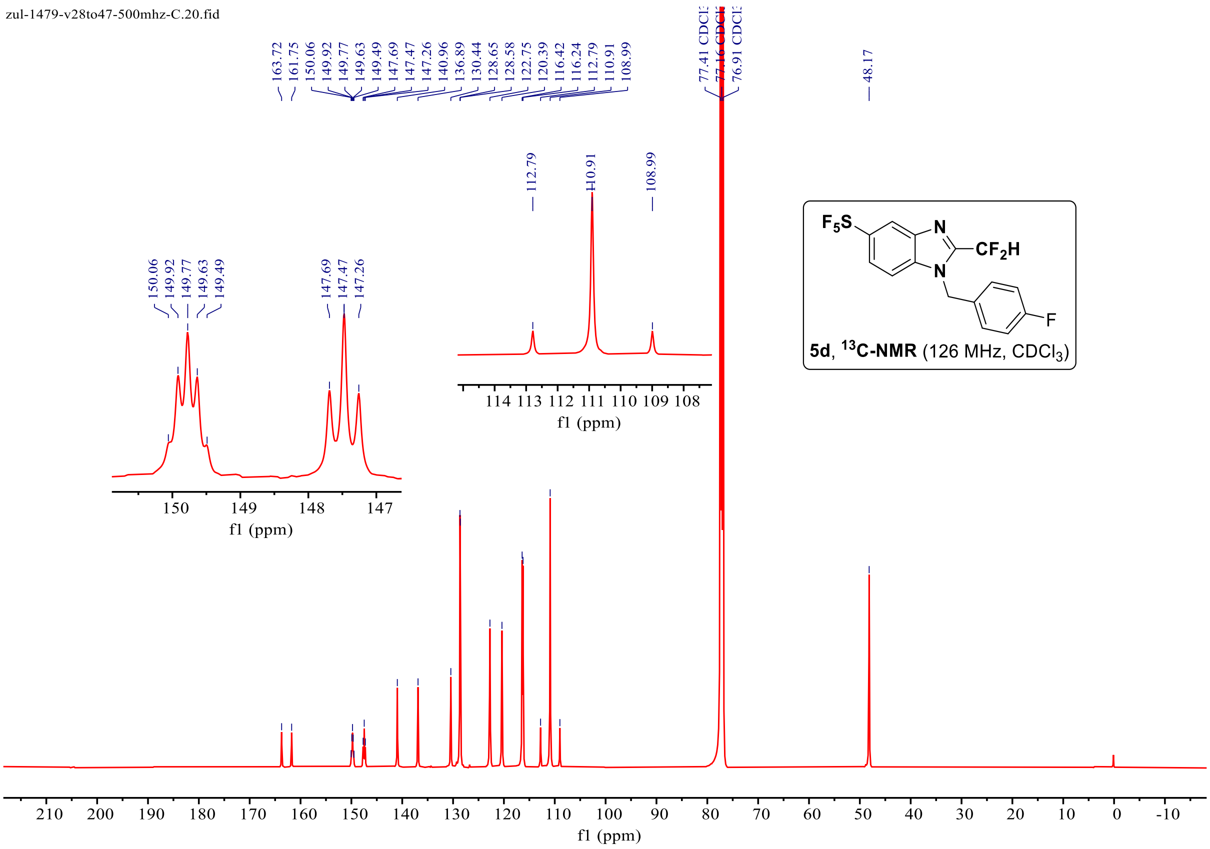
zul-1479-v28to47-500mhz-C.20.fid
163.72
161.75
150.06
149.92
149.77
149.63
149.49
147.69
147.47
147.26
140.96
136.89
130.44
128.65
128.58
122.75
120.39
116.42
116.24
112.79
110.91
108.99
77.41 CDCl3
77.16 CDCl3
76.91 CDCl3
48.17
F5S
N
CF2H
N
F
5d, 13C-NMR (126 MHz, CDCl3)
f1 (ppm)

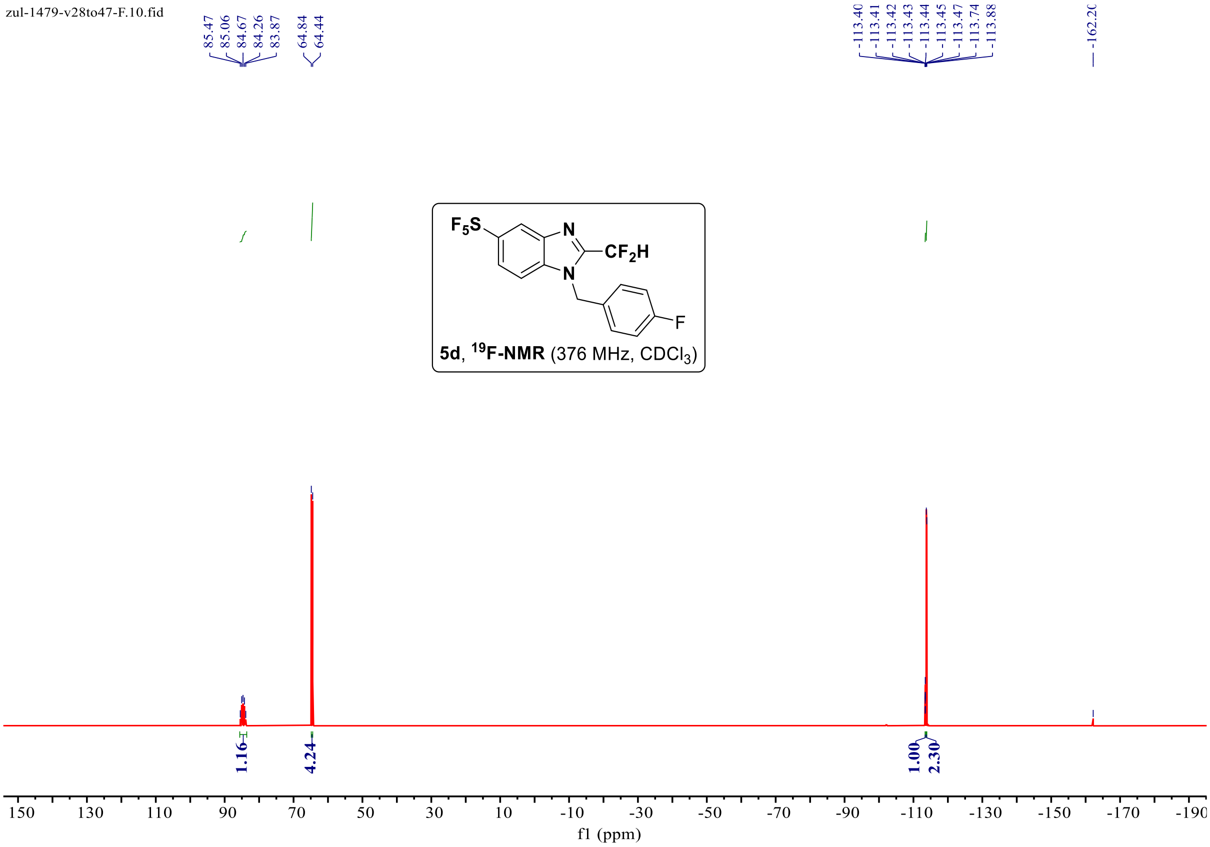
zul-1479-v28to47-F.10.fid
85.47
85.06
84.67
84.26
83.87
64.84
64.44
-113.40
-113.41
-113.42
-113.43
-113.44
-113.45
-113.47
-113.74
-113.88
-162.20
F5S
N
CF2H
N
F
5d, 19F-NMR (376 MHz, CDCl3)
1.16
4.24
1.00
2.30
f1 (ppm)

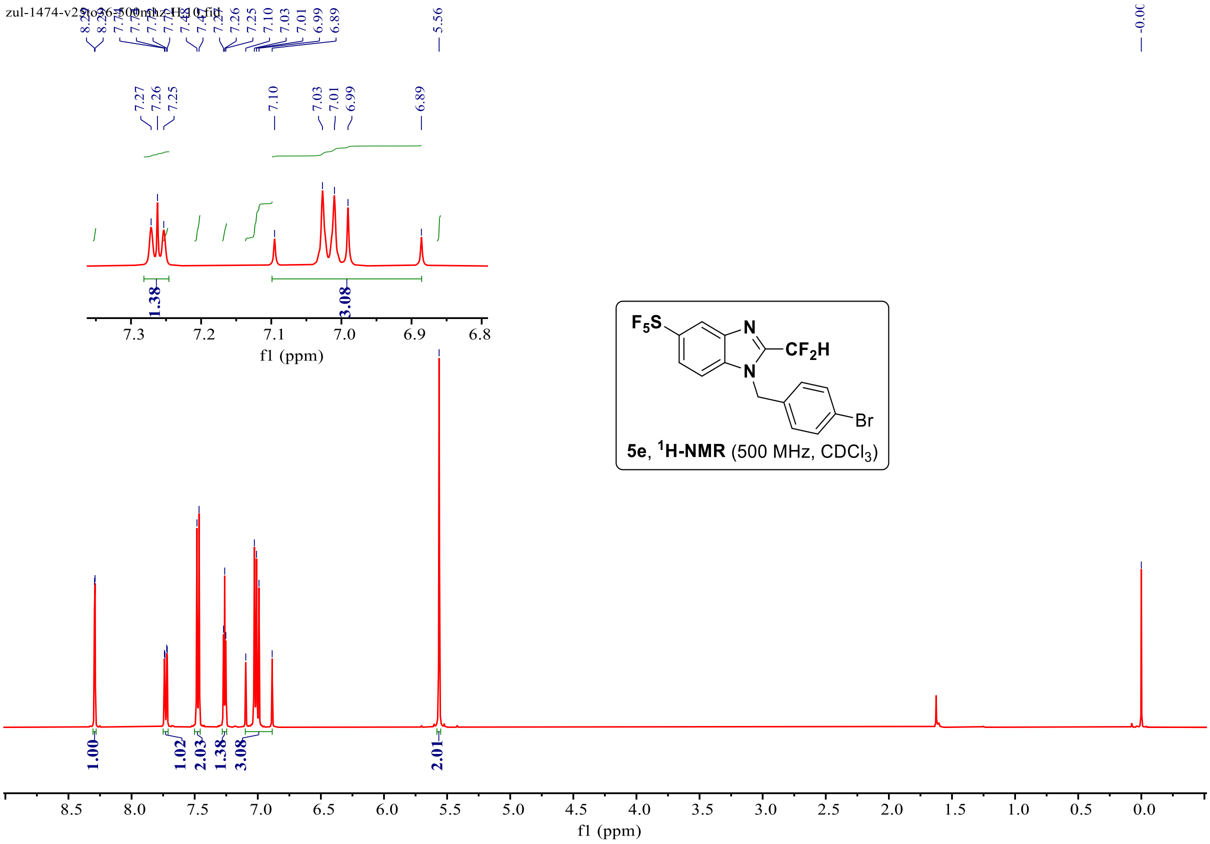
zul-1474-v25to36-500mhz-H.10.fid
5.56
-0.00
7.27
7.26
7.25
7.10
7.03
7.01
6.99
6.89
1.38
3.08
f1 (ppm)
F5S
N
CF2H
N
Br
5e, 1H-NMR (500 MHz, CDCl3)
1.00
1.02
2.03
1.38
3.08
2.01
f1 (ppm)

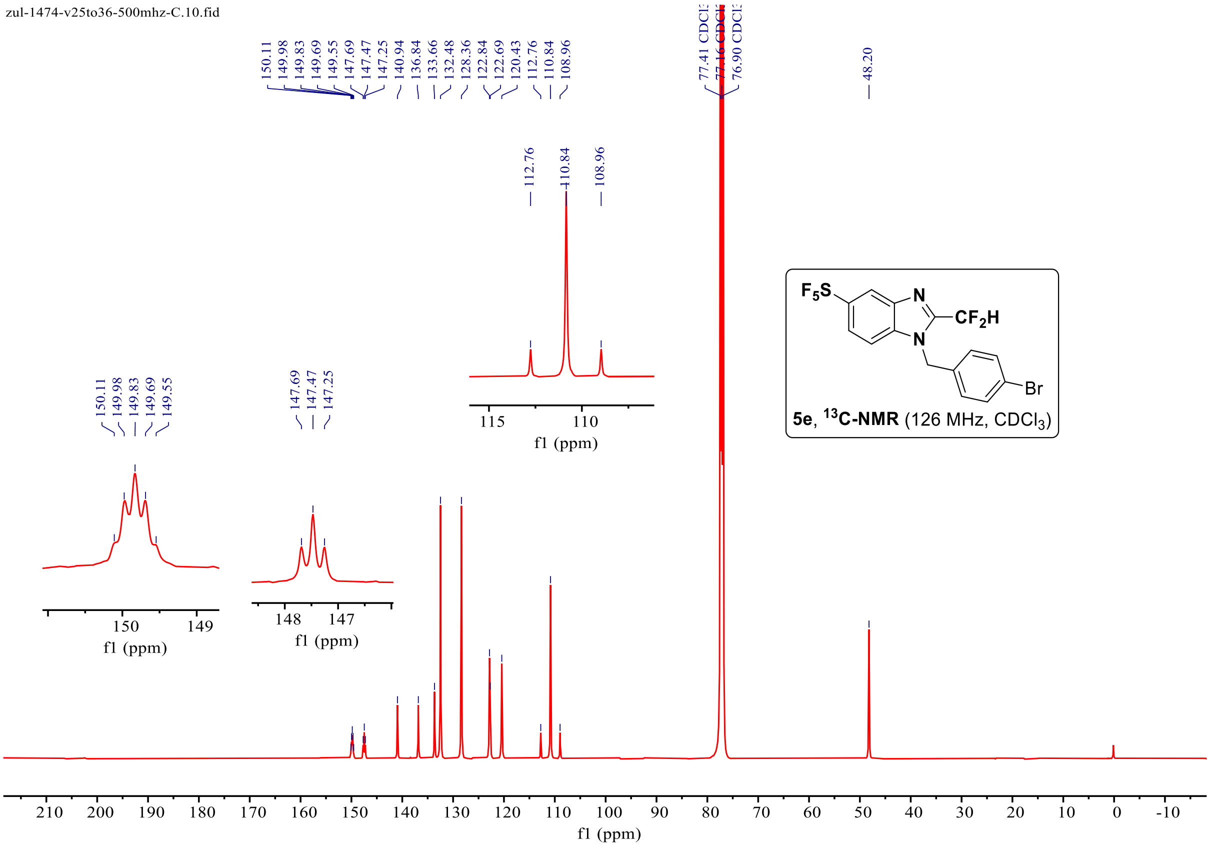
zul-1474-v25to36-500mhz-C.10.fid
150.11
149.98
149.83
149.69
149.55
147.69
147.47
147.25
140.94
136.84
133.66
132.48
128.36
122.84
122.69
120.43
112.76
110.84
108.96
77.41 CDCl3
77.16 CDCl3
76.90 CDCl3
48.20
f1 (ppm)
F5S
N
CF2H
N
Br
5e, 13C-NMR (126 MHz, CDCl3)
f1 (ppm)

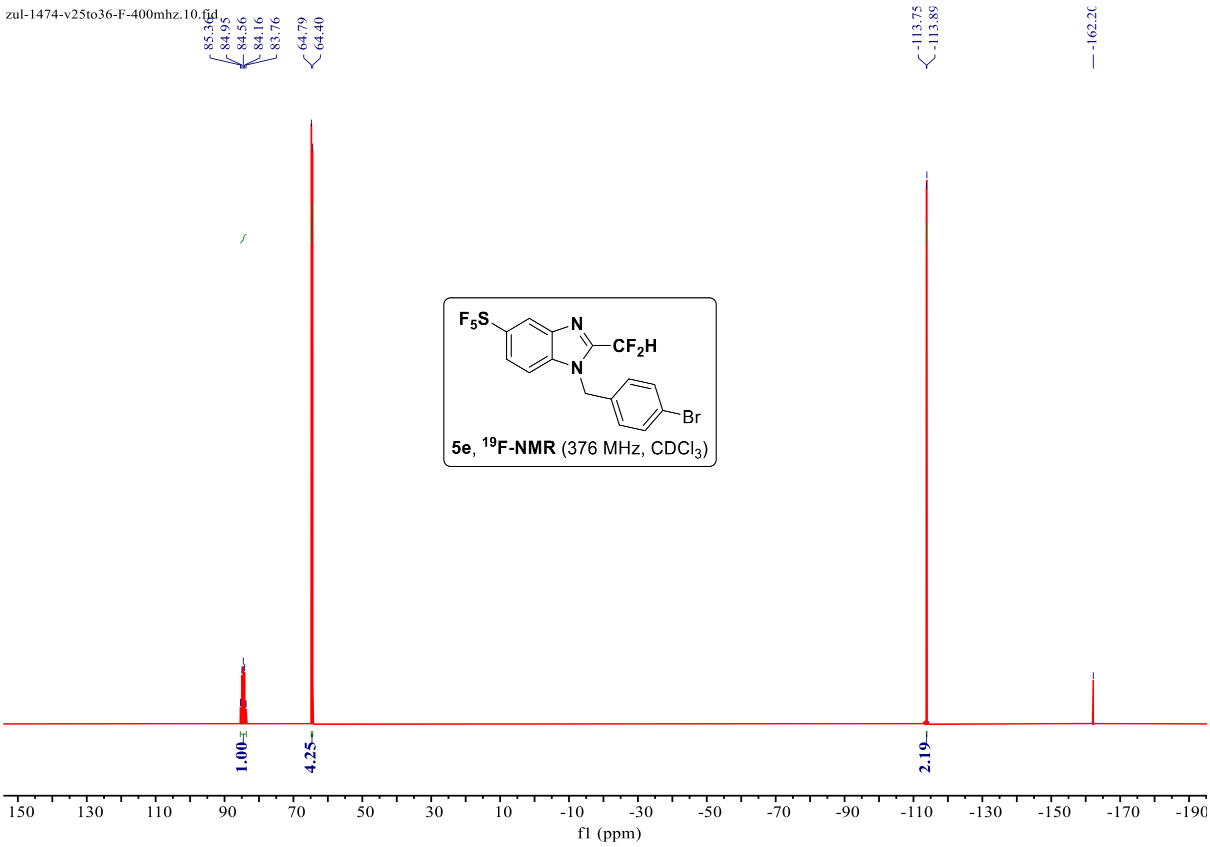
zul-1474-v25to36-F-400mhz.10.fid
85.36 84.95 84.56 84.16 83.76 64.79 64.40
-113.75 -113.89
-162.20
1.00
4.25
2.19
f1 (ppm)
5e, 19F-NMR (376 MHz, CDCl3)

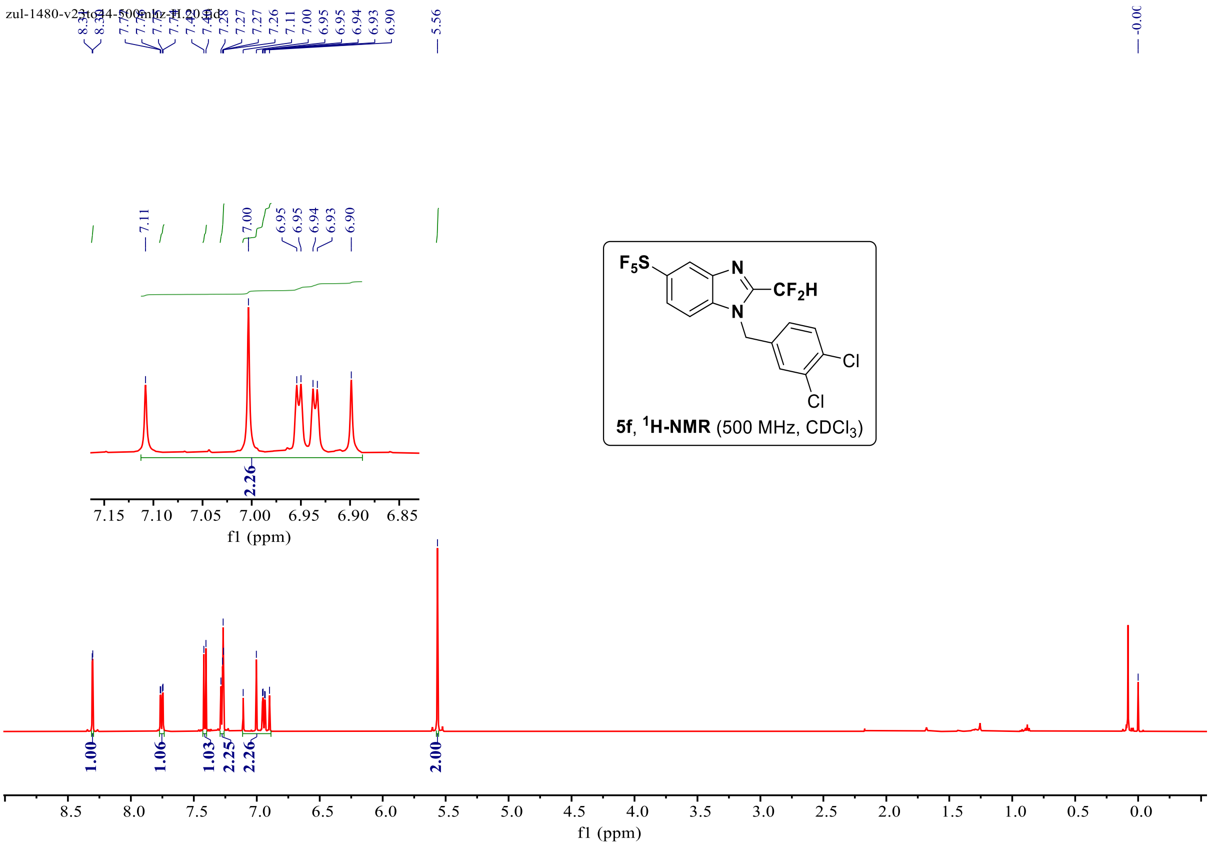
7.27 7.27 7.26 7.11 7.00 6.95 6.95 6.94 6.93 6.90
5.56
-0.00
2.26
f1 (ppm)
1.00
1.06
1.03
2.25
2.26
2.00
5f, 1H-NMR (500 MHz, CDCl3)

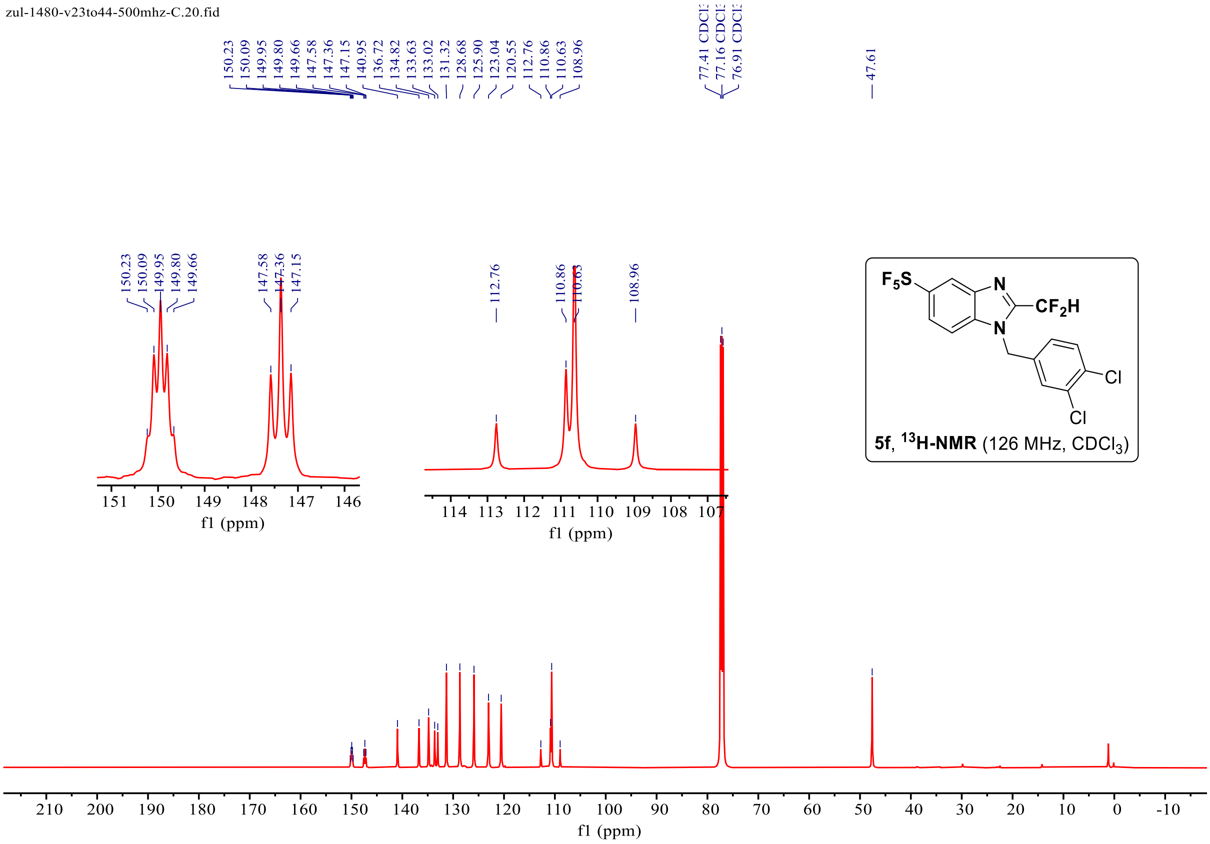
zul-1480-v23to44-500mhz-C.20.fid
150.23 150.09 149.95 149.80 149.66 147.58 147.36 147.15 140.95 136.72 134.82 133.63 133.02 131.32 128.68 125.90 123.04 120.55 112.76 110.86 110.63 108.96
77.41 CDCl3 77.16 CDCl3 76.91 CDCl3
47.61
F5S
N
CF2H
N
Cl
Cl
5f, 13H-NMR (126 MHz, CDCl3)
f1 (ppm)

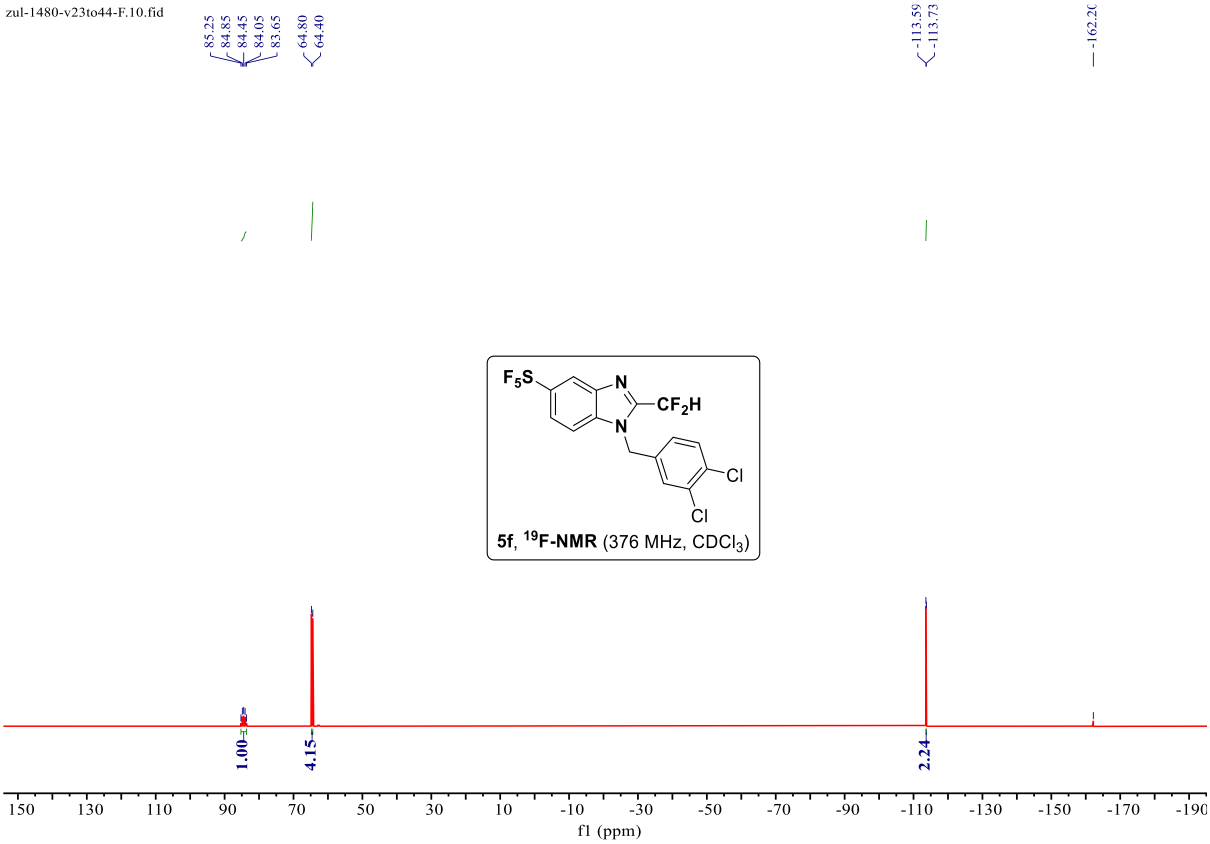
zul-1480-v23to44-F.10.fid
85.25 84.85 84.45 84.05 83.65
64.80 64.40
-113.59 -113.73
-162.20
F5S
N
CF2H
N
Cl
Cl
5f, 19F-NMR (376 MHz, CDCl3)
1.00
4.15
2.24
f1 (ppm)
